# A Unified Approach to Phytosiderophore Natural Products

**DOI:** 10.1002/chem.202004004

**Published:** 2020-10-28

**Authors:** Nicolas Kratena, Tobias Gökler, Lara Maltrovsky, Eva Oburger, Christian Stanetty

**Affiliations:** ^1^ Institute of Applied Synthetic Chemistry TU Wien Getreidemarkt 9 1060 Vienna Austria; ^2^ Institute of Soil Research BOKU Vienna Konrad-Lorenz-Strasse 24 3430 Tulln Austria

**Keywords:** micronutrients, mugineic acid, natural products, phytosiderophores, total synthesis

## Abstract

This work reports on the concise total synthesis of eight natural products of the mugineic acid and avenic acid families (phytosiderophores). An innovative „east‐to‐west“ assembly of the trimeric products resulted in a high degree of divergence enabling the formation of the final products in just 10 or 11 steps each with a minimum of overall synthetic effort. Chiral pool starting materials (l‐malic acid, threonines) were employed for the outer building blocks while the middle building blocks were accessed by diastereo‐ and enantioselective methods. A highlight of this work consists in the straightforward preparation of epimeric hydroxyazetidine amino acids, useful building blocks on their own, enabling the first synthesis of 3’’‐hydroxymugineic acid and 3’’‐hydroxy‐2’‐deoxymugineic acid.

## Graphical abstract

**Phytosiderophores** are natural products enabling plants to absorb essential metal ions from the soil. Until now only specific solutions towards particular phytosiderophores had been reported. In this work, the entire family of these compounds is made synthetically accessible by using a divergent synthetic strategy.

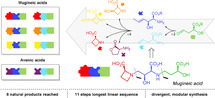

Micronutrient acquisition is an important factor in growth and survival of any living organism. Plants are stationary and need to fill all their needs from the soil they grow in. In calcareous, high pH soils, the solubility and therefore plant availability of some of these crucial metal ions, like iron, zinc and copper, is diminished, to a degree that it greatly inhibits plant growth and leads to chlorosis of the leaves.[^1^, ^2^] On top of reduced yield, micronutrient deficiencies in major crops like wheat, barley, rice and maize carry over to local consumers, causing micronutrient deficiency in humans („hidden hunger“) with severe negative effects on child growth, development and disease resistance.^[3]^

A unique strategy used by gramineous plants for the uptake of Fe and potentially also Zn and Cu relies on phytosiderophores (PS), which are multidentate chelators of metal ions. PS are exuded by roots of grass species into the surrounding soil (i.e. rhizosphere) where they can complex Fe^III^ ions from soil particles.^[4]^ The PS–Fe^III^ 1:1 complex is then taken up as whole complex and the iron liberated within the cell. This complexation strategy renders grass species more efficient in Fe acquisition compared to non‐grass species, particularly in high pH soil, where soluble Fe concentrations are low. Investigations into plants molecular mechanisms, and consequently efforts towards the synthesis of these natural products have been reported since the late 1970s.[^5a^, ^5b^, ^5c^, ^5d^, ^5e^, ^5f^, ^5g^, ^5h^]

Variation in naturally occurring PS (see Figure 1) arises from hydroxy groups present on C‐2 of the western (left) and middle subunits, respectively, while the eastern hydroxyacid fragment is conserved throughout the mugineic, avenic and distichonic acid series. The general synthetic strategy is closely related to the biosynthesis^[6]^ and specific synthetic solutions were published for avenic acid A (AVA, **I**), deoxymugineic acid (DMA, **IV**), mugineic acid (MA, **VIII**) and 3’’‐epi‐hydroxy‐MA (**VI**).

**Figure 1:**
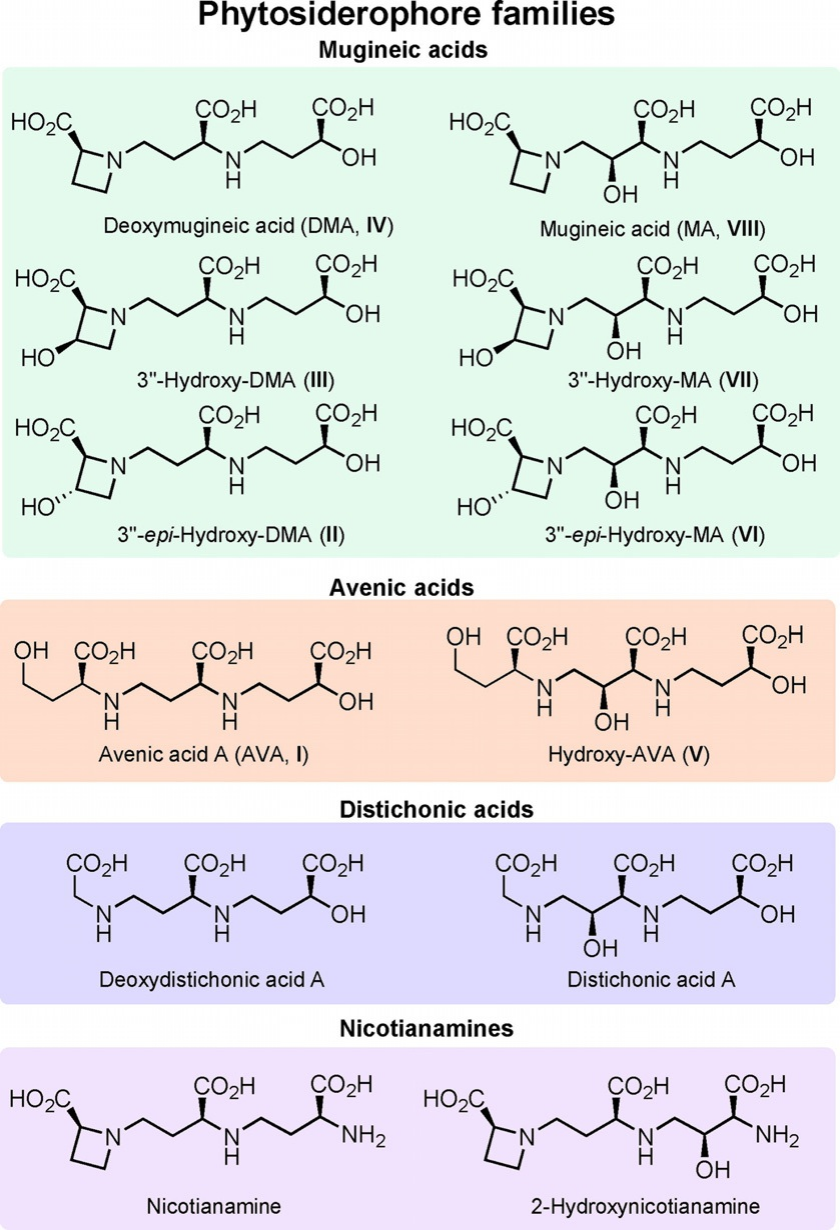
Phytosiderophore natural product families.

In the state‐of‐the‐art mugineic acid synthesis by Namba et al.,[^5c^, ^5d^] which used a „west‐to‐east“ approach, stereoselectivity issues arose during the introduction of the 2’‐hydroxygroup by allylic oxidation requiring late stage separation of diastereomers by means of preparative HPLC, thus, limiting access to larger quantities of MA (**VIII**). In earlier work CS and co‐workers synthesized ^13^C_4_‐labelled PS in a similar fashion.^[5e]^ The activity of the synthetic community notwithstanding, to date, useful quantities of all PS in their natural and isotopically (^13^C) labelled form (used as internal standards for trace analysis in soil) are currently not available, making it difficult for groups studying these plant mechanisms to efficiently carry out their research. In this light, no general approach to the more complex mugineic acids (**II**, **III**, **VI** and **VII**) has so far been reported, most likely due to lack of availability of building blocks **4** and **5**. Aiming for more members of this class of compounds and due to the high diversity of „western“ fragments present in naturally occurring PS, the opposite direction of assembly was recognized as a tentatively superior approach which has thus far been widely neglected by the synthetic community.^[5a]^ Within the framework of an interdisciplinary project we set out to establish a general solution for all members of the mugineic and avenic acid family in natural form compatible with an application towards ^13^C_2_‐labelled versions. These are required as standards for high performance trace quantification in complex matrices as well as for biodegradation experiments.

## Results and Discussion

**Figure 2:**
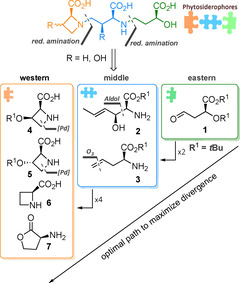
Retrosynthetic rationale: divergent synthesis to access all phytosiderophores, demonstrated with all members of the mugineic and avenic acid family.

As mentioned above our aim was to synthesize eight different PS from „east‐to‐west“ from one common set of building blocks. Thus, the best transformations available to date to arrive at the necessary building blocks **1**–**7** efficiently (see Figure 2) were required. Three of the required building blocks (**3**, **6**, **7**) are commercially available in unprotected form. While there was ample literature on the synthesis of eastern subunit **1**, the chemistry of hydroxylated azetidines like **4** and **5** was still underdeveloped. A palladium catalyzed C−H cyclization^[7]^ was identified as a possible access to these unnatural amino acids. Regarding building block **2** we envisioned introducing the two required stereocenters simultaneously in an aldol‐type 1,2‐addition of a glycine equivalent,^[8]^ which in the future can be prepared from commercially available ^13^C_2_‐labelled glycine. The olefins in **2** and **3** serve as masked aldehydes to be liberated for reductive amination during the course of the synthesis. Notably, this strategy also allows for the preparation of distichonic acids and nicotianamines (Figure 1) by starting from amine protected derivatives of **2** and **3** as eastern fragments, thus unifying the access to all naturally occurring PS through these seven building blocks. Throughout the synthesis acid labile protecting groups (*t*Bu, Boc) were employed to reduce step count and enable a clean deprotection during the endgame.

**Scheme 1:**
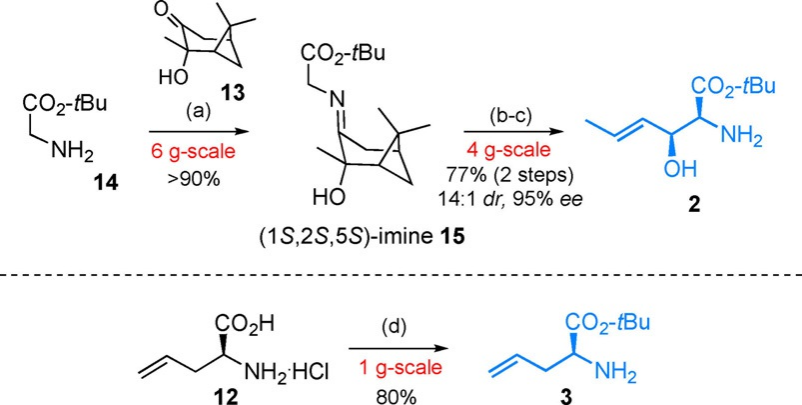
Synthesis of middle building blocks, Reagents and conditions: (a) BF_3_‐OEt_2_, C_6_H_6_, Dean–Stark trap, reflux, 2 h; (b) TiCl(O*i*Pr)_3_, (*E*)‐crotonaldehyde, Et_3_N, CH_2_Cl_2_, 0 °C, 4 h; (c) 15 % citric acid, THF, 4 °C, 40 h; (d) HClO_4_, *t*BuOAc, rt, 24 h.

For the synthesis of middle subunit **2** a highly stereoselective addition of a glycine equivalent to crotonaldehyde was required. The method of Solladié‐Cavallo[^9a^, ^9b^] was conceivably qualified to produce the required *erythro*‐aminoalcohol **2** in high optical purity. Accordingly, the chiral pinanone auxiliary **13** was attached to *tert*‐butyl protected glycine (**14**) under Lewis acid catalysis (See Scheme 1). The resulting imine (**15**) was then added in a titanium mediated aldol reaction to crotonaldehyde, giving rise to a hydroxyimine (not depicted), which due to limited stability (retro‐Aldol) was subsequently hydrolysed under acidic conditions delivering subunit **2** in three steps in a 77 % overall yield. Protected non‐labelled allylglycine **3** was prepared in a straightforward fashion in one step from commercial l‐allylglycine (**12**).

The synthesis of hydroxylated l‐azetidines **4** and **5** was carried out starting from protected l‐ or d‐threonine derivatives **18** and *ent*‐**18** in a three‐step procedure. Initial attachment of a 2‐picolinamide (PA) directing group (DG) was followed by cyclisation under palladium catalysed oxidative C−H activation conditions developed by Chen et al.^[7]^ The *tert*‐butyl protecting groups in **19** and *ent*‐**19** proved to be ideal for the overall success of this route due to their excellent stability, while silyl ethers and other protecting groups were not suitable.

**Scheme 2:**
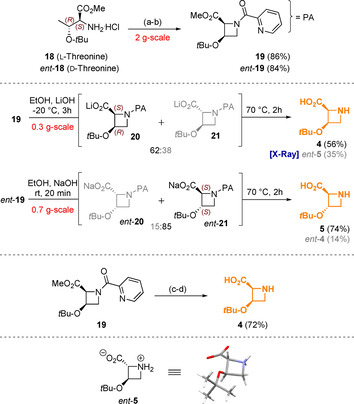
Synthesis of western subunits, l‐azetidines **4** and **5**: Reagents and conditions: (a) 2‐picolinic acid, EDCI⋅HCl, DMAP, DIPEA, HOBt, DCM, rt, 24 h; (b) Pd(OAc)_2_, PhI(OAc)_2_, AcOH, C_6_H_5_Me, 100 °C, 20 h; (c) Me_3_SnOH, DCE, 80 °C, 20 h; (d) NaHCO_3_, EtOH, then NaOH, −15 °C to 70 °C, 3 h.

Next, the methyl ester and DG cleavage was evaluated, at first under basic conditions. When a hydroxide base was employed at room temperature, epimerization to the more stable^[10]^ *trans*‐azetidines (**21** and *ent*‐**21**) proceeded rapidly and with almost full conversion. Subsequently, the amide was cleaved by further heating the reaction mixture. This turned out to be a very efficient solution to access **5**. An influence in temperature and base was found, with epimerization being slower than ester cleavage at lower temperatures. Nevertheless, only a 62:38 mixture of the desired (*cis*‐azetidine **4**) and undesired product (*ent*‐**5**) could be obtained with this method. The assigned stereochemistry was proven by X‐ray diffraction of pure *ent*‐**5** (see Scheme 2, Deposition Number 2007994 contains the supplementary crystallographic data for this paper. These data are provided free of charge by the joint Cambridge Crystallographic Data Centre and Fachinformationszentrum Karlsruhe Access Structures service www.ccdc.cam.ac.uk/structures). While the 56 % isolated yield of **4** in this step are acceptable for such an elusive material, a selective approach was also developed. Thus, the methyl ester in **19** was cleaved first with the mild reagent Me_3_SnOH.^[11]^ No epimerization at C‐2 was observed and the picolinamide could be subsequently cleaved with base after careful neutralization of the carboxylic acid. The amino acids **4** and **5** were separated from any minor isomers present by preparative HPLC and obtained in >60 % overall yield from **18** and *ent*‐**18**.

The eastern subunit aldehyde **1** was prepared in a good yield over 5 steps as reported[^5c^, ^5e^] and assembly of the two central dimeric intermediates could commence.

**Scheme 3:**
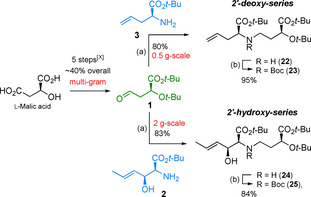
First point of divergence, assembly of building blocks towards key dimeric targets 23 and 25: Reagents and conditions: (a) NaCNBH_3_, AcOH, MgSO_4_, MeOH, 0 °C to rt; (b) Boc_2_O, THF, 40 °C, 16 h.

**Scheme 4:**
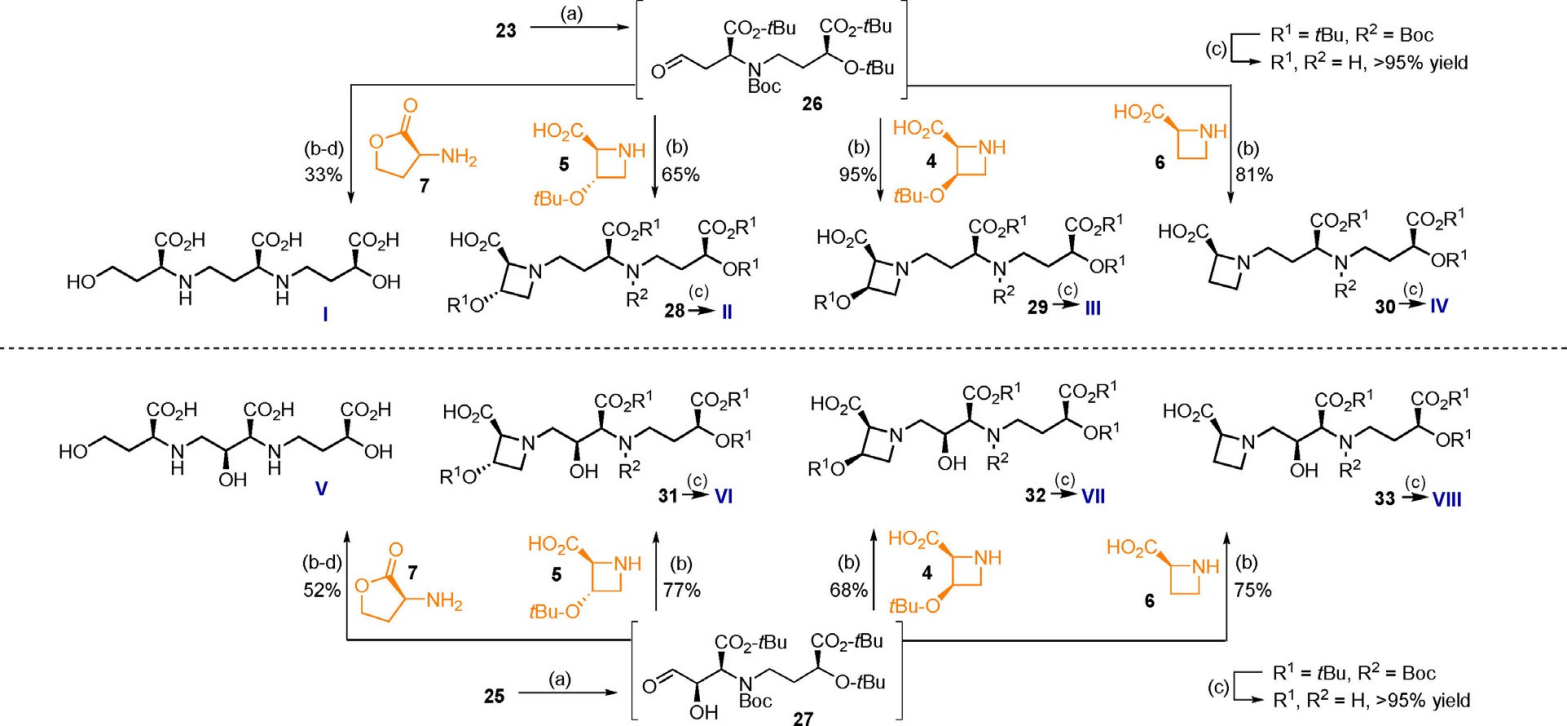
Second point of divergence, assembly and deprotection of target molecules **I**‐**VIII**: Reagents and conditions: (a) O_3_, MeOH, then (Me)_2_S, −78 °C to rt, 18 h, (b) NaCNBH_3_, MeOH, AcOH, MgSO_4_, 0 °C to rt, 1–4 h, (c) 6 m HCl, THF, anisole, rt, 24 h, (d) 2.5 % KOH, rt, 20 h.

Aminoesters **2** and **3** were alkylated under reductive conditions (NaCNBH_3_) with aldehyde **1**. A smooth reaction was achieved in both instances giving 83 % and 80 % of dimeric amines **22** and **24** upon chromatographic purification at decent scale (Scheme 3). Attachment of a Boc protecting group was realized in high yield (Boc_2_O, THF) and the resulting carbamates **23** and **25** proved to be suitably stable storage compounds. The dimeric compounds **23** and **25** now enter the second stage of diversification towards two sets of four phytosiderophores each, with a 2’‐hydroxy and 2’‐deoxy‐structure, respectively. The olefin was cleaved by ozonolysis yielding aldehydes **26** and **27** which were subsequently treated with one of the four respective western building blocks **4**–**7** under reductive amination conditions, as before (Scheme 4). Thanks to the universal protecting group strategy throughout the synthesis, global deprotection of the molecules proved facile and was induced by treatment with 6 m HCl. The resulting amino acids **I**–**VIII** were purified on Dowex® resin and isolated as the respective ammonium salts. For highly pure material additional trituration steps or preparative HPLC can be carried out.^[5e]^ To the best of our knowledge this represents the first total synthesis of 3’’‐*epi*‐hydroxy‐DMA (**II**), 3’’‐hydroxy‐DMA (**III**), hydroxyavenic acid A (**V**) and 3’’‐hydroxy‐MA (**VII**).

A concise and modular synthesis of phytosiderophore natural products was achieved. Starting from l‐malic acid, target compounds **I**–**VIII** could be prepared in 10 or 11 steps longest linear sequence (15–25 % overall yield). The required building blocks could all be efficiently accessed by C−H activation and stereoselective aldol reaction. Common key intermediates (**23** and **25**) allow very flexible and fast resynthesis and delivery of materials for the planned applications within our current project and beyond. As mentioned before, by employing properly protected building blocks **2** and **3** as „eastern“ fragments and compound **14** as „western“ fragment, access to the nicotianaamine and distichonic acid family is well within the scope of the developed methodology.

Further work on fully assembled ^13^C_2_‐labelled versions of compounds **I–VIII** using the presented strategy as well as development towards scale up (gram‐scale) of the avenic acid and mugineic acid syntheses and other PS are currently ongoing in our laboratories and will be reported in due time.

## Conflict of interest

The authors declare no conflict of interest.

## Supporting information

As a service to our authors and readers, this journal provides supporting information supplied by the authors. Such materials are peer reviewed and may be re‐organized for online delivery, but are not copy‐edited or typeset. Technical support issues arising from supporting information (other than missing files) should be addressed to the authors.

Supplementary
