# Supplementary material for: A Unified Approach to Phytosiderophore Natural Products
Source: Chemistry. 2020 Oct 28;27(2):577–80. doi: 10.1002/chem.202004004 (PMC7821100; doi:10.1002/chem.202004004)
Supplement: Supplementary file 1 — Supplementary [file CHEM-27-577-s001.pdf]

Chemistry–A European Journal

Supporting Information

A Unified Approach to Phytosiderophore Natural Products

Nicolas Kratena,[a] Tobias Gökler,[a] Lara Maltrovsky,[a] Eva Oburger,[b] and Christian Stanetty*[a]

Table of Contents

General information 2

Synthesis of eastern Fragment **1** 5

1. (*S*)-2-(2,2-Dimethyl-5-oxo-1,3-dioxolan-4-yl)acetic acid (35) 5
2. Ethyl (*S*)-2-(2,2-dimethyl-5-oxo-1,3-dioxolan-4-yl)acetate (36) 6
3. (*S*)-4-Ethoxy-2-hydroxy-4-oxobutanoic acid (37) 7
4. 1-(*tert*-Butyl) 4-ethyl (*S*)-2-(*tert*-butoxy)succinate (38) 8
5. *tert*-Butyl (*S*)-2-(*tert*-butoxy)-4-oxobutanoate (1) 9

Synthesis of Middle Fragments **2** and **3** 10

6. *tert*-Butyl (*S*)-2-aminopent-4-enoate (3) 10
7. *tert*-Butyl 2-(((1*S*,2*S*,5*S*)-2-hydroxy-2,6,6-trimethylbicyclo[3.1.1]heptan-3-ylidene) amino)acetate (15) 11
8. *tert*-Butyl (2*S*,3*S*,*E*)-2-amino-3-hydroxyhex-4-enoate (2) 12

Synthesis of Western Fragments **4**, **5**, **6** and **7** 14

9. Methyl *O*-(*tert*-butyl)-*N*-picolinoyl-L-threoninate (41) 14
10. Methyl *O*-(*tert*-butyl)-*N*-picolinoyl-D-threoninate (*ent*-41) 15
11. Methyl (2*S*,3*R*)-3-(*tert*-butoxy)-1-picolinoylazetidine-2-carboxylate (19) 16
12. Methyl (2*R*,3*S*)-3-(*tert*-butoxy)-1-picolinoylazetidine-2-carboxylate (*ent*-19) 17
13. (2*S*,3*S*)-3-(*tert*-Butoxy)azetidine-2-carboxylic acid (5) 18
14. (2*S*,3*R*)-3-(*tert*-Butoxy)azetidine-2-carboxylic acid (4) 19
15. (2*R*,3*S*)-3-(*tert*-Butoxy)-1-picolinoylazetidine-2-carboxylic acid (42) and (2*S*,3*R*)-3-(*tert*-butoxy)azetidine-2-carboxylic acid (4) 20
16. (*S*)-3-Aminodihydrofuran-2(3H)-one hydrochloride (7) 21

Synthesis of dimeric compounds **23** and **25** 22

17. *tert*-Butyl (2*S*,3*S*,*E*)-2-(((S)-3,4-di-*tert*-butoxy-4-oxobutyl)amino)-3-hydroxyhex-4-enoate (24) 22
18. *tert*-Butyl (2*S*,3*S*,*E*)-2-((*tert*-butoxycarbonyl)((S)-3,4-di-tert-butoxy-4-oxobutyl) amino)-3-hydroxyhex-4-enoate (25) 23
19. *tert*-Butyl (*S*)-2-(((*S*)-3,4-di-*tert*-butoxy-4-oxobutyl)amino)pent-4-enoate (22) 24
20. *tert*-Butyl (*S*)-2-((*tert*-butoxycarbonyl)((*S*)-3,4-di-*tert*-butoxy-4-oxobutyl)amino) pent-4-enoate (23) 25

Synthesis of trimers **28-33**, **44-45** 26

21. *tert*-Butyl (2*S*,3*R*)-2-((*tert*-butoxycarbonyl)((*S*)-3,4-di-*tert*-butoxy-4-oxobutyl) amino)-3-hydroxy-4-oxobutanoate (27) 26
22. *tert*-Butyl (2*S*)-2-((*tert*-butoxycarbonyl)((*S*)-3,4-di-*tert*-butoxy-4-oxobutyl) amino)-4-oxobutanoate (26) 27
23. (*S*)-1-((2*S*,3*S*)-4-(*tert*-Butoxy)-3-((*tert*-butoxycarbonyl)((*S*)-3,4-di-*tert*-butoxy-4-oxobutyl)amino)-2-hydroxy-4-oxobutyl)azetidine-2-carboxylic acid (33) 28
24. (*S*)-1-((2*S*,3*S*)-4-(*tert*-Butoxy)-3-((*tert*-butoxycarbonyl)((*S*)-3,4-di-*tert*-butoxy-4-oxobutyl)amino)-2-hydroxy-4-oxobutyl)azetidine-2-carboxylic acid (31) 29

25. (*S*)-1-((2*S*,3*R*)-4-(*tert*-Butoxy)-3-((*tert*-butoxycarbonyl)((*S*)-3,4-di-*tert*-butoxy-4-oxobutyl)amino)-2-hydroxy-4-oxobutyl)azetidine-2-carboxylic acid (32) 30

26. *tert*-Butyl (2*S*,3*S*)-2-((*tert*-butoxycarbonyl)((*S*)-3,4-di-*tert*-butoxy-4-oxobutyl) amino)-3-hydroxy-4-(((*S*)-2-oxotetrahydrofuran-3-yl)amino)butanoate (44) 31

27. (*S*)-1-((*S*)-4-(*tert*-Butoxy)-3-((*tert*-butoxycarbonyl)((*S*)-3,4-di-*tert*-butoxy-4-oxobutyl)amino)-4-oxobutyl)azetidine-2-carboxylic acid (30) 32

28. (2*S*,3*S*)-3-(*tert*-Butoxy)-1-((*S*)-4-(*tert*-butoxy)-3-((*tert*-butoxycarbonyl)((*S*)-3,4-di-*tert*-butoxy-4-oxobutyl)amino)-4-oxobutyl)azetidine-2-carboxylic acid (28) 33

29. (2*S*,3*R*)-3-(*tert*-Butoxy)-1-((*S*)-4-(*tert*-butoxy)-3-((*tert*-butoxycarbonyl)((*S*)-3,4-di-*tert*-butoxy-4-oxobutyl)amino)-4-oxobutyl)azetidine-2-carboxylic acid (29) 34

30. *tert*-butyl (*S*)-2-(*tert*-Butoxy)-4-(((*S*)-1-(*tert*-butoxy)-1-oxo-4-(((*S*)-2-oxotetra-hydrofuran-3-yl)amino)butan-2-yl)(*tert*-butoxycarbonyl) amino)butanoate (45) 35

Synthesis of final compounds **I-VIII** 36

31. Mugineic acid (VIII) 36

32. 3’’-*epi*-Hydroxy mugineic acid (VI) 37

33. 3’’-Hydroxy mugineic acid (VII) 38

34. (2*S*,3*S*)-2-(((*S*)-3-Carboxy-3-hydroxypropyl)amino)-3-hydroxy-4-(((*S*)-2-oxotetrahydrofuran-3-yl)amino)butanoic acid (46) 39

35. Hydroxyavenic acid A (V) 40

36. Deoxymugineic acid (IV) 41

37. 3’’-*epi*-Hydroxy-deoxymugineic acid (II) 42

38. 3’’-Hydroxy-deoxymugineic acid (III) 43

39. (*S*)-4-(((*S*)-1-Carboxy-3-(((*S*)-2-oxotetrahydrofuran-3-yl)amino)propyl)amino)-2-hydroxybutanoic acid (47) 44

40. Avenic acid A (I) 45

Spectra 46

References 86

General information

All chemicals were used directly from commercial sources and used without further purification unless specifically stated. Dry triethylamine and DIPEA were distilled over CaH_2

and stored over BaO. $ClTi(O\textit{i}Pr)_3$ (95%, Sigma-Aldrich) was distilled at 0.1 mbar over a short-path condenser. Allyl bromide was freshly distilled and stored over $CaCl_2$. Water-free solvents were available at the institute from a PureSolv EN 1-4 Enclosed solvent drying plant or purchased from Acros Organics. Ion-exchange resins were washed with the respective solvent prior to use. TLC analysis for reaction monitoring and analyzing fraction from column chromatography was performed on silica gel 60 F^{254}-plates. The spots were visualized using UV light (254 nm) followed by staining the plates with anisaldehyde solution (180 mL EtOH, 10 mL anisaldehyde, 10 mL H_2SO_4 conc., 2 mL AcOH), potassium permanganate solution (3.0 g $KMnO_4$, 20.0 g K_2CO_3, 250mg KOH, 300 mL H_2O) or cerium molybdate solution ("Mostain", 21 g $(NH_4)_6Mo_7O_{24}\cdot 4\ H_2O$, 1 g $Ce(SO_4)$, 231 mL conc. H_2SO_4, 500 mL H_2O). NMR spectra were recorded at 298.15 K in the solvent indicated with an Avance UltraShield 400 and an Avance III HD 600 spectrometer. All spectra were calibrated to the solvent residual peak. Chemical shifts (δ) and coupling constants (J) were expressed in ppm and Hz, respectively. In proton NMR code J-couplings refer to $J_{H,H}$ couplings unless specified. Assignments of signals were obtained by careful analysis of extensive 2D-NMR experiments (COSY, HSQC, HMBC). Optical rotation was measured on an Anton Paar MCP 500 at the specified conditions, $[\alpha]^D$ values are given in 10^{-1} deg cm^2g^{-1}. Melting points were recorded on a Kofler-type Leica Galen III or a BÜCHI Melting Point B-545with a 40%/90% threshold. Preparative HPLC was carried out using an Autopurification system from Waters with an ACQUITY QDa detector, combined with a 2998 Photodiode array detector. Separation was conducted with a XSELECT CSH C18 5 µm 4.6 x 150 mm column for analytical samples and a XSELECT CSH Prep C18 5 µm OBD 30 x 150 column for preparative runs. HPLC grade methanol and water, containing 0.1% formic acid were used as eluent system. Ozonolysis was carried out using a Triogen LAB2B air-cooled ozone generator with a pure oxygen flow of ~120 L/h. For single crystal X-ray diffraction analysis, crystals were embedded in perfluorinated polyether and mounted on MITGEN™ loops. X-ray diffraction data were obtained in a cold stream of nitrogen at T = 100 K on a Bruker APEX-II diffractometer with Mo-Kα radiation. The collection strategy for the measurement was optimized with APEX-2 using ω- and φ-scans. After the integration of the data with SAINT, a semi-empirical absorption correction was performed with SADABS. The crystal structures were solved by direct methods and refined using the SHELXTL program package. All H atoms were placed geometrically and refined in a riding model approximation. The N-bound H atoms were located from a difference Fourier map and refined with usual distance restraints. Crystallographic data for the structure reported in this paper have been deposited with the Cambridge Crystallographic Data Centre as

supplementary publication no. CCDC 2007994. The HR-MS analysis was carried out from methanol or acetonitrile or water or a mixture of these solvents (concentration: 10 μM) by using an Agilent G7167B multi sampler, an Agilent G7120A binary pump with degasser, an Agilent G7116B oven and Agilent 6545 Q-TOF mass spectrometer equipped with an dual AJS ion score. Enantiomeric excess was determined *via* normal phase HPLC with a ChiralPak AS-H (250 mm x 4.6 mm ID) or IB (250 mm x 4.6 mm ID) column on a Thermo Scientific/Dionex Ultimate 3000 HPLC using mixtures of *n*-hexane/*n*-heptane and *i*PrOH/EtOH. Solvent mixtures (for chromatography) are given as volume ratios in the format Solvent 1/Solvent 2 = X:Y.

Compounds were named according to IUPAC systematic standards. Numbering of compounds for assignments does not always correspond to IUPAC but was consistently used in this document as shown in the exemplary structures below:

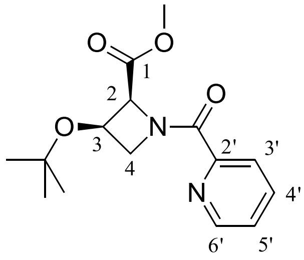

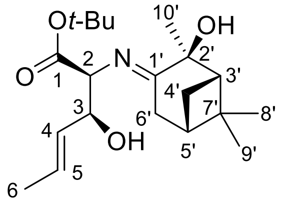

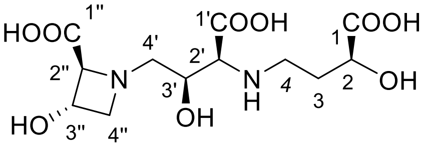

Protecting groups and substituents will be assigned as O*t*Bu, *N*-Boc, COO*t*Bu, **CH$_2$**CH$_3$ etc.

Spectra at the end will be presented in the same order as experimental details on the following pages.

Synthesis of eastern Fragment **1**

1. (*S*)-2-(2,2-Dimethyl-5-oxo-1,3-dioxolan-4-yl)acetic acid (35)

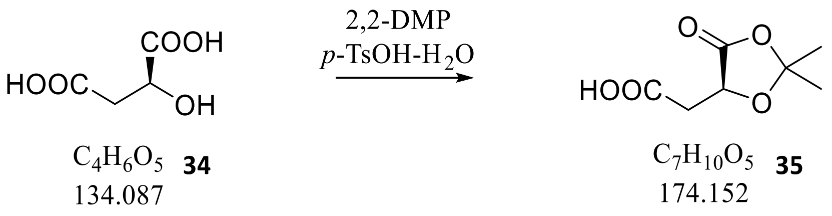

Procedure: L-Malic acid **34** (10.0 g, 74.6 mmol, 1 eq.) was weighed into a flame dried 250-mL round bottom flask, *p*-toluenesulfonic acid monohydrate (141 mg, 0.75 mmol, 0.01 eq) was added to the solution and the mixture dissolved in 2,2-dimethoxypropane (70 mL, 0.58 mol, 7.7 eq.) at room temperature. The reaction was then stirred at room temperature for 5.5 h.

Workup: Sodium bicarbonate (84.0 mg, 0.01 eq.) was dissolved in H_2O (40 mL) and added to the reaction mixture over the course of 1 minute. Immediately afterwards CH_2Cl_2 (70 mL) was added and the mixture was extracted twice with CH_2Cl_2 (170 mL total). The solution was dried over $MgSO_4$ and evaporated to dryness to give 12.9 g of crude material.

Purification: The crude product was dissolved in dry Et_2O/CH_2Cl_2 = 1:1 (50 mL) with mild heating (40 °C) and then diluted with *n*-hexane (150 mL). A solid formed and the solution was concentrated to half of its volume and the crystals collected by filtration to yield 11.5 g of **35** as a fine microcrystalline material (89% yield, 66 mmol).

m.p.: 102 – 104 °C (hexane), Lit: 107 – 108 °C [1], $[\alpha]_{20}^{D}$ = +5.3 (c 1.02, MeOH), Lit: +5.9 (c 0.9, $CHCl_3$) [1]. ^{1}H NMR (600 MHz, $CDCl_3$) δ 4.72 (dd, *J* = 6.6, 3.8 Hz, 1H, H-2), 3.00 (dd, *J* = 17.3, 3.8 Hz, 1H, H-3a), 2.86 (dd, *J* = 17.3, 6.6 Hz, 1H, H-3b), 1.63 (d, *J* = 0.8 Hz, 3H, C**CH_3**-a), 1.58 (d, *J* = 0.7 Hz 3H, C**CH_3**-b). ^{13}C NMR (50 MHz, $CDCl_3$) δ 174.8, 172.0, 111.5, 70.5, 36.1, 26.9, 26.0. HR-MS (ESI): *m/z:* calc. for $NaC_7H_{10}O_5$ $[M+Na]^+$: 197.0420, found: 197.0422.

2. Ethyl (*S*)-2-(2,2-dimethyl-5-oxo-1,3-dioxolan-4-yl)acetate (36)

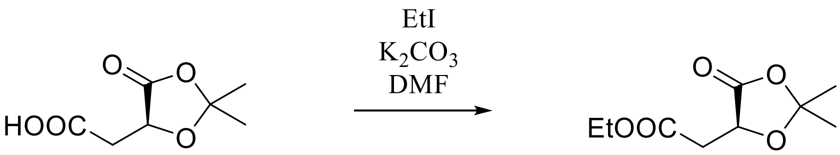

$C_7H_{10}O_5$ **35**
174.152

$C_9H_{14}O_5$ **36**
202.206

Procedure: The carboxylic acid **35** (12.0 g, 69 mmol, 1 eq.) was weighed in a flame-dried Schlenk flask, followed by the addition of potassium carbonate (18.7 g, 135 mmol, 2 eq.), as a solution in dry DMF (45 mL). Then, the suspension was stirred at room temperature for 10 min and ethyl iodide (8.3 mL, 0.1 mol, 1.5 eq.) was introduced to the reaction vessel. Henceforward, the heterogenous mixture was stirred vigorously at room temperature und argon. After 3h TLC analysis showed complete consumption of the starting material.

Workup: A mixture of saturated NH_4Cl solution and H_2O (1/1, 40 mL) was added, followed by introducing small amounts of Et_2O (in total 40 mL). The resulting biphasic mixture was extracted with Et_2O (3×30 mL). The combined, organic fractions were washed with minimal amount of brine (10 mL), dried over $MgSO_4$ and evaporated to dryness, to give 11.1 g of crude material.

Purification: Flash chromatography was carried out on 22 g silica gel, using a LP/EtOAc mixture (9:1) as eluent. Excluding the residual amount of DMF (quantified by ^{1}H-NMR) the obtained mass of product **36** was 7.7 g (56%, 38 mmol).

R_f = 0.73 (LP/EtOAc, 3:2). $[\alpha]_{20}^{D}$ = +12.5 (c 1.1, MeOH), Lit: $[\alpha]_D^{20}$= +3.7 (c 1.5, $CHCl_3$) [2]. ^{1}H NMR (600 MHz, $CDCl_3$) δ 4.70 (dd, *J* = 6.5, 3.8 Hz, 1H, H-2), 4.18 (qd, *J* = 7.1, 2.0 Hz, 2H, **CH_2**CH_3), 2.90 (dd, *J* = 17.0, 3.9 Hz, 1H, H-3a), 2.77 (dd, *J* = 17.0, 6.5 Hz, 1H, H-3b), 1.61 (s, 3H, C**CH_3**-a), 1.55 (s, 3H, C**CH_3**-b), 1.26 (t, *J* = 7.1 Hz, 3H, CH_2**CH_3**). ^{13}C NMR (151 MHz, $CDCl_3$) δ 172.2, 169.3, 111.2, 70.8, 61.3, 36.4, 26.9, 26.0, 14.2. HR-MS (ESI): *m/z:* calc. for $NaC_9H_{14}O_5$ $[M+Na]^+$: 225.0732, found: 225.0736.

3. (*S*)-4-Ethoxy-2-hydroxy-4-oxobutanoic acid (37)

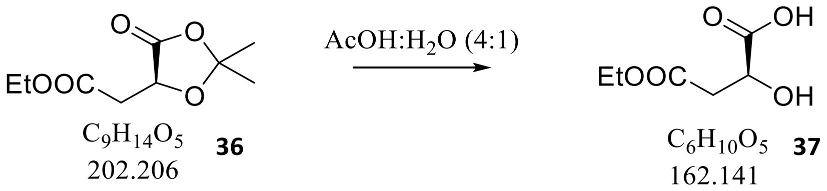

Procedure: The ester **36** (4.6 g, 23 mmol, 1 eq.) was transferred to a 25-mL round bottom flask. The colorless oil was dissolved in a mixture of AcOH/H_2O (4:1, 12 mL) and stirred at 100 °C for 1.5 h. The TLC analysis showed complete consumption of starting material.

Workup/Purification: The reaction mixture was evaporated to dryness. Small amounts of toluene (in total 30 mL) were transferred to the resulting yellowish oil and subsequently co-evaporated. This process was repeated 4 times, before the obtained colorless oil was dried under high vacuum until constant weight, yielding 3.76 g (97%, 22.5 mmol) of product **37** as a thick oil.

$[\alpha]_D^{20}$ = -6.8 (c 1.0, MeOH), Lit: $[\alpha]_D^{20}$= -6.95 (c 1.6, MeOH) [2]. ^{1}H NMR (600 MHz, $CDCl_3$) δ 4.56 (dd, *J* = 6.3, 4.4 Hz, 1H, H-2), 4.23 – 4.16 (m, 2H, **CH$_2$**CH_3), 2.92 (dd, *J* = 16.8, 4.4 Hz, 1H, H-3a), 2.85 (dd, *J* = 16.8, 6.2 Hz, 1H, H-3b), 1.27 (t, *J* = 7.1 Hz, 3H, CH_2**CH$_3$**). ^{13}C NMR (151 MHz, $CDCl_3$) δ 177.0, 171.4, 67.1, 61.6, 38.4, 14.2. HR-MS (ESI): *m/z:* calc. for $C_6H_{10}O_5$ $[M\text{-H}]^-$: 163.0601, found: 163.0610.

4. 1-(*tert*-Butyl) 4-ethyl (*S*)-2-(*tert*-butoxy)succinate (38)

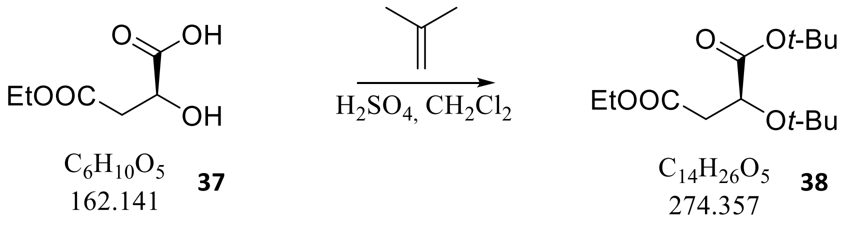

Procedure: The hydroxyacid **37** (1.58 g, 9.74 mmol, 1 eq.) was dissolved in dry CH_2Cl_2 (10 mL) and the resulting solution was transferred to a flame-dried high-pressure vessel. The reaction mixture was chilled to circa -30 °C by an acetone/liq. N_2 bath and bubbled isobutylene through the solution for 2 min, until the volume of condensed isobutylene was approximately the same as the volume of the solvent. The removal of the cooling bath was followed by the addition of conc. sulfuric acid (90 µL, 1.8 mmol, 0.18 eq.). The vessel was sealed and stirred at room temperature for 4h.

Workup: The solution was quenched by the addition of saturated $NaHCO_3$ solution (20 mL) and extracted with EtOAc (3× in total 100 mL). The combined organic phases were washed with $NaHCO_3$ solution (10 mL), followed by the washing with a small amount of brine (5 mL) and drying over $MgSO_4$. Then the organic phases were evaporated to dryness, giving 2.8 g of crude material.

Purification: The crude material was purified by column chromatography on 30 g silica gel, using a mixture of LP/EtOAc = 10:1 as eluent, providing 2.21 g (83%, 8 mmol) of product **38** as thick oil as well as 0.42 g of the mono *tert*-butylated byproduct.

R_f = 0.63 (LP/EtOAc = 5:1). $[\alpha]_D^{20}$ = -37.9 (c 0.8, MeOH), Lit: $[\alpha]_D^{21}$= -37.3 (c 1.5, $CHCl_3$) [3]. ^{1}H NMR (600 MHz, $CDCl_3$) δ 4.32 (dd, *J* = 8.3, 5.1 Hz, 1H, H-2), 4.20 – 4.08 (m, 2H, $\mathbf{CH_2}CH_3$), 2.64 (dd, *J* = 15.2, 5.1 Hz, 1H, H-3a), 2.58 (dd, *J* = 15.2, 8.3 Hz, 1H, H-3b), 1.45 (s, 9H, COO*t*Bu), 1.26 (t, *J* = 7.1 Hz, 3H, $CH_2\mathbf{CH_3}$), 1.19 (s, 9H, O*t*Bu). ^{13}C NMR (151 MHz, $CDCl_3$) δ 172.5, 170.6, 81.4, 75.4, 69.2, 60.8, 39.6, 28.0 (3C), 27.9 (3C), 14.3. HR-MS (ESI): *m/z:* calc. for $C_{14}H_{27}O_5$ $[M+H]^+$: 275.1853, found: 275.1870.

5. ***tert*-Butyl (*S*)-2-(*tert*-butoxy)-4-oxobutanoate (1)**

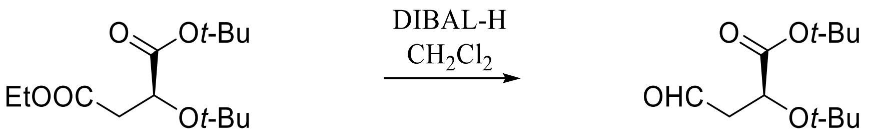

$C_{14}H_{26}O_5$ 274.357 **38** $C_{12}H_{22}O_4$ 230.304 **1**

Procedure: Diester **38** (1.5 g, 5.5 mmol, 1 eq.) was transferred to a flame-dried Schlenk flask and dissolved in CH_2Cl_2 (30 mL) under a steady stream of argon. Then the mixture was chilled to -70 °C with an acetone/liquid nitrogen bath, followed by the dropwise addition of 1M diisobutylaluminium hydride (5.2 g, 7.4 mL, 7.4 mmol, 1.3 eq.) in heptane. The resulting solution was stirred 3.5 h at this temperature. Complete consumption of starting material was checked with TLC (LP/EtOAc = 6:1) analysis.

Workup: After the removal of the cooling bath, the reaction was quenched by the addition of MeOH (3.5 mL). Then, the reaction mixture was transferred to an Erlenmeyer flask, filled with a mixture of CH_2Cl_2 and saturated Na-K-tartrate solution (80 mL, 1:1), and stirred for 20 min. The resulting biphasic mixture was extracted with CH_2Cl_2 (3×, total 200 mL). The pooled organic phases were washed with a minimal amount of brine, dried over $MgSO_4$ and evaporated to dryness, providing 1.2 g of crude material.

Purification: The crude product was purified *via* flash column chromatography on 30 g silica gel, using a mixture of LP/EtOAc = 10:1 as eluent, yielding 1.06 g (84%, 4.6 mmol) of aldehyde **1** as a colorless oil which solidifies when stored below 0 °C.

R_f = 0.53 (LP/EtOAc, 2:1). $[\alpha]_D^{20}$ = -6.3 (c 1.02, MeOH), ^{1}H NMR (600 MHz, $CDCl_3$) δ: 9.75 (t, *J* = 1.9 Hz, 1H, H-4), 4.38 (dd, *J* = 7.9, 4.7 Hz, 1H, H-2), 2.73 (ddd, *J* = 16.4, 7.9, 2.1 Hz, 1H, H-3a), 2.63 (ddd, *J* = 16.4, 4.7, 1.8 Hz, 1H, H-3b), 1.46 (s, 9H, COO*t*Bu), 1.20 (s, 9H, O*t*Bu). ^{13}C NMR (151 MHz, $CDCl_3$) δ: 199.7, 172.3, 81.6, 75.6, 67.5, 47.3, 27.9 (6C). HR-MS (ESI): *m/z:* calc. for $NaC_{12}H_{22}O_4$ $[M+Na]^+$: 253.1410, found: 253.1422.

Synthesis of Middle Fragments **2** and **3**

6. ***tert*-Butyl (*S*)-2-aminopent-4-enoate (3)**

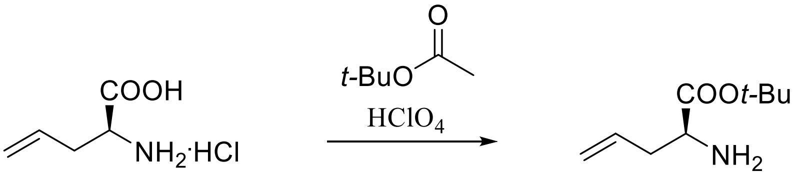

$C_5H_{10}ClNO_2$ **12**
151.590

$C_9H_{17}NO_2$ **3**
171.240

Procedure: The amino acid hydrochloride **12** (0.2 g, 1.3 mmol, 1 eq.) was charged in a flame-dried round bottom flask, followed by the addition of *tert*-butyl acetate (10.4 mL, 76.0 mmol, 60 eq.). After the dropwise addition of 70% perchloric acid (166 mg, 1.70 mmol, 1.3 eq.) the reaction was stirred at room temperature for 24 h under an argon atmosphere. The opaque precipitation of strating material dissolved over the course of 3 h and the reaction mixture was homogenous upon workup.

Workup: The mixture was poured onto $NaHCO_3$ solution (10 mL) and H_2O (5 mL) and the flask washed with EtOAc (circa 5 mL). The mixture was further treated with Na_2CO_3 until evolution of gas subsided and pH was ~9. The phases were separated and the aq. phase extracted with EtOAc (3×, total 50 mL). The combined organic phases were washed with brine (2 mL) dried over $MgSO_4$ and evaporated to dryness to give 194 mg of amine **3** (89%, 1.13 mmol) as a colorless oil. No further purification was necessary.

R_f = 0.44 (CH_2Cl_2/MeOH, 20:1). $[\alpha]_D^{20}$ = -0.8 (c 1.0, $CHCl_3$), Lit: -0.91 (c 0.98, $CHCl_3$) [4]. ^1H NMR (600 MHz, $CDCl_3$) δ 5.77 – 5.64 (m, 1H, H-4), 5.20 – 5.16 (m, 1H, H-5a), 5.16 – 5.14 (m, 1H, H-5b), 3.60 (dd, *J* = 6.5, 5.3 Hz, 1H, H-2), 2.55 – 2.48 (m, 1H, H-3b), 2.46 (d, *J* = 7.0 Hz, 1H, H-3a), 1.45 (s, 9H, COO*t*Bu). ^{13}C NMR (151 MHz, $CDCl_3$) δ 172.4, 132.1, 119.5, 82.1, 53.4, 37.7, 27.9 (3C). HR-MS (ESI): *m/z:* calc. for $C_9H_{18}NO_2$ $[M+H]^+$: 172.1332, found: 172.1333.

7. ***tert*-Butyl 2-(((1*S*,2*S*,5*S*)-2-hydroxy-2,6,6-trimethylbicyclo[3.1.1]heptan-3-ylidene) amino)acetate (15)**

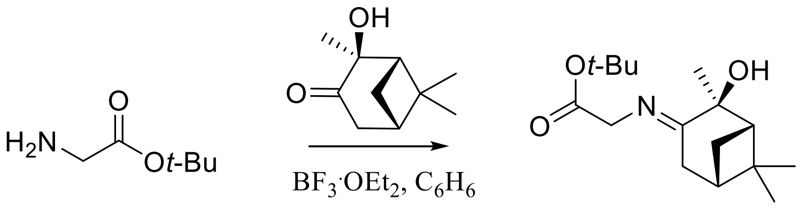

$C_6H_{13}NO_2$ **14**
131.175

$C_{16}H_{27}NO_3$ **15**
281.396

Procedure: A Dean-Stark apparatus was installed and filled with dry benzene. In a 250-mL round bottom flask (*S*,*S*,*S*)-pinanone (4.11 g, 24.4 mmol, 1.00 eq.) was charged followed by 150 mL of benzene. Then *tert*-butyl glycine **14** (4.50 g, 34.2 mmol, 1.40 eq.) was added to the solution *via* syringe. The solution was heated to reflux before adding boron trifluoride etherate (0.21 mL, 1.71 mmol, 0.07 eq.) *via* syringe to the reaction mixture and continuing the reflux. After 2 h TLC analysis indicated complete conversion of pinanone.

Workup: The yellow solution was concentrated under reduced pressure and exclusion of air (40 °C water bath, ca. 20 mL) and was then directly applied to a chromatography column.

Purification: The solution of crude product was purified on 40 g silica gel *via* flash chromatography using LP/EtOAc = 2:1 containing 0.5% triethylamine as eluent. The pooled fractions provided 6.79 g of product **15** (ca. 80% purity by ^{1}H-NMR, impurity: pinanone, 77% yield, 18.8 mmol) as well as 0.68 g of recovered **14**.

The product obtained after the flash chromatography can be employed in the next transformation; an analytically pure sample was obtained by performing a second chromatography (LP/EtOAc = 6:1) on 100 mg of product.

R_f = 0.28 (LP/EtOAc, 3:1). $[\alpha]_D^{20}$ = -6.1 (c = 0.87, CH_2Cl_2), Lit: -10.0 (c 1.47, C_6H_6) [5], ^{1}H NMR (600 MHz, $CDCl_3$) δ 4.08 (m, 2H, H-2), 2.61 (s, 1H, OH), 2.53 – 2.41 (m, 2H, H-6'), 2.34 (m, 1H, H-4'a), 2.07 (t, *J* = 5.9 Hz, 1H, H-3'), 2.05 – 2.01 (m, 1H, H-5'), 1.56 (d, *J* = 10.7 Hz, 1H, H-4'b), 1.52 (s, 3H, H-10'), 1.48 (s, 9H, *t*-Bu), 1.32 (s, 3H, H-9'), 0.87 (s, 3H, H-8'). ^{13}C NMR (151 MHz, $CDCl_3$) δ 179.8, 169.5, 81.5, 76.6, 53.6, 50.4, 38.7, 38.4, 33.8, 28.4, 28.3 (3C), 28.2, 27.5, 23.0. HR-MS (ESI): Calc. *m/z* 304.1883 $[M+Na]^+$, found: 304.1889.

8. *tert*-Butyl (2*S*,3*S*,*E*)-2-amino-3-hydroxyhex-4-enoate (2)

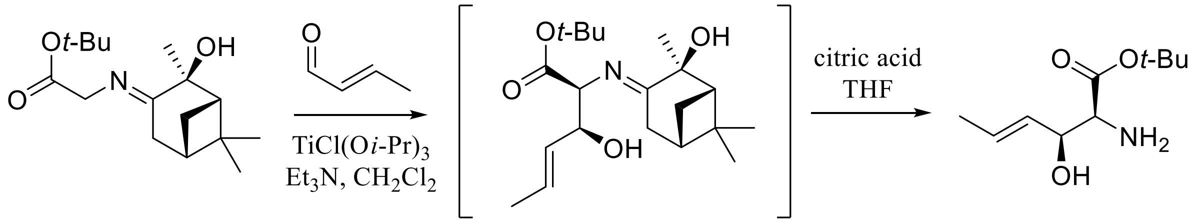

$C_{16}H_{27}NO_3$ **15**
281.396

$C_{20}H_{33}NO_4$ **16**
351.487

$C_{10}H_{19}NO_3$ **2**
201.266

1st step: **Procedure:** In a flame-dried Schlenk flask imine **15** (4.07 g, 80% purity, 11.0 mmol, 1.00 eq.) was charged in dry CH_2Cl_2 (70 mL). The solution was chilled to 0 °C in an ice-bath. Ti(O*i*-Pr)$_3$Cl (4.15 g, 15.9 mmol, 1.45 eq.) was dissolved in dry CH_2Cl_2 (3 mL) and added dropwise to the chilled solution. Then *trans*-crotonaldehyde (1.18 mL, 14.3 mmol, 1.30 eq.), was added followed by anhydrous Et_3N (3.81 mL, 27.5 mmol, 2.50 eq.) in a dropwise fashion. A yellow solid formed over the course of the reaction. The mixture was stirred at 0 °C for 5 h or until TLC (LP/EtOAc = 1:1) showed complete conversion. **Workup:** The reactiion was quenched by addition of brine (60 mL) and extracted with EtOAc (3× 125 mL) and Et_2O (2× 70 mL). The combined organic phases were washed with 20 mL of brine, dried over $MgSO_4$ and evaporated to give 4.91 g of a yellow oil (**16**) that was used immediately in the next step.

Procedure: In a 250-mL round bottom flask the crude material from the last step was dissolved in 100 mL THF. Citric acid (15%, 140 mL) was added at 0 °C and the mixture was placed to stand in the fridge at 4 °C over 70 h. **Workup:** The mixture was first extracted Et_2O (3× with 50 mL). The aqueous phase was neutralized by addition of Na_2CO_3 (until the evolution of gas subsided) and then basified by addition of 2N NaOH to reach pH > 10. The aqueous phase was extracted with EtOAc (3× 100 mL) and Et_2O (4× 50 mL). The pooled extracts were washed with 10 mL brine, were dried over $MgSO_4$ and concentrated under vacuum to give 2.53 g. From the first extract (*S*,*S*,*S*)-pinanone could be recovered by flash chromatography (ca. 70% recovery). **Purification:** The crude product was further purified on 100 g silica gel using CH_2Cl_2/MeOH = 98:2 containing 0.1 % Et_3N as eluent to provide after evaporation of the product fractions 1.70 g (77%, 8.46 mmol) of amine **2**.

R_f = 0.55 (CH_2Cl_2/MeOH, 10:1). $[\alpha]_D^{20}$ = -7.1 (c 1.07, $CHCl_3$). ^{1}H NMR (600 MHz, CD_2Cl_2): δ 5.72 (dqd, *J* = 15.3, 6.5, 1.2 Hz, 1H, H-5), 5.37 (ddq, *J* = 15.2, 6.7, 1.7 Hz, 1H, H-4), 4.23 (dd, *J* = 6.9, 4.9, 1H, H-3), 3.45 (d, *J* = 4.9 Hz, 1H, H-2), 2.02 (br s, 2H, NH_2), 1.68 (ddd, *J* = 6.5, 1.7, 0.9 Hz, 3H, H-6), 1.43 (s, 9H, COO*t*Bu). ^{13}C NMR (151 MHz, CD_2Cl_2): δ 173.0, 129.3,

129.1, 81.9, 73.1, 59.7, 28.3 (3C), 18.0. HR-MS (ESI): Calc. *m/z* 352.2483 [*M*+H]$^+$, found: 352.2489.

The hydrochloride of **2** was formed by dissolving 110 mg of **2** in 5 mL dry Et_2O and adding 2M HCl in Et_2O (0.3 mL, 0.6 mmol, 1.1 eq.) dropwise at 0 °C. The mixture was stirred in an ice-bath for 20 min before filtrating the solids to give 125 mg of **2**·HCl (96%). Analysis of the pure hydrochlorides in proton NMR revealed a diastereomeric ratio of 14:1.

Amine **2** (80 mg) was also protected with Cbz ($NaHCO_3$, THF, CbzCl) to give 82 mg (62%) of the protected compound **39**. A racemic sample of Cbz-protected amine **39** was obtained by a 2-step procedure from **14**·HCl. First Cbz protection (CbzCl, pyridine, CH_2Cl_2 [6]), gave Cbz protected *t*-butyl glycine **40** which reacted in a chelation mediated aldol reaction (LDA, then ClTi(O*i*Pr)$_3$, then crotonaldehyde, THF, -78 °C, [7]) to give **39** as an approximately 1:6 mixture of *syn*:*anti* diastereomers. The materials were analyzed on chiral HPLC and revealed a ratio of enantiomers of ca. 93(*S*,*S*):2.5(*R*,*S*+*S*,*R*):4(*R*,*R*) at retention times 32.6, 28.2 and 24.9 min (See chromatograms below). This corresponds to an enantiomeric excess of ca. 95% regarding the desired pair of diastereomers.

Figure 1: Chiral HPLC trace of racemic **39**

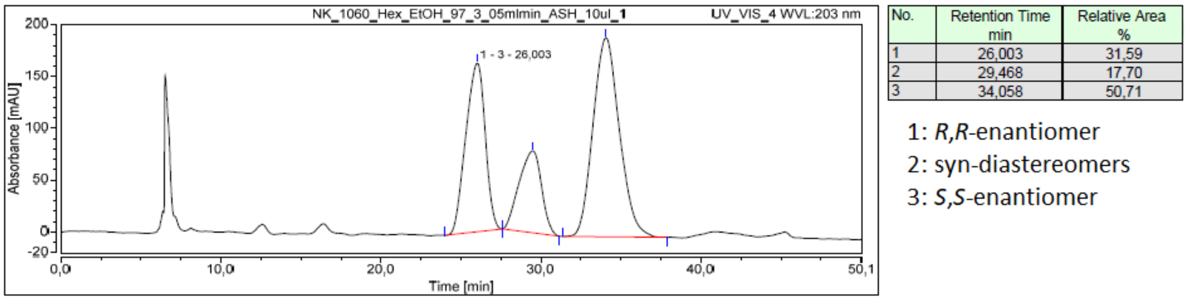

No.	Retention Time min	Relative Area %
1	26,003	31,59
2	29,468	17,70
3	34,058	50,71

1: *R*,*R*-enantiomer
2: syn-diastereomers
3: *S*,*S*-enantiomer

Figure 2: Chiral HPLC trace of **39** derived from pinanone Aldol

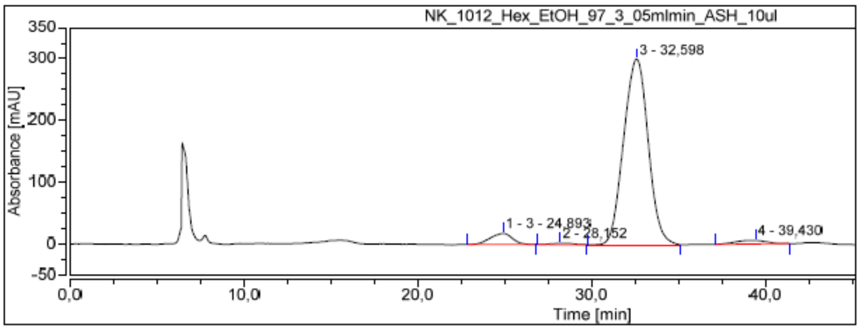

No.	Retention Time min	Relative Area %
1	24,893	4,88
2	28,152	0,59
3	32,598	92,27

1: *R*,*R*-enantiomer
2: syn-diastereomers
3: *S*,*S*-enantiomer

Synthesis of Western Fragments **4**, **5**, **6** and **7**

9. Methyl *O*-(*tert*-butyl)-*N*-picolinoyl-L-threoninate (41)

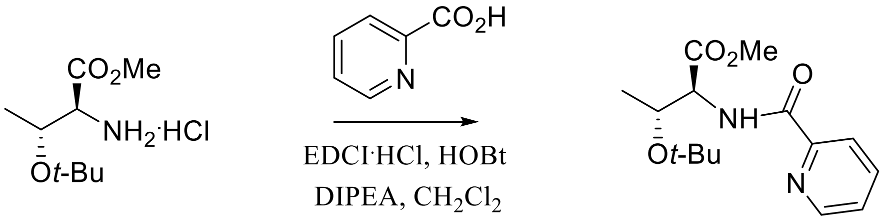

$C_9H_{20}ClNO_3$ 225.713 **18**

$C_{15}H_{22}N_2O_4$ 294.351 **41**

Procedure: In a 50-mL round-bottom flask protected L-threonine hydrochloride **18** (1.00 g, 4.43 mmol, 1.00 eq.) was charged and dry CH_2Cl_2 (22 mL) was added. Diisopropylethylamine (2.30 mL, 13.3 mmol, 3.00 eq.) was added dropwise, followed by 2-picolinic acid (654 mg, 5.32 mmol, 1.20 eq.), HOBt hydrate (814 mg, 5.32 mmol, 1.20 eq.) and EDCI hydrochloride (1.02 g, 5.32 mmol, 1.20 eq.). The clear yellow solution was stirred for 16 h at room temperature.

Workup: The reaction was quenched by addition of H_2O (10 mL). The resulting mixture was extracted with CH_2Cl_2 (3× 20 mL), washed with brine (10 mL), dried over $MgSO_4$, filtrated and evaporated to give 2.1 g of crude product.

Purification: The crude material was purified *via* flash chromatography (20 g silica gel, LP/EtOAc = 8:1 → 5:1) to give product fractions which were pooled and solvents stripped to provide 1.28 g of pure product **41** (98%, 4.35 mmol).

R_f = 0.27 (LP/EtOAc, 2:1). m.p.: 127 – 128 °C (LP/EtOAc). $[\alpha]_D^{20}$ = +69.9 (c 0.77, CH_2Cl_2). ^{1}H NMR (400 MHz, $CDCl_3$) δ 8.68 (d, *J* = 9.3 Hz, 1H, NH), 8.62 (ddd, *J* = 4.8, 1.7, 0.9 Hz, 1H, H-6'), 8.17 (m, 1H, H-3'), 7.83 (m, 1H, H-4'), 7.43 (ddd, *J* = 7.6, 4.8, 1.3 Hz, 1H, H-5'), 4.71 (dd, *J* = 9.4, 2.3 Hz, 1H, H-2), 4.33 (qd, *J* = 6.3, 2.3 Hz, 1H, H-3), 3.72 (s, 3H, COOMe), 1.23 (d, *J* = 6.3 Hz, 3H, H-4), 1.18 (s, 9H, O*t*Bu). ^{13}C NMR (101 MHz, $CDCl_3$) δ 171.4, 164.9, 149.7, 148.5, 137.3, 126.4, 122.5, 74.3, 67.8, 58.3, 52.4, 28.5 (3C), 21.0. HR-MS (ESI): *m/z:* calc. for $C_{15}H_{23}N_2O_4$ $[M+H]^+$: 295.1653, found: 295.1656.

10. Methyl *O*-(*tert*-butyl)-*N*-picolinoyl-D-threoninate (*ent*-41)

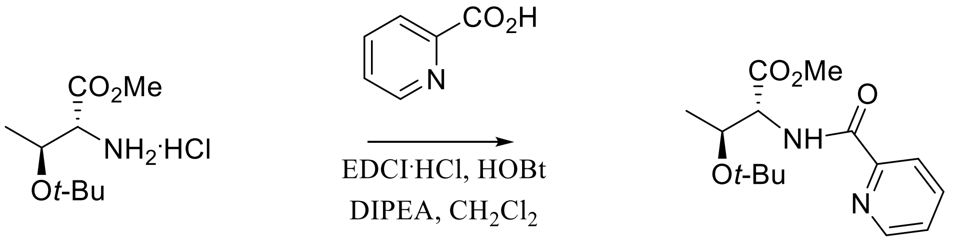

$C_9H_{20}ClNO_3$ 225.713 *ent*-**18**

$C_{15}H_{22}N_2O_4$ 294.351 *ent*-**41**

Procedure: A 50-mL round-bottom flask was charged with protected D-threonine hydrochloride *ent*-**18** (1.00 g, 4.43 mmol, 1 eq.) and dissolved in dry CH_2Cl_2 (20 mL). Diisopropylethylamine (2.30 mL, 13.3 mmol, 3 eq.) was added dropwise, followed by 2-picolinic acid (600 mg, 4.87 mmol, 1.1 eq.), HOBt hydrate (746 mg, 4.87 mmol, 1.1 eq.) and EDCI hydrochloride (930 mg, 4.87 mmol, 1.1 eq.). The clear yellow solution was stirred for 5 h at room temperature.

Workup: The reaction was quenched by addition of H_2O (10 mL). The resulting mixture was extracted with CH_2Cl_2 (3× 20 mL), washed with brine (10 mL), dried over $MgSO_4$, filtrated and evaporated to give 2 g of crude product.

Purification: The crude material was purified *via* flash chromatography (20 g silica gel, LP/EtOAc = 7:1) to give product fractions which were pooled and solvents stripped to provide 1.26 g of pure product *ent*-**41** (97%, 4.28 mmol).

R_f = 0.27 (LP/EtOAc, 2:1). $[\alpha]_D^{20}$ = -70.2 (c 0.83, CH_2Cl_2), ^{1}H NMR (600 MHz, $CDCl_3$) δ 8.67 (d, *J* = 9.3 Hz, 1H, NH), 8.62 (ddd, *J* = 4.8, 1.7, 0.9 Hz, 1H, H-6'), 8.17 (d, *J* = 7.8 Hz, 1H, H-3'), 7.83 (td, *J* = 7.7, 1.7 Hz, 1H, H-4'), 7.42 (ddd, *J* = 7.6, 4.8, 1.3 Hz, 1H, H-5'), 4.71 (dd, *J* = 9.3, 2.2 Hz, 1H, H-2), 4.33 (qd, *J* = 6.3, 2.2 Hz, 1H, H-3), 3.73 (s, 3H, COOMe), 1.23 (d, *J* = 6.3 Hz, 3H, H-4), 1.17 (s, 9H, O*t*Bu).^{13}C NMR (151 MHz, $CDCl_3$) δ 171.4, 165.0, 149.7, 148.5, 137.3, 126.4, 122.4, 77.2, 74.3, 67.8, 58.2, 52.4, 28.5 (3C), 21.1. HR-MS (ESI): *m/z:* calc. for $NaC_{15}H_{22}N_2O_4$ $[M+Na]^+$: 317.1472, found: 317.1481.

11. Methyl (2*S*,3*R*)-3-(*tert*-butoxy)-1-picolinoylazetidine-2-carboxylate (19)

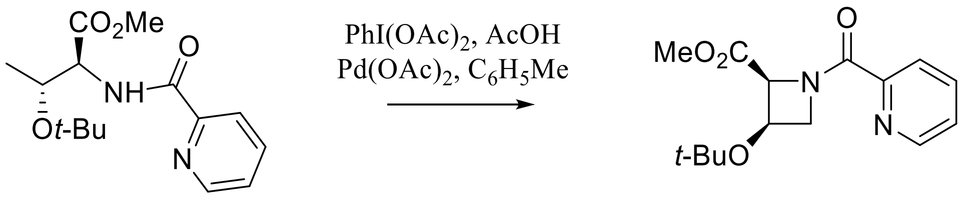

$C_{15}H_{22}N_2O_4$ 294.351 **41** → $C_{15}H_{20}N_2O_4$ 292.335 **19**

Procedure: In an argon flushed 250-mL round-bottom flask amide **41** (2.54 g, 8.63 mmol, 1 eq.) was weighed in followed by dry toluene (80 mL). PIDA (6.95 g, 21.6 mmol, 2.5 eq.), palladium acetate (97 mg, 0.40 mmol, 5 mol%) and acetic acid (1.000 mL, 17.27 mmol, 2 eq.) were added to the solution at room temperature. The reaction was heated to 105 °C in an oil bath in a closed vessel and stirred at that temperature for 18 h.

Workup/Purification: After the indicated period of time the reaction was allowed to cool to rt and TLC showed complete conversion. The solution was concentrated to 1/8th volume and applied to a 60 g silica gel column using LP/EtOAc = 3:1 as eluent giving 2.25 g of azetidine **19** as light-yellow crystals (89%, 7.70 mmol).

R_f = 0.15 (LP/EtOAc, 2:1), m.p.: 86 – 87 °C (EtOAc), $[\alpha]_D^{20}$ = -67.4 (c 1.66, CH_2Cl_2), Mixture, ca. 3:1 of rotamers, minor rotamer („-1") set as 1H in integrals, in accordance to literature [9]: ^{1}H NMR (400 MHz, $CDCl_3$) δ 8.57 (ddd, *J* = 4.8, 1.8, 0.9 Hz, 1H, H-6'-1), 8.52 – 8.39 (m, 3H, H-6'-2), 8.14 (dt, *J* = 7.9, 1.1 Hz, 3H, H-5'), 7.78 (td, *J* = 7.7, 1.8 Hz, 3H, H-4'), 7.36 (ddd, *J* = 7.6, 4.8, 1.3 Hz, 1H, H-3'-1), 7.32 (ddd, *J* = 7.6, 4.8, 1.3 Hz, 3H, H-3'-2), 5.64 (dd, *J* = 7.5, 1.4 Hz, 3H, H-2-1), 5.09 (dd, *J* = 7.6, 1.3 Hz, 1H, H-2-2), 5.03 – 4.93 (m, 1H, H-3-2), 4.73 (td, *J* = 7.3, 5.8 Hz, 4H, H-4a), 4.64 (ddd, *J* = 10.1, 5.7, 1.3 Hz, 1H, H-4b-2), 4.40 (dd, *J* = 10.1, 7.1 Hz, 3H, H-3-1), 4.20 (ddd, *J* = 10.1, 5.8, 1.5 Hz, 3H, H-4b-1), 3.80 (s, 3H, H-11-2), 3.66 (s, 9H, H-11-1), 1.18 (s, 27H, O*t*Bu-1), 1.18 (s, 9H, O*t*Bu-2). ^{13}C NMR (101 MHz, $CDCl_3$) δ 168.9, 168.0, 164.9, 164.7, 151.7, 151.4, 148.2, 147.8, 137.0, 136.8, 125.6, 125.5, 124.1, 123.7, 75.3, 75.1, 73.7, 68.4, 64.0, 63.6, 63.2, 62.8, 58.6, 52.0, 51.8, 28.1 (3C), 28.0 (3C). HR-MS (ESI): *m/z:* calc. for $C_{15}H_{21}N_2O_4$ $[M+H]^+$: 293.1496, found: 293.1506.

12. Methyl (2*R*,3*S*)-3-(*tert*-butoxy)-1-picolinoylazetidine-2-carboxylate (*ent*-19)

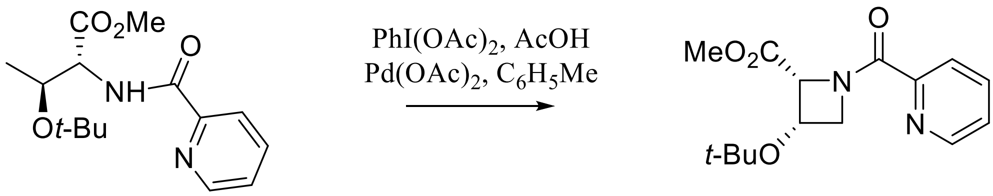

$C_{15}H_{22}N_2O_4$ 294.351 *ent*-**41**

$C_{15}H_{20}N_2O_4$ 292.335 *ent*-**19**

Procedure: In an argon flushed 250-mL round-bottom flask amide *ent*-**41** (1.20 g, 4.10 mmol, 1 eq.) was weighed in, followed by dry toluene (40 mL). PIDA (3.28 g, 10.2 mmol, 2.5 eq.), palladium acetate (45 mg, 0.20 mmol, 5 mol%) and acetic acid (0.47 mL, 8.2 mmol, 2 eq.) were added to the solution at room temperature. The reaction was subsequently heated to 104 °C in an oil bath, the reaction vessel was sealed and stirred at that temperature for 18 h.

Workup/Purification: After the indicated period of time the reaction was allowed to cool to rt and TLC showed complete conversion. The solution was concentrated to 1/8th volume and applied to a 30 g silica gel column using LP/EtOAc = 3:1 as eluent giving 1.04 g of azetidine *ent*-**19** as light-yellow crystals (88%, 3.57 mmol).

R_f = 0.15 (LP/EtOAc, 2:1). m.p.: 85 – 88 °C (LP/EtOAc). $[\alpha]_D^{20}$ = +67.4 (c 0.9, CH_2Cl_2), Mixture, ca. 3:1 of rotamers, minor rotamer („-1“) set as 1H in integrals: ^{1}H NMR (400 MHz, $CDCl_3$) δ 8.57 (ddd, *J* = 4.8, 1.8, 0.9 Hz, 1H, H-6‘-1), 8.52 – 8.39 (m, 3H, H-6‘-2), 8.14 (dt, *J* = 7.9, 1.1 Hz, 3H, H-5‘), 7.78 (td, *J* = 7.7, 1.8 Hz, 3H, H-4‘), 7.36 (ddd, *J* = 7.6, 4.8, 1.3 Hz, 1H, H-3‘-1), 7.32 (ddd, *J* = 7.6, 4.8, 1.3 Hz, 3H, H-3‘-2), 5.64 (dd, *J* = 7.5, 1.4 Hz, 3H, H-2-1), 5.09 (dd, *J* = 7.6, 1.3 Hz, 1H, H-2-2), 5.03 – 4.93 (m, 1H, H-3-2), 4.73 (td, *J* = 7.3, 5.8 Hz, 4H, H-4a), 4.64 (ddd, *J* = 10.1, 5.7, 1.3 Hz, 1H, H-4b-2), 4.40 (dd, *J* = 10.1, 7.1 Hz, 3H, H-3-1), 4.20 (ddd, *J* = 10.1, 5.8, 1.5 Hz, 3H, H-4b-1), 3.80 (s, 3H, H-11-2), 3.66 (s, 9H, H-11-1), 1.18 (s, 27H, O*t*Bu-1), 1.18 (s, 9H, O*t*Bu-2). ^{13}C NMR (101 MHz, $CDCl_3$) δ 168.9, 168.0, 164.9, 164.7, 151.7, 151.4, 148.2, 147.8, 137.0, 136.8, 125.6, 125.5, 124.1, 123.7, 75.3, 75.1, 73.7, 68.4, 64.0, 63.6, 63.2, 58.6, 52.0, 51.8, 27.98 (3C), 27.97 (3C). HR-MS (ESI): *m/z:* calc. for $NaC_{15}H_{20}N_2O_4$ $[M+Na]^+$: 315.1315, found: 315.1331.

13. (2*S*,3*S*)-3-(*tert*-Butoxy)azetidine-2-carboxylic acid (5)

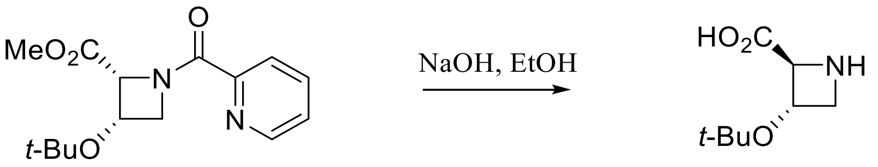

$C_{15}H_{20}N_2O_4$ *ent*-**19** 292.335 $C_8H_{15}NO_3$ **5** 173.212

Procedure: A 50-mL round bottom flask was charged with amide *ent*-**19** (760 mg, 2.60 mmol, 1.00 eq.) and dissolved in 2.5 mL "wet" ethanol (96%), followed by solid NaOH (520 mg, 13.0 mmol, 5.0 eq.). After 30 minutes full consumption of starting material was detected on TLC, and the reaction was heated to 70 °C in an oil bath. After 2 h the reaction was allowed to cool to room temperature.

Workup: The reaction mixture was diluted with distilled H_2O (35 mL) and extracted once with Et_2O (ca. 25 mL). The aqueous phase was neutralized to pH ~5 with 2M HCl and then extracted again with Et_2O. The aqueous phase was then evaporated to dryness to give 1.1 g of crude material.

Purification: The crude product was dissolved in MeOH/H_2O = 1:1, filtered over syringe filter and separated on the preparative HPLC (18-27% MeOH in H_2O) affording the product **5** (major, 5.2 min retention time) as a white solid weighing 335 mg (74%, 1.9 mmol) after lyophilization. The undesired isomer *ent*-**4** (65 mg, 14%) was also obtained.

5: m.p.: 185 – 187 °C. $[\alpha]_D^{20}$ = +23.4 (c 0.50, MeOH), ^{1}H NMR (600 MHz, CD_3OD) δ 4.58 (ddd, *J* = 6.7 Hz, 5.9 Hz, 5.9 Hz, 1H, H-3), 4.49 (d, *J* = 5.9 Hz, 1H, H-2), 4.09 (ddd, *J* = 10.7, 6.8, 0.9 Hz, 1H, H-4a), 3.76 (dd, *J* = 10.8, 5.9 Hz, 1H, H-4b), 1.23 (s, 9H, O*t*Bu). ^{13}C NMR (151 MHz, CD_3OD) δ 171.4, 76.7, 70.3, 68.6, 53.8, 28.4 (3C). HR-MS (ESI): *m/z:* calc. for $C_8H_{16}NO_3$ $[M+H]^+$: 174.1125, found: 174.1131.

14. (2*S*,3*R*)-3-(*tert*-Butoxy)azetidine-2-carboxylic acid (4)

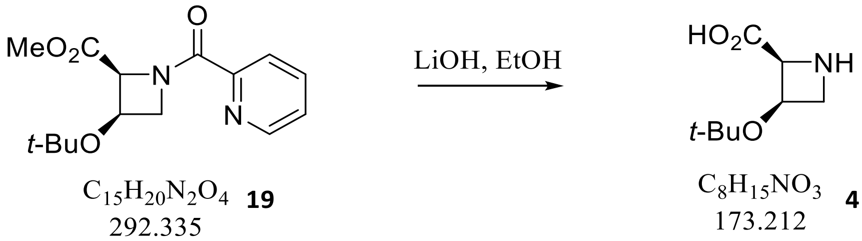

Procedure: The amide **19** (292 mg, 1 mmol) was weighed into a 25 mL round-bottom flask and dissolved in dry EtOH (10 mL). The solution was chilled to -16 °C in an ice/NaCl bath and stirred. LiOH solution was prepared (220 mg, 5 mmol, 5 eq. in 2.3 mL H_2O) and added dropwise to the reaction over 3 minutes. After 30 min the mixture was allowed to reach 0 °C the reaction and stirred for 2.5 hours. Then, the mixture was heated to 70°C in an oil bath and stirred at that temperature for 2 h.

Workup: After the indicated time the reaction was allowed to cool to room temperature and was acidified with 2M HCl (3 mL) to pH ~5 and evaporated to dryness to give 677 mg, ratio of isomers 62:38 (determined by ^{1}H-NMR, favoring desired product **4**).

Purification: The crude product was dissolved in 1:1 MeOH/H_2O, filtered over syringe filter and were separated on the preparative HPLC (16 → 25% MeOH in H_2O) affording the product **4** (major, 4 min retention time): 97 mg (56%, 0.2 mmol) as well as 60 mg of *ent*-**5** (35%).

4: m.p.: 210 °C (decomp.), $[\alpha]_D^{20}$ = -60.8 (c 0.33, MeOH), ^{1}H NMR (400 MHz, CD_3OD) δ 4.77 (ddd, *J* = 7.9, 6.8, 5.7 Hz, 1H, H-3), 4.70 (ddd, *J* = 7.8, 1.8, 0.8 Hz, 1H, H-2), 4.22 (ddd, *J* = 10.7, 6.8, 1.8 Hz, 1H, H-4a), 3.90 (ddd, *J* = 10.8, 5.7, 0.8 Hz, 1H, H-4b), 1.20 (s, 9H, O*t*Bu). ^{13}C NMR (101 MHz, D_2O) δ 169.6, 76.6, 67.5, 63.1, 54.6, 27.0 (3C). HR-MS (ESI): *m/z:* calc. for $C_8H_{16}NO_3$ $[M+H]^+$: 174.1125, found: m/z = 174.1131.

ent-**5**: m.p.: 186 °C (MeOH). $[\alpha]_D^{20}$ = -23.8 (c 0.90, MeOH), ^{1}H NMR (400 MHz, CD_3OD) δ 4.59 (ddd, *J* = 6.7, 6.0 Hz, 5.9 Hz, 1H, H-3), 4.50 (d, *J* = 5.9 Hz, 1H, H-2), 4.11 (dd, *J* = 11.2, 6.3 Hz, 1H, H-4a), 3.78 (dd, *J* = 10.8, 6.0 Hz, 1H, H-4b), 1.25 (s, 9H, O*t*Bu).^{13}C NMR (101 MHz, CD_3OD) δ 171.6, 76.7, 70.4, 68.7, 53.7, 28.4 (3C), HR-MS: m/z: calc. for $C_8H_{16}NO_3$ $[M+H]^+$: 174.1125, found: m/z = 174.1129. Crystal data (CCDC 2007994). $C_8H_{15}NO_3$, M = 173.21, monoclinic, a = 10.377(4). b = 5.955 (3), c = 14.753(6) Å, space group $P2_1$ (no. 4).

15. (2*R*,3*S*)-3-(*tert*-Butoxy)-1-picolinoylazetidine-2-carboxylic acid (42) and (2*S*,3*R*)-3-(*tert*-butoxy)azetidine-2-carboxylic acid (4)

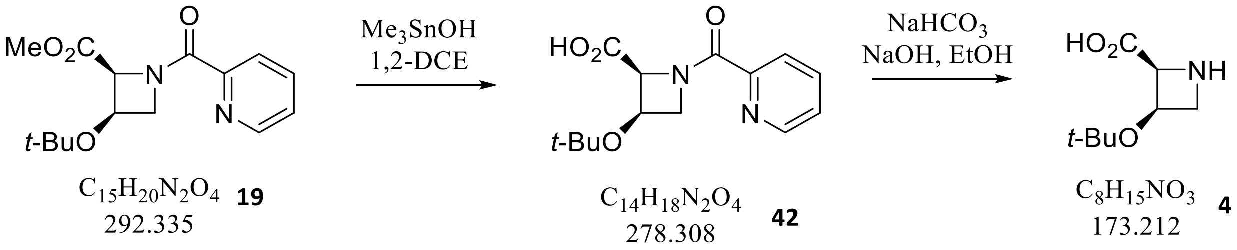

Procedure: Amide **19** (20 mg, 0.07 mmol, 1 eq.) was weighed into an 8-mL screw vial and dissolved in 0.3 mL dry 1,2-dichloroethane. The atmosphere was changed to argon and Me_3SnOH (111 mg, 0.615 mmol, 9 eq.) was introduced in one portion. The resulting suspension was heated to 80 °C for 22 hours.

Workup: The reaction was diluted with 20 mL $CHCl_3$ and washed three times with 0.02 M $KHSO_4$ solution and once with brine. The organic phase was evaporated to dryness to give crude product **42**.

Purification: Amide **42** can be used as a crude material in the next step. An analytically pure sample was acquired by preparative HPLC (60 to 65% methanol in water, 0.1% formic acid) and gave 14 mg (74%, 0.05 mmol) of **42** in a pure form.

$[\alpha]_D^{20}$ = -71.8 (c 0.65, MeOH). Mixture, ca. 3:1 of rotamers, minor rotamer („-2“) set as 1H in integrals, ^{1}H NMR (600 MHz, $CDCl_3$) δ 8.58 (s, 1H, H-6’-2), 8.47 – 8.40 (m, 3H, H-6’-1), 8.13 (d, *J* = 7.8 Hz, 4H, H-5’), 7.78 (t, *J* = 7.7 Hz, 4H, H-4’), 7.37 (s, 1H, H-3’-2), 7.31 (d, *J* = 6.2 Hz, 3H, H-3’-1), 5.68 – 5.53 (m, 3H, H-2-1), 5.12 (d, *J* = 7.6 Hz, 1H, H-2-2), 4.94 (d, *J* = 9.3 Hz, 1H, H-4a-2), 4.75 (q, *J* = 6.9 Hz, 3H, H-3), 4.64 (d, *J* = 9.7 Hz, 1H, H-4b-2), 4.41 (dd, *J* = 10.1, 7.0 Hz, 3H, H-4a-1), 4.24 – 4.11 (m, 3H, H-4b-1), 1.18 (s, 27H, O*t*Bu). ^{13}C NMR (151 MHz, $CDCl_3$) major rotamer δ 173.4, 164.9, 151.1, 147.7, 136.9, 125.6, 123.7, 75.4, 73.4, 63.2, 58.6, 27.8 (3C). minor rotamer δ 173.4, 164.9, 151.1, 148.2, 136.9, 125.7, 124.2, 75.4, 68.3, 64.0, 62.8, 27.8 (3C). HR-MS (ESI): *m/z:* calc. for $C_{14}H_{19}N_2O_4$ $[M+H]^+$: 279.1340, found: 279.1341.

Procedure: Crude compound **42** (17 mg, ca. 90% purity, 55 μmol) was dissolved in dry ethanol (2 mL) and chilled to -15 °C (ice/NaCl). $NaHCO_3$ (9 mg, 0.11 mmol, 2 eq.) dissolved in a few drops of water (ca. 0.15 mL) was then added. After stirring for 15 minutes at -15 °C sodium hydroxide as an ethanolic solution (0.15 mL,78 mg/mL solution in 80% EtOH, 0.22 mmol, 4 eq.) was added dropwise and the reaction was allowed to reach 0 °C over 15 minutes. The reaction mixture was then heated to 70 °C for 2 hours.

Workup: Afterwards the reaction was cooled to room temperature and neutralized with 2M HCl (pH = 6). The solution was evaporated to dryness to give crude product as a colorless solid.

Purification: The crude material was purified on prep HPLC using 18-27% methanol in water to give fractions which were lyophilized to give 7 mg of product **4** (72% yield, 0.04 mmol).

For analytical data see **14.**

16. (*S*)-3-Aminodihydrofuran-2(3H)-one hydrochloride (7)

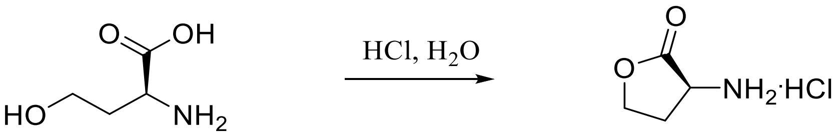

$C_4H_9NO_3$ **43**
119.120

$C_4H_8ClNO_2$ **7**
137.563

Procedure: L-Homoserine **43** (1.00 g, 8.14 mmol, 1 eq.) was charged in a 25-mL round-bottom flask and dissolved in 3M hydrochloric acid (6.0 mL, 57 mmol, 7 eq.). Afterwards the reaction was heated to reflux for 5 h followed by sealing the reaction vessel and stirring at room temperature for 68 h.

Workup: The reaction mixture was evaporated to dryness, adding minimal amount of MeOH in order to remove the water azeotropically. The product **7** solidified to white crystalline material that weighed 1.20 g (99%, 8.11 mmol) after drying at Schlenk vacuum.

m.p.: 223 – 226 °C (decomp.), Lit: 227 °C (decomp.) [10]. $[\alpha]_D^{20}$ = -26.8 (c 0.80, water), ^1H NMR (400 MHz, MeOD) δ 4.53 (ddd, *J* = 9.1, 1.1 Hz, 1H, H-4a), 4.38 (ddd, *J* = 11.0, 9.2, 6.0 Hz, 1H, H-2), 4.36 (dd, *J* = 11.6, 8.9 Hz, 1H, H-4b), 2.74 (dddd, *J* = 12.5, 8.9, 6.0, 1.2 Hz, 1H, H-3a), 2.35 (dddd, *J* = 12.5, 11.6, 11.0, 9.1 Hz, 1H, H-3b).^{13}C NMR (101 MHz, CD_3OD) δ 172.7, 66.0, 48.1, 27.0. HR-MS (ESI): Calc. *m/z* 102.0550 $[M\text{-}Cl]^+$, found: 102.0551.

Synthesis of dimeric compounds **23** and **25**

17. *tert*-Butyl (2*S*,3*S*,*E*)-2-(((S)-3,4-di-*tert*-butoxy-4-oxobutyl)amino)-3-hydroxyhex-4-enoate (24)

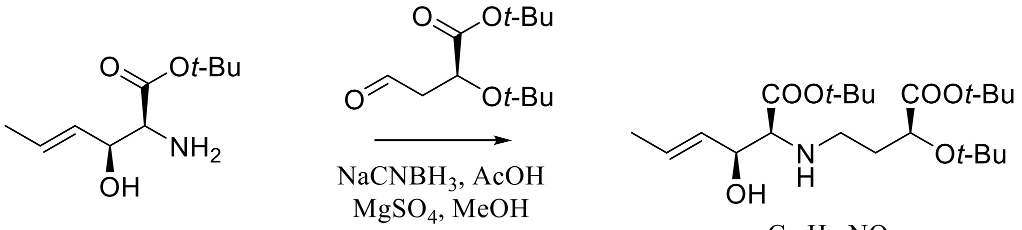

$C_{10}H_{19}NO_3$ 201.266 **2**

$C_{22}H_{41}NO_6$ 415.571 **24**

Procedure: A flame-dried Schlenk flask was charged with amine **2** (1.14 g, 5.64 mmol, 1 eq.) in 45 mL dry methanol followed by anhydrous $MgSO_4$ (1.35 g) and aldehyde **1** (1.29 g, 5.60 mmol, 0.97 eq.) in dry methanol (11 mL) at 0 °C. Glacial acetic acid (0.64 mL, 11.2 mmol, 2 eq.) was added shortly after followed by $NaCNBH_3$ (495 mg, 7.8 mmol, 1.4 eq.) as a solid in small portions. The reaction was stirred at room temperature for 2 h until no amine was present on TLC (CH_2Cl_2/MeOH = 95:5).

Workup: The reaction mixture was quenched by the addition of H_2O (70 mL) and saturated $NaHCO_3$ solution (35 mL). It was extracted twice with EtOAc (100 mL) and Et_2O (2× 60 mL). The combined organic phases were washed with brine (15 mL), dried over $MgSO_4$ and concentrated under reduced pressure to dryness to give 3.24 g of crude material.

Purification: The crude material was purified on 100 g silica gel using LP/Et_2O = 1.5:1 + 0.5% triethylamine as eluent to give 1.92 g of product **24** (83%, 4.63 mmol).

R_f: 0.52 (CH_2Cl_2/MeOH = 20:1). $[\alpha]_D^{20}$ = -42.0 (c 0.56, CH_2Cl_2). ^{1}H NMR (600 MHz, $CDCl_3$) δ 5.77 (dd, *J* = 15.0, 6.9 Hz, 1H, H-5'), 5.38 (dd, *J* = 14.2, 5.5 Hz, 1H, H-4'), 4.43 (m, 1H, H-3'), 4.05 (dd, *J* = 5.3 Hz, 1H, H-2), 3.44 (m, 1H, H-2'), 2.95 (m, 1H, H-4a), 2.82 (m, 1H, H-4b), 2.00 (m, 1H, H-3a), 1.88 (m, 1H, H3-b), 1.68 (d, *J* = 6.4 Hz, 4H, H-6'), 1.46 (s, 9H, COO*t*Bu), 1.46 (s, 9H, C'OO*t*Bu), 1.20 (s, 9H, O*t*Bu). ^{13}C NMR (151 MHz, $CDCl_3$) δ 173.3, 169.4, 129.2, 128.0, 83.1, 81.4, 75.7, 70.9, 70.2, 66.2, 45.4, 32.8, 28.2 (3C), 28.0 (3C), 27.9 (3C), 17.8. HR-MS (ESI): *m/z:* calc. for $NaC_{22}H_{41}NO_6$ $[M+Na]^+$: 438.2826, found: m/z = 438.2839.

18. *tert*-Butyl (2*S*,3*S*,*E*)-2-((*tert*-butoxycarbonyl)((S)-3,4-di-tert-butoxy-4-oxobutyl) amino)-3-hydroxyhex-4-enoate (25)

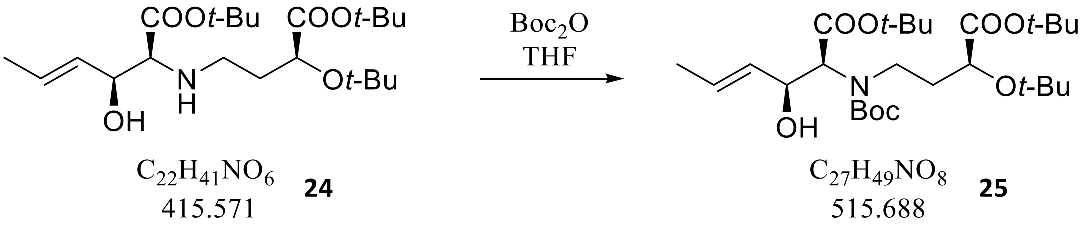

24 **25**

Procedure: A 250-mL round-bottom flask was charged with amine **24** (2.46 g, 5.93 mmol, 1 eq.) dissolved in THF (75 mL). Boc_2O (5.18 g, 23.7 mmol) was added dropwise dissolved in THF (10 mL). The mixture was heated to 40 °C and stirred for 24 h.

Workup: The solution was concentrated under reduced pressure and was directly applied to the column for purification.

Purification: The crude material was purified over 110 g silica gel using LP/EtOAc = 10:1 as eluent to give 2.57 g (84%, 5.00 mmol) of product **25** as a thick colorless oil.

R_f: 0.59 (LP/EtOAc = 3:1), $[\alpha]_D^{20}$ = -54.8 (c 1.1, MeOH), Mixture of rotamers, ca 55:45: ^{1}H NMR (600 MHz, $CDCl_3$) δ 5.73 (ddd, *J* = 15.4, 9.1, 6.3 Hz, 1H, H-5'), 5.50 – 5.36 (m, 1H, H-4'), 4.76 (app t, *J* = 7.8 Hz, 1H, H-3'-1), 4.70 (app t, *J* = 8.1 Hz, 1H, H-3'-2), 4.10 – 4.02 (m, 1H, OH), 3.83 (ddt, *J* = 17.1, 8.8, 4.6 Hz, 1H, H-2), 3.53 (ddd, *J* = 15.2, 9.5, 6.3 Hz, 1H, H-4a-1), 3.44 (ddd, *J* = 14.1, 10.7, 5.1 Hz, 1H, H-4a-2), 3.38 (d, *J* = 8.2 Hz, 1H, H-2'), 3.11 (ddd, *J* = 13.9, 9.8, 6.3 Hz, 1H, H-4b-1), 2.98 (ddd, *J* = 14.2, 10.6, 5.3 Hz, 1H, H-4b-2), 1.87 (dddd, *J* = 19.3, 10.6, 9.9, 4.5 Hz, 2H, H-3), 1.70 (d, *J* = 6.5 Hz, 3H, H-6'), 1.56 – 1.31 (m, 27H, COO*t*Bu, C'OO*t*Bu, Boc), 1.18 (s, 9H, O*t*Bu). ^{13}C NMR (151 MHz, $CDCl_3$) δ 173.7, 171.5, 155.0, 154.2, 129.7, 129.2, 82.8, 82.5, 81.0, 80.4, 74.9, 74.8, 71.8, 71.6, 70.3, 70.1, 66.7, 66.1, 47.6, 47.4, 33.2, 32.6, 28.53 (3C), 28.46 (3C), 28.2 (3C), 28.1 (3C), 28.0 (3C), 18.0. HR-MS (ESI): Calc. *m/z* 516.3531 $[M+H]^+$, found: 516.3538.

19. *tert*-Butyl (*S*)-2-(((*S*)-3,4-di-*tert*-butoxy-4-oxobutyl)amino)pent-4-enoate (22)

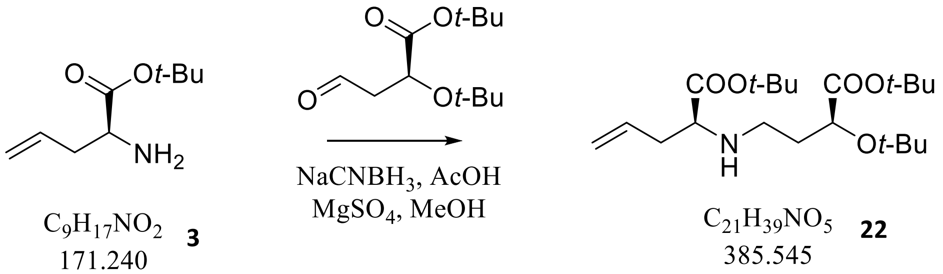

$C_9H_{17}NO_2$ **3**
171.240

$C_{21}H_{39}NO_5$ **22**
385.545

Procedure: A flame-dried Schlenk flask was charged with amine **3** (0.40 g, 2.3 mmol, 1.05 eq.) in dry methanol (5 mL) followed by anhydrous $MgSO_4$ (0.9 g) and aldehyde **1** (0.51 g, 2.2 mmol, 1 eq.) in dry methanol (2 mL) at 0 °C. Glacial acetic acid (0.25 mL, 4.4 mmol, 2 eq.) was added shortly after followed by $NaCNBH_3$ (0.19 g, 3.1 mmol, 1.4 eq.) as a solid in small portions. The reaction was stirred at room temperature for 1 hour until no aldehyde or amine was present on TLC (CH_2Cl_2/MeOH = 95:5).

Workup: H_2O as well as saturated $NaHCO_3$ solution (10 mL each) were added and the reaction was extracted with EtOAc (3× 50 mL) and Et_2O (30 mL). Combined organic phases were washed with brine (10 mL), dried over $MgSO_4$ and evaporated to give 950 mg crude material.

Purification: The crude material was purified on 50 g silica gel using LP/Et_2O = 3:1 + 0.5% Et_3N and the product fractions were evaporated to give 0.68 g of dimer **22** (80%, 1.8 mmol)

R_f = 0.31 (LP/EtOAc = 6:1), $[\alpha]_D^{20}$ = -4.1 (c 1.3, $CHCl_3$), ^{1}H NMR (600 MHz, $CDCl_3$) δ 5.80 – 5.68 (m, 1H, H-4'), 5.08 (dd, *J* = 17.1, 1.9 Hz, 1H, H-5b'), 5.07 – 5.02 (m, 2H, H-5a'), 3.92 (dd, *J* = 6.9, 5.9 Hz, 1H, H-2), 3.18 (app t, *J* = 6.4 Hz, 1H, H-2'), 2.76 – 2.65 (m, 1H, H-3a), 2.62 – 2.55 (m, 1H, H-3b), 2.35 (t, *J* = 6.8 Hz, 2H, H-4), 1.79 – 1.71 (m, 2H, H-3'), 1.44 (s, 9H, COO*t*Bu), 1.43 (s, 9H, C'OO*t*Bu), 1.15 (s, 9H, O*t*Bu).^{13}C NMR (151 MHz, $CDCl_3$) δ 174.0, 173.9, 133.9, 117.9, 81.3, 80.8, 75.1, 70.2, 61.3, 44.1, 37.8, 34.3, 28.3 (3C), 28.1 (3C), 28.0 (3C). HR-MS (ESI): Calc. *m/z* 386.2901 $[M+H]^+$, found: 386.2917.

20. *tert*-Butyl (*S*)-2-((*tert*-butoxycarbonyl)((*S*)-3,4-di-*tert*-butoxy-4-oxobutyl)amino) pent-4-enoate (23)

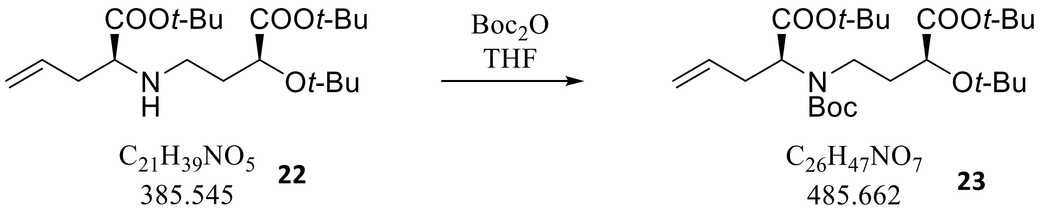

Procedure: A 100-mL round-bottom flask was charged with starting material **22** (640 mg, 1.66 mmol, 1 eq.) dissolved in THF (20 mL). Boc_2O (1.45 g, 6.64 mmol, 4 eq.) was added dropwise dissolved in THF (5 mL). The mixture was heated to 40 °C and stirred for 24 h.

Workup: The solution was concentrated under reduced pressure and was directly applied to the column for purification.

Purification: The crude material was purified *via* column chromatography over 40 g silica gel using LP/EtOAc = 11:1 as eluent to give 769 mg (95%, 1.58 mmol) of the title compound **23** as a thick colorless oil.

R_f = 0.4 (CH_2Cl_2/MeOH = 20:1). $[\alpha]_D^{20}$ = -28.8 (c 0.7, $CHCl_3$), Mixture of rotamers: ^{1}H NMR (600 MHz, $CDCl_3$) δ 5.85 – 5.70 (m, 1H, H-4'), 5.10 (d, *J* = 1.5 Hz, 1H, H-5a'), 5.08 (d, *J* = 1.5 Hz, 1H, H-5b'), 4.15 (d, *J* = 4.5 Hz, 1H, H-2'-1), 3.90 – 3.82 (m, 1H, H-2'-2), 3.82 – 3.77 (m, 1H, H-2), 3.44 – 3.31 (m, 1H, H-4a-1, H-4b-2), 3.30 – 3.15 (m, 1H, H-4a-2), 3.06 – 2.96 (m, 1H, H-4b-1), 2.75 – 2.65 (m, 1H, H-3a), 2.60 – 2.51 (m, 1H, H-3b), 1.89 (d, *J* = 11.9 Hz, 2H, H-3'), 1.44 (s, 27H, COO*t*Bu, C'OO*t*Bu, Boc), 1.16 (s, 9H, O*t*Bu) ^{13}C NMR (151 MHz, $CDCl_3$) δ 173.8, 173.7, 170.5, 170.4, 155.5, 155.1, 135.0, 117.6, 117.5, 81.44, 81.37, 80.9, 80.4, 80.0, 77.4, 77.0, 74.8, 74.7, 70.5, 70.4, 61.3, 60.8, 45.4, 44.6, 34.6, 33.9, 33.8, 32.8, 28.5 (3C), 28.2 (3C), 28.11 (3C), 28.08 (3C), 28.0 (3C). HR-MS (ESI): calc. for $NaC_{26}H_{47}NO_7$ $[M+Na]^+$: 508.3245, found: 508.3248,

Synthesis of trimers **28-33**, **44-45**

21. *tert*-Butyl (2*S*,3*R*)-2-((*tert*-butoxycarbonyl)((*S*)-3,4-di-*tert*-butoxy-4-oxobutyl) amino)-3-hydroxy-4-oxobutanoate (27)

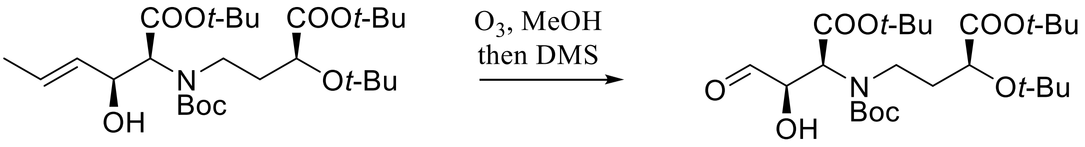

$C_{27}H_{49}NO_8$ 515.688 **25**

$C_{25}H_{45}NO_9$ 503.633 **27**

Procedure: A Schlenk flask containing a solution of olefin **25** (245 mg, 0.480 mmol) in methanol (9 mL) was chilled to -78 °C in an acetone/N_2 bath and ozone in oxygen was bubbled through the reaction until the reaction retained a blue color (ca. 2 min). Immediately after this event oxygen was bubbled through the reaction for 2 minutes (until coloration subsided). After 15 minutes there was added dimethylsulfide (0.13 mL, 1.9 mmol, 4 eq.) and the reaction was allowed to reach room temperature over night.

Workup: The reaction mixture was concentrated and the crude aldehyde **27** was dried under vacuum (< 0.1 mbar) to constant weight, 254 mg (106%, contaminated with residual DMSO). This crude material was used directly in reductive amination reactions.

22. *tert*-Butyl (2*S*)-2-((*tert*-butoxycarbonyl)((*S*)-3,4-di-*tert*-butoxy-4-oxobutyl) amino)-4-oxobutanoate (26)

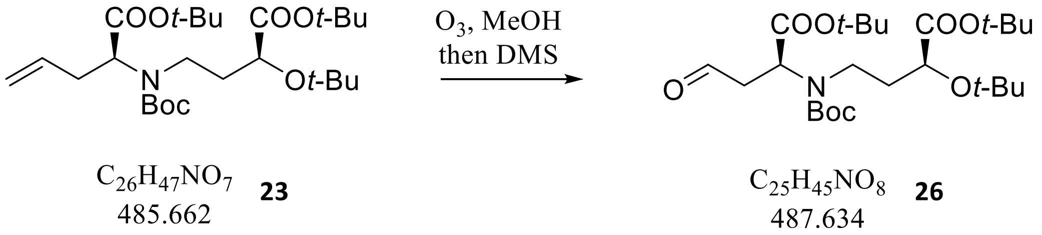

23 **26**

Procedure: A Schlenk flask containing a solution of olefin **23** (72 mg, 0.15 mmol) in methanol (5 mL) was chilled to -78 °C in an acetone/N_2 bath and ozone in oxygen was bubbled through the reaction until the reaction retained a blue color (ca. 1 min). Immediately after this event argon was bubbled through the reaction for 2 minutes (until coloration subsided). After 15 minutes there was added dimethylsulfide (37 mg, 0.60 mmol, 4 eq.) and the reaction was allowed to reach room temperature over night.

Workup: The reaction mixture was concentrated and the crude aldehyde **26** was dried under vacuum (< 0.1 mbar) to constant weight, 72 mg (99%, 1.5 mmol) This crude material was used directly in reductive amination reactions.

^{1}H NMR (600 MHz, $CDCl_3$) δ 9.80 (d, *J* = 9.2 Hz, 1H, CHO), 4.30 (t, *J* = 6.4 Hz, 1H, H-2), 3.88 – 3.77 (m, 1H, H-2'), 3.49 (s, 1H, H-3'a), 3.44 – 3.32 (m, 1H, H-4a), 3.15 (td, *J* = 14.4, 12.6, 5.6 Hz, 1H, H-3'b), 2.88 (dd, *J* = 18.0, 6.6 Hz, 1H, H-4b), 1.89 (dd, *J* = 13.8, 5.7 Hz, 2H, H-3), 1.47 (s, 9H, Boc), 1.45 (s, 9H, C'OO*t*Bu), 1.43 (s, 9H, COO*t*Bu), 1.18 (s, 9H, O*t*Bu).

23. (*S*)-1-((2*S*,3*S*)-4-(*tert*-Butoxy)-3-((*tert*-butoxycarbonyl)((*S*)-3,4-di-*tert*-butoxy-4-oxobutyl)amino)-2-hydroxy-4-oxobutyl)azetidine-2-carboxylic acid (33)

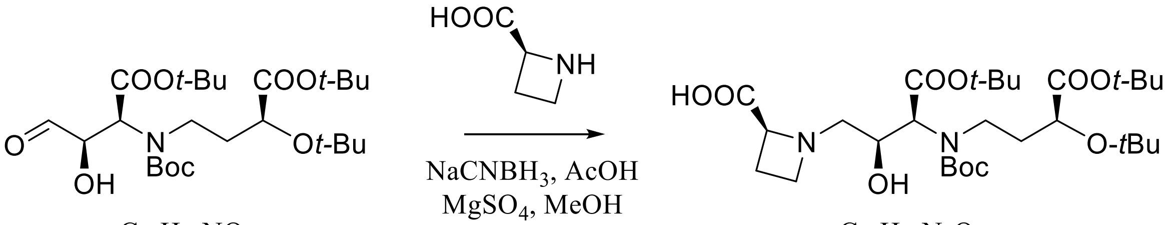

$C_{25}H_{45}NO_9$ 503.633 **27**

$C_{29}H_{52}N_2O_{10}$ 588.739 **33**

Procedure: In a 25-mL round bottom flask aldehyde **27** (0.13 g, 95% purity, 0.23 mmol) was dissolved in dry methanol (4 mL) and $MgSO_4$ (0.5 g) was added. To the suspension was added azetidine **6** (30 mg, 0.30 mmol, 1.3 eq.) as a solid, followed by acetic acid (0.02 mL, 0.34 mmol, 1.5 eq.). After 10 minutes stirring at 0 °C the reducing agent was added in one portion. After addition of $NaCNBH_3$ (21 mg, 0.34 mmol, 1.5 eq.) the reaction was allowed to warm to rt. After 3 h the reaction was worked up.

Workup: The mixture was quenched with water (0.5 mL) filtered over Celite and evaporated to dryness at 30 °C in a water bath.

Purification: The crude material was directly purified on the preparative HPLC using 80-98% methanol in water and 0.1% formic acid, to give fractions which were lyophilized to give 100 mg of product **33** (75%, 0.17 mmol).

$[\alpha]_D^{20}$ = -80.0 (c 1.0, MeOH). ^{1}H NMR (600 MHz, CD_3OD) δ 6.36 (dd, *J* = 9.4 Hz, 1H, H-2"), 6.00 (dd, *J* = 8.7 Hz, 1H, H-3'), 5.60 (dd, *J* = 9.5 Hz, 1H, H-3"a), 5.48 (dd, *J* = 9.1, 3.2 Hz, 1H, H-3"b), 5.45 (d, *J* = 2.3 Hz, 1H, H-2), 5.40 (d, *J* = 5.7 Hz, 1H, H-2'), 5.24 (ddd, *J* = 15.4, 11.5, 4.7 Hz, 1H, H-4a), 5.01 – 4.95 (m, 1H, H-4'a), 4.83 (d, *J* = 12.2 Hz, 1H, H-4'b), 4.75 – 4.59 (m, 1H, H-4b), 4.23 (d, *J* = 10.3 Hz, 1H, H-4"a), 4.11 (dd, *J* = 14.9, 6.0 Hz, 1H, H-4"b), 3.59 – 3.51 (m, 1H, H-3a), 3.44 (dddd, *J* = 13.4, 11.2, 8.8, 4.8 Hz, 1H, H-3b), 3.05 (s, 9H, COO*t*Bu), 3.04 (s, 9H, COO*t*Bu), 3.02 (s, 9H, Boc), 2.75 (s, 9H, O*t*Bu).^{13}C NMR (151 MHz, CD_3OD) δ 175.7, 172.7, 169.9, 156.9, 83.3, 82.5, 82.1, 76.0, 71.2, 68.7, 67.7, 66.9, 66.1, 58.1, 51.3, 49.0, 34.0, 28.8, 28.3, 28.2, 28.2, 22.9, 15.5. HR-MS (ESI): *m/z:* calc. for $C_{29}H_{53}N_2O_{10}$ $[M+H]^+$: 589.3695, found: m/z = 589.3705.

24. (*S*)-1-((2*S*,3*S*)-4-(*tert*-Butoxy)-3-((*tert*-butoxycarbonyl)((*S*)-3,4-di-*tert*-butoxy-4-oxobutyl)amino)-2-hydroxy-4-oxobutyl)azetidine-2-carboxylic acid (31)

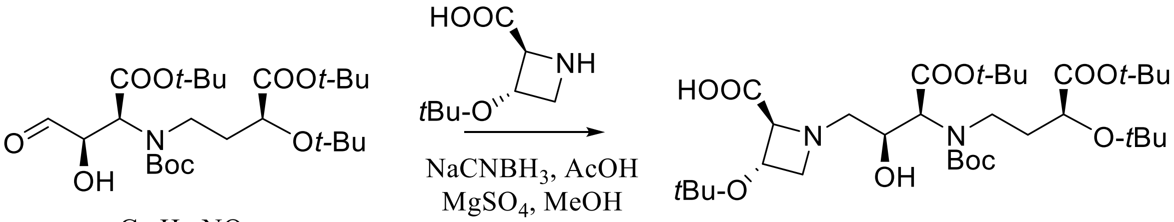

$C_{25}H_{45}NO_9$ 503.633 **27**

$C_{33}H_{60}N_2O_{11}$ 660.846 **31**

Procedure: In a 25-mL round bottom flask aldehyde **27** (76 mg, 90% purity, 0.13 mmol) was dissolved in 3 mL dry methanol and $MgSO_4$ (0.3 g) was added. To the suspension was added azetidine **5** (29 mg, 0.17 mmol, 1.3 eq.) as a solid, followed by acetic acid (0.01 mL, 0.19 mmol, 1.5 eq.). After 10 minutes stirring at 0 °C the reducing agent was added in one portion. After addition of $NaCNBH_3$ (11 mg, 0.18 mmol, 1.4 eq.) the reaction was allowed to warm to rt. After 2 h the reaction was worked up.

Workup: The mixture was quenched with water (0.4 mL) filtered over celite and evaporated to dryness at 35 °C in a water bath.

Purification: The crude material was directly purified on the preparative HPLC using 80-98% methanol and 0.1% formic acid, to give fractions which were lyophilized to give 66 mg of product **31** (77%, 0.1 mmol) as a colorless oil.

$[\alpha]_D^{20}$ = -42.7 (c 0.98, MeOH), ^{1}H NMR (600 MHz, CD_3OD, mixture of rotamers ca. 7:1, shifts given for major rotamer) δ 4.56 – 4.49 (m, 2H, H-2", H-3"), 4.50 – 4.44 (m, 1H, H-3'), 4.37 (dd, *J* = 10.8, 4.6 Hz, 1H, H-4"a), 3.91 (dd, *J* = 8.9, 3.6 Hz, 1H, H-2), 3.85 (d, *J* = 6.0 Hz, 1H, H-2'), 3.71 – 3.65 (m, 1H, H-4a), 3.64 – 3.57 (m, 1H, H-4"b), 3.46 – 3.39 (m, 1H, H4'-a), 3.35 – 3.33 (m, 1H, H4'-b), 3.11 (ddd, *J* = 13.9, 11.1, 4.9 Hz, 1H, H-4b), 1.99 (dddd, *J* = 14.2, 11.4, 4.5 Hz, 1H, H-3a), 1.88 (dddd, *J* = 13.5, 11.3, 8.9, 4.8 Hz, 1H, H-3b), 1.50 (s, 9H, Boc), 1.48 (s, 9H, COO*t*Bu), 1.46 (s, 9H, COO*t*Bu), 1.23 (s, 9H, O*t*Bu), 1.19 (s, 9H, O*t*Bu). ^{13}C NMR (151 MHz, CD_3OD) δ 175.7, 171.4, 169.9, 156.9, 83.3, 82.4, 82.2, 78.1, 76.8, 76.0, 71.2, 67.9, 66.3, 66.1, 61.3, 59.3, 49.9, 34.0, 28.8, 28.4, 28.3, 28.3, 28.2. HR-MS (ESI): *m/z:* calc. for $C_{33}H_{61}N_2O_{11}$ $[M+H]^+$: 661.4270, found: m/z = 661.4290.

25. (*S*)-1-((2*S*,3*R*)-4-(*tert*-Butoxy)-3-((*tert*-butoxycarbonyl)((*S*)-3,4-di-*tert*-butoxy-4-oxobutyl)amino)-2-hydroxy-4-oxobutyl)azetidine-2-carboxylic acid (32)

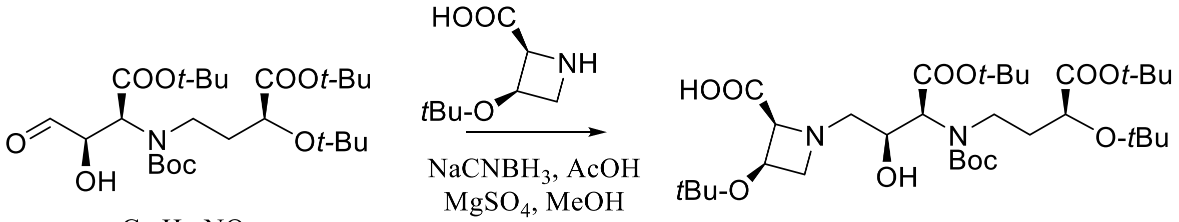

$C_{25}H_{45}NO_9$ 503.633 **27**

$C_{33}H_{60}N_2O_{11}$ 660.846 **32**

Procedure: In a 25-mL round bottom flask aldehyde **27** (40 mg, 95% purity, 0.08 mmol, 1 eq.) was dissolved in dry methanol (2 mL) and $MgSO_4$ (0.5 g) was added. To the suspension was added azetidine **4** (17 mg, 0.1 mmol, 1.3 eq.) as a solid at 0 °C, followed by acetic acid (0.01 mL, 0.16 mmol, 2 eq.). After 5 minutes stirring at 0 °C the reducing agent was added in one portion. After addition of $NaCNBH_3$ (8.3 mg, 0.12 mmol, 1.5 eq.) the reaction was allowed to warm to rt. After 3 h the reaction was worked up.

Workup: The mixture was filtered over celite and evaporated to dryness at 30 °C in a water bath.

Purification: The crude material was directly purified on the preparative HPLC using 80-98% methanol and 0.1% formic acid, to give fractions which were lyophilized to give 35 mg of product **32** (68%, 0.05 mmol) as a colorless oil.

$[\alpha]_D^{20}$ = -63.8 (c 1.3, CH_2Cl_2), ^{1}H NMR (600 MHz, $CDCl_3$) δ 4.73 (ddd, *J* = 7.9, 5.4 Hz, 1H, H-3''), 4.61 (dd, *J* = 11.8, 5.4 Hz, 1H, H-4''a), 4.52 (d, *J* = 8.0 Hz, 1H, H-2''), 4.35 (dd, *J* = 9.5, 3.7 Hz, 1H, H-3'), 4.17 – 4.10 (m, 1H, H-4''b), 3.89 – 3.82 (m, 1H, H-2'), 3.78 (d, *J* = 12.2 Hz, 1H, H-4a), 3.76 – 3.75 (m, 1H, H-4'a), 3.73 (dd, *J* = 9.2, 3.4 Hz, 1H, H-2), 3.15 (ddd, *J* = 14.1, 11.4, 5.1 Hz, 1H, H-4b), 2.95 (dd, *J* = 12.6, 9.5 Hz, 1H, H-4'b), 1.94 – 1.86 (m, 1H, H-3a), 1.86 – 1.77 (m, 1H, H-3b), 1.44 (s, 9H, Boc), 1.42 (s, 9H, COO*t*Bu), 1.41 (s, 9H, COO*t*Bu), 1.17 (s, 9H, O*t*Bu), 1.15 (s, 9H, O*t*Bu). ^{13}C NMR (151 MHz, $CDCl_3$) δ 173.7, 168.4, 165.8, 155.4, 81.6, 80.7, 80.5, 76.1, 75.6, 74.5, 69.8, 66.9, 64.1, 61.0, 60.5, 59.8, 48.5, 32.2, 28.3, 27.96, 27.93, 27.89, 27.87. HR-MS (ESI): *m/z* calc. for $C_{29}H_{53}N_2O_{10}$ $[M+H]^+$: 661.4270, found: 661.4293.

26. *tert*-Butyl (2*S*,3*S*)-2-((*tert*-butoxycarbonyl)((*S*)-3,4-di-*tert*-butoxy-4-oxobutyl) amino)-3-hydroxy-4-(((*S*)-2-oxotetrahydrofuran-3-yl)amino)butanoate (44)

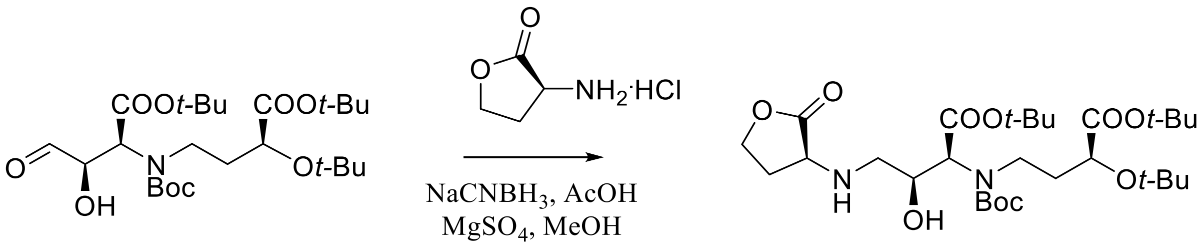

$C_{25}H_{45}NO_9$ **27**
503.633

$C_{29}H_{52}N_2O_{10}$ **44**
588.739

Procedure: In a 50-mL round bottom flask aldehyde **27** (224 mg, 0.44 mmol, 1 eq.) was dissolved in dry methanol (8 mL) and small amount of $MgSO_4$ (0.5 g) was added. To the suspension was added lactone **7** (73 mg, 0.53 mmol, 1.2 eq.) as a solid, followed by acetic acid (0.03 mL, 0.44 mmol, 1 eq.) at 0 °C. After 10 minutes stirring at room temperature the reducing agent was added (39 mg, 0.62 mmol, 1.4 eq) and the reaction was allowed to reach room temperature. After 3 h the reaction was worked up.

Workup: The reaction mixture was filtered and evaporated to dryness at 30 °C in a water bath.

Purification: The crude was purified on a 19 g silica gel column using LP/EtOAc = 1.5:1 as eluent to give 170 mg of product **44** (65%, 0.29 mmol) as a colorless oil.

R_f = 0.11 (LP/EtOAc = 2:1). $[\alpha]_D^{20}$ = -48.7 (c 0.43, CH_2Cl_2), Mixture of rotamers 60:40, some ^{13}C-signals doubled. ^1H NMR (600 MHz, CD_3OD) δ 4.40 (ddd, *J* = 8.8, 2.2 Hz, 1H, H-4''a), 4.35 – 4.27 (m, 1H, H-2'), 4.24 (ddd, *J* = 10.2, 9.0, 6.2 Hz, 1H, H-4''b), 4.02 – 3.93 (m, 1H, H-3'), 3.93 – 3.89 (m, 1H, H-2), 3.65 (dd, *J* = 9.3 Hz, 1H, H-2''), 3.61 – 3.53 (m, 1H, H-4a), 3.19 (ddd, *J* = 14.2, 11.2, 4.9 Hz, 1H, H-4b), 2.90 – 2.80 (m, 1H, H-4'a), 2.79 – 2.71 (m, 1H, H-4'b), 2.54 (dddd, *J* = 12.4, 8.3, 6.3, 2.2 Hz, 1H, H-3''a), 2.08 (m, 1H, H-3''b), 1.96 – 1.93 (m, 1H, H-3a), 1.88 (dddd, *J* = 13.4, 11.2, 8.7, 4.9 Hz, 1H, H-3b), 1.50 (s, 9H, Boc), 1.48 (s, 9H, COO*t*Bu), 1.46 (s, 9H, COO*t*Bu), 1.19 (s, 9H, O*t*Bu). ^{13}C NMR (151 MHz, CD_3OD) δ 177.8, 174.4, 174.3, 169.9, 169.7, 155.5, 154.6, 81.8, 81.6, 81.1, 80.5, 80.5, 74.7, 74.6, 70.1, 69.9, 69.4, 69.3, 65.8, 63.8, 55.9, 49.5, 48.5, 33.0, 29.6, 27.4, 27.0, 26.8, 26.8. HR-MS (ESI): *m/z* calc. for $NaC_{29}H_{52}N_2O_{10}$ $[M+Na]^+$: 611.3514, found: 611.3534.

27. (*S*)-1-((*S*)-4-(*tert*-Butoxy)-3-((*tert*-butoxycarbonyl)((*S*)-3,4-di-*tert*-butoxy-4-oxobutyl)amino)-4-oxobutyl)azetidine-2-carboxylic acid (30)

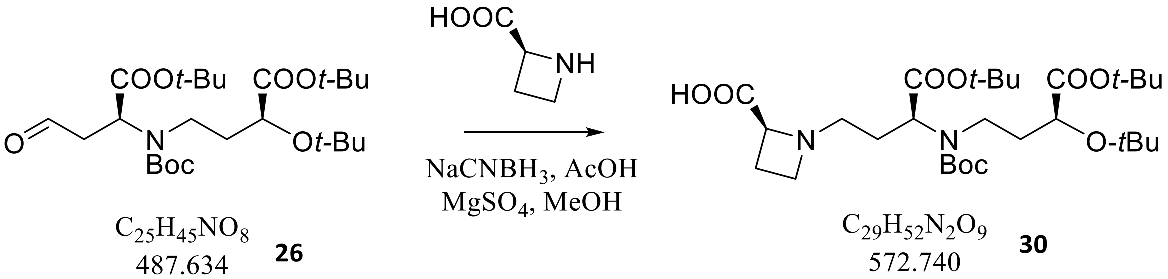

$C_{25}H_{45}NO_8$
487.634 **26**

$C_{29}H_{52}N_2O_9$
572.740 **30**

Procedure: In a 25-mL round bottom flask aldehyde **26** (44 mg, 95% purity, 0.09 mmol) was dissolved in 3 mL dry methanol and $MgSO_4$ (0.2 g) was added. To the suspension was added azetidine **6** (11 mg, 0.11 mmol, 1.2 eq.) as a solid, followed by acetic acid (0.01 mL, 0.17 mmol, 2 eq.). After 10 minutes stirring at 0 °C, $NaCNBH_3$ (9 mg, 0.14 mmol, 1.5 eq.) was added in one portion and the reaction was allowed to warm to rt. After 2 h the reaction was worked up.

Workup: The reaction mixture was filtered over Celite and evaporated to dryness at 30 °C in a water bath.

Purification: The crude material was directly purified on the preparative HPLC using 80-98% methanol and 0.1% formic acid, to give fractions which were lyophilized to give 42 mg of product **30** (81%, 0.07 mmol).

$[\alpha]_D^{20}$ = -52.8 (c 0.8, $CHCl_3$). ^{1}H NMR (600 MHz, $CDCl_3$) δ 4.65 (m, 1H, H-2''), 4.23 (m, 1H, H-4'), 3.84 (m, 1H, H-2'), 3.82 – 3.74 (m, 1H, H-4'b), 3.64 (m, 1H, H-2), 3.55 (m, 1H, H-4a), 3.37 (m, 1H, H-4''a), 3.30 (m, 1H, H-4''b), 2.96 (m, 1H, H-4b), 2.72 (m, 2H, H-3''), 2.35 (m, 1H, H-3'a), 2.04 (m, 1H, H-3'b), 1.92 – 1.84 (m, 2H, H-3), 1.45 (s, 18H, COO*t*Bu), 1.42 (s, 9H, NBoc), 1.17 (s, 9H, O*t*Bu). ^{13}C NMR (151 MHz, $CDCl_3$) δ 173.7, 170.7, 170.2, 155.2, 82.2, 81.0, 80.8, 74.8, 70.6, 66.6, 59.8, 52.9, 50.5, 46.5, 33.3, 28.5 (3C), 28.1 (6C), 28.0 (3C), 25.5, 21.3. HR-MS (ESI): *m/z:* calc. for $C_{29}H_{53}N_2O_9$ $[M+H]^+$: 573.3746, found: m/z = 573.3754.

28. (2*S*,3*S*)-3-(*tert*-Butoxy)-1-((*S*)-4-(*tert*-butoxy)-3-((*tert*-butoxycarbonyl)((*S*)-3,4-di-*tert*-butoxy-4-oxobutyl)amino)-4-oxobutyl)azetidine-2-carboxylic acid (28)

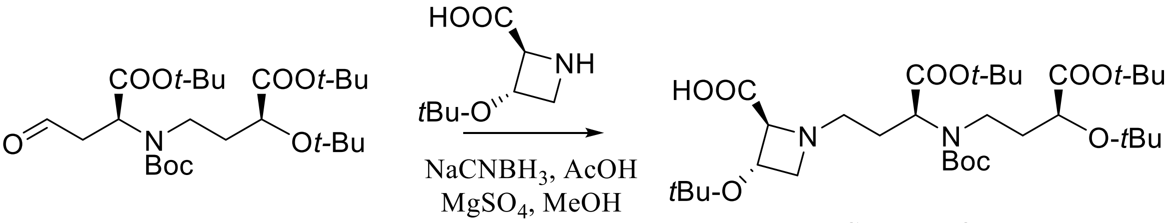

$C_{25}H_{45}NO_8$ **26**
487.634

$C_{33}H_{60}N_2O_{10}$ **28**
644.847

Procedure: In a 25-mL round bottom flask aldehyde **26** (76 mg, 95% purity, 0.16 mmol) was dissolved in 3 mL dry methanol and $MgSO_4$ (0.2 g) was added. To the suspension was added azetidine **5** (36 mg, 0.3 mmol, 1.3 eq.) as a solid, followed by acetic acid (0.013 mL, 0.231 mmol, 1.4 eq.). After 10 minutes stirring at 0 °C, $NaCNBH_3$ (15 mg, 0.23 mmol, 1.4 eq.) was added in one portion and the reaction was allowed to warm to rt. After 3.5 h the reaction was worked up.

Workup: Water (0.2 mL) was added to quench the reaction and the resulting mixture was filtered over Celite and evaporated to dryness at 30 °C in a water bath.

Purification: The crude material was directly purified on the preparative HPLC using 80-98% methanol and 0.1% formic acid, to give fractions which were lyophilized to give 67 mg of product **28** (65%, 0.1 mmol) as a colorless oil.

$[\alpha]_D^{20}$ = -38.8 (c 1.34, CH_2Cl_2). Mixture of rotamers ~2:1, ^{13}C-signals are doubled, ^{1}H overlap to multiplets: ^{1}H NMR (600 MHz, CD_3OD) δ 4.49 (ddd, *J* = 6.3 Hz, 1H, H-3''), 4.42 (d, *J* = 6.0 Hz, 1H, H-2''), 4.32 (dd, *J* = 10.5, 6.7 Hz, 1H, H-4''a), 4.00 – 3.94 (m, 1H, H-2'), 3.93 – 3.89 (m, 1H, H-2), 3.68 – 3.56 (m, 1H, H-4''b), 3.60 – 3.49 (m, 1H, H-4a), 3.39 (ddd, *J* = 11.8, 10.1, 5.9 Hz, 1H, H-4'a), 3.26 – 3.20 (m, 1H, H-4'b), 3.11 – 3.00 (m, 1H, H-4b), 2.32 (s, 1H, H-3'a), 2.06 (s, 1H, H-3'b), 2.00 (d, *J* = 13.2 Hz, 1H, H-3a), 1.98 – 1.79 (m, 1H, H-3b), 1.48 (s, 18H, COO*t*Bu), 1.45 (s, 9H, Boc), 1.24 (s, 9H, O*t*Bu), 1.19 (s, 9H, O*t*Bu). ^{13}C NMR (151 MHz, CD_3OD) δ 174.41, 174.40, 169.8, 169.7, 169.19, 169.15, 155.0, 154.7, 81.9, 81.6, 81.1, 80.4, 76.2, 75.5, 74.8, 74.6, 70.0, 69.8, 64.5, 60.0, 59.9, 59.4, 59.1, 53.4, 53.2, 46.4, 33.0, 32.2, 27.4, 27.3, 27.0, 26.94, 26.89, 26.84, 26.82, 25.1. HR-MS (ESI): *m/z* calc. for $C_{33}H_{61}N_2O_{10}$ $[M+H]^+$: 645.4321, found: 645.4344.

29. (2*S*,3*R*)-3-(*tert*-Butoxy)-1-((*S*)-4-(*tert*-butoxy)-3-((*tert*-butoxycarbonyl)((*S*)-3,4-di-*tert*-butoxy-4-oxobutyl)amino)-4-oxobutyl)azetidine-2-carboxylic acid (29)

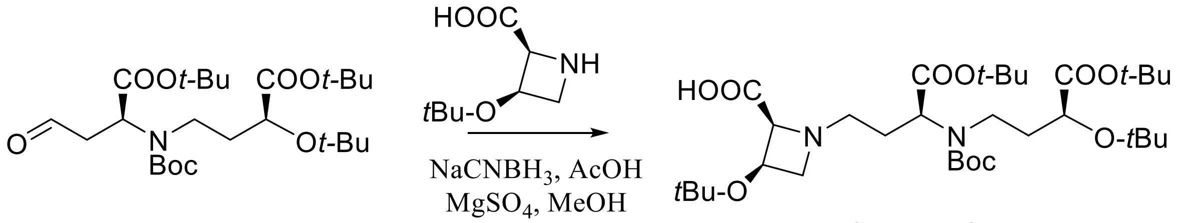

$C_{25}H_{45}NO_8$ **26**
487.634

$C_{33}H_{60}N_2O_{10}$ **29**
644.847

Procedure: In a 25-mL round bottom flask starting material **26** (36 mg, 95% purity, 0.07 mmol, 1 eq.) was dissolved in dry methanol (2 mL) and $MgSO_4$ (0.5 g) was added. To the suspension was added azetidine **4** (12 mg, 0.09 mmol, 1.2 eq.) as a solid at -10 °C, followed by acetic acid (0.008 mL, 0.140 mmol, 1.5 eq.). After 5 minutes stirring at -10 °C $NaCNBH_3$ (7.1 mg, 0.11 mmol, 1.5 eq.) was added in one portion and the reaction was allowed to warm to rt. After 3 h the reaction was worked up.

Workup: The reaction mixture was filtered over Celite and evaporated to dryness at 30 °C in a water bath.

Purification: The crude material was dissolved in MeOH/water = 1:1 and directly purified on the preparative HPLC using 80-98% methanol and 0.1% formic acid, to give fractions which were lyophilized to give 45 mg of product **29** (95%, 0.07 mmol) as a colorless oil.

$[\alpha]_D^{20}$ = -70.1 (c 0.7, CH_2Cl_2), Mixture of rotamers: ^{1}H NMR (600 MHz, CD_3OD) δ 4.76 (ddd, *J* = 6.7, 6.1, 3.2 Hz, 1H, H-2''), 4.72 (d, *J* = 7.4 Hz, 1H, H-3''), 4.09 (dd, *J* = 11.2, 6.1 Hz, 1H, H-4''a), 3.93 (m, 3H, H-4''b/H-2'/H-2), 3.59 (d, *J* = 14.2 Hz, 1H, H-4a), 3.31 (dt, *J* = 3.3, 1.6 Hz, 1H, H-4'a), 3.22 – 3.14 (m, 1H, H-4'b), 3.04 (ddd, *J* = 13.8, 11.2, 4.8 Hz, 1H, H-4b), 2.39 – 2.21 (m, 1H, H-3'a), 2.00 (d, *J* = 12.0 Hz, 2H, H-3'b/H-3a), 1.89 – 1.83 (m, 1H, H-3b), 1.48 (s, 18H, COO*t*Bu), 1.45 (s, 9H, Boc), 1.19 (18H, 2× O*t*Bu). ^{13}C NMR (151 MHz, CD_3OD) δ 175.8, 171.2, 171.1, 168.5, 156.5, 156.1, 83.3, 83.0, 82.5, 81.9, 76.8, 76.2, 76.0, 74.9, 71.4, 71.2, 64.1, 62.2, 62.1, 60.8, 60.6, 53.3, 53.1, 49.4, 49.3, 49.1, 49.0, 48.9, 48.7, 48.6, 47.6, 34.3, 33.6, 28.8, 28.7, 28.4, 28.3, 28.23, 28.20, 26.8. HR-MS (ESI): *m/z* calc. for $C_{33}H_{61}N_2O_{10}$ $[M+H]^+$: 645.4321, found: 645.4330.

30. *tert*-butyl (*S*)-2-(*tert*-Butoxy)-4-(((*S*)-1-(*tert*-butoxy)-1-oxo-4-(((*S*)-2-oxotetra-hydrofuran-3-yl)amino)butan-2-yl)(*tert*-butoxycarbonyl) amino)butanoate (45)

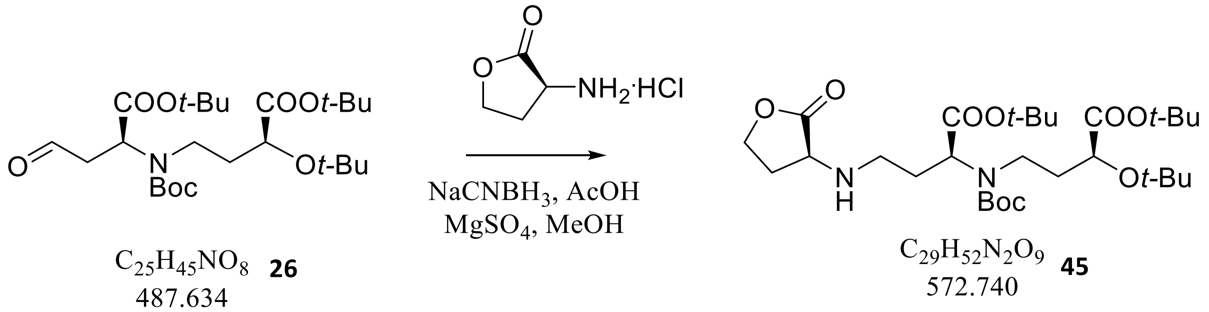

$C_{25}H_{45}NO_8$ **26**
487.634

$C_{29}H_{52}N_2O_9$ **45**
572.740

Procedure: In a Schlenk flask aldehyde **26** (90 mg, 95%, 0.18 mmol, 1eq.) was dissolved in dry methanol (5 mL) and $MgSO_4$ (0.2 g) was added. To the suspension was added lactone **7** (30 mg, 0.21 mmol, 1.2 eq.) as a solid in one portion, followed by the addition of acetic acid (10 µl, 0.17 mmol, 1 eq.) at 0 °C. After 10 minutes stirring at 0 °C $NaCNBH_3$ (15 mg, 0.24 mmol, 1.4 eq.) was added and the reaction was allowed to warm to room temperature and stirred for 1 hour.

Workup: The reaction mixture was quenched with distilled H_2O (0.1 mL) and subsequently filtered and evaporated to dryness at 30 °C in a water bath.

Purification: The residue was purified on a 10 g silica gel column using LP/EtOAc = 1.5:1 → 1:1.5 as eluent to give 70 mg of the title compound **45** (67%, 0.12 mmol).

$[\alpha]_D^{20}$ = -36.8 (c 0.6, CH_2Cl_2). ^{1}H NMR (600 MHz, $CDCl_3$) δ 4.42 – 4.33 (m, 1H, H-4''a), 4.26 – 4.21 (m, 1H, H-2'), 4.17 (ddd, *J* = 9.6, 6.4 Hz, 1H, H-4''b), 3.84 (dddd, *J* = 7.0, 2.6, 1.0 Hz, 1H, H2-2), 3.80 (dt, *J* = 7.9, 4.3 Hz, 1H, H2-1), 3.53 (dd, *J* = 9.2 Hz, 1H, H-2''), 3.47 – 3.34 (m, 1H, H-4a), 3.00 (ddd, *J* = 17.7, 9.5, 3.9 Hz, 1H, H-4b), 2.80 (dd, *J* = 12.5, 6.7 Hz, 1H, H-4'a), 2.70 (dd, *J* = 12.3, 6.1 Hz, 1H, H-4'b), 2.49 (dddd, *J* = 12.5, 8.4, 6.3, 2.2 Hz, 1H, H-3'a), 2.20 – 2.14 (m, 1H, H-3''), 2.08 (m, 1H, H-3'b), 1.91 – 1.82 (m, 1H, H-3), 1.43 (s, 27H, COO*t*Bu), 1.16 (s, 9H, O*t*Bu). ^{13}C NMR (151 MHz, $CDCl_3$) δ 177.2, 173.8, 173., 170.80, 170.7, 155.5, 155.0, 81.4, 80.9, 80.5, 80.2, 74.8, 74.7, 70.4, 70.3, 65.8, 59.1, 58.6, 56.6, 56.5, 45.1, 44.8, 44.6, 33.8, 32.8, 31.21, 30.6, 30.1, 28.5, 28.1, 28.05, 27.98. HR-MS (ESI): *m/z* calc. for $NaC_{29}H_{59}N_2O_9$ $[M+H]^+$: 595.3565, found: 595.3566.

Synthesis of final compounds **I-VIII**

31. Mugineic acid (VIII)

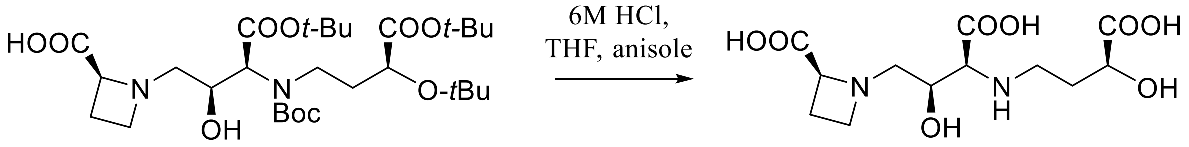

$C_{29}H_{52}N_2O_{10}$ 588.739 **33**

$C_{12}H_{20}N_2O_8$ 320.298 **VIII**

Procedure: To a solution of starting material **33** (0.10 g, 0.17 mmol, 1 eq.) in THF (2 mL) was added anisole (0.1 mL, 0.8 mmol, 5 eq.) followed by 6M HCl (2.50 mL, 15.3 mmol, 90 eq.) which was pre-chilled to 0 °C. The reaction mixture was stirred in an ice bath at first and allowed to reach room temperature over 1 hour. Reaction was stirred for 24 h then workup was executed.

Workup: The reaction was diluted with water (5 mL) and extracted with Et_2O (5 mL). The aqueous phase was separated and evaporated to remove THF and then lyophilized to give 66 mg of crude product.

Purification: The crude material was purified over 0.7 mL Dowex® 50WX8 200-400 resin using water → 1M ammonia as eluent giving product fractions that were evaporated to provide 54 mg of mugineic acid **VIII** (99%, 0.17 mmol) as a colorless glass.

To further increase purity, 50 mg of **VIII** were stirred with 1 mL dry ethanol for 3 hours and the solids (39 mg) collected by filtration. This step was repeated using 0.7 mL methanol to provide highly pure **VIII** (25 mg).

$[\alpha]_D^{20}$ = -69.8 (c 0.29, H_2O), Lit: -63.5 (c 0.31, water) [3], -64.6 (c 0.43, water) [8]. ^{1}H NMR (600 MHz, D_2O, pH = 6) δ 4.95 (t, *J* = 9.6 Hz, 1H, H-2''), 4.47 (dt, *J* = 9.8, 2.9 Hz, 1H, H-3'), 4.42 (dd, *J* = 8.1, 4.1 Hz, 1H, H-2), 4.11 (dd, *J* = 9.7, 4.0 Hz, 1H, H-4'a), 4.05 (app q, *J* = 9.6 Hz, 1H, H-4''a), 3.92 (d, *J* = 3.1 Hz, 1H, H-2'), 3.57 (dd, *J* = 13.4, 9.4 Hz, 1H, H-4''b), 3.45 (dd, *J* = 13.2, 2.6 Hz, 1H, H-4'b), 3.35 (ddd, *J* = 12.6, 8.3, 6.1 Hz, 1H, H-4a), 3.26 (ddd, *J* = 12.5, 8.5, 6.0 Hz, 1H, H-4b), 2.76 – 2.69 (m, 1H, H-3''a), 2.66 – 2.56 (m, 1H, H-3''b), 2.30 (m, 1H, H-3a), 2.12 (m, 1H, H-3b). ^{13}C NMR (151 MHz, D_2O) δ 176.8, 172.6, 168.8, 68.4 (C-2), 67.5 (C-2''), 64.5 (C-3'), 64.5 (C-2'), 55.9 (C-4''), 51.1 (C-4'), 44.7 (C-4), 29.4 (C-3), 21.7 (C-3''). HR-MS (ESI): *m/z* calc. for $C_{12}H_{21}N_2O_8$ $[M+H]^+$: 321.1293, found: 321.1296.

32. 3''-*epi*-Hydroxy mugineic acid (VI)

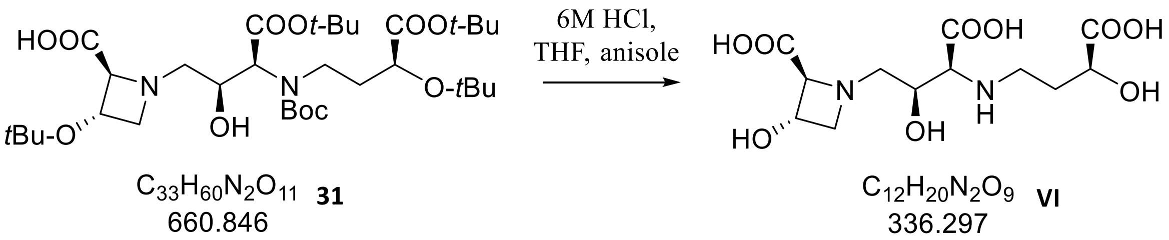

Procedure: To a solution of starting material **31** (58 mg, 0.09 mmol) in THF (1.5 mL) was added anisole (0.05 mL, 0.44 mmol, 5 eq.) followed by 6M HCl (1.5 mL, 7.9 mmol, 90 eq.) which was pre-chilled to 0 °C. The reaction mixture was stirred in an ice bath at first and allowed to reach room temperature over 1 hour. Reaction was stirred for 24 h then workup was executed.

Workup: Reaction was diluted with water (5 mL) and extracted with Et_2O (5 mL). The aqueous phase was separated and evaporated to remove THF and then lyophilized to give 47 mg of crude product.

Purification: The crude material was purified over 0.7 mL Dowex® 50WX8 200-400 resin using water → 1M ammonia as eluent giving product fractions that were evaporated to provide 30 mg of 3"-epi-OH-mugineic acid **VI** (>99%, 0.09 mmol) as a white solid.

m.p.: 188 °C (decomp.). $[\alpha]_D^{20}$ = -30.3 (c 1.10, H_2O), Lit: -33.2 (c 1.02, H_2O) [11]. ^{1}H NMR (600 MHz, D_2O, pH = 6) δ 4.91 (d, *J* = 6.9 Hz, 1H, H-2"), 4.74 (app q, *J* = 7.2 Hz, 1H, H-3"), 4.52 (dt, *J* = 9.4, 2.9 Hz, 1H, H-3'), 4.48 – 4.38 (m, 2H, H-4"), 4.07 (d, *J* = 3.0 Hz, 1H, H-2'), 3.94 (dd, *J* = 10.6, 7.3 Hz, 1H, H-2), 3.73 – 3.65 (m, 1H, H-4'a), 3.60 (dd, *J* = 13.8, 3.0 Hz, 1H, H-4'b), 3.37 (ddd, *J* = 12.6, 8.6, 6.1 Hz, 1H, H-4a), 3.29 (ddd, *J* = 12.5, 8.8, 6.0 Hz, 1H, H-4b), 2.38 – 2.22 (m, 1H, H-3a), 2.13 (dtd, *J* = 14.4, 8.5, 5.9 Hz, 1H, H-3b). ^{13}C NMR (151 MHz, D_2O) δ 179.9 (C-1), 171.0 (C-1"), 169.2 (C-1'), 77.1 (C-2"), 70.4 (C-2), 64.8 (C-3'), 64.6 (C-2'), 64.2 (C-3"), 59.6 (C-4"), 57.1 (C-4'), 45.1 (C-4), 30.0 (C-3). HR-MS (ESI): *m/z* calc. for $C_{12}H_{21}N_2O_9$ $[M+H]^+$: 337.1242, found: 337.1250.

33. 3''-Hydroxy mugineic acid (VII)

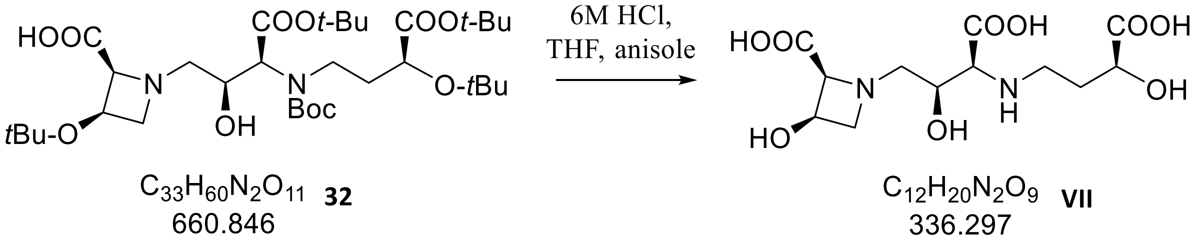

Procedure: To a solution of starting material **32** (46 mg, 0.07 mmol, 1 eq.) in THF (1.5 mL) was added anisole (0.04 mL, 0.35 mmol, 5 eq.) followed by 6M HCl (1.4 mL, 8.4 mmol, 120 eq.) which was pre-chilled to 0 °C. The reaction mixture was stirred in an ice bath at first and allowed to reach room temperature over 1 hour. Reaction was stirred for 24 h then workup was executed.

Workup: Reaction was diluted with water (5 mL) and extracted with Et_2O (5 mL). The aqueous phase was separated and evaporated to remove THF and then lyophilized to give 50 mg of crude product.

Purification: The crude material was purified over 0.7 mL Dowex® 50WX8 200-400 resin using water → 1M ammonia as eluent giving product fractions that were evaporated to provide 23 mg of 3"-hydroxy mugineic acid **VI** (96%, 0.07 mmol) as a colorless glass.

$[\alpha]_D^{20}$ = -35.4 (c 0.37, H_2O). ^{1}H NMR (600 MHz, D_2O, pH = 5) δ 4.92 (d, *J* = 6.7 Hz, 1H, H-2'), 4.82 (dt, *J* = 7.6, 5.1 Hz, 1H, H-3'), 4.43 (dt, *J* = 8.6, 3.1 Hz, 1H, H-3"), 4.25 (dd, *J* = 11.5, 6.4 Hz, 1H, H-4'a), 4.16 (dd, *J* = 7.3, 4.5 Hz, 1H, H-2), 4.02 (dd, *J* = 11.4, 2.5 Hz, 1H, H-4'b), 3.83 (d, *J* = 3.1 Hz, 1H, H-2"), 3.47 (dd, *J* = 13.5, 8.5 Hz, 1H, H-4"a), 3.41 (dd, *J* = 13.5, 2.9 Hz, 1H, H-4"b), 3.29 (ddd, *J* = 12.5, 8.0, 6.4 Hz, 1H, H-4a), 3.18 (ddd, *J* = 12.6, 8.0, 6.3 Hz, 1H, H-4b), 2.18 (dddd, *J* = 14.5, 8.0, 6.5, 4.5 Hz, 1H, H-3a), 2.03 (dddd, *J* = 14.3, 7.0 Hz, 1H, H-3b). ^{13}C NMR (151 MHz, D_2O) δ 181.0 (C-1), 170.7 (C-1'), 170.5 (C-1"), 73.8 (C-2'), 69.9 (C-2), 64.8 (C-3"), 64.3 (C-2"), 62.7 (C-3'), 59.9 (C-4'), 55.2 (C-4"), 44.2 (C-4), 29.0 (C-3). HR-MS (ESI): *m/z* calc. for $C_{12}H_{21}N_2O_9$ $[M+H]^+$: 337.1242, found: 337.1240.

34. (2*S*,3*S*)-2-(((*S*)-3-Carboxy-3-hydroxypropyl)amino)-3-hydroxy-4-(((*S*)-2-oxotetrahydrofuran-3-yl)amino)butanoic acid (46)

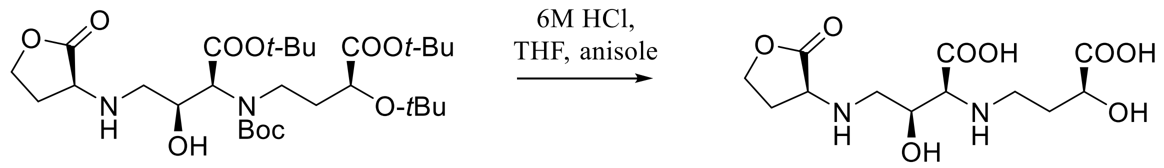

$C_{29}H_{52}N_2O_{10}$ **44**
588.739

$C_{12}H_{20}N_2O_8$ **46**
320.298

Procedure: To a solution of starting material **44** (46 mg, 0.08 mmol) in 1 mL THF was added anisole (0.04 mL, 0.39 mmol, 5 eq.) followed by 6M HCl (1.7 mL, 9.4 mmol, 120 eq.) which was pre-chilled to 0 °C. The reaction mixture was stirred in an ice bath at first and allowed to reach room temperature over 1 hour. Reaction was stirred for 24 h then workup was executed.

Workup: Reaction was diluted with water (5 mL) and extracted with 5 mL Et_2O. The aqueous phase was separated and evaporated to remove THF and then lyophilized to give 31 mg (>99%, 0.08 mmol) of crude product **46**. The crude product was used immediately and without further purification.

$[\alpha]_D^{20}$ = +2.2 (c 0.85, H_2O), ^1H NMR (600 MHz, D_2O, pH = 5) δ 4.41 – 4.36 (m, 1H, H-3'), 4.22 (d, *J* = 5.4 Hz, 1H, H-2'), 4.03 (dd, *J* = 8.3, 3.7 Hz, 1H, H-2), 3.65 (td, *J* = 6.5, 3.2 Hz, 2H, H-4''), 3.57 (ddd, *J* = 13.5, 10.0, 5.9 Hz, 1H, H-4a), 3.44 – 3.40 (m, 1H, H-4b), 3.39 (t, *J* = 6.7 Hz, 1H, H-2''), 3.10 (dd, *J* = 14.7, 3.3 Hz, 2H, H-4'a), 3.01 (dd, *J* = 14.9, 2.1 Hz, 2H, H-4'b), 2.04 (dddd, *J* = 12.9, 6.4 Hz, 1H, H-3''a), 1.98 (ddt, *J* = 9.8, 5.9, 3.6 Hz, 1H, H-3a), 1.83 (dddd, *J* = 18.7, 9.4, 5.4, 5.4 Hz, 1H, H-3b), 1.69 (dddd, *J* = 13.7, 6.7, 6.7 Hz, 1H, H-3''b). ^{13}C NMR (151 MHz, D_2O) δ 181.0 (COOH), 176.2 (COOH), 174.6 (COOH), 70.6 (C-2), 68.7 (C-2'), 66.1 (C-3'), 59.2 (C-4''), 58.1 (C-2''), 51.8 (C-4'), 48.5 (C-4), 34.0 (C-3''), 31.7 (C-3). HR-MS (ESI): *m/z* calc. for $C_{12}H_{20}N_2O_8$ $[M+H]^+$: 321.1293, found: 321.1314.

35. Hydroxyavenic acid A (V)

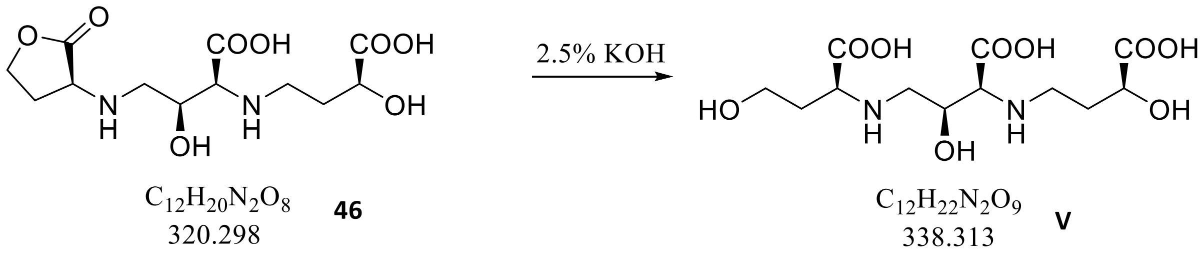

46

V

Procedure: To starting material **43** (17 mg, 0.05 mmol) was added dropwise at room temperature a freshly prepared aqueous solution of KOH (2.5% weight, 120 mg in 5 mL water, 2.12 mmol, 40 eq.) and the resulting solution was stirred over night (21 hours) at room temperature.

Workup and Purification: The reaction mixture was acidified with 2M HCl to pH = 2 and applied directly to a Dowex® 50WX8 column (4 g, washed neutral with water then eluted with NH_4OH). Pooled fractions were lyophilized to give 14 mg (78%, 0.48 mmol) of hydroxyavenic acid A (**V**) as a white foam.

m.p.: 233 – 235 °C (H_2O), $[\alpha]_D^{20}$ = -6.1 (c 0.45, H_2O). ^{1}H NMR (600 MHz, D_2O, pH = 5) δ 1H NMR (600 MHz, D2O) δ 4.50 (dt, *J* = 8.6, 3.4 Hz, 1H, H-3'), 4.16 (dd, *J* = 7.3, 4.6 Hz, 1H, H-2), 3.88 (d, *J* = 3.1 Hz, 1H, H-2'), 3.82 (t, *J* = 6.0 Hz, 1H, H-2''), 3.77 (t, *J* = 6.2 Hz, 2H, H-4''), 3.35 (dd, *J* = 13.0, 8.5 Hz, 1H, H-4a, H-4'a), 3.32 – 3.26 (m, 2H, H-4'b), 3.20 (ddd, *J* = 12.6, 8.1, 6.3 Hz, 1H, H-4b), 2.24 – 2.15 (m, 1H, H-3a), 2.15 – 2.07 (m, 1H, H-3''), 2.06 – 1.99 (m, 1H, H-3b).^{13}C NMR (151 MHz, D_2O) δ 179.9 (C-1), 173.2 (C-1''), 169.5 (C-1'), 70.4 (C-2), 64.7 (C-2'), 64.6 (C-3'), 61.1 (C-2''), 58.5 (C-4''), 48.4 (C-4'), 44.9 (C-4), 31.4 (C-3), 30.0 (C-3''). HR-MS (ESI): *m/z* calc. for $C_{12}H_{23}N_2O_9$ $[M+H]^+$: 339.1398, found: 339.1399.

36. Deoxymugineic acid (IV)

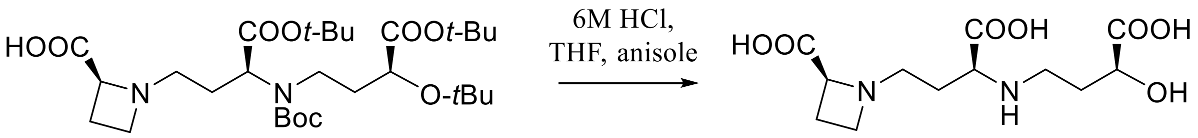

$C_{29}H_{52}N_2O_9$
572.740 **30**

$C_{12}H_{20}N_2O_7$
304.299 **IV**

Procedure: To a solution of starting material **30** (82 mg, 0.14 mmol) in 1.5 mL THF was added anisole (0.08 mL, 0.71 mmol, 5 eq.) followed by 6M HCl (2.4 mL, 13 mmol, 90 eq.) which was pre-chilled to 0 °C. The reaction mixture was stirred in an ice bath at first and allowed to reach room temperature over 1 hour. Reaction was stirred for 24 h then workup was executed.

Workup: Reaction was diluted with water (10 mL) and extracted twice with Et_2O (10 mL). The aqueous phase was separated and evaporated to remove THF and then lyophilized to give 59 mg of crude product.

Purification: The crude material was purified over 0.5 mL Dowex® 50WX8 200-400 resin using water → 1M ammonia as eluent giving product fractions that were lyophilized to provide 44 mg of 3'-deoxymugineic acid **III** (95%, 0.14 mmol) as a fluffy white solid.

$[\alpha]_D^{20}$ = -27.1 (c 0.6, MeOH). ^{1}H NMR (600 MHz, D_2O, pH = 6) δ 4.75 (t, *J* = 9.7 Hz, 1H, H-2''), 4.14 (dd, *J* = 7.2, 4.5 Hz, 1H, H-2), 4.09 (td, *J* = 9.8, 4.3 Hz, 1H, H-4a'), 3.96 (app q, *J* = 9.6 Hz, 1H, H-4b'), 3.78 (dd, *J* = 8.8, 4.3 Hz, 1H, H-2'), 3.46 (ddd, *J* = 13.1, 9.4, 6.1 Hz, 1H, H-4a''), 3.40 – 3.31 (m, 1H, H-4b''), 3.22 (dt, *J* = 14.7, 7.4 Hz, 1H, H-4a), 3.16 (ddd, *J* = 12.6, 8.2, 6.2 Hz, 1H, H-4b), 2.79 – 2.69 (m, 1H, H-3a''), 2.59 – 2.48 (m, 1H, H-3b''), 2.20 (dddd, *J* = 17.0, 12.6, 5.9, 5.9 Hz, 2H, H-3'), 2.16 – 2.08 (m, 2H, H-3), 2.01 (dt, *J* = 14.0, 7.0 Hz, 1H, H-3b). ^{13}C NMR (151 MHz, D_2O) δ 179.8 (C-1), 173.0 (C-1''), 171.9 (C-1'), 70.2 (C-2), 66.7 (C-2''), 59.4 (C-2'), 51.1 (C-4'), 50.3 (C-4''), 44.1 (C-4), 30.2 (C-3), 24.4 (C-3'), 20.9 (C-3''). HR-MS (ESI): *m/z* calc. for $C_{12}H_{21}N_2O_7$ $[M+H]^+$: 305.1344, found: 305.1344.

37. 3''-*epi*-Hydroxy-deoxymugineic acid (II)

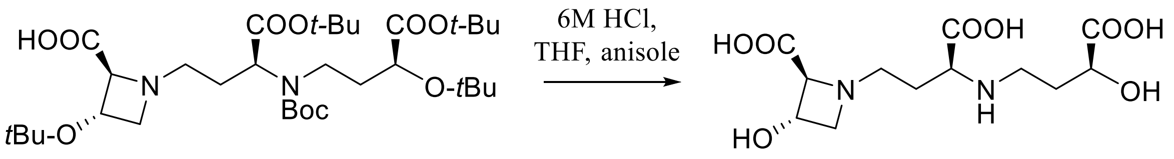

$C_{33}H_{60}N_2O_{10}$ **28**
644.847

$C_{12}H_{20}N_2O_8$ **II**
320.298

Procedure: To a solution of starting material **28** (27 mg, 0.04 mmol) in 1 mL THF was added anisole (0.02 mL, 0.20 mmol, 5 eq.) followed by 6M HCl (1.1 mL, 6.3 mmol, 150 eq.) which was pre-chilled to 0 °C. The reaction mixture was stirred in an ice bath at first and allowed to reach room temperature over 1 hour. Reaction was stirred for 24 h then workup was executed.

Workup: Reaction was diluted with water (5 mL) and extracted with Et_2O (5 mL). The aqueous phase was separated and evaporated to remove THF and then lyophilized to give 19 mg of crude product.

Purification: The crude material was purified over 0.4 mL Dowex® 50WX8 200-400 resin using water → 1M ammonia as eluent giving product fractions that were evaporated to provide 13 mg of 3''-epi-hydroxy-3'-deoxymugineic acid **II** (98%, 0.04 mmol) as a colorless oil.

$[\alpha]_D^{20}$ = -19.8 (c 0.44, H_2O). ^{1}H NMR (600 MHz, D_2O, pH = 6) δ 4.50 (app q, *J* = 6.8 Hz, 1H, H-3''), 4.28 (d, *J* = 6.4 Hz, 1H, H-2''), 4.19 (dd, *J* = 9.9, 7.0 Hz, 1H, H-4''a), 4.14 (dd, *J* = 7.3, 4.4 Hz, 1H, H-2), 3.74 (dd, *J* = 7.8, 5.1 Hz, 1H, H-2'), 3.57 – 3.47 (m, 1H, H-4''b), 3.32 (ddd, *J* = 14.0, 7.1, 7.1 Hz, 1H, H-4'a), 3.24 (dt, *J* = 11.1, 6.0 Hz, 1H, H-4'b), 3.21 – 3.11 (m, 2H, H-4), 2.15 (dddd, *J* = 17.1, 9.3, 7.0, 3.6 Hz, 2H, H-3), 2.09 (ddd, *J* = 14.3, 7.2, 7.2 Hz, 1H, H-3'a), 2.00 (dddd, *J* = 14.2, 7.8, 7.8 Hz, 1H, H-3'b). ^{13}C NMR (151 MHz, D_2O) δ 179.9 (COOH), 173.1 (COOH), 172.6 (COOH), 76.5 (C-2''), 70.2 (C-3''), 64.1 (C-2), 60.2 (C-2'), 59.1 (C-4''), 53.2 (C-4'), 44.1 (C-4), 30.4 (C-3'), 25.2 (C-3). HR-MS (ESI): *m/z* calc. for $C_{12}H_{21}N_2O_8$ $[M+H]^+$: 321.1293, found: 321.1304.

38. 3’’-Hydroxy-deoxymugineic acid (III)

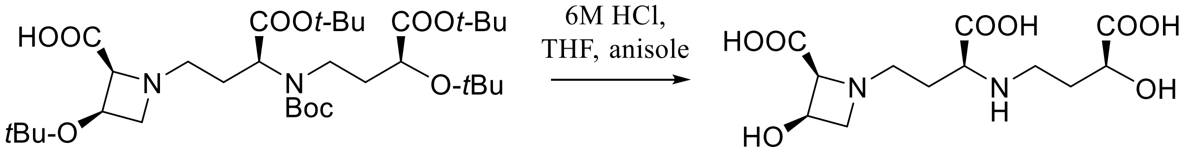

$C_{33}H_{60}N_2O_{10}$ **29**
644.847

$C_{12}H_{20}N_2O_8$ **III**
320.298

Procedure: To a solution of starting material **29** (23 mg, 0.04 mmol) in 0.5 mL THF was added anisole (0.02 mL, 0.20 mmol, 5 eq.) followed by 6M HCl (1.5 mL, 7.9 mmol, 150 eq.) which was pre-chilled to 0 °C. The reaction mixture was stirred in an ice bath at first and allowed to reach room temperature over 1 hour. Reaction was stirred for 24 h then workup was executed.

Workup: Reaction was diluted with water (5 mL) and extracted twice with Et_2O (5 mL). The aqueous phase was separated and evaporated to remove THF and then lyophilized to give 21 mg of crude product.

Purification: The crude material was purified over 0.5 mL Dowex® 50WX8 200-400 resin using water → 1M ammonia as eluent giving product fractions that were evaporated to provide 12 mg of 3''-hydroxy-3'-deoxymugineic acid **III** (>99%, 0.04 mmol) as a colorless oil.

$[\alpha]_D^{20}$ = -38.7 (c 0.42, H_2O), ^{1}H NMR (600 MHz, D_2O, pH = 6) δ 4.79 (m, 1H, H-2) 4.69 (d, *J* = 6.6 Hz, 1H, H-2''), 4.13 (dd, *J* = 7.3, 4.5 Hz, 1H, H-3''), 4.08 (dd, *J* = 11.1, 6.3 Hz, 1H, H-4a''), 3.92 – 3.87 (m, 1H, H-4b''), 3.71 (dd, *J* = 8.0, 5.0 Hz, 1H, H-2'), 3.37 – 3.29 (m, 1H, H-4a'), 3.29 – 3.22 (m, 1H, H-4b'), 3.20 – 3.14 (m, 1H, H-4a), 3.16 – 3.08 (m, 1H, H-4b), 2.20 – 2.09 (m, 2H, H-3a/H-3a'), 2.07 (dd, *J* = 14.4, 6.4 Hz, 1H, H-3b'), 2.00 (dddd, *J* = 14.2, 14.1, 6.3, 6.3 Hz, 1H, H-3b). ^{13}C NMR (151 MHz, D_2O) δ 179.9 (C-1), 172.8 (C-1'), 170.3 (C-1''), 73.2 (C-3''), 70.2 (C-2), 62.7 (C-2''), 60.1 (C-2'), 59.4 (C-4''), 51.6 (C-4'), 44.1 (C-4), 30.5 (C-3'), 25.2 (C-3). HR-MS (ESI): *m/z* calc. for $C_{12}H_{21}N_2O_8$ $[M+H]^+$: 321.1293, found: 321.1298.

39. (*S*)-4-(((*S*)-1-Carboxy-3-(((*S*)-2-oxotetrahydrofuran-3-yl)amino)propyl)amino)-2-hydroxybutanoic acid (47)

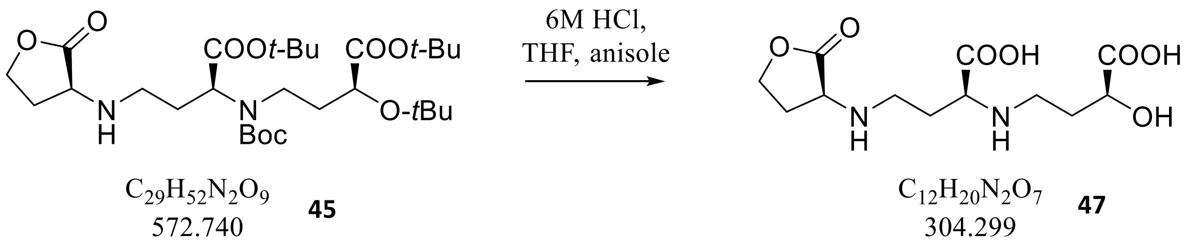

Procedure: To a solution of starting material **45** (61 mg, 0.11 mmol) in 1.1 mL THF was added anisole (0.06 mL, 0.53 mmol, 5 eq.) followed by 6M HCl (1.8 mL, 9.6 mmol, 90 eq.) which was pre-chilled to 0 °C. The reaction mixture was stirred in an ice bath at first and allowed to reach room temperature over 1 hour. Reaction was stirred for 24 h then workup was executed.

Workup: Reaction was diluted with water (10 mL) and extracted twice with Et_2O (10 mL). The aqueous phase was separated and evaporated to remove THF and then lyophilized to give 38 mg of product **47** (90% purity according to ^{1}H-NMR, 98% yield) as off-white frothy bubbles that solidified. No further purification was necessary.

$[\alpha]_D^{20}$ = -7.7 (c 1.13, H_2O), ^{1}H NMR (600 MHz, D_2O, pH = 6) δ 4.60 (t, *J* = 9.2 Hz, 1H, H-4''a), 4.51 (dd, *J* = 11.7, 8.9 Hz, 1H, H-2''), 4.43 – 4.37 (m, 2H, H-4''b, H2), 4.15 – 4.09 (m, 1H, H-2'), 3.57 (dt, *J* = 12.6, 8.4 Hz, 1H, H-4'a), 3.40 (dt, *J* = 12.6, 7.3 Hz, 1H, H-4'b), 3.29 (dddd, *J* = 27.8, 12.5, 8.7, 6.1 Hz, 2H, H-4), 2.85 – 2.76 (m, 1H, H-3''a), 2.45 (ddd, *J* = 11.9, 9.3 Hz, 1H, H-3''b), 2.38 (app q, *J* = 7.9 Hz, 2H, H-3'), 2.26 (dddd, *J* = 16.2, 14.5, 7.4, 5.0 Hz, 1H, H-3a), 2.08 (dddd, *J* = 14.4, 8.5, 5.9 Hz, 1H, H-3b). ^{13}C NMR (151 MHz, D_2O) δ 176.3 (COOH), 173.1 (COOH), 169.9 (COOH), 67.9 (C-4''), 67.3 (C-2), 57.5 (C-2'), 54.8 (C-2''), 43.6 (C-4), 43.0 (C-4'), 29.5 (C-3), 25.7 (C-3''), 25.3 (C-3'). HR-MS (ESI): *m/z* calc. for $C_{12}H_{21}N_2O_7$ $[M+H]^+$: 305.1344, found: 305.1343.

40. Avenic acid A (I)

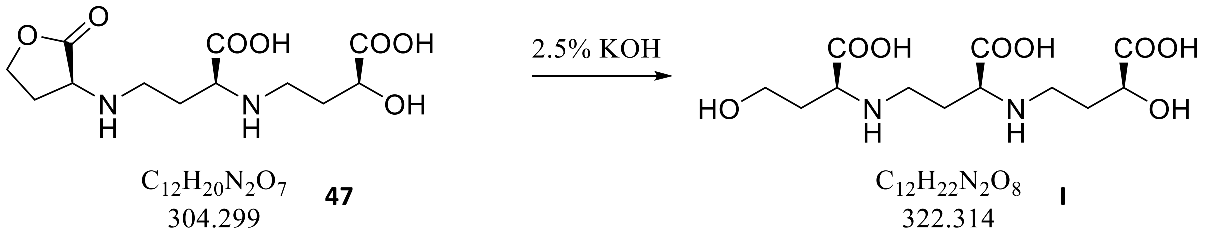

Procedure: To starting material **47** (30 mg, 0.99 mmol) was added dropwise at room temperature a freshly prepared aqueous solution of KOH (2.5% weight, 240 mg in 9.20 mL water, 3.94 mmol, 40 eq.) and the resulting solution was stirred over night (16 hours) at room temperature.

Workup: The reaction mixture was acidified with 2M HCl to pH = 2 and applied directly to a Dowex® 50WX8 column (0.5 mL, washed neutral with water then eluted with 1M NH_4OH). Pooled fractions were lyophilized to give 15 mg (49%, 0.48 mmol) of avenic acid A (**I**) as an off-white foam. No further purification was necessary.

m.p.: >300 °C [12], $[\alpha]_D^{20}$ = +14.1 (c 0.2, H_2O). ^{1}H NMR (600 MHz, D_2O, pH = 4-5) δ 3.93 (dd, *J* = 8.4, 3.8 Hz, 1H, H-2), 3.53 (dd, *J* = 7.0 Hz, 2H, H-4''), 3.03 (app t, *J* = 6.8 Hz, 1H, H-2''), 2.96 (dd, *J* = 7.8, 5.8 Hz, 1H, H-2'), 2.59 – 2.51 (m, 1H, H-4a), 2.47 – 2.35 (m, 3H, H-4b, H-4'), 1.81 – 1.73 (m, 1H, H-3a), 1.73 – 1.64 (m, 4H, H-3b, H-3'', H-3'a), 1.57 (ddd, *J* = 10.6, 7.9, 5.5 Hz, 1H, H-3'b). ^{13}C NMR (151 MHz, D_2O) δ 179.5 (C-1''), 173.1 (C-1'), 172.1 (C-1), 70.0 (C-2), 60.8 (C-2'), 59.7 (C-2''), 58.2 (C-4''), 44.0 (C-4'), 43.8 (C-4), 31.5 (C-3), 30.2 (C-3''), 25.8 (C-3'). HR-MS (ESI): *m/z* calc. for $C_{12}H_{23}N_2O_8$ [*M*+H]$^+$: 323.1449, found: 323.1451.

Spectra

Figure 3: ^{1}H-NMR (400 MHz, $CDCl_3$) of (*S*)-2-(2,2-dimethyl-5-oxo-1,3-dioxolan-4-yl)acetic acid (**35**)

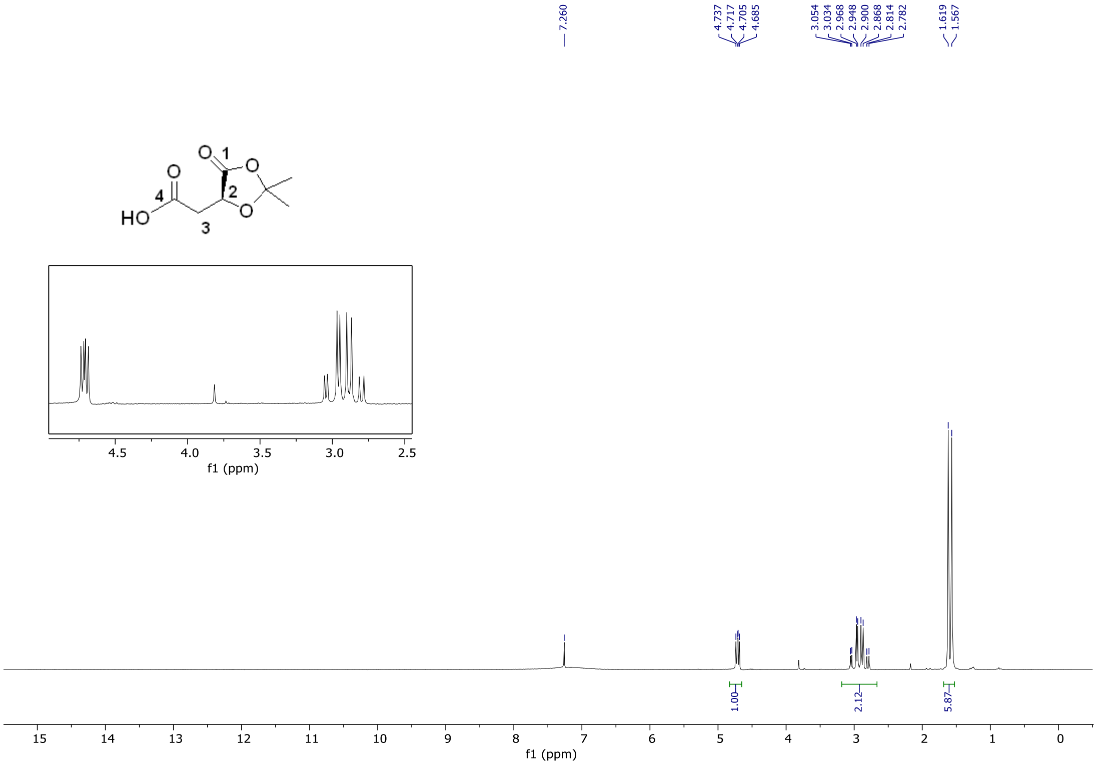

Figure 4: ^{13}C-NMR (50 MHz, $CDCl_3$) of (*S*)-2-(2,2-dimethyl-5-oxo-1,3-dioxolan-4-yl)acetic acid (**35**)

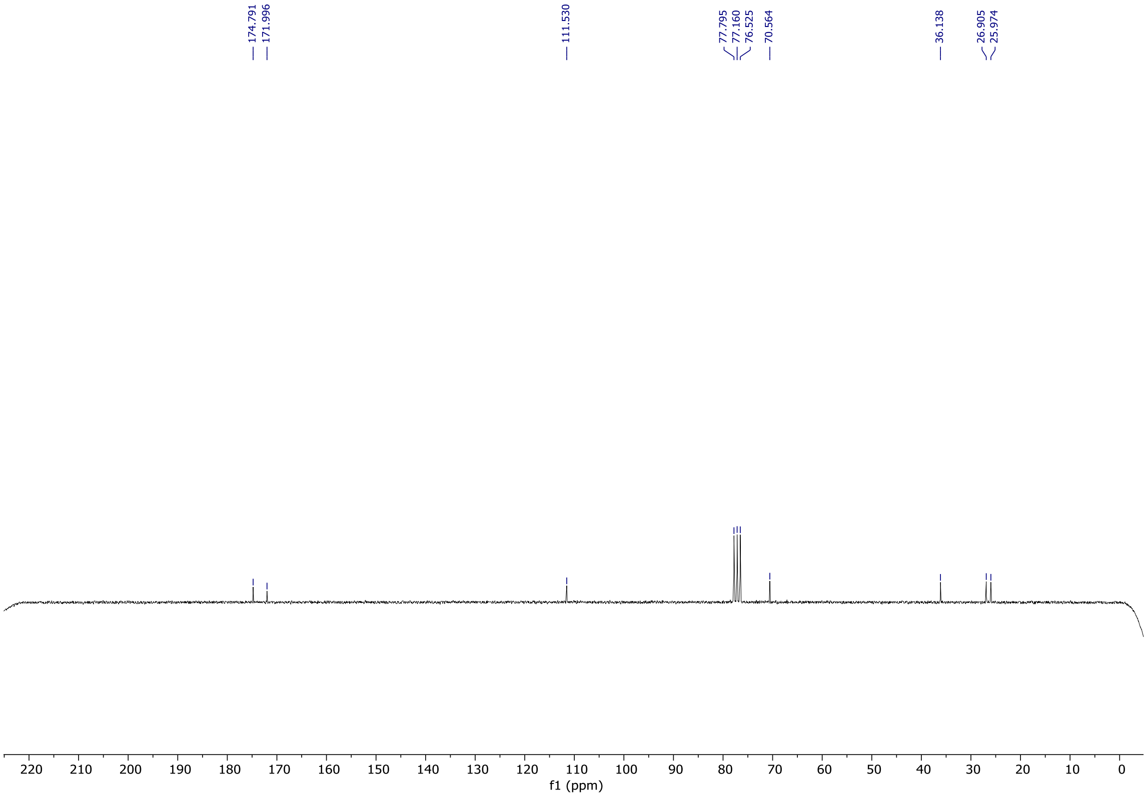

Figure 5: ^{1}H-NMR (400 MHz, $CDCl_3$) of ethyl (*S*)-2-(2,2-dimethyl-5-oxo-1,3-dioxolan-4-yl)acetate (**36**)

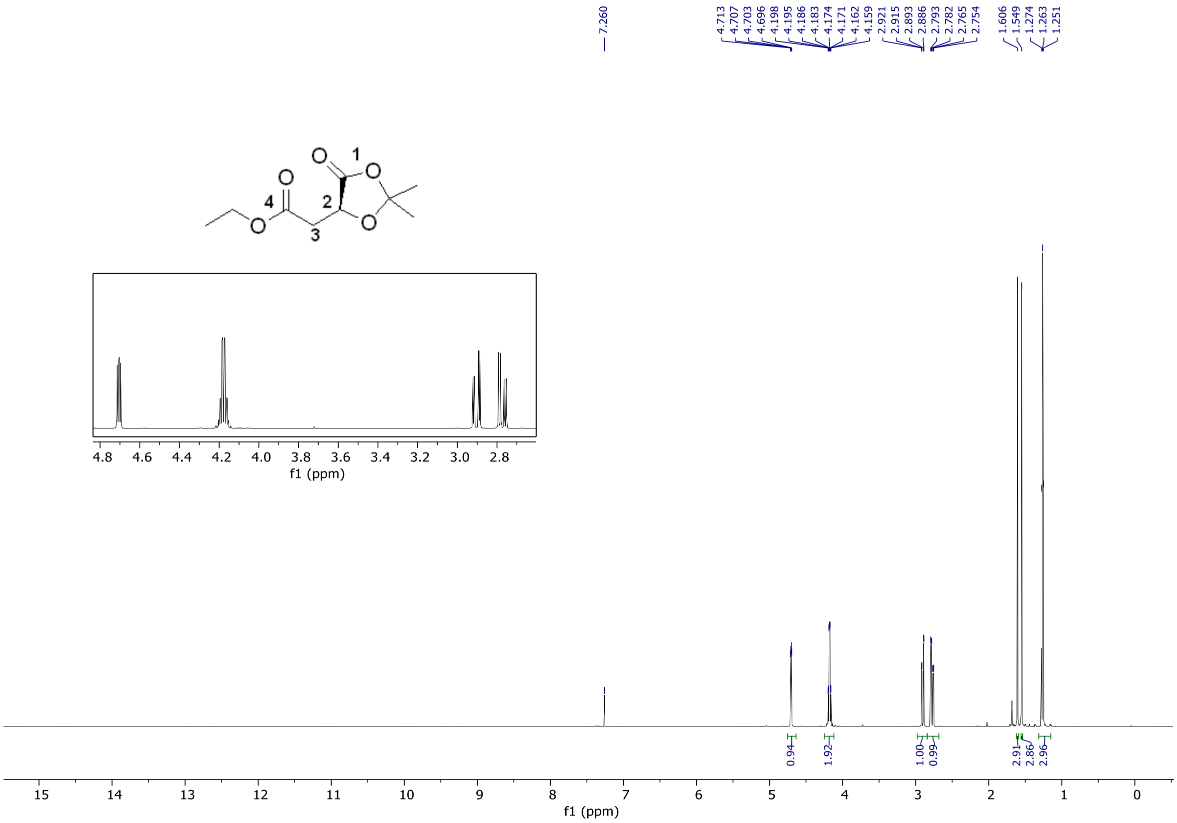

Figure 6: ^{13}C-NMR (101 MHz, $CDCl_3$) of ethyl (*S*)-2-(2,2-dimethyl-5-oxo-1,3-dioxolan-4-yl)acetate (**36**)

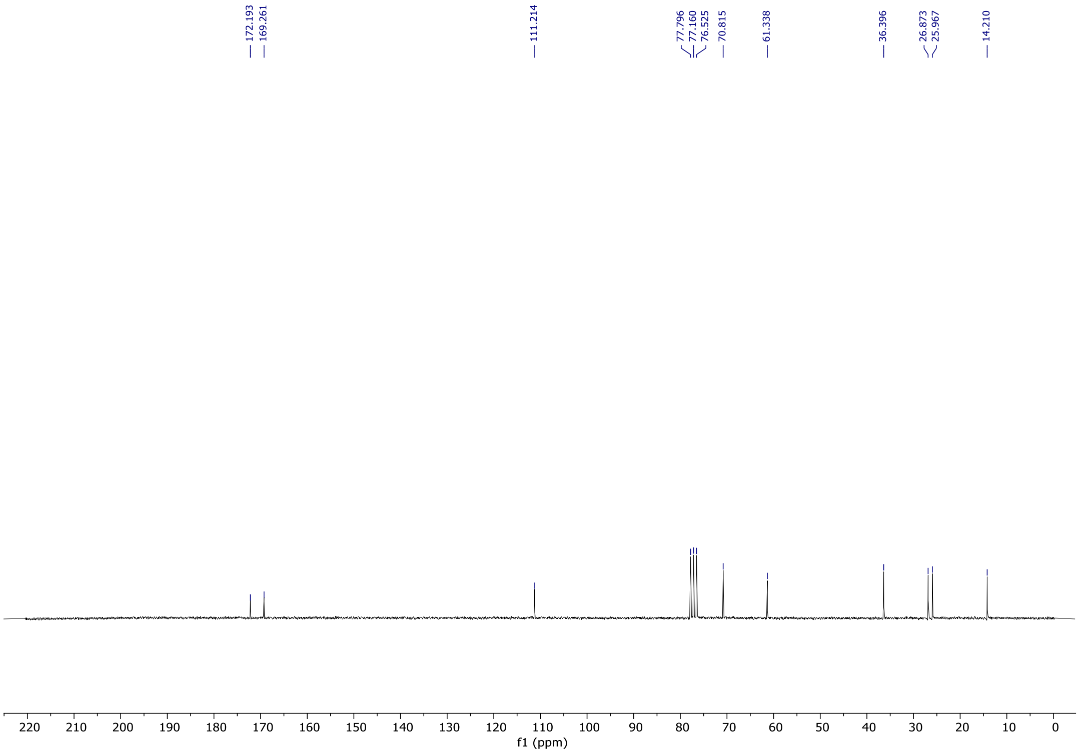

Figure 7: ^{1}H-NMR (600 MHz, $CDCl_3$) of (*S*)-4-ethoxy-2-hydroxy-4-oxobutanoic acid (**37**)

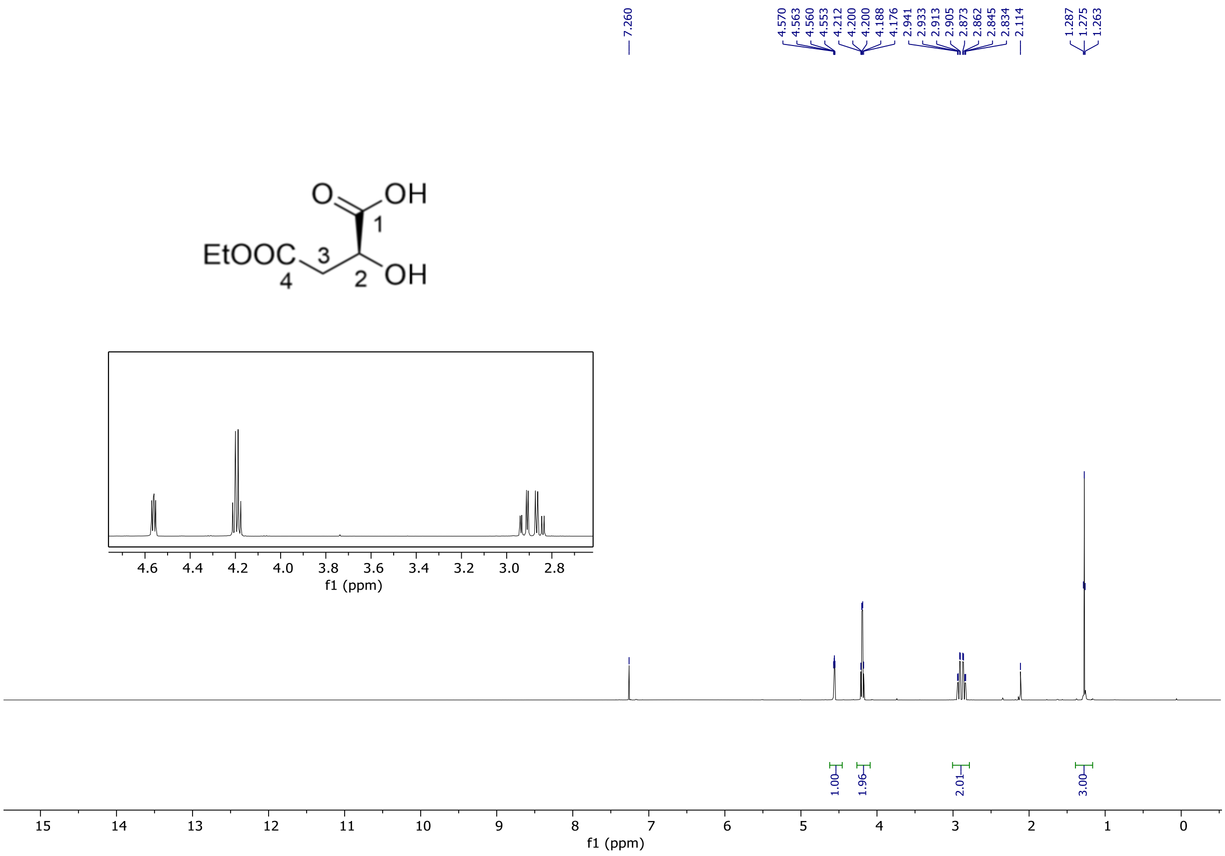

Figure 8: ^{13}C-DEPTQ (151 MHz, $CDCl_3$) of (*S*)-4-ethoxy-2-hydroxy-4-oxobutanoic acid (**37**)

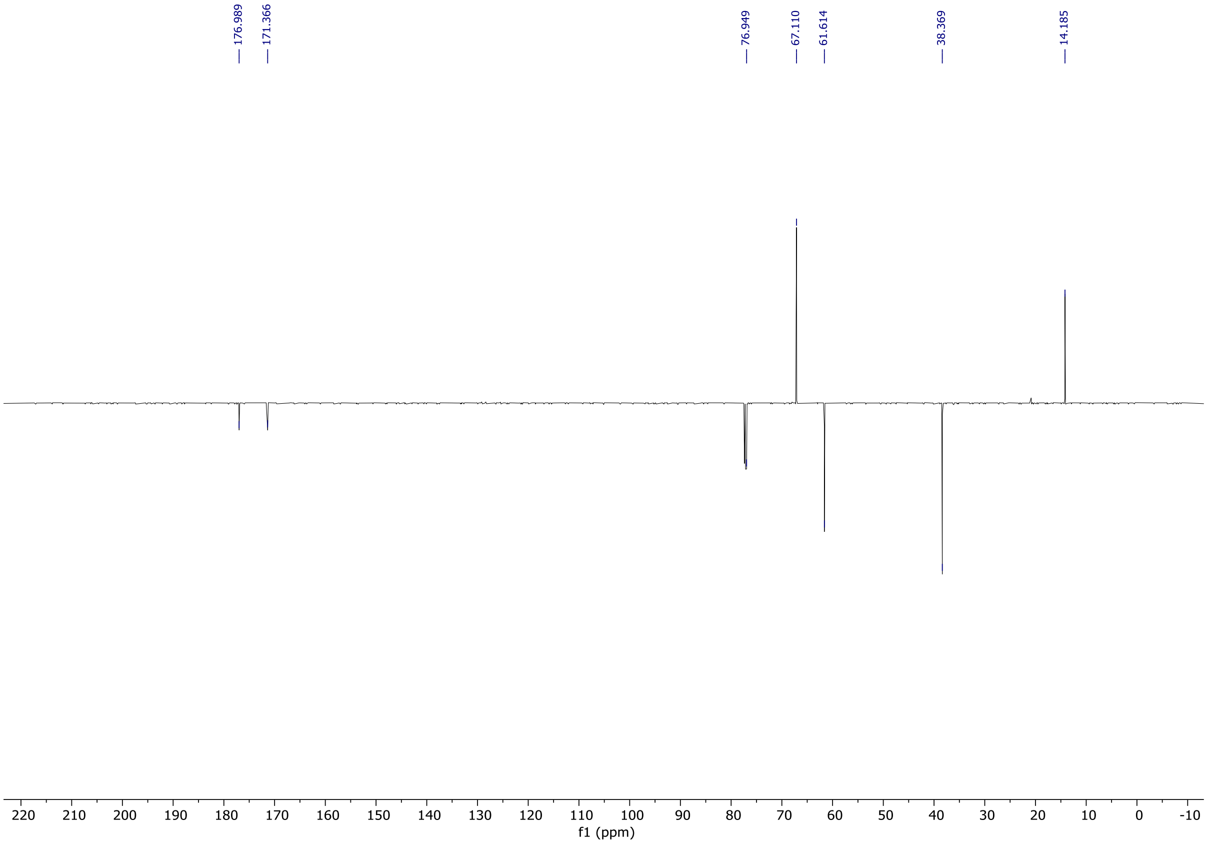

Figure 9: ^{1}H-NMR (600 MHz, $CDCl_3$) of 1-(*tert*-butyl) 4-ethyl (*S*)-2-(*tert*-butoxy)succinate (**38**)

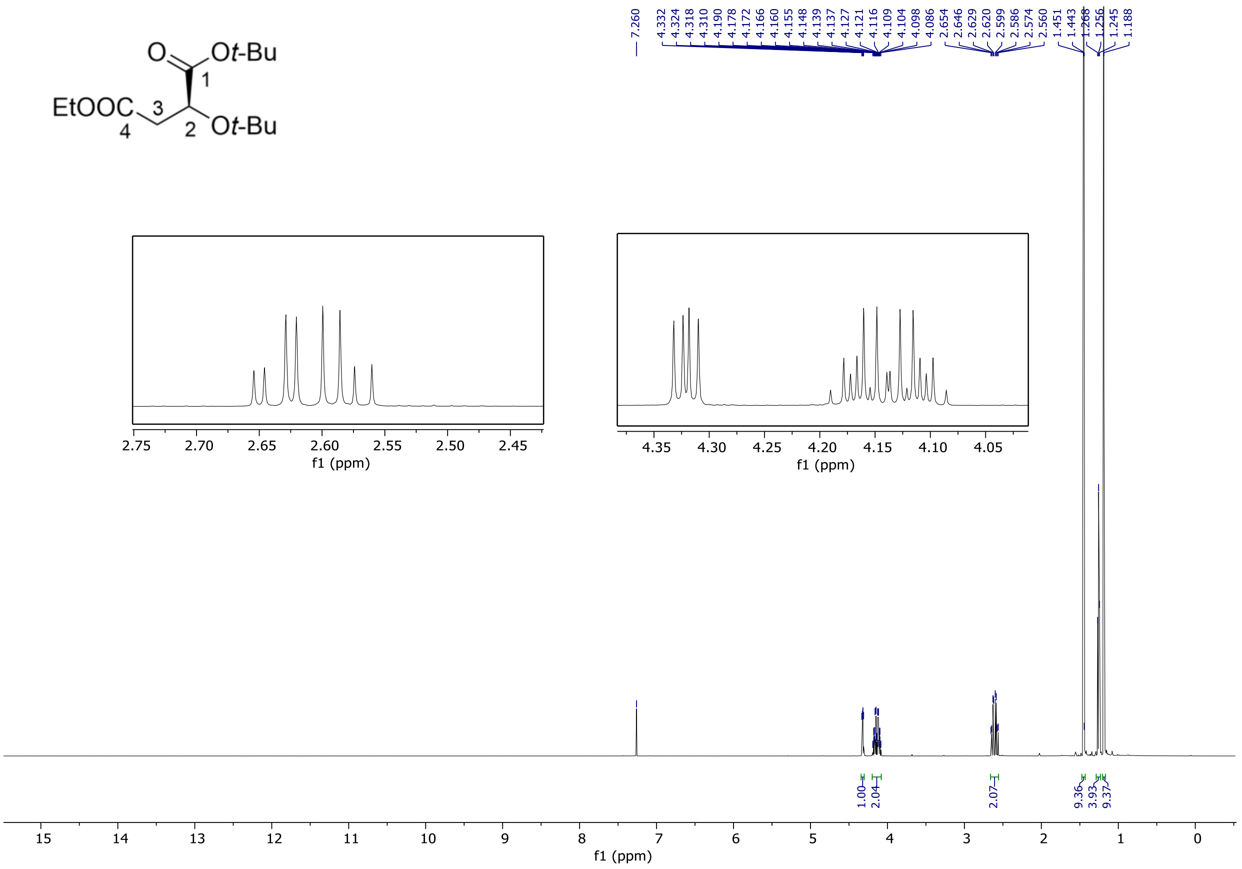

Figure 10: ^{13}C-DEPTQ (151 MHz, $CDCl_3$) of 1-(*tert*-butyl) 4-ethyl (*S*)-2-(*tert*-butoxy)succinate (**38**)

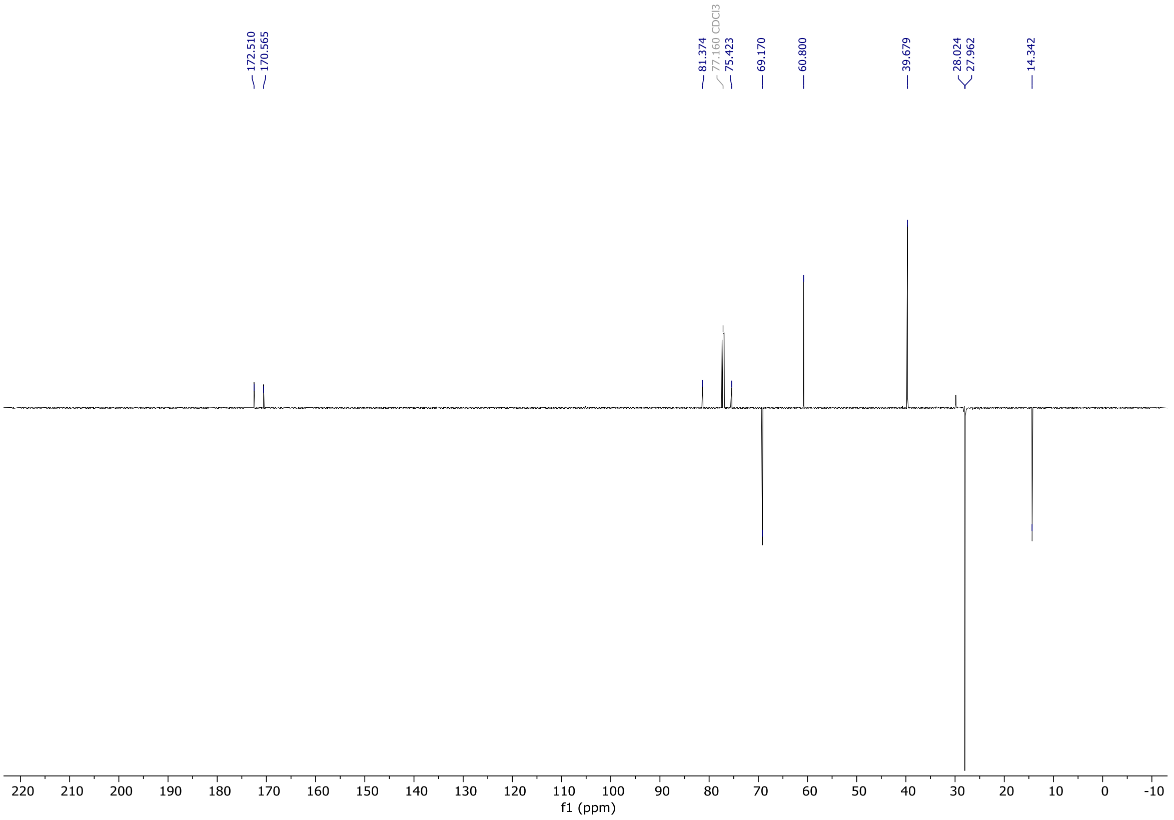

Figure 11: ^{1}H-NMR (600 MHz, $CDCl_3$) of *tert*-butyl (*S*)-2-(*tert*-butoxy)-4-oxobutanoate (**1**)

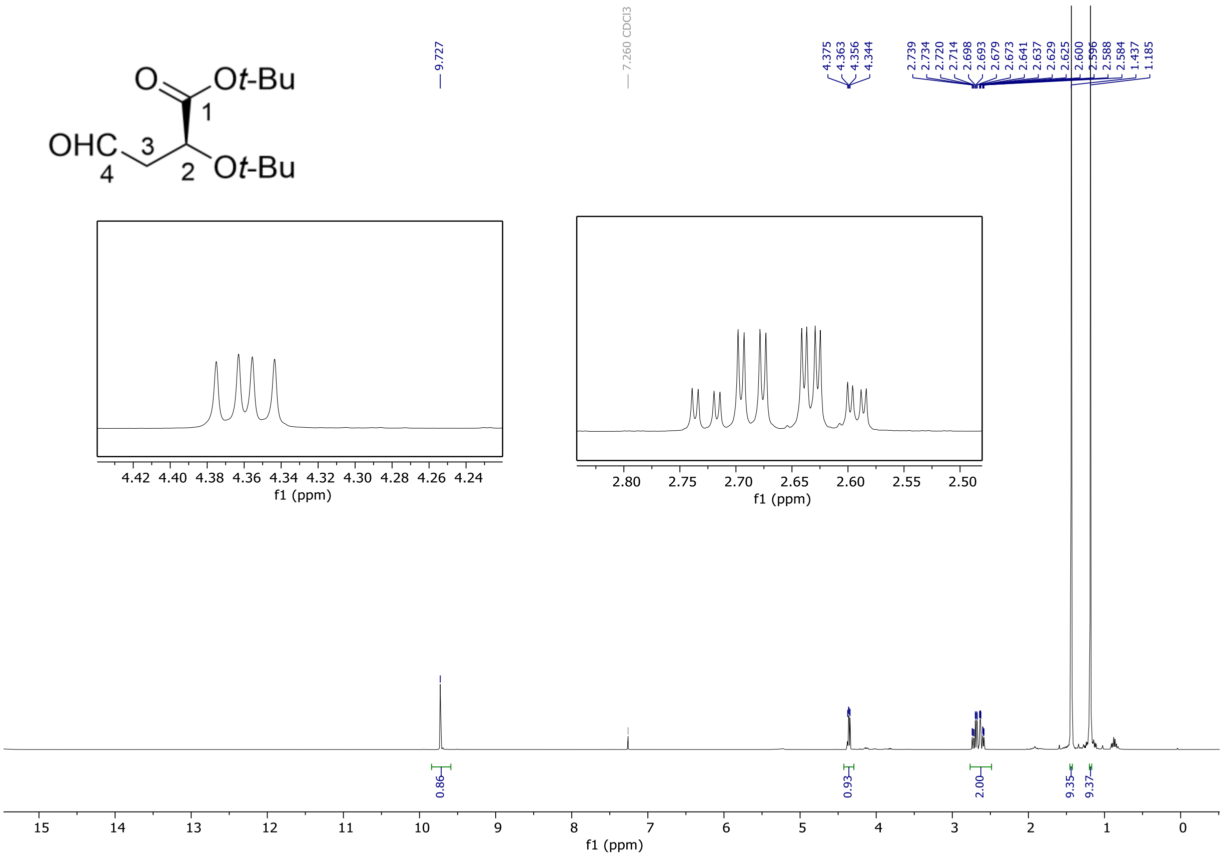

Figure 12: ^{13}C-DEPTQ (151 MHz, $CDCl_3$) of *tert*-butyl (*S*)-2-(*tert*-butoxy)-4-oxobutanoate (**1**)

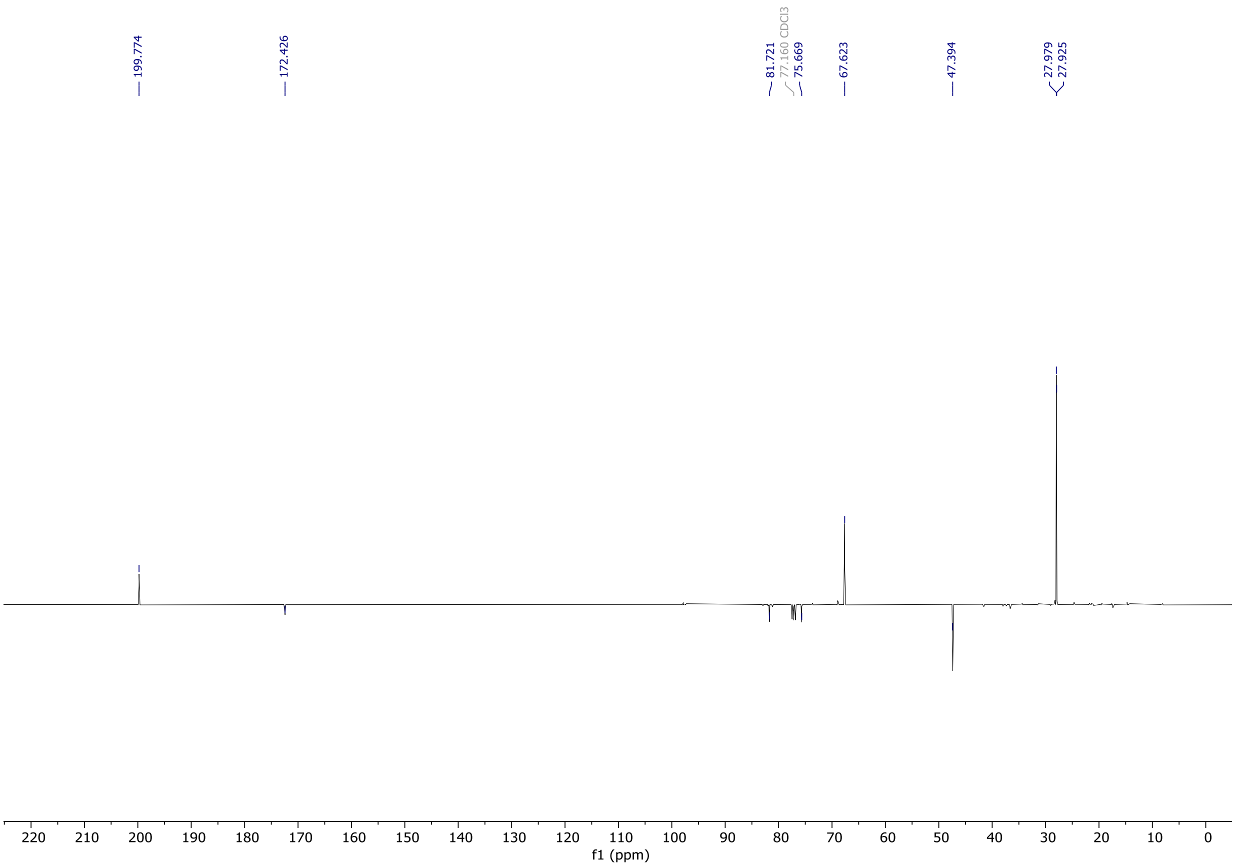

Figure 13: ^{1}H-NMR (600 MHz, $CDCl_3$) of *tert*-butyl (*S*)-2-aminopent-4-enoate (**3**)

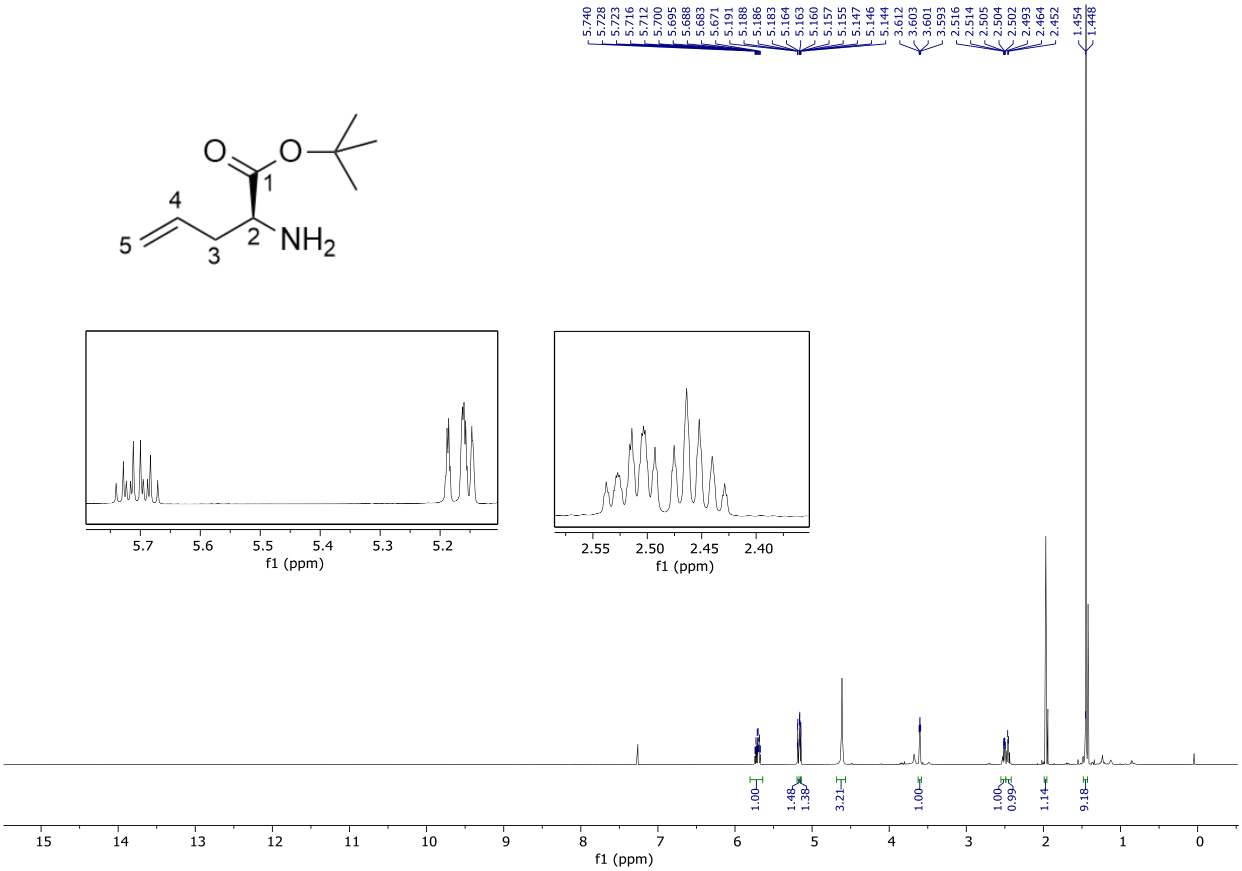

Figure 14: ^{13}C-DEPTQ (151 MHz, $CDCl_3$) of *tert*-butyl (*S*)-2-aminopent-4-enoate (**3**)

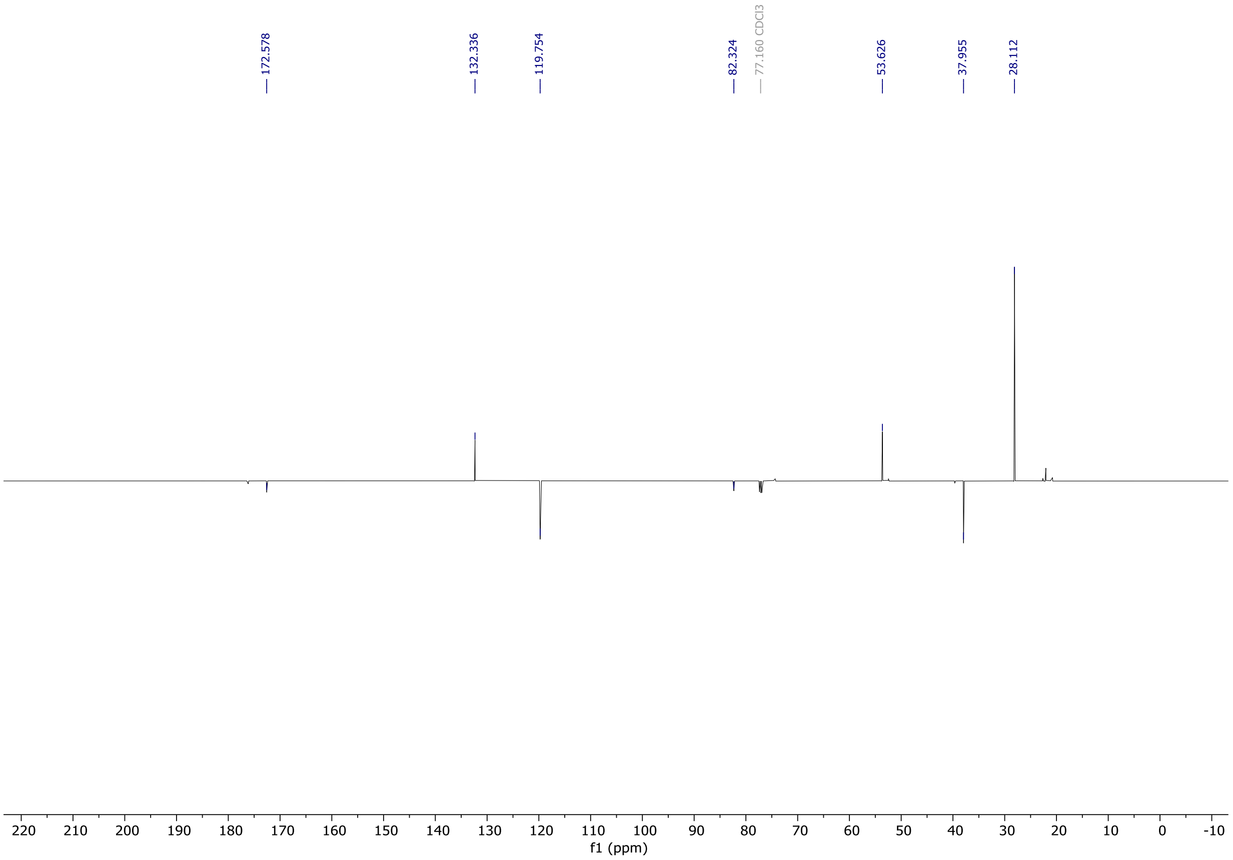

Figure 15: ^{1}H-NMR (400 MHz, $CDCl_3$) of *tert*-butyl 2-(((1*S*,2*S*,5*S*)-2-hydroxy-2,6,6-trimethylbicyclo[3.1.1]heptan-3-ylidene)amino)acetate (**15**)

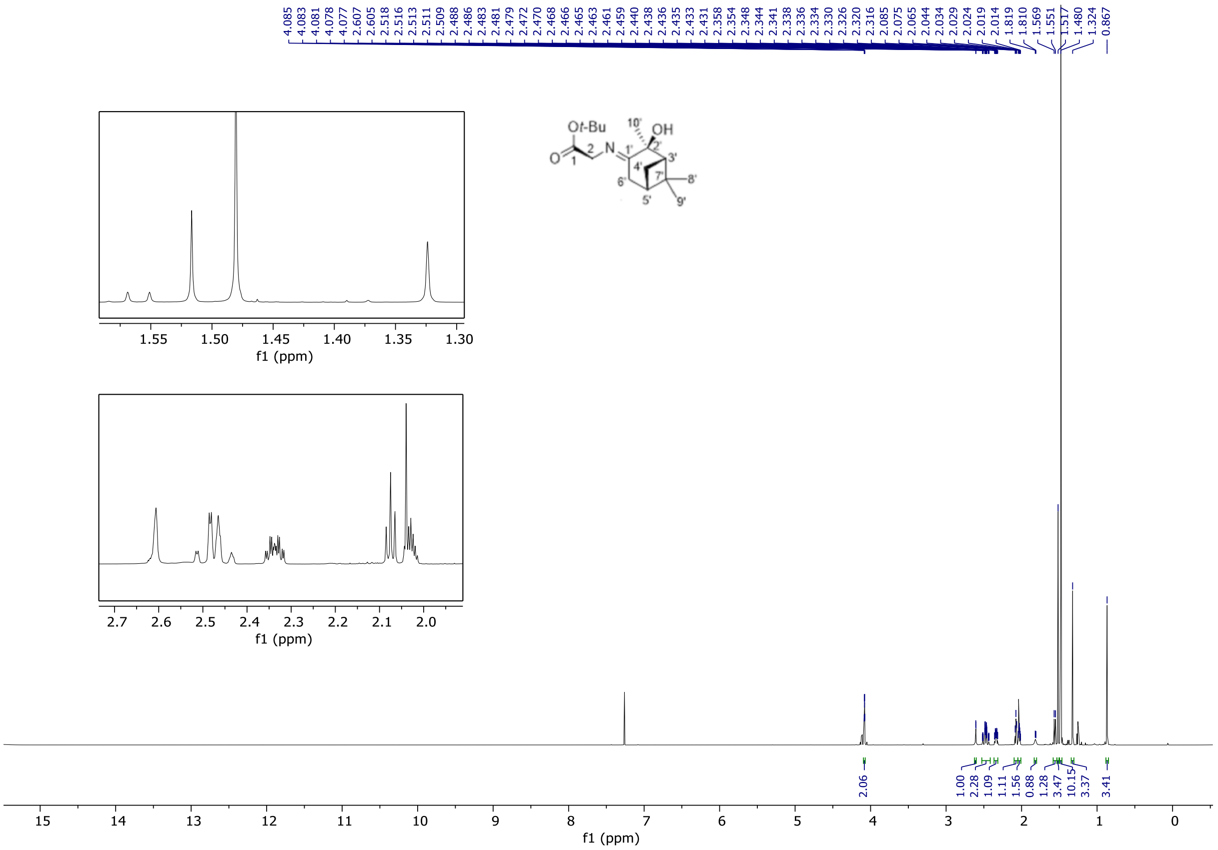

Figure 16: ^{13}C-NMR (101 MHz, $CDCl_3$) of *tert*-butyl 2-(((1*S*,2*S*,5*S*)-2-hydroxy-2,6,6-trimethylbicyclo[3.1.1]heptan-3-ylidene)amino)acetate (**15**)

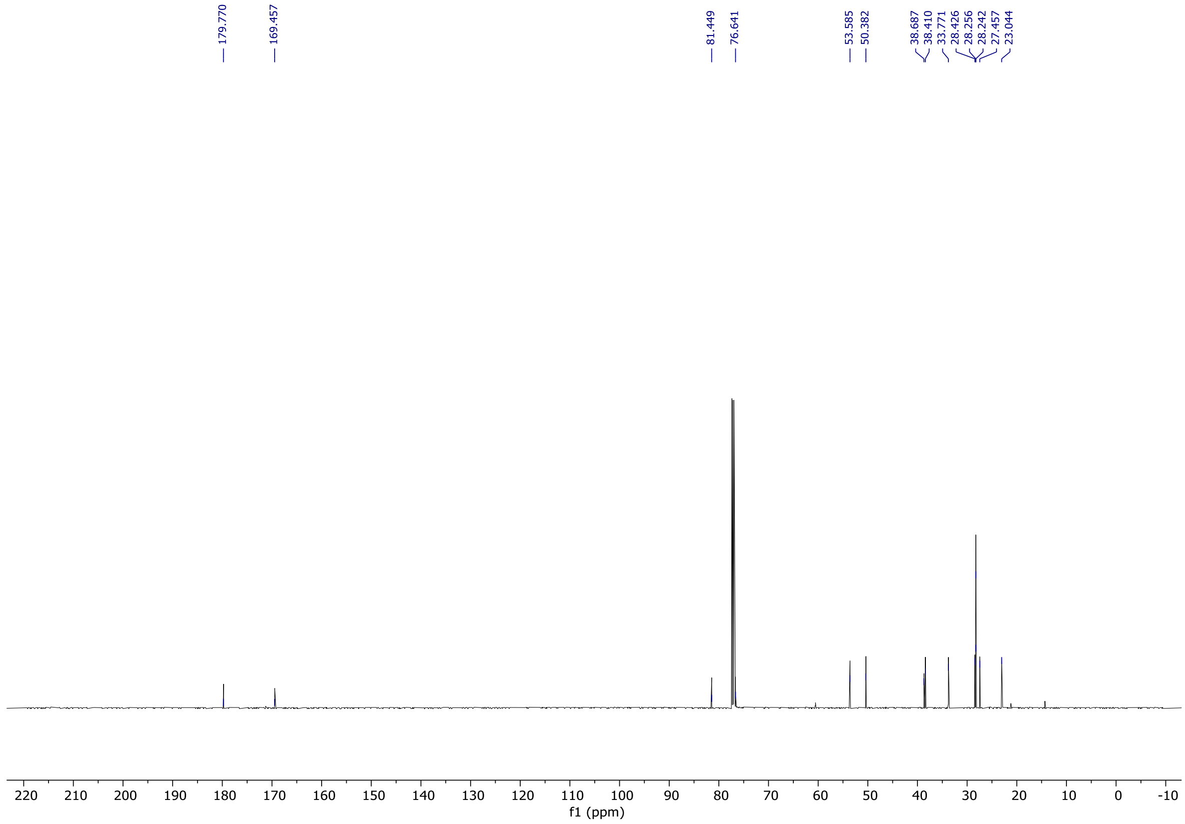

Figure 17: ^{1}H-NMR (600 MHz, CD_2Cl_2) of *tert*-butyl (2*S*,3*S*,*E*)-2-amino-3-hydroxyhex-4-enoate (**2**)

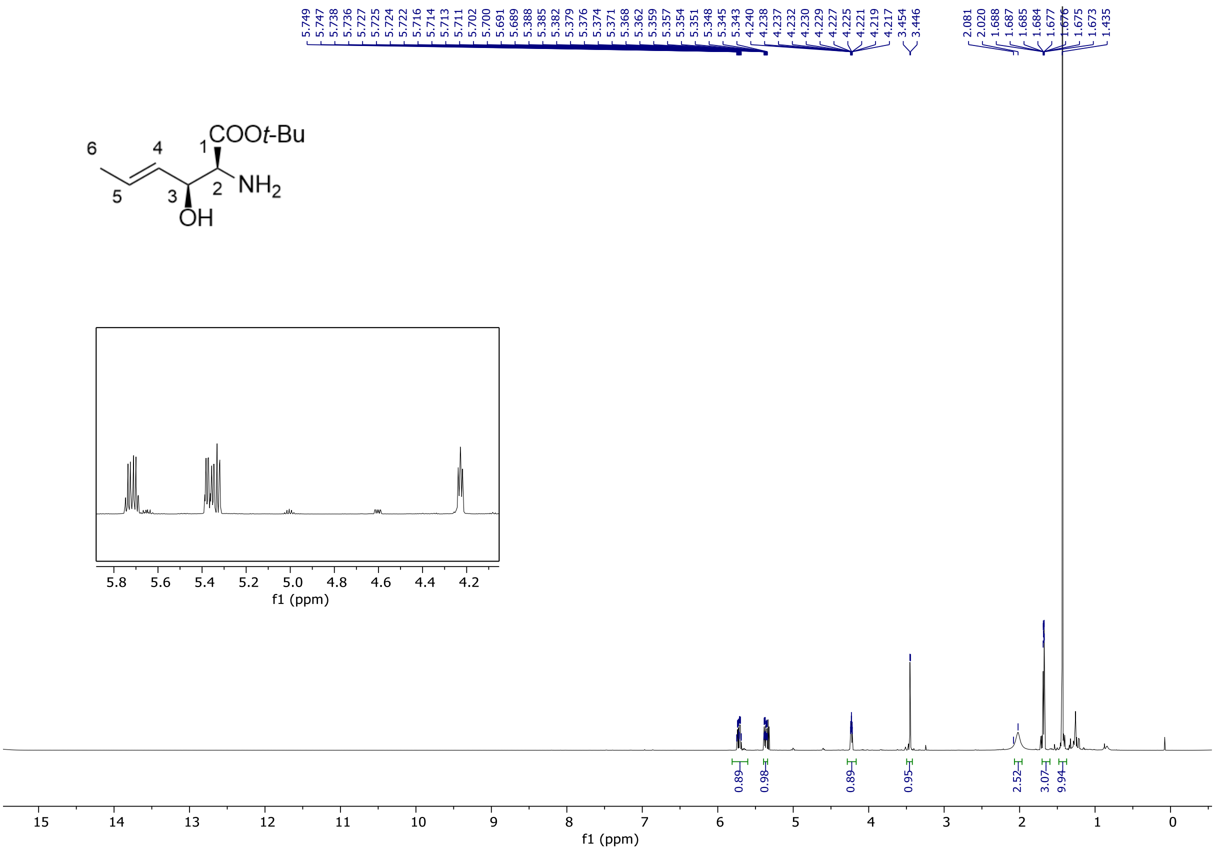

Figure 18: ^{13}C-NMR (151 MHz, CD_2Cl_2) of *tert*-butyl (2*S*,3*S*,*E*)-2-amino-3-hydroxyhex-4-enoate (**2**)

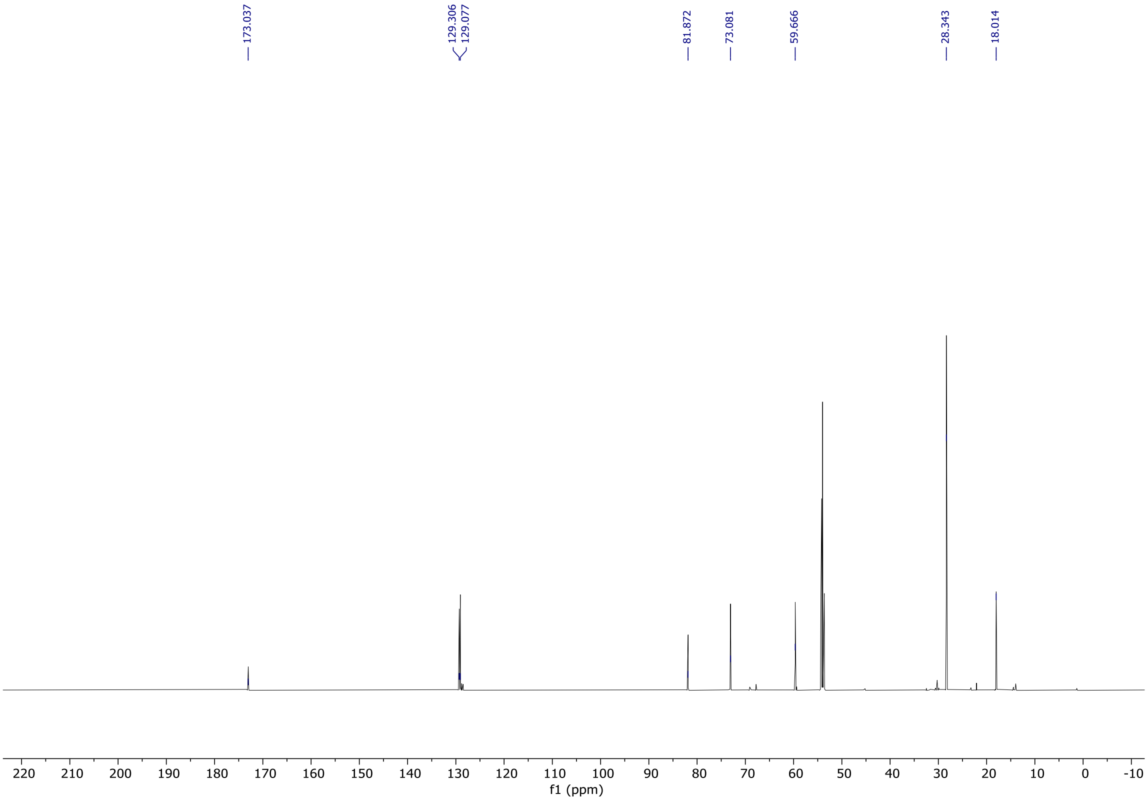

Figure 19: ^{1}H-NMR (600 MHz, CD_2Cl_2) of *tert*-butyl (2*S*,3*S*,*E*)-2-amino-3-hydroxyhex-4-enoate hydrochloride (**2**·HCl)

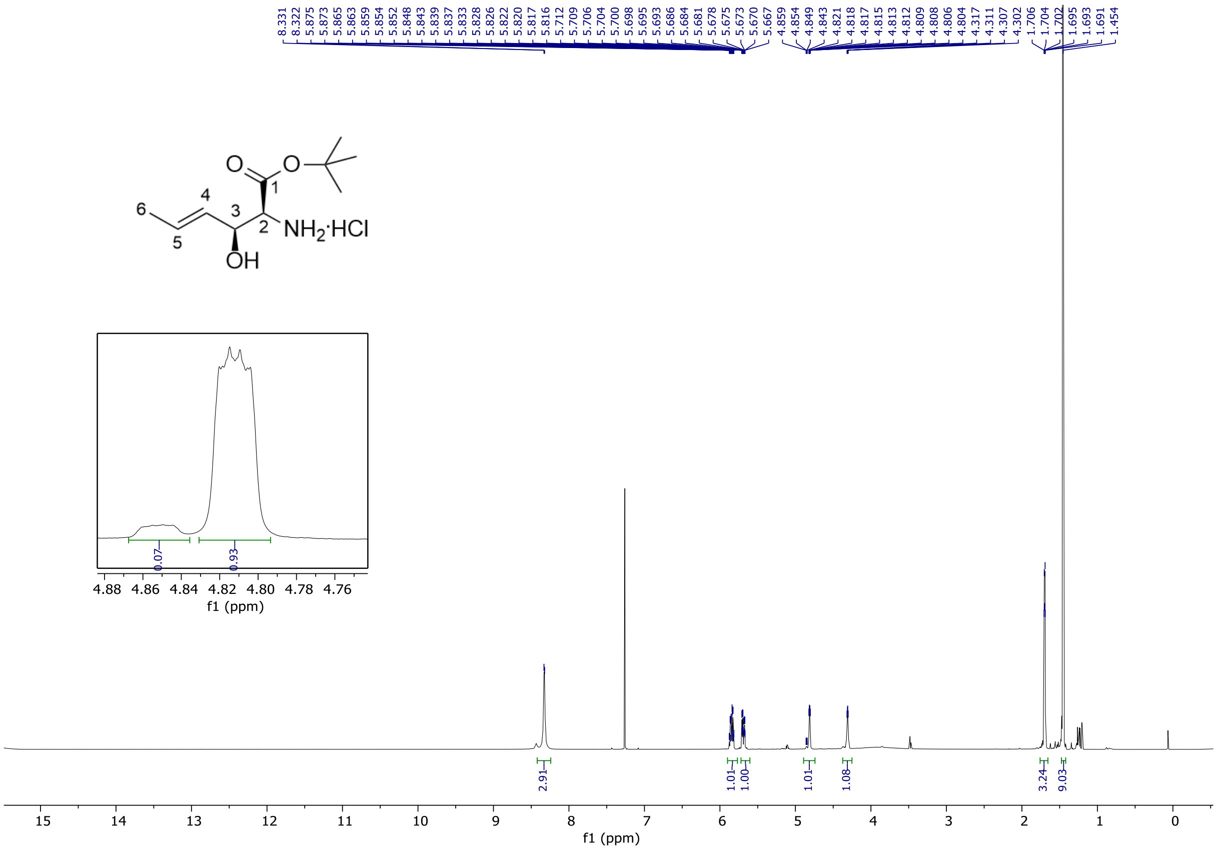

Figure 20: ^{13}C-NMR (600 MHz, CD_2Cl_2) of *tert*-butyl (2*S*,3*S*,*E*)-2-amino-3-hydroxyhex-4-enoate hydrochloride (**2**·HCl)

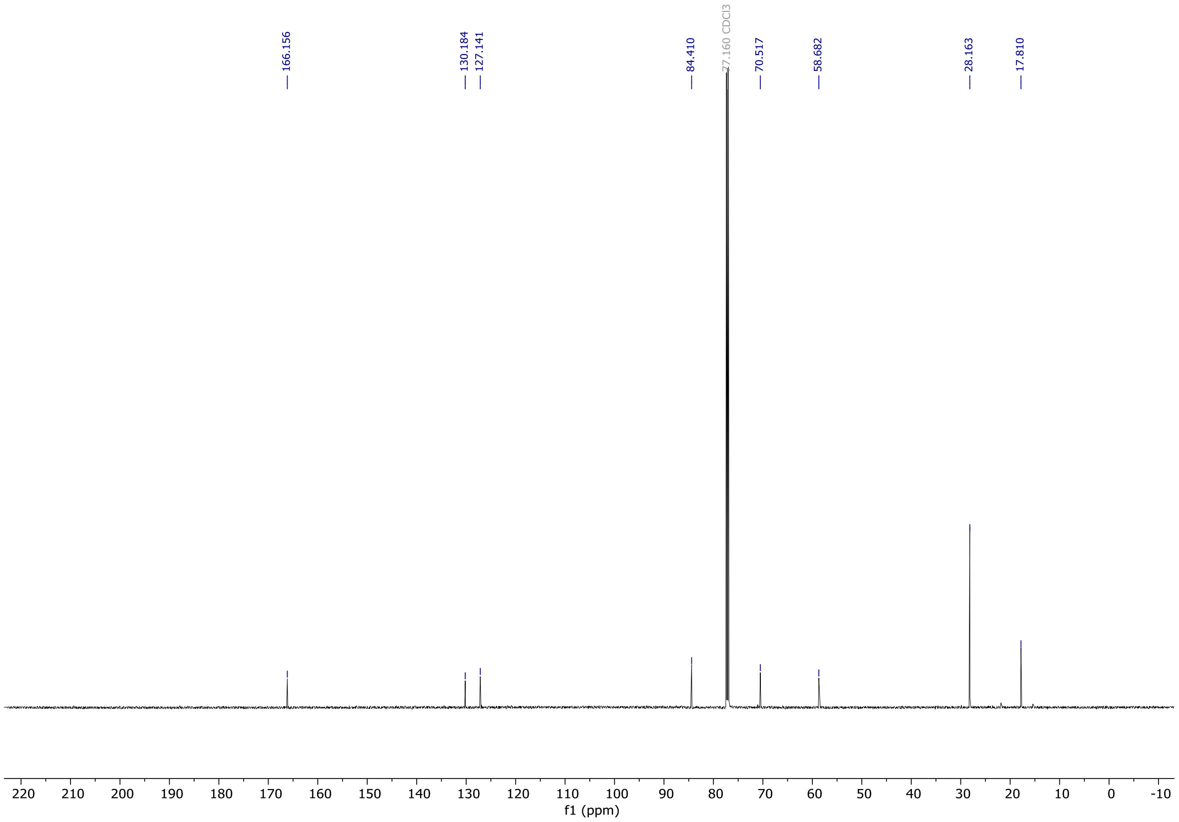

Figure 21: ^{1}H-NMR (400 MHz, $CDCl_3$) of methyl *O*-(*tert*-butyl)-*N*-picolinoyl-L-threoninate (**41**)

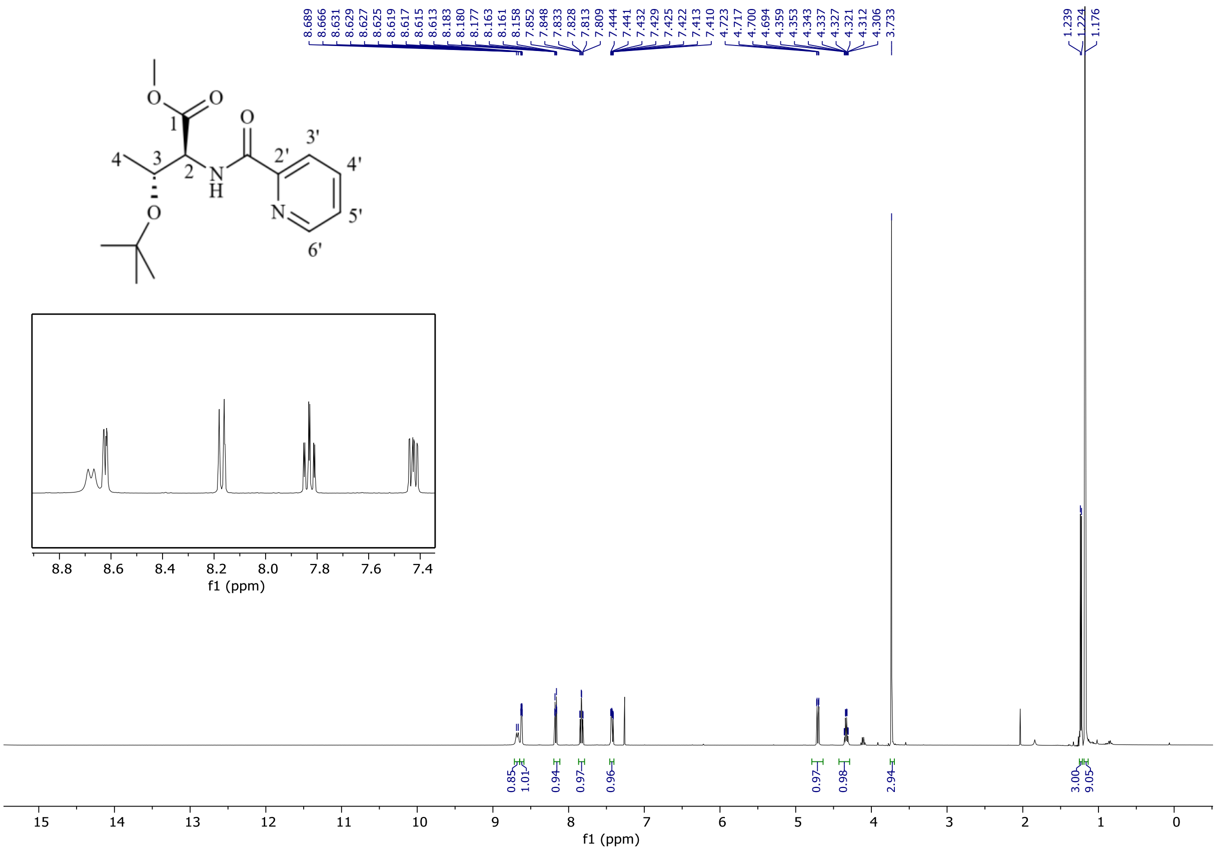

Figure 22: ^{13}C-DEPTQ (101 MHz, $CDCl_3$) of methyl *O*-(*tert*-butyl)-*N*-picolinoyl-L-threoninate (**41**)

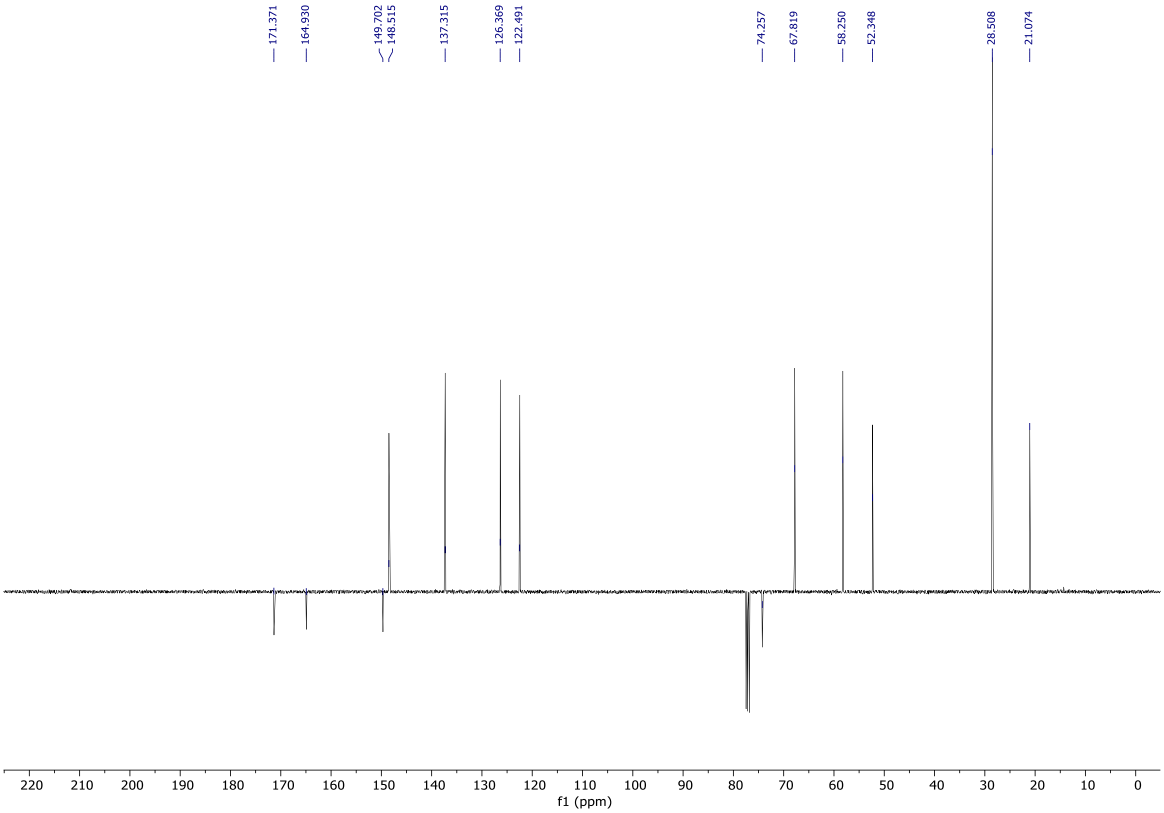

Figure 23: ^{1}H-NMR (600 MHz, $CDCl_3$) of methyl *O*-(*tert*-butyl)-*N*-picolinoyl-D-threoninate (*ent*-**41**)

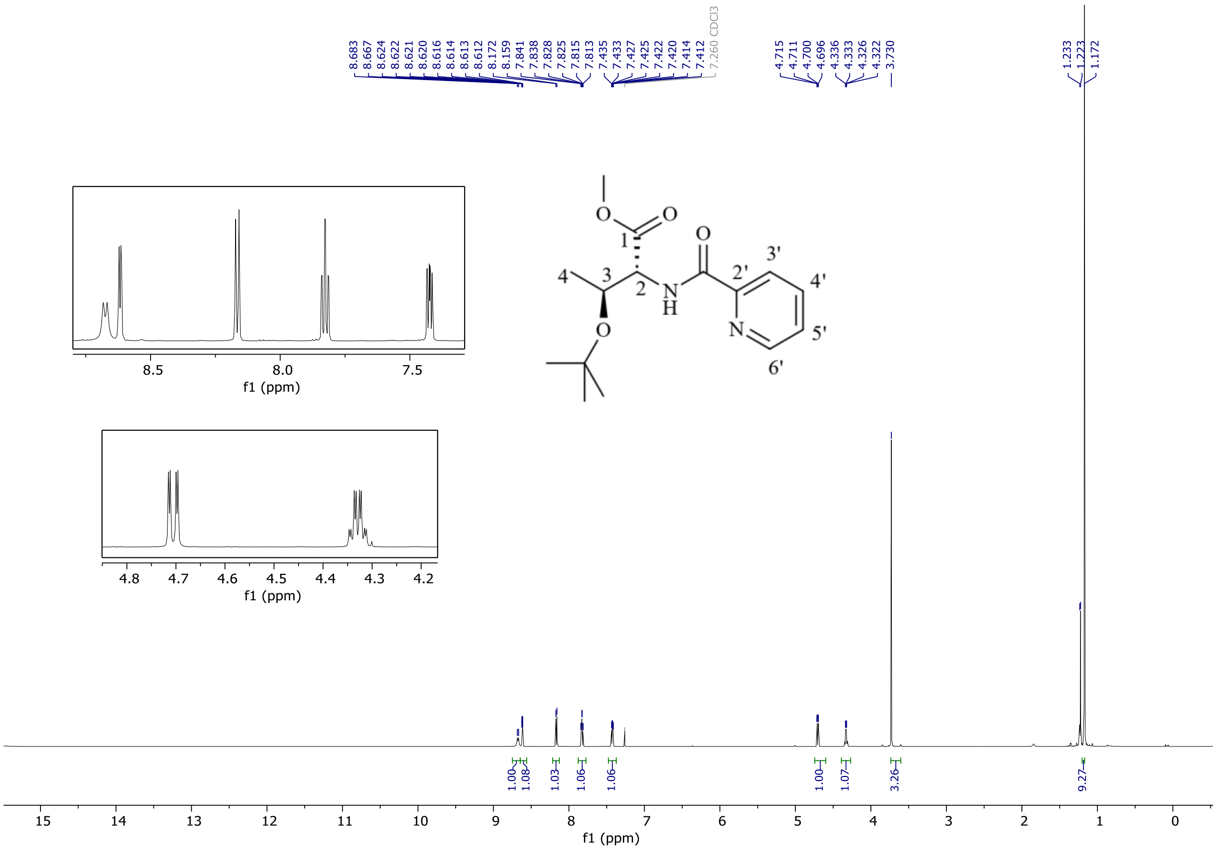

Figure 24: ^{13}C-NMR (151 MHz, $CDCl_3$) of Methyl *O*-(*tert*-butyl)-*N*-picolinoyl-D-threoninate (*ent*-**41**)

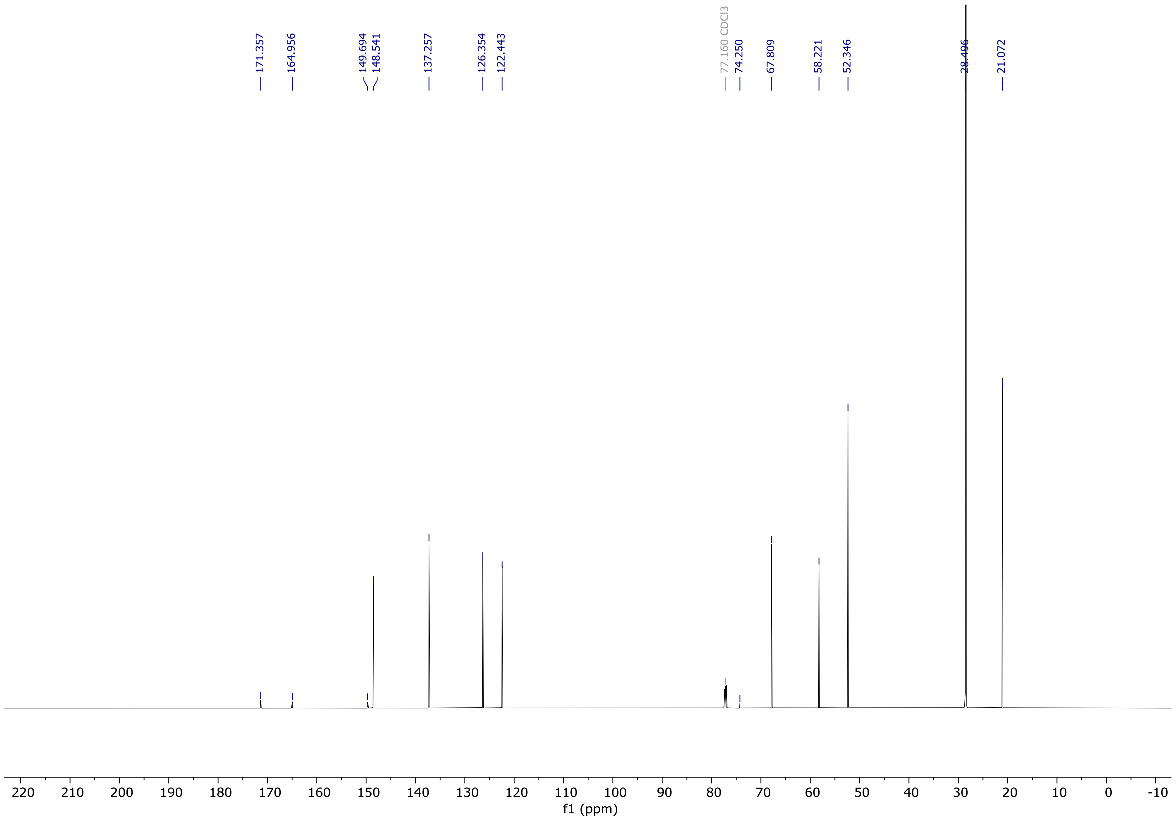

Figure 25: ^{1}H-NMR of (400 MHz, $CDCl_3$) of methyl (2*S*,3*R*)-3-(*tert*-butoxy)-1-picolinoylazetidine-2-carboxylate (**19**)

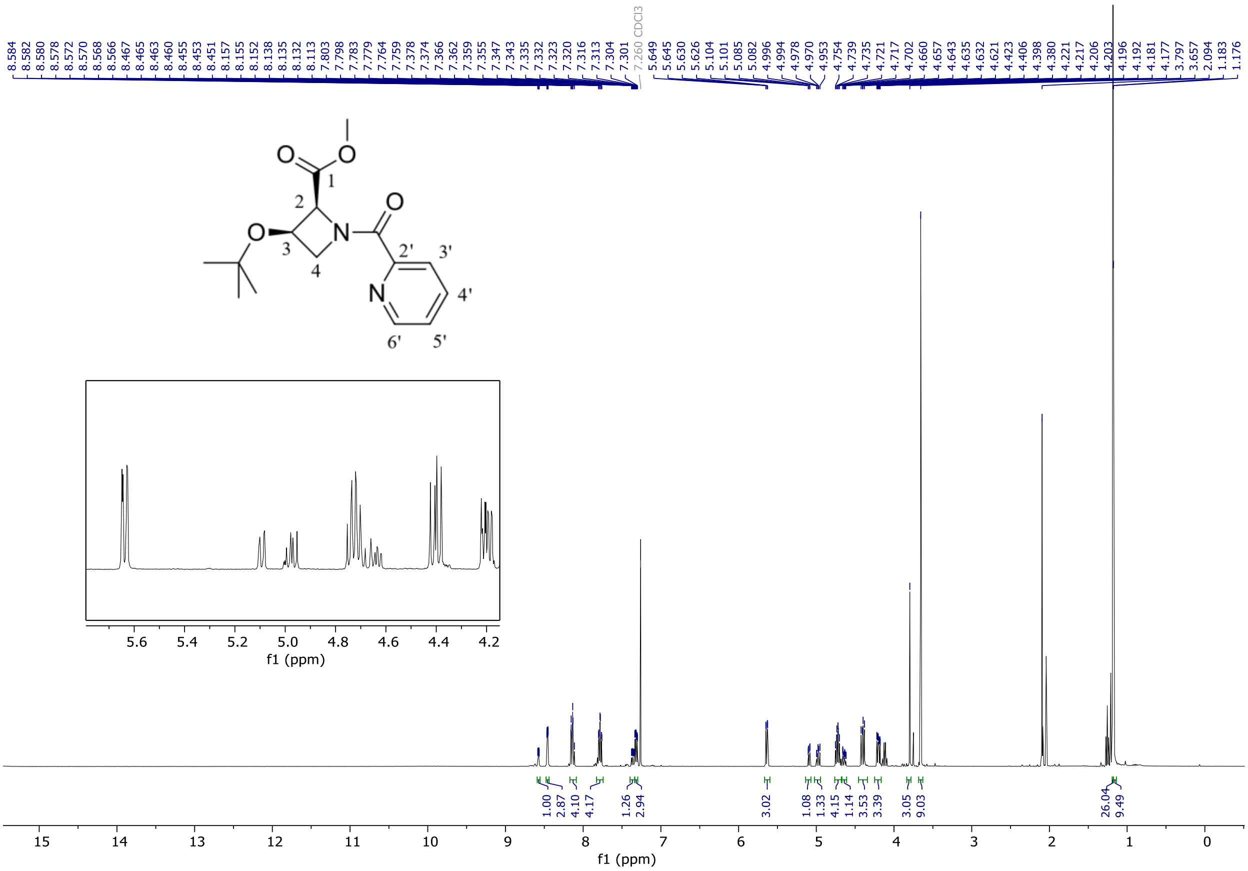

Figure 26: ^{13}C-DEPTQ (101 MHz, $CDCl_3$) of Methyl (2*S*,3*R*)-3-(*tert*-butoxy)-1-picolinoylazetidine-2-carboxylate (**19**)

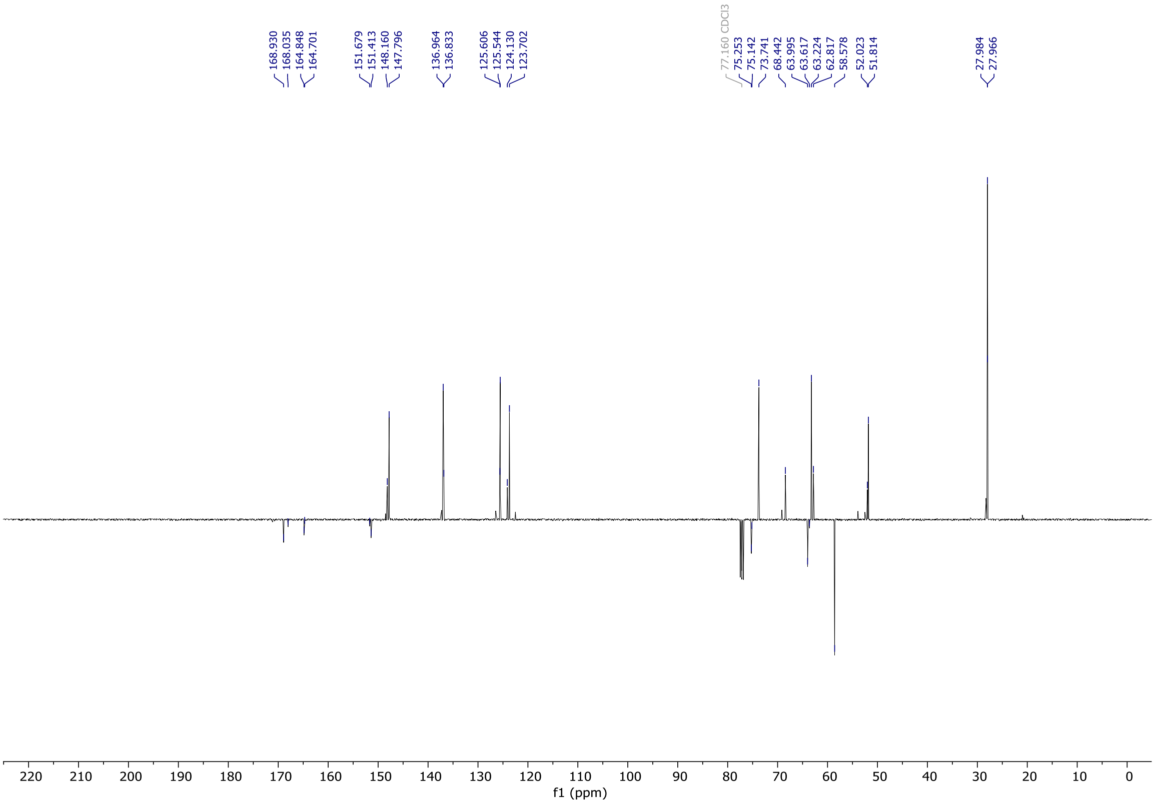

Figure 27: ^{1}H-NMR (600 MHz, $CDCl_3$) of methyl (2*R*,3*S*)-3-(*tert*-butoxy)-1-picolinoylazetidine-2-carboxylate (*ent*-**19**)

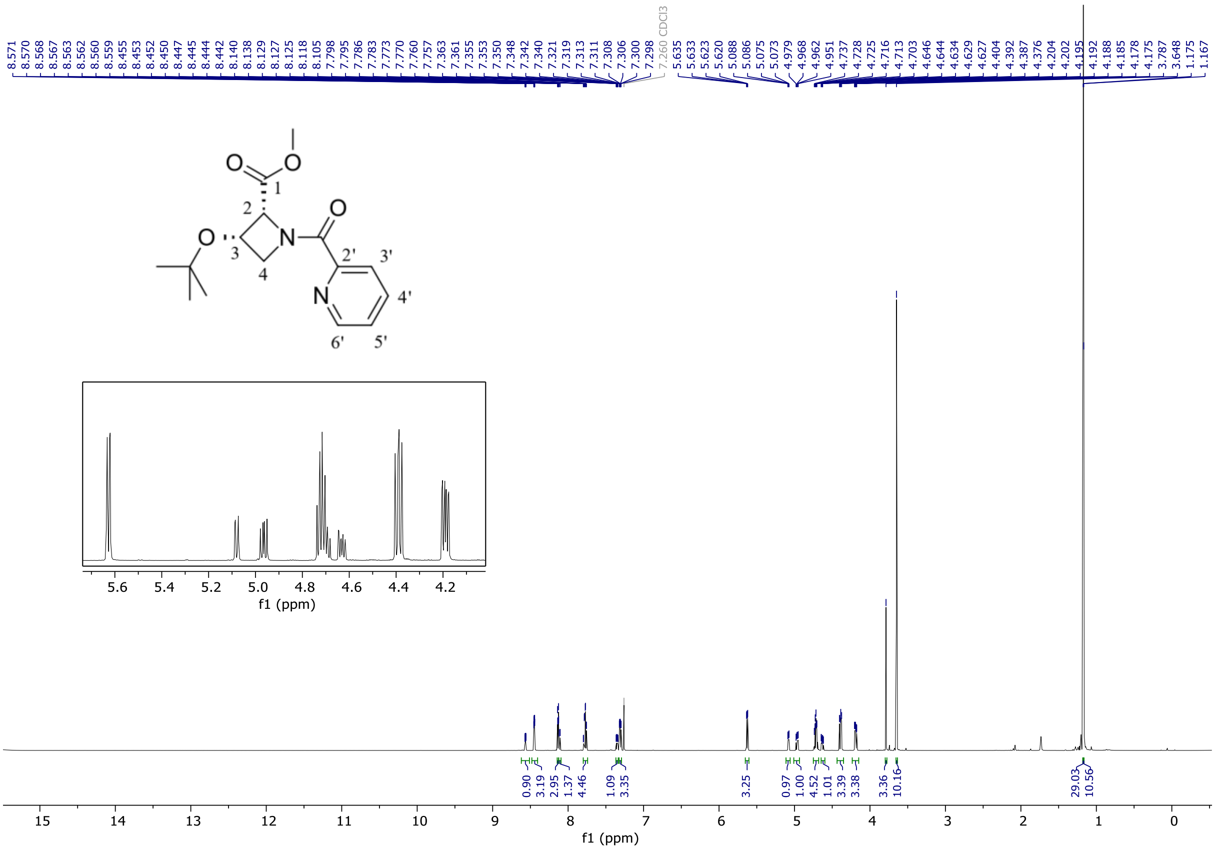

Figure 28: ^{13}C-DEPTQ (151 MHz, $CDCl_3$) of methyl (2*R*,3*S*)-3-(*tert*-butoxy)-1-picolinoylazetidine-2-carboxylate (*ent*-**19**)

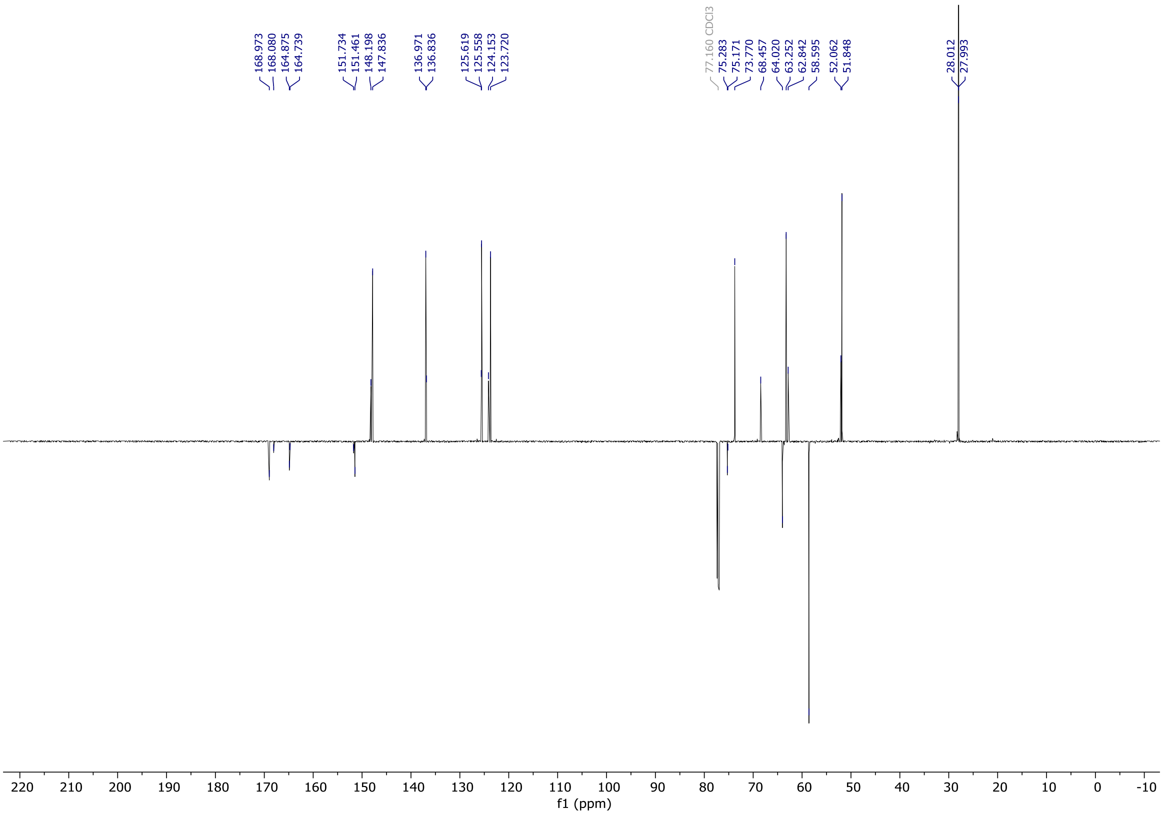

Figure 29: ^{1}H-NMR of (600 MHz, CD_3OD) of (2*S*,3*S*)-3-(*tert*-butoxy)azetidine-2-carboxylic acid (**5**)

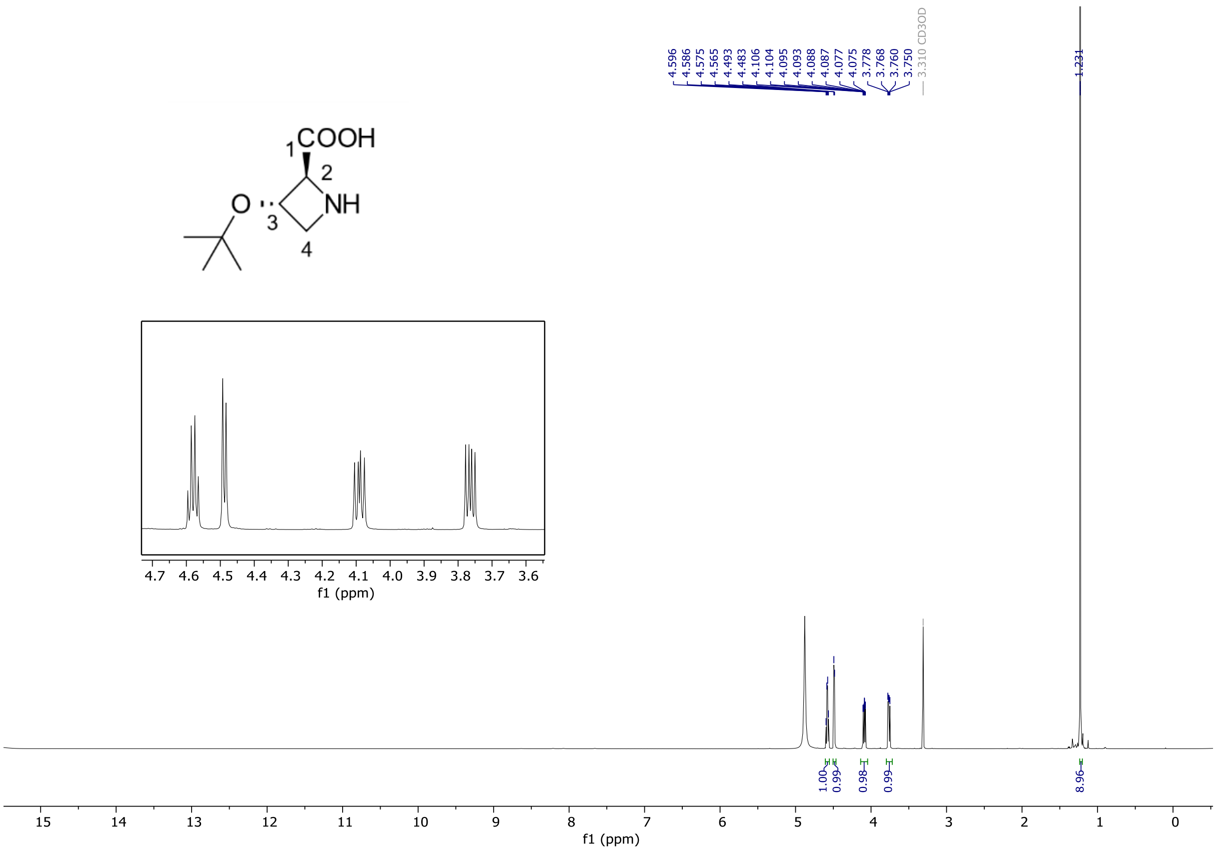

Figure 30: ^{13}C-DEPTQ (151 MHz, CD_3OD) of (2*S*,3*S*)-3-(*tert*-butoxy)azetidine-2-carboxylic acid (**5**)

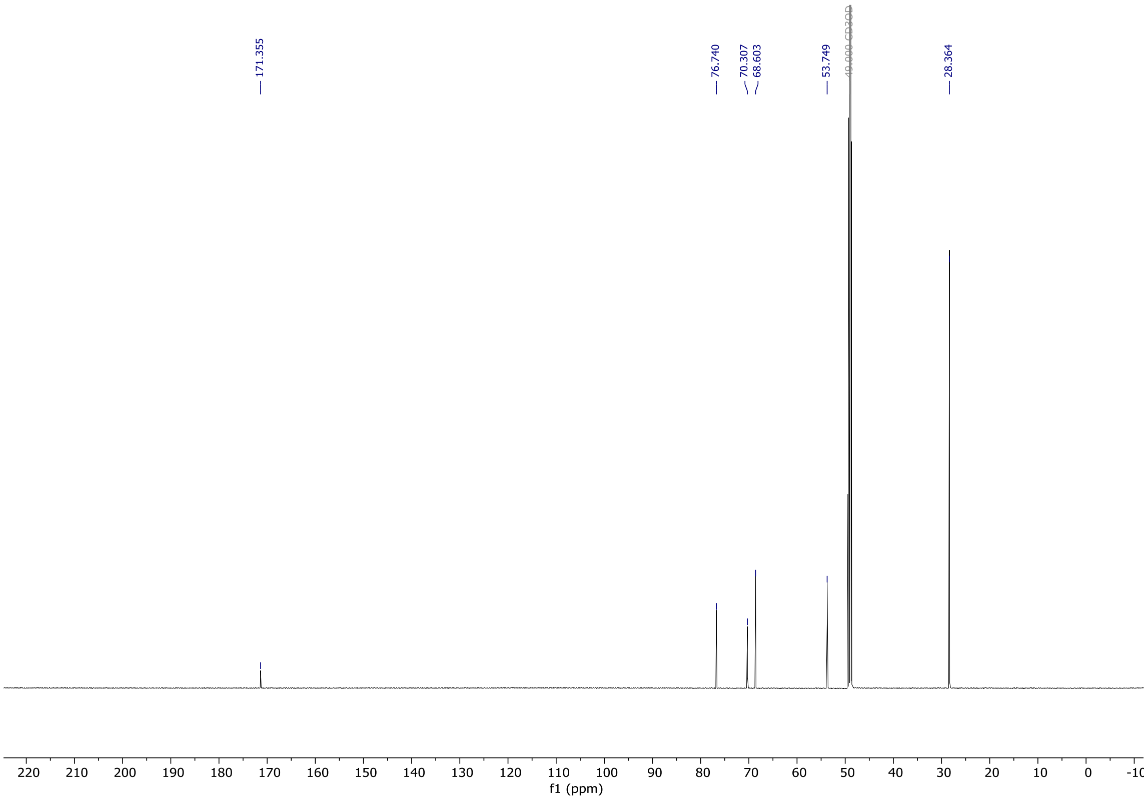

Figure 31: ^{1}H-NMR (400 MHz, CD_3OD) of (2*S*,3*R*)-3-(*tert*-butoxy)azetidine-2-carboxylic acid (**4**)

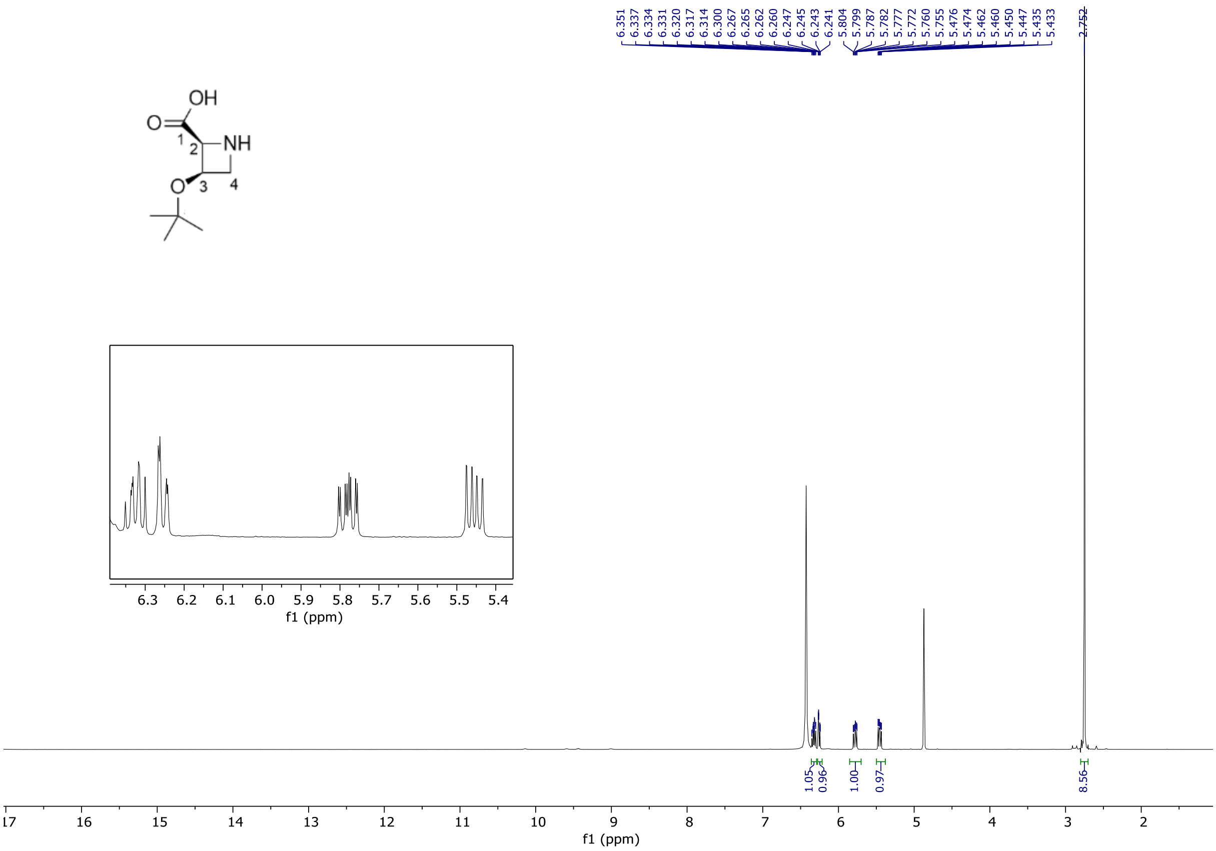

Figure 32: ^{13}C-DEPTQ of (101 MHz, D_2O) of (2*S*,3*R*)-3-(*tert*-butoxy)azetidine-2-carboxylic acid (**4**)

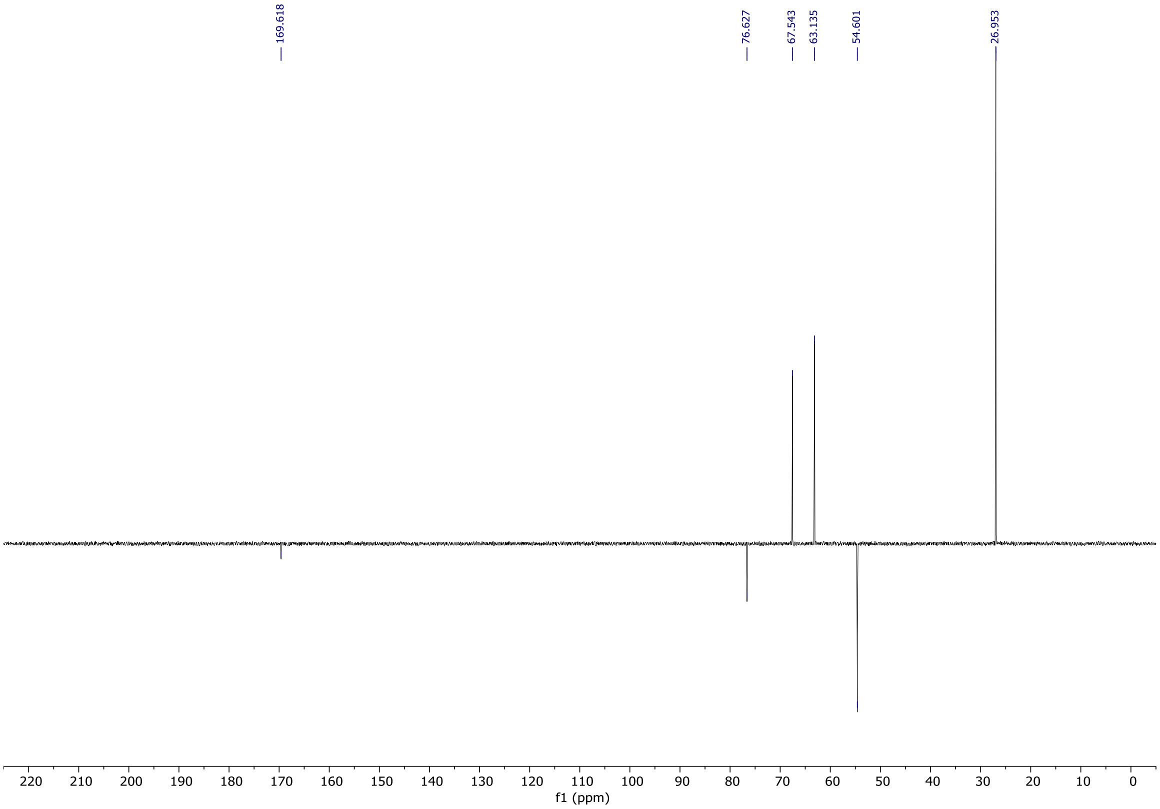

Figure 33: ^{1}H-NMR (400 MHz, CD_3OD) of (2*R*,3*R*)-3-(*tert*-butoxy)azetidine-2-carboxylic acid (*ent*-**5**)

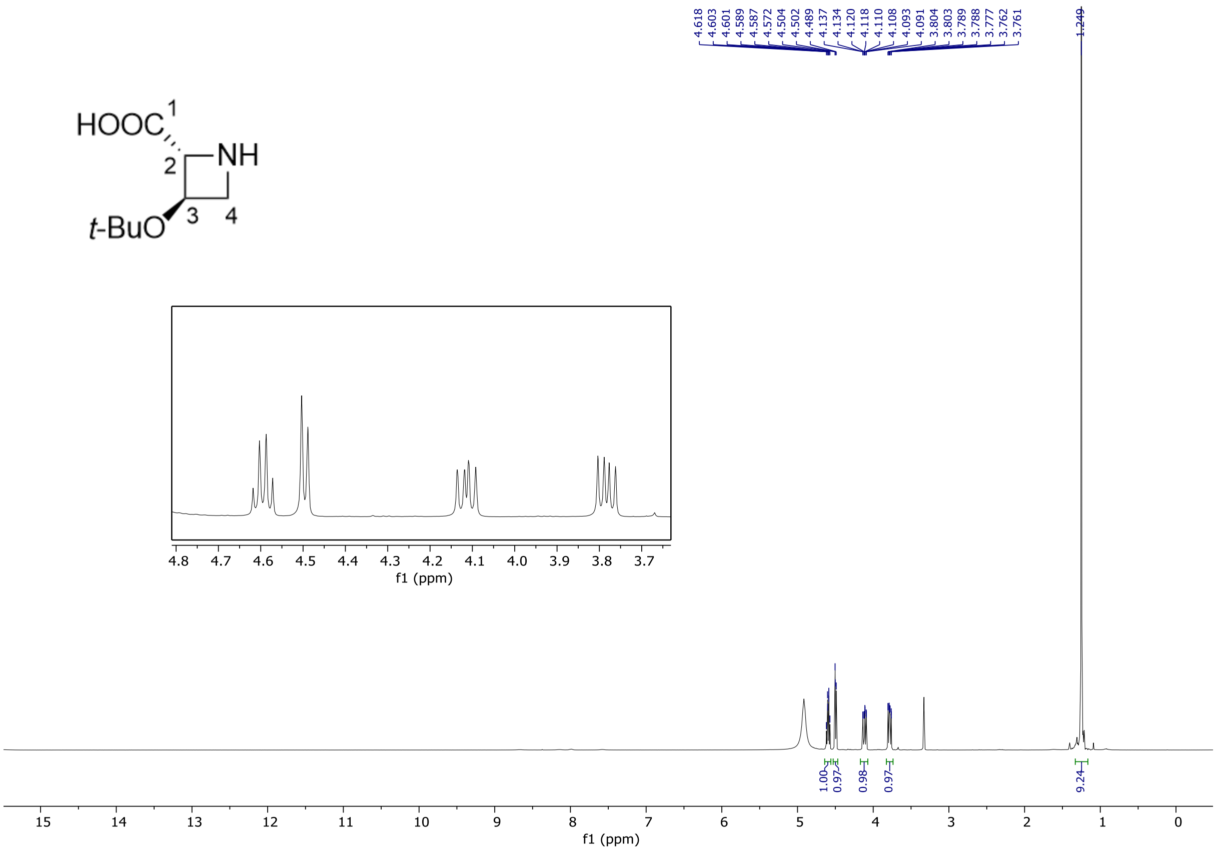

Figure 34: ^{13}C-APT (101 MHz, CD_3OD) of (2*R*,3*R*)-3-(*tert*-butoxy)azetidine-2-carboxylic acid (*ent*-**5**)

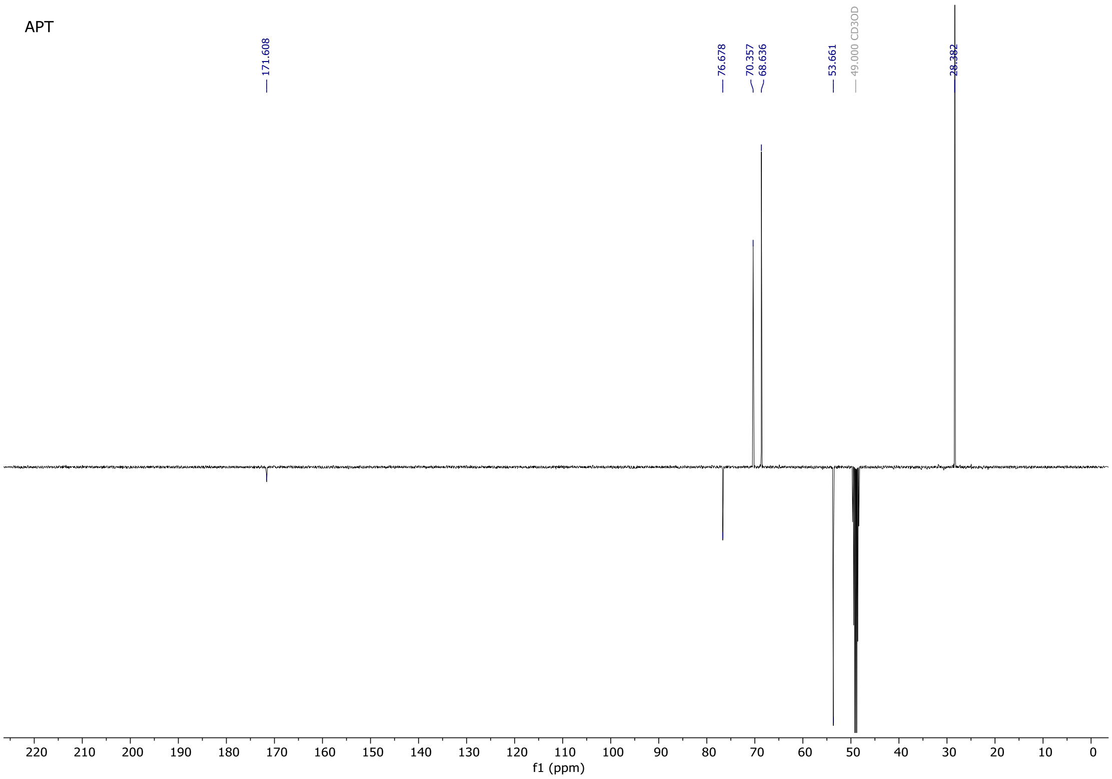

Figure 35: ^{1}H-NMR (600 MHz, $CDCl_3$) of (2*R*,3*S*)-3-(*tert*-butoxy)-1-picolinoylazetidine-2-carboxylic acid (**42**)

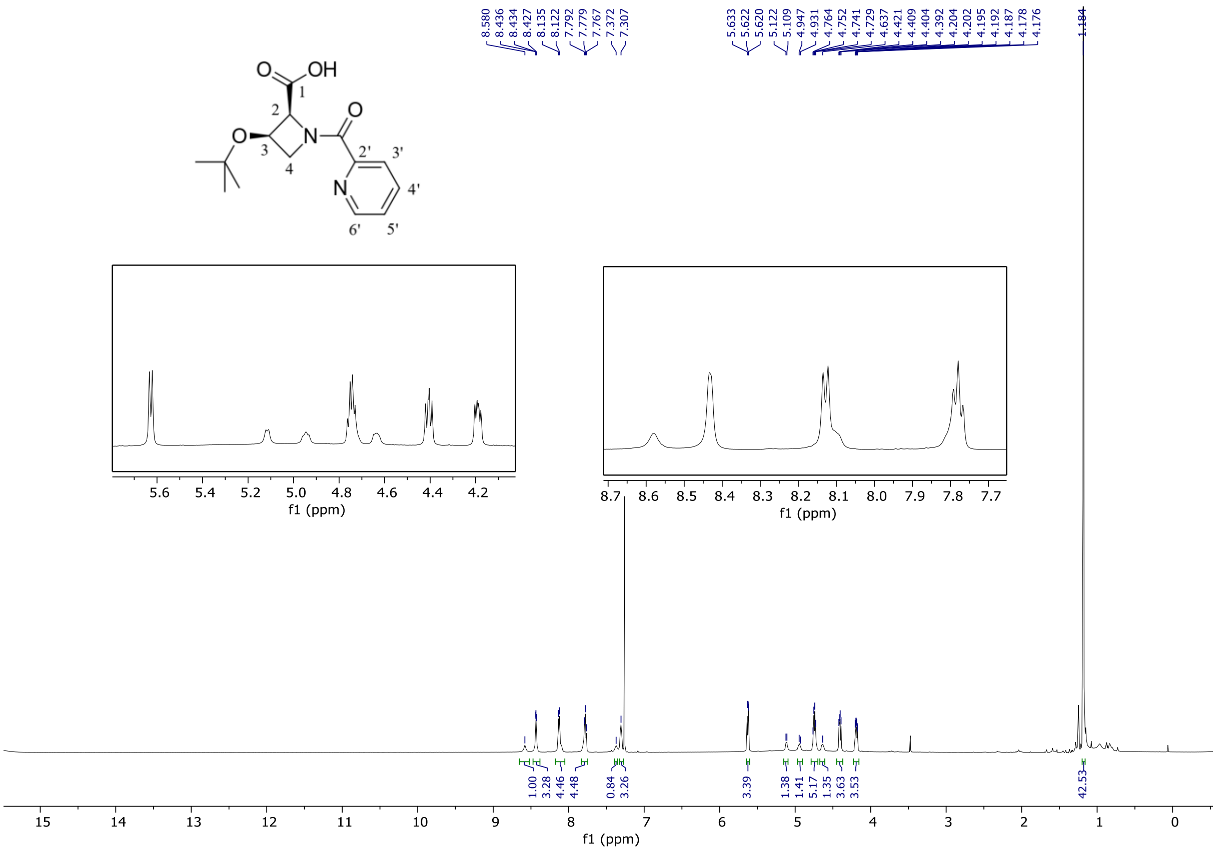

Figure 36: ^{13}C-DEPTQ (151 MHz, $CDCl_3$) of (2*R*,3*S*)-3-(*tert*-butoxy)-1-picolinoylazetidine-2-carboxylic acid (**42**)

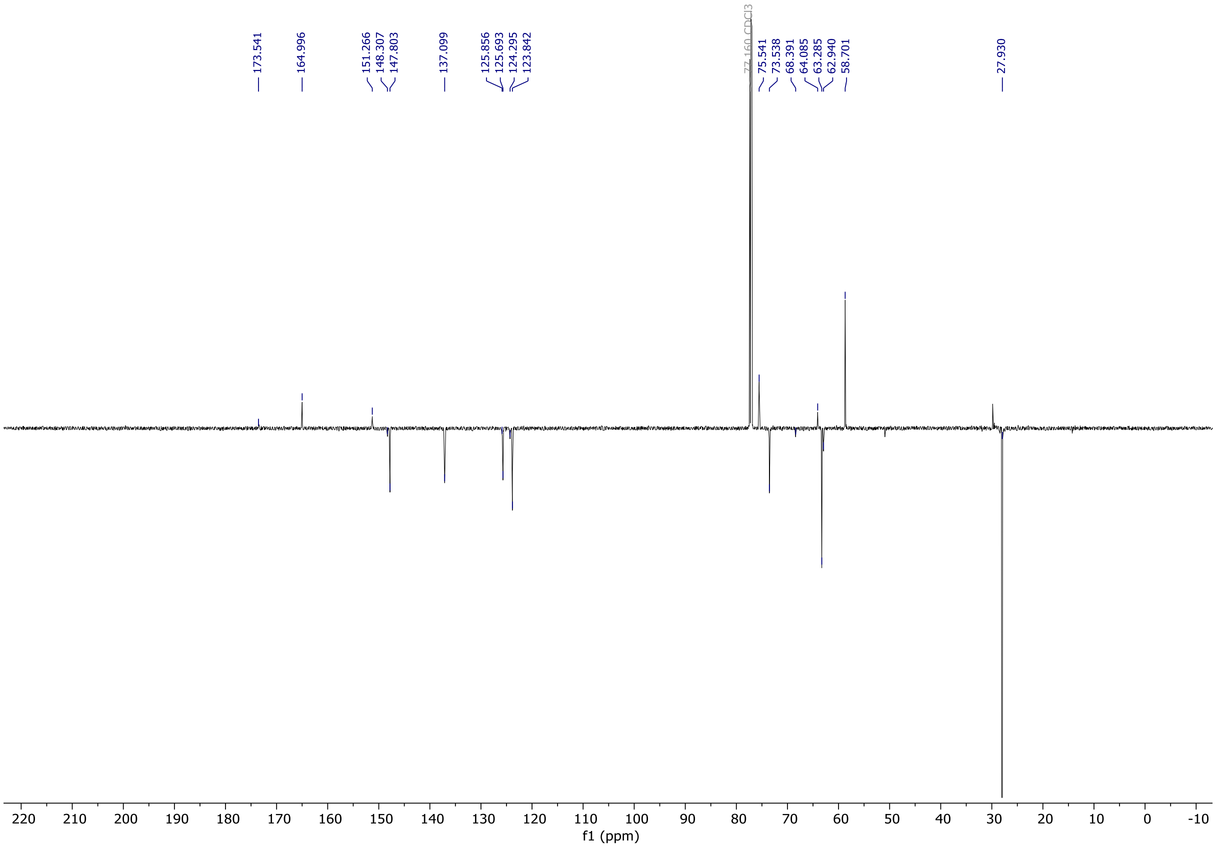

Figure 37: ^{1}H-NMR (600 MHz, CD_3OD) of (*S*)-3-aminodihydrofuran-2(3H)-one hydrochloride (**7**)

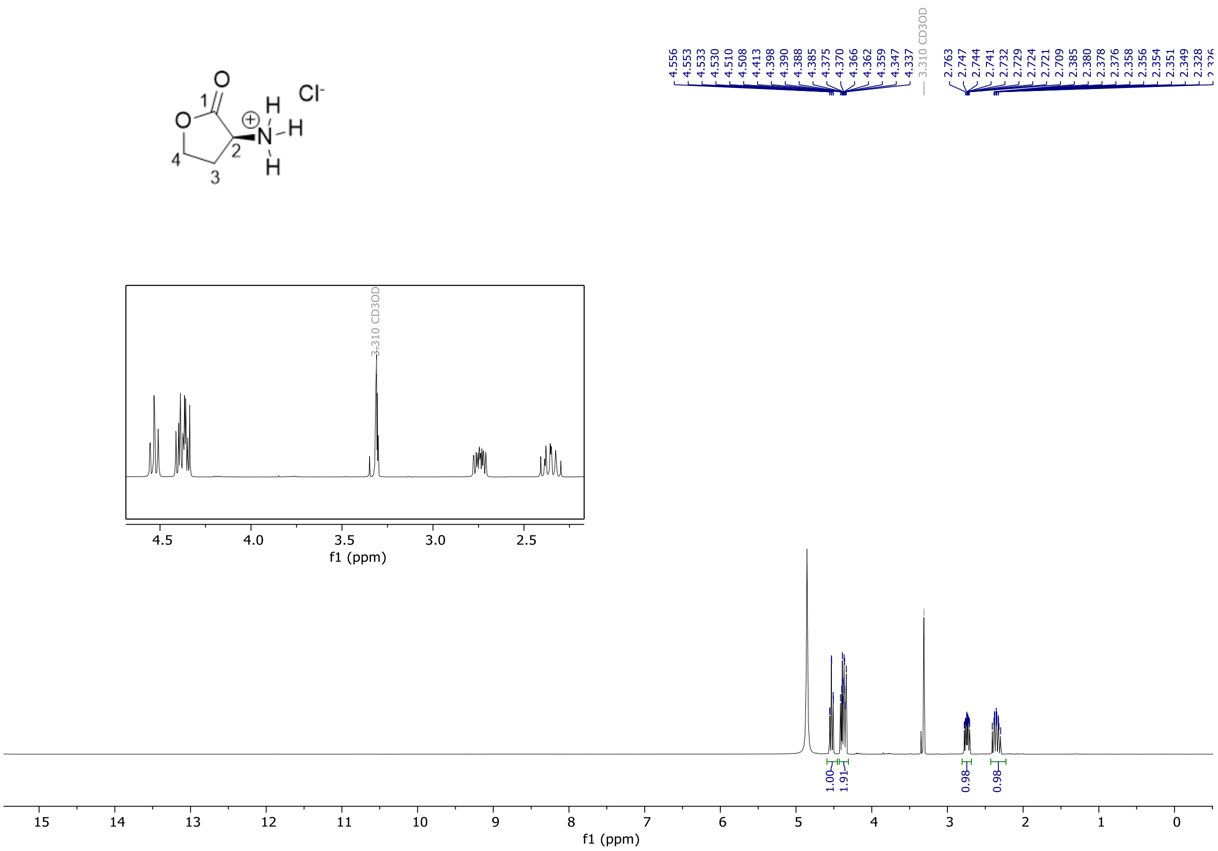

Figure 38: ^{13}C-NMR of (151 MHz, CD_3OD) of (*S*)-3-aminodihydrofuran-2(3H)-one hydrochloride (**7**)

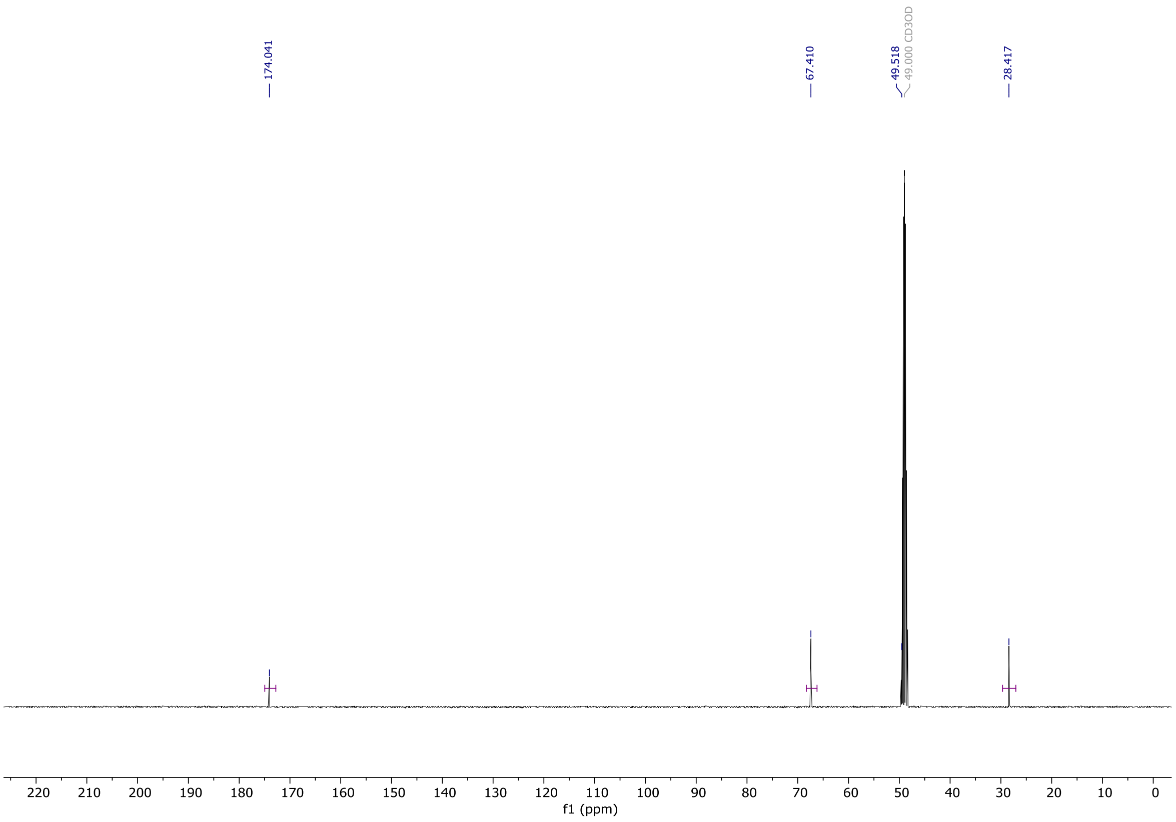

Figure 39: ^{1}H-NMR (600 MHz, $CDCl_3$) of *tert*-butyl (2*S*,3*S*,*E*)-2-(((*S*)-3,4-di-*tert*-butoxy-4-oxobutyl)amino)-3-hydroxyhex-4-enoate (**24**)

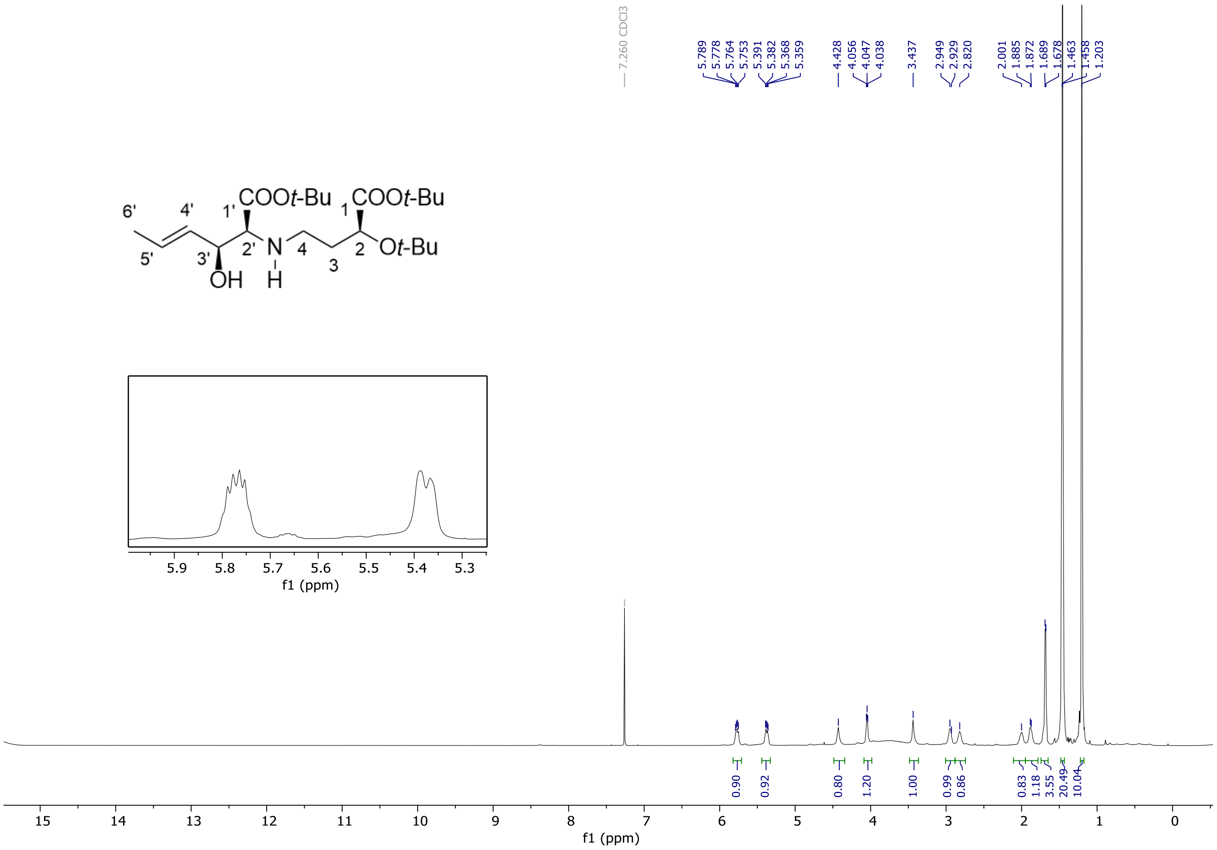

Figure 40: ^{13}C-NMR (151 MHz, $CDCl_3$) of *tert*-butyl (2*S*,3*S*,*E*)-2-(((*S*)-3,4-di-*tert*-butoxy-4-oxobutyl)amino)-3-hydroxyhex-4-enoate (**24**)

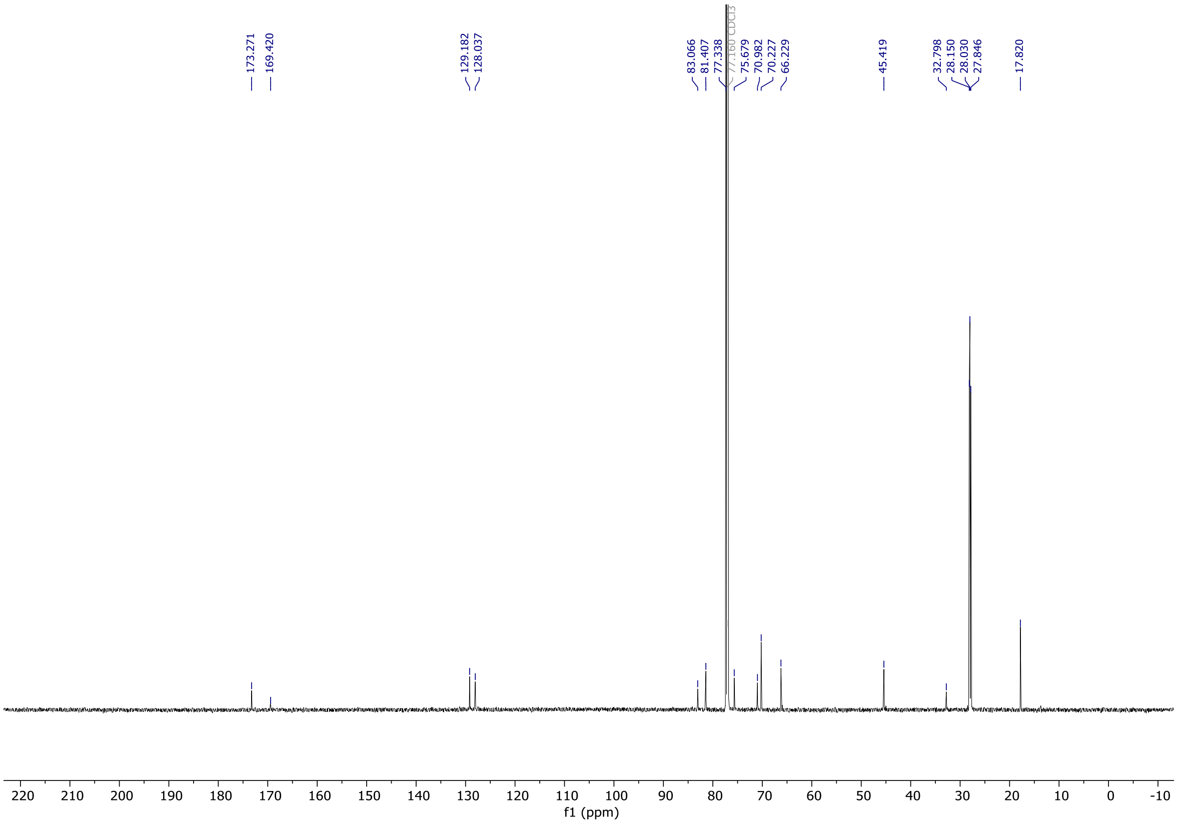

Figure 41: ^{1}H-NMR (600 MHz, $CDCl_3$) of *tert*-butyl (2*S*,3*S*,*E*)-2-((*tert*-butoxycarbonyl)((S)-3,4-di-*tert*-butoxy-4-oxobutyl) amino)-3-hydroxyhex-4-enoate (**25**)

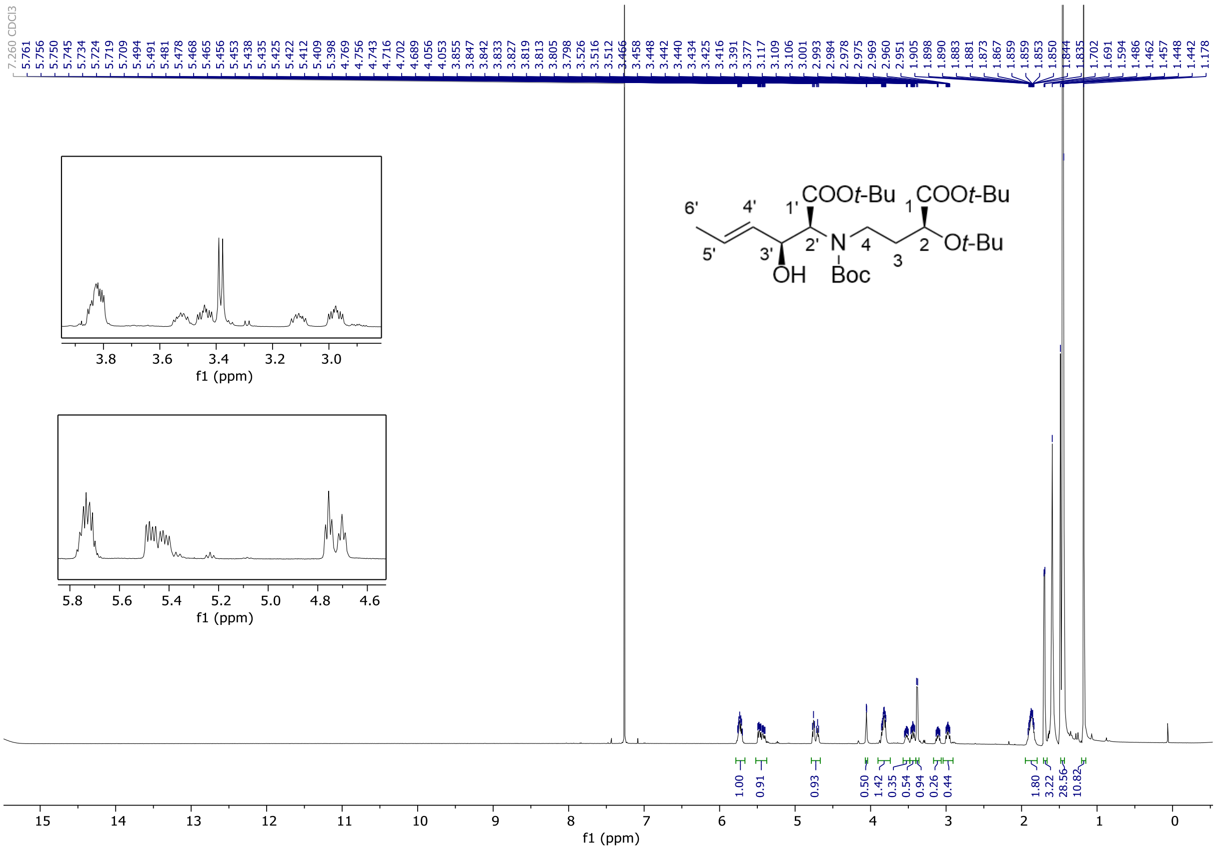

Figure 42: ^{13}C-NMR (151 MHz, $CDCl_3$) *tert*-butyl (2*S*,3*S*,*E*)-2-((*tert*-butoxycarbonyl)((S)-3,4-di-*tert*-butoxy-4-oxobutyl) amino)-3-hydroxyhex-4-enoate (**25**)

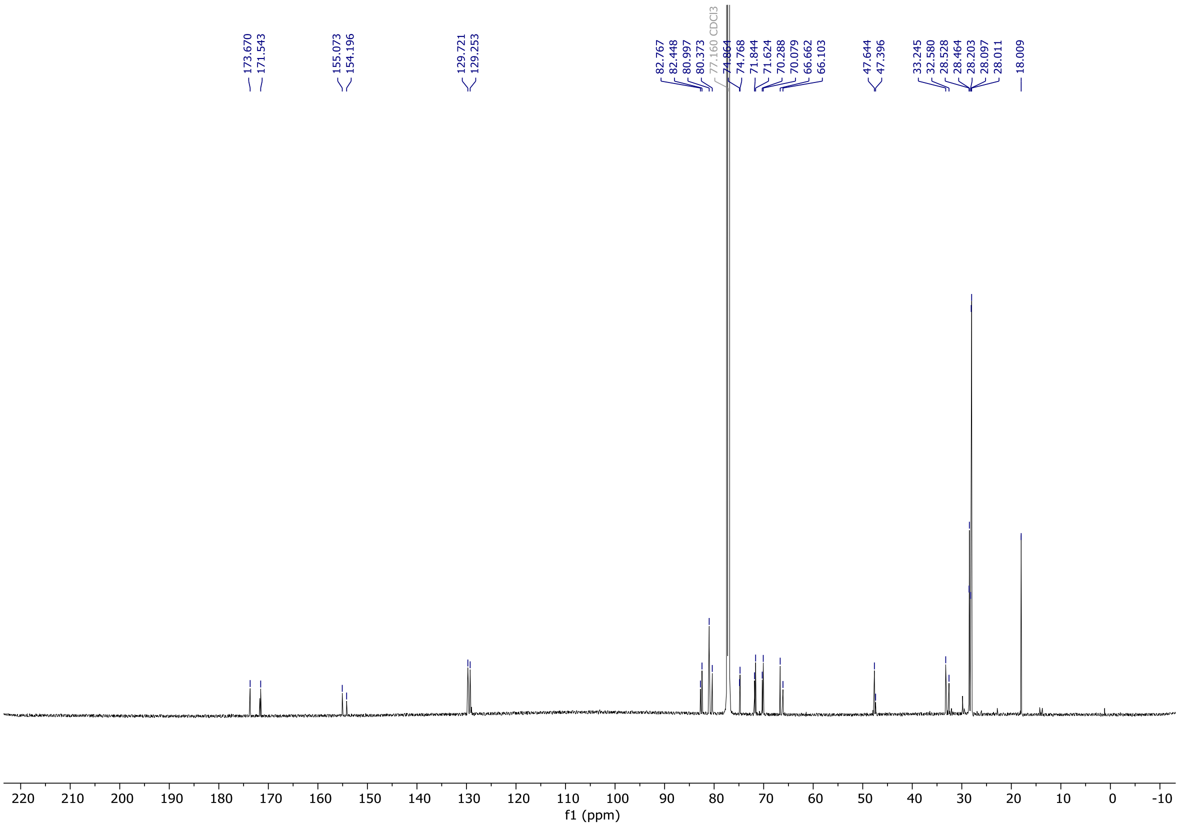

Figure 43: ^{1}H-NMR (600 MHz, $CDCl_3$) of *tert*-butyl (*S*)-2-(((*S*)-3,4-di-*tert*-butoxy-4-oxobutyl)amino)pent-4-enoate (**22**)

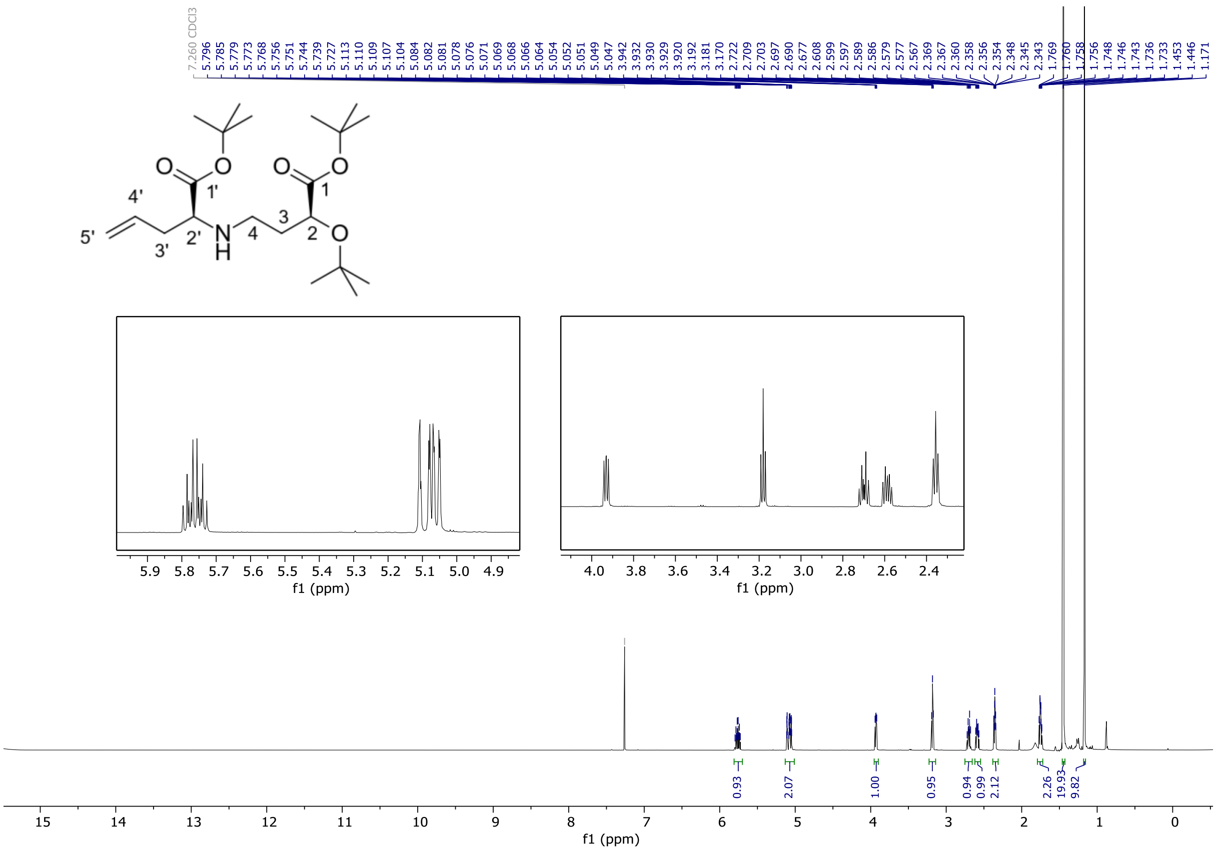

Figure 44: ^{13}C-DEPTQ (151 MHz, $CDCl_3$) of *tert*-butyl (*S*)-2-(((*S*)-3,4-di-*tert*-butoxy-4-oxobutyl)amino)pent-4-enoate (**22**)

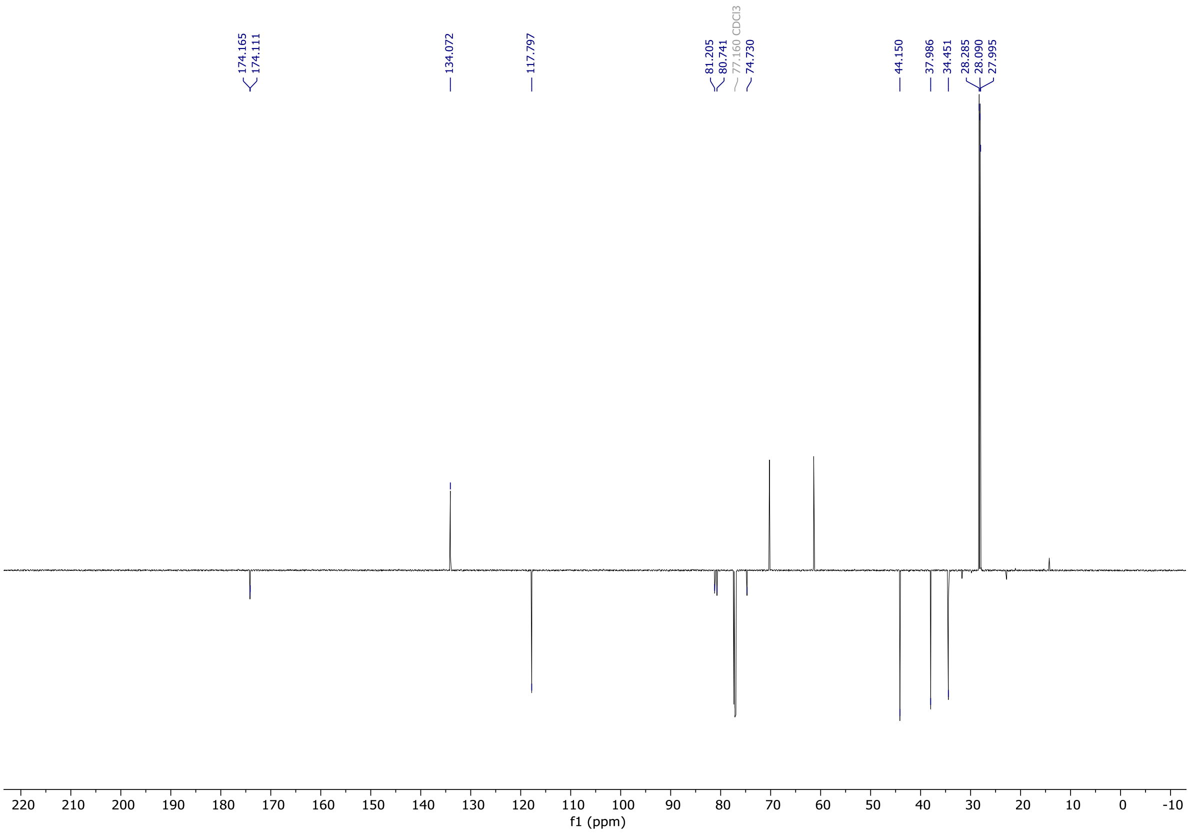

Figure 45: ^{1}H-NMR (600 MHz, $CDCl_3$) of *tert*-butyl (*S*)-2-((*tert*-butoxycarbonyl)((*S*)-3,4-di-*tert*-butoxy-4-oxobutyl)amino) pent-4-enoate (**23**)

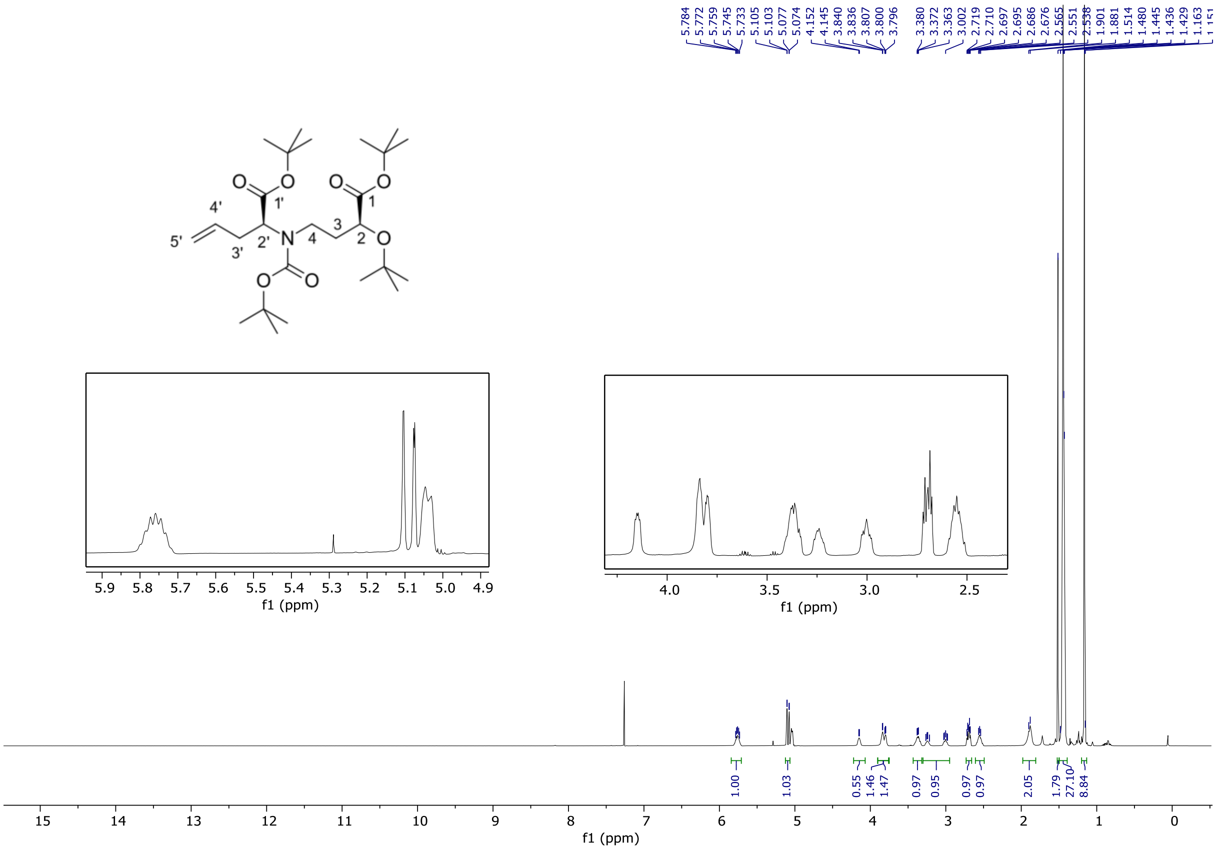

Figure 46: ^{13}C-DEPTQ (151 MHz, $CDCl_3$) of *tert*-butyl (*S*)-2-((*tert*-butoxycarbonyl)((*S*)-3,4-di-*tert*-butoxy-4-oxobutyl)amino) pent-4-enoate (**23**)

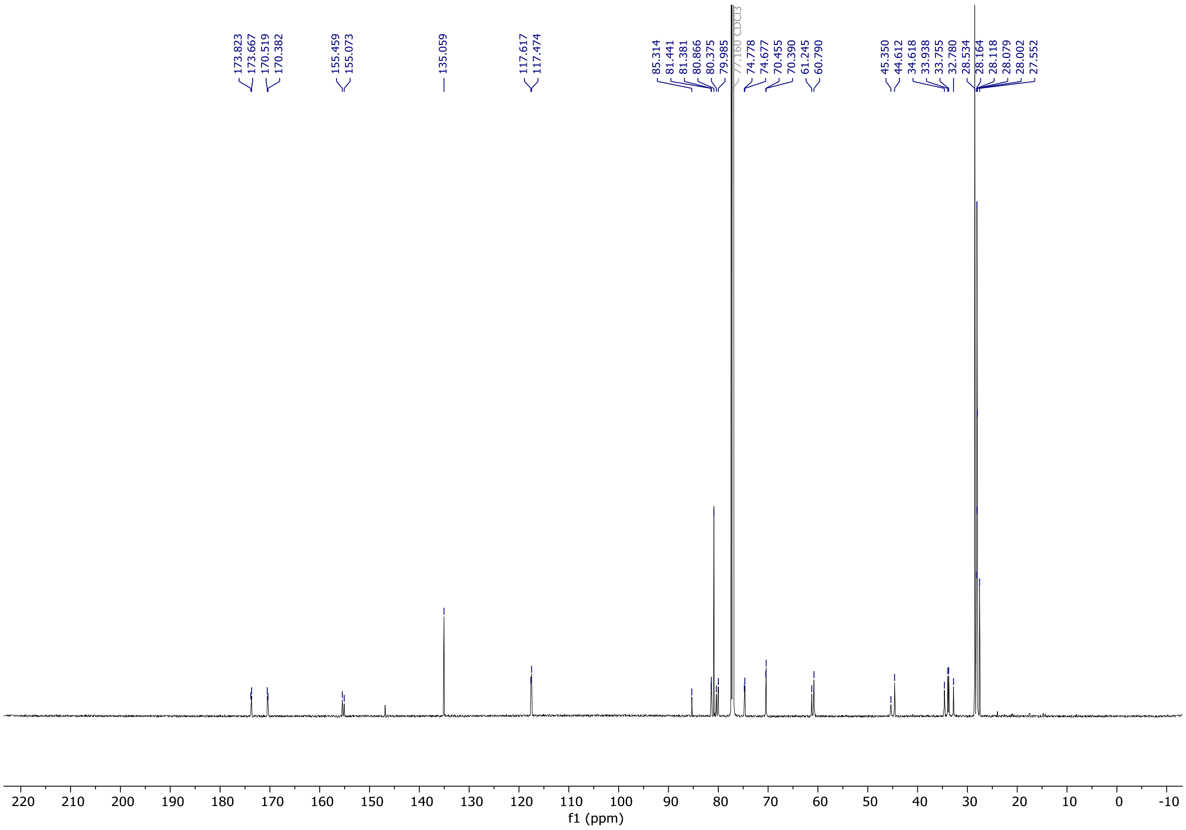

Figure 47: ^{1}H-NMR (600 MHz, CD_3OD) of (*S*)-1-((2*S*,3*S*)-4-(*tert*-butoxy)-3-((*tert*-butoxycarbonyl)((*S*)-3,4-di-*tert*-butoxy-4-oxobutyl)amino)-2-hydroxy-4-oxobutyl)azetidine-2-carboxylic acid (**33**)

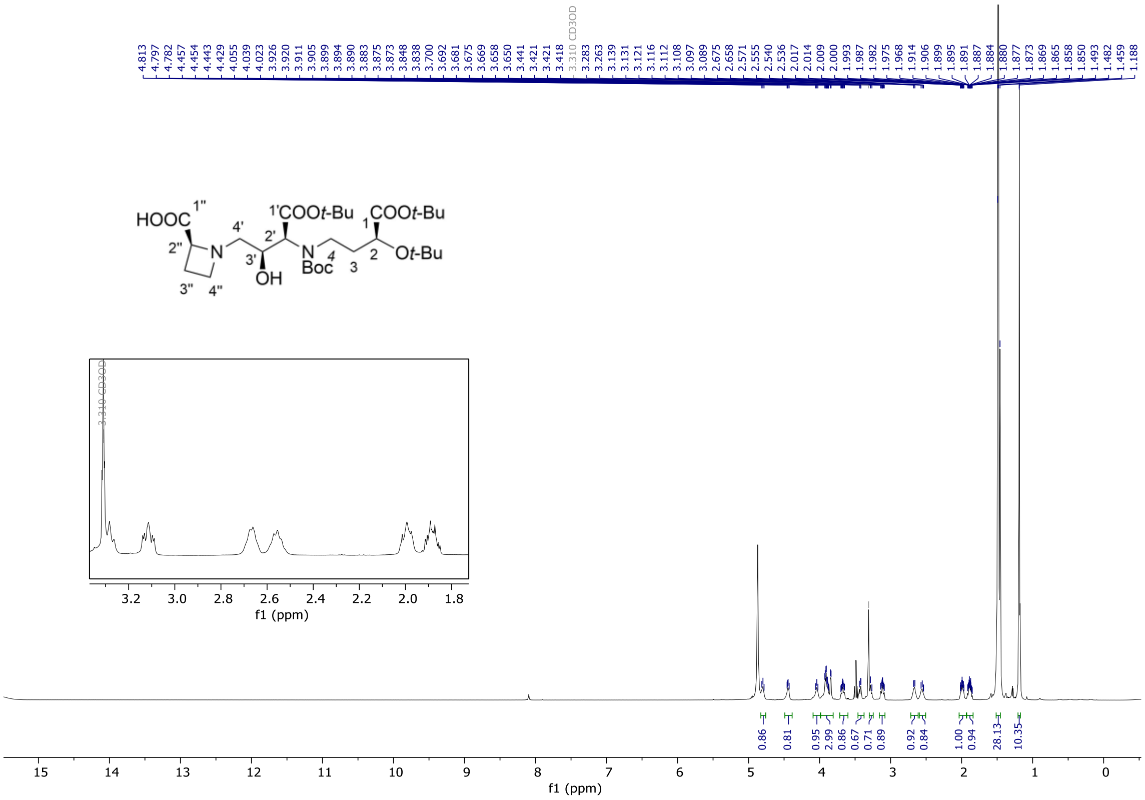

Figure 48: ^{13}C-NMR (151 MHz, CD_3OD) of (*S*)-1-((2*S*,3*S*)-4-(*tert*-butoxy)-3-((*tert*-butoxycarbonyl)((*S*)-3,4-di-*tert*-butoxy-4-oxobutyl)amino)-2-hydroxy-4-oxobutyl)azetidine-2-carboxylic acid (**33**)

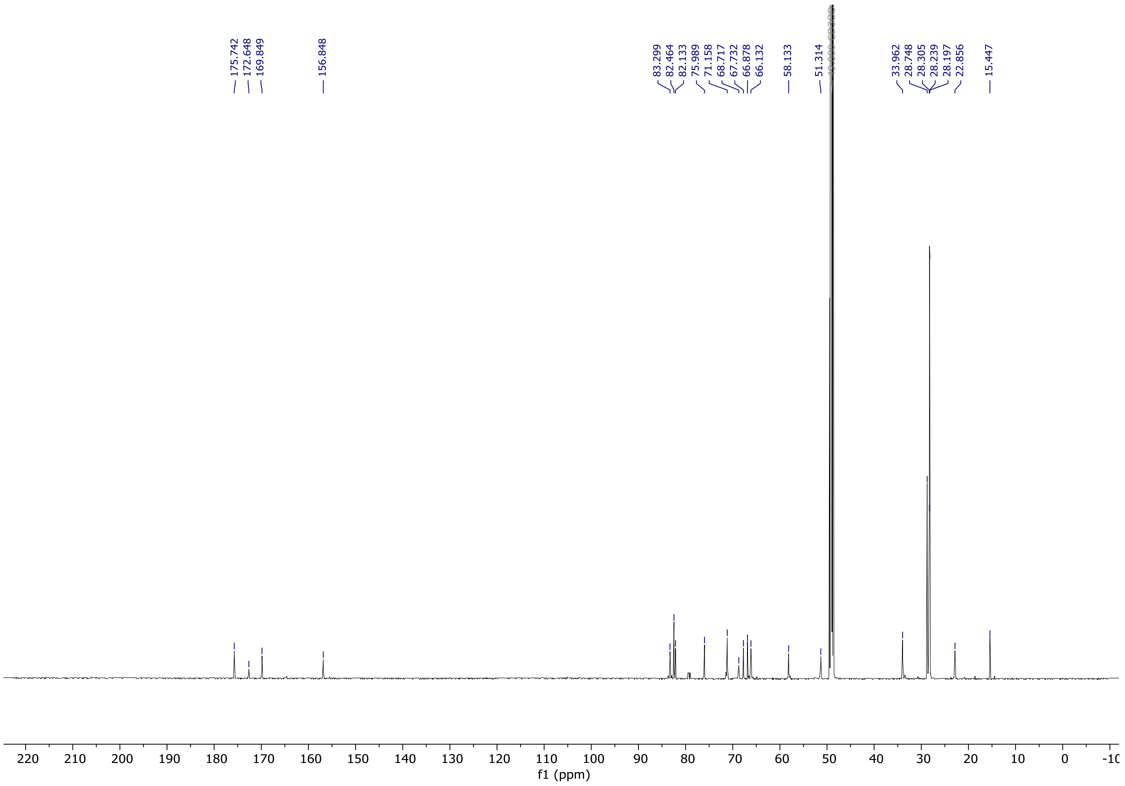

Figure 49: ^{1}H-NMR (600 MHz, CD_3OD) of (*S*)-1-((2*S*,3*S*)-4-(*tert*-butoxy)-3-((*tert*-butoxycarbonyl)((*S*)-3,4-di-*tert*-butoxy-4-oxobutyl)amino)-2-hydroxy-4-oxobutyl)azetidine-2-carboxylic acid (**31**)

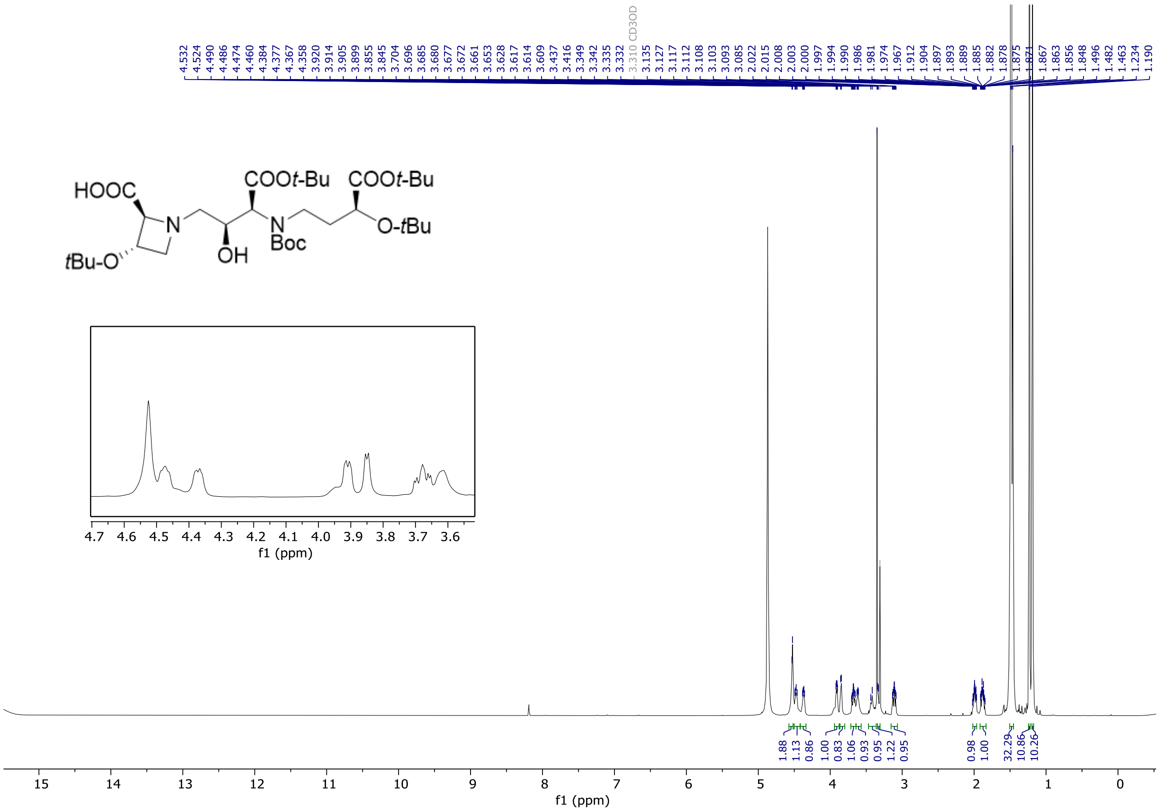

Figure 50: ^{13}C-NMR (151 MHz, CD_3OD) of (*S*)-1-((2*S*,3*S*)-4-(*tert*-butoxy)-3-((*tert*-butoxycarbonyl)((*S*)-3,4-di-*tert*-butoxy-4-oxobutyl)amino)-2-hydroxy-4-oxobutyl)azetidine-2-carboxylic acid (**31**)

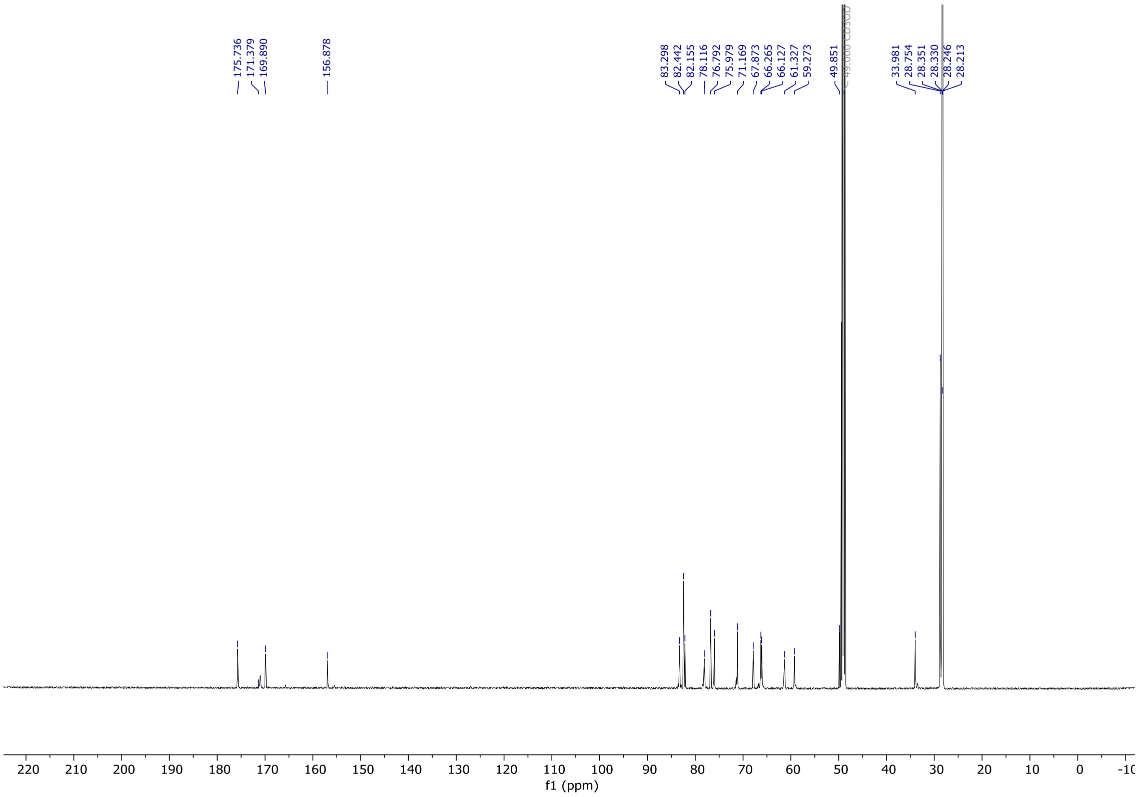

Figure 51: ^{1}H-NMR (600 MHz, $CDCl_3$) of (*S*)-1-((2*S*,3*R*)-4-(*tert*-butoxy)-3-((*tert*-butoxycarbonyl)((*S*)-3,4-di-*tert*-butoxy-4-oxobutyl)amino)-2-hydroxy-4-oxobutyl)azetidine-2-carboxylic acid (**32**)

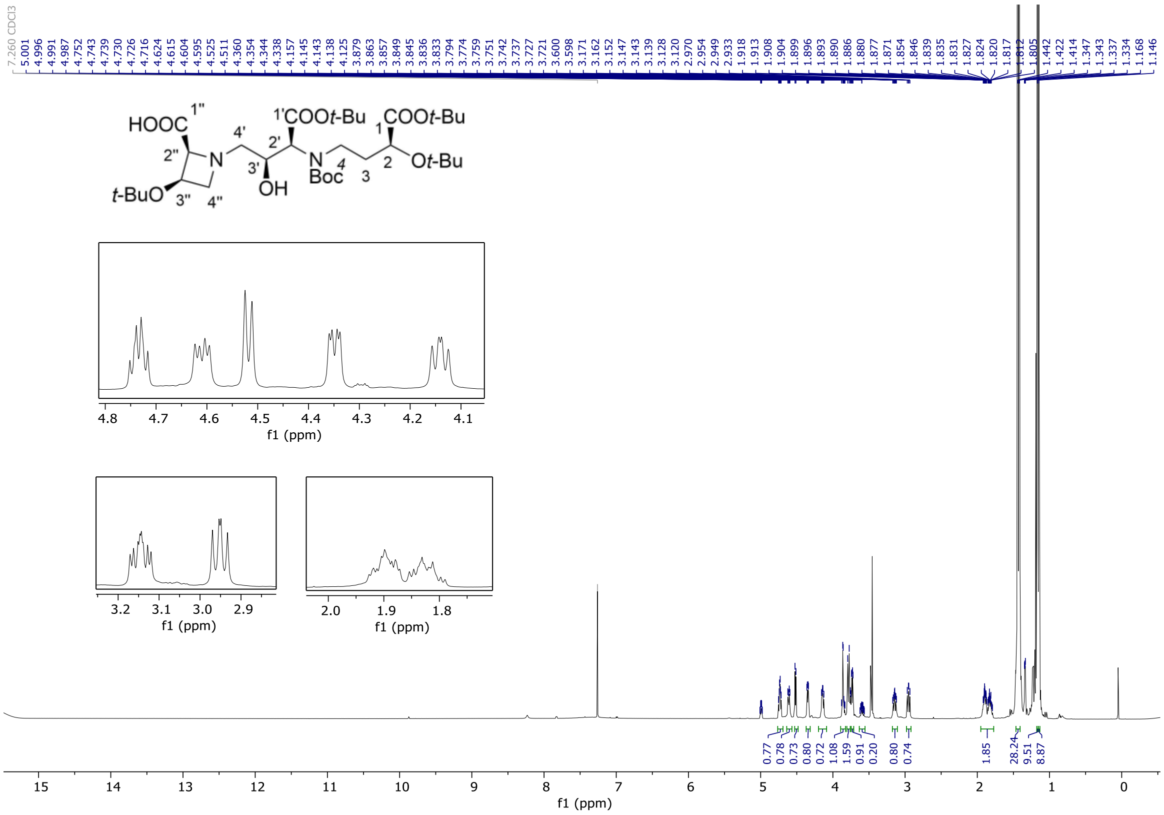

Figure 52: ^{13}C-DEPTQ (151 MHz, $CDCl_3$) of (*S*)-1-((2*S*,3*R*)-4-(*tert*-butoxy)-3-((*tert*-butoxycarbonyl)((*S*)-3,4-di-*tert*-butoxy-4-oxobutyl)amino)-2-hydroxy-4-oxobutyl)azetidine-2-carboxylic acid (**32**)

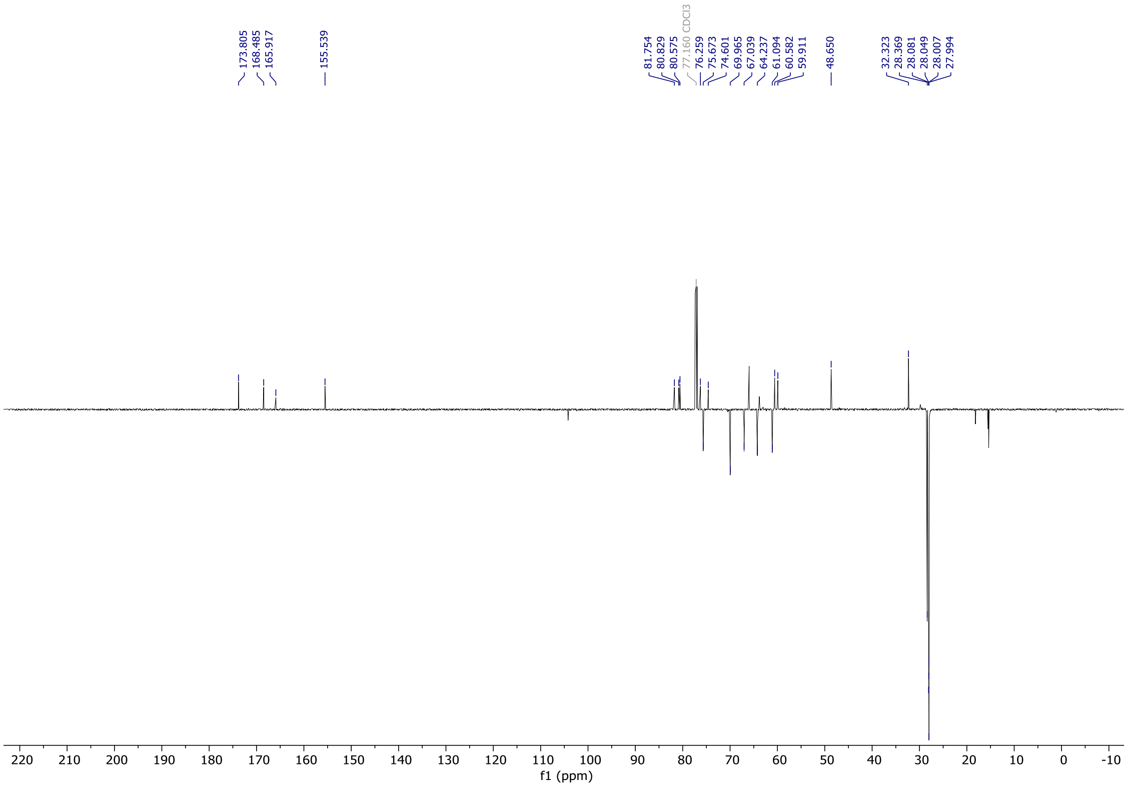

Figure 53: ^{1}H-NMR (600 MHz, CD_3OD) of *tert*-butyl (2*S*,3*S*)-2-((*tert*-butoxycarbonyl)((*S*)-3,4-di-*tert*-butoxy-4-oxobutyl) amino)-3-hydroxy-4-(((*S*)-2-oxotetrahydrofuran-3-yl)amino)butanoate (**44**)

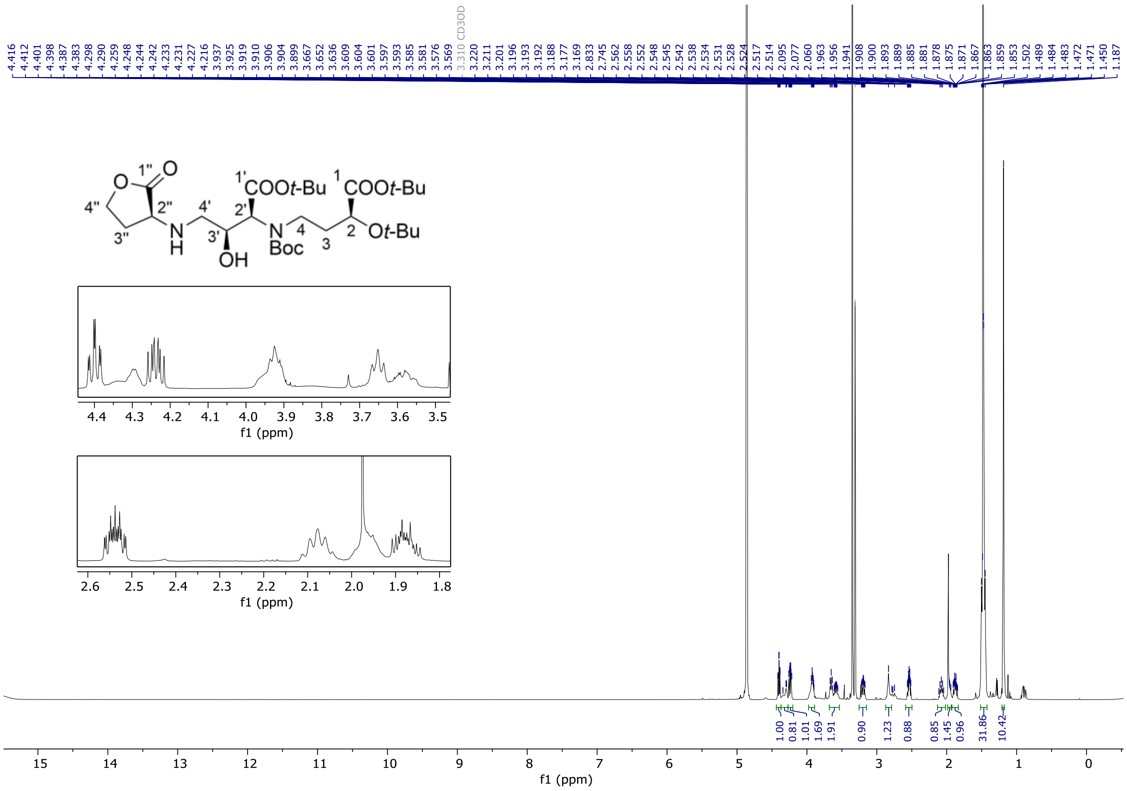

Figure 54: ^{13}C-NMR (151 MHz, CD_3OD) of *tert*-butyl (2*S*,3*S*)-2-((*tert*-butoxycarbonyl)((*S*)-3,4-di-*tert*-butoxy-4-oxobutyl) amino)-3-hydroxy-4-(((*S*)-2-oxotetrahydrofuran-3-yl)amino)butanoate (**44**)

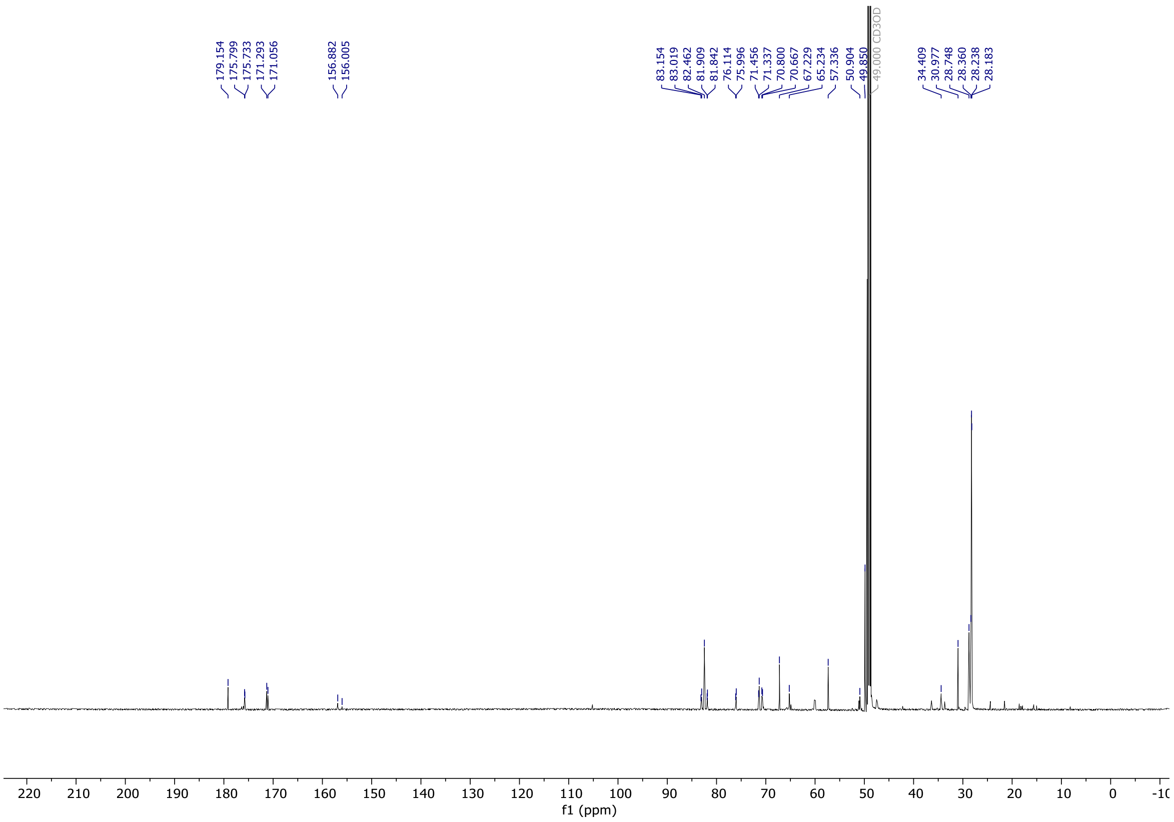

Figure 55: ^{1}H-NMR (600 MHz, $CDCl_3$) of (*S*)-1-((*S*)-4-(*tert*-butoxy)-3-((*tert*-butoxycarbonyl)((*S*)-3,4-di-*tert*-butoxy-4-oxobutyl)amino)-4-oxobutyl)azetidine-2-carboxylic acid (**30**)

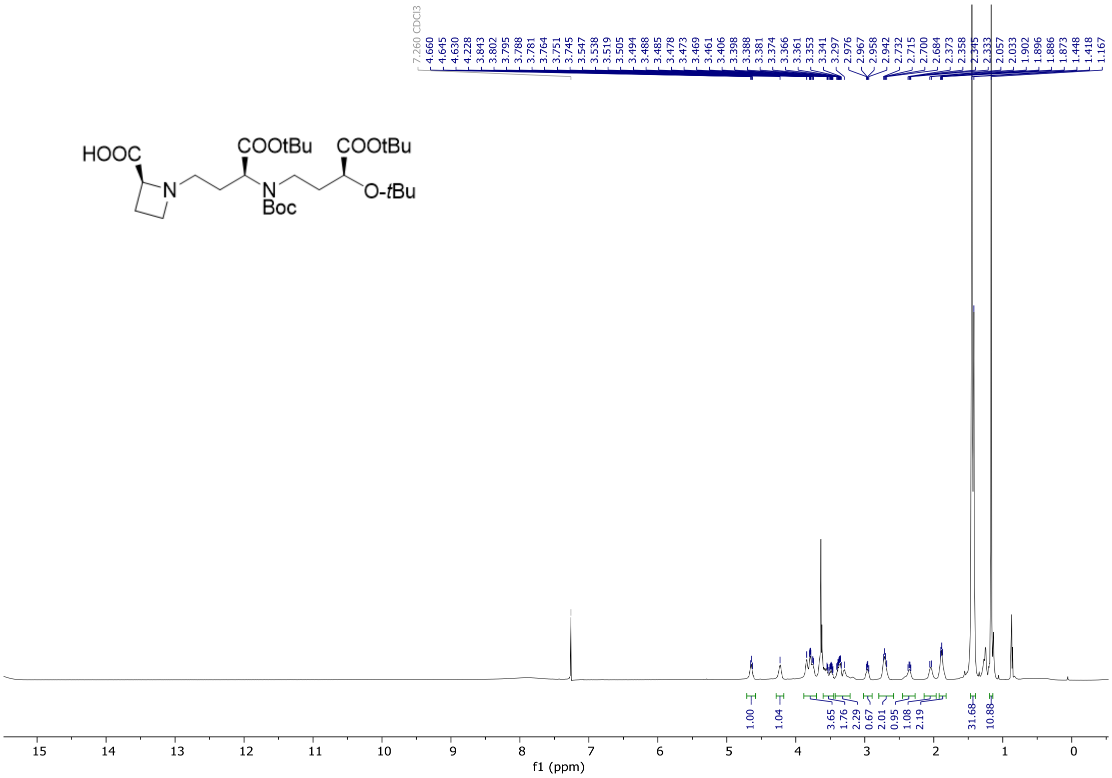

Figure 56: ^{13}C-NMR (151 MHz, $CDCl_3$) of (*S*)-1-((*S*)-4-(*tert*-butoxy)-3-((*tert*-butoxycarbonyl)((*S*)-3,4-di-*tert*-butoxy-4-oxobutyl)amino)-4-oxobutyl)azetidine-2-carboxylic acid (**30**)

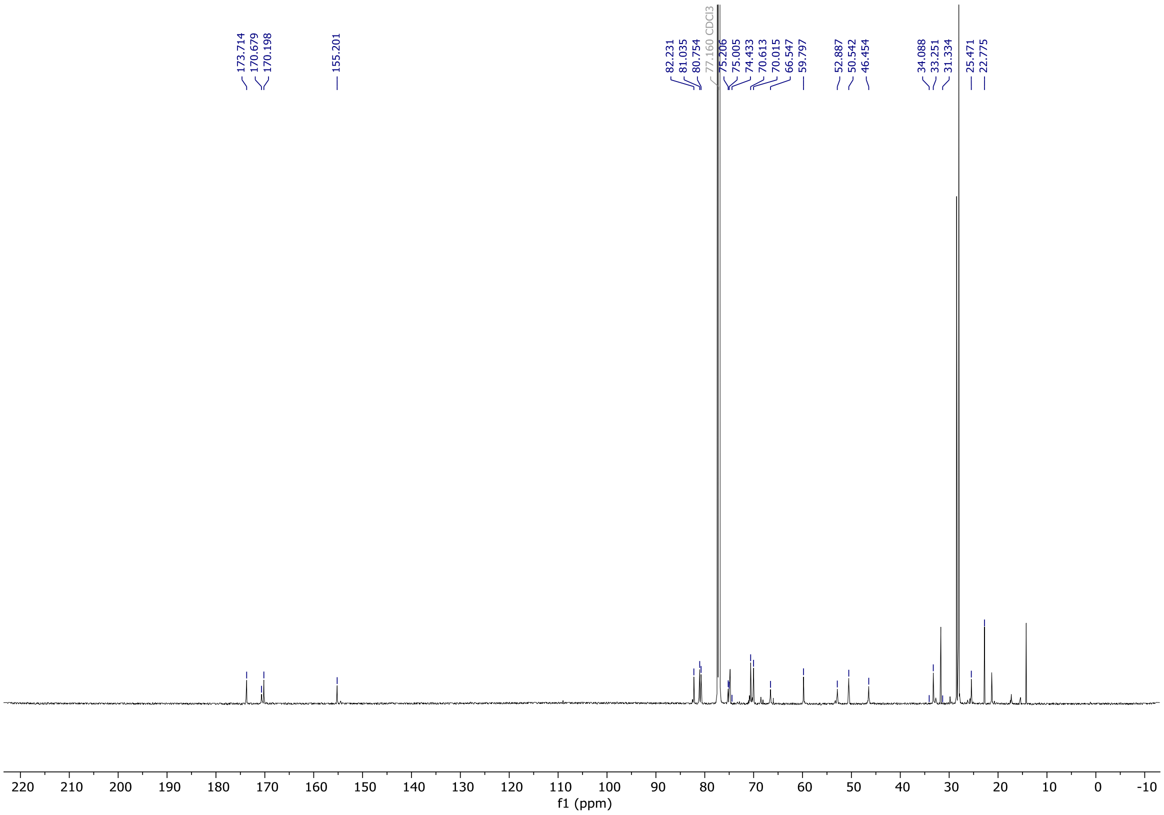

Figure 57: ^{1}H-NMR (600 MHz, CD_3OD) of (2*S*,3*S*)-3-(*tert*-butoxy)-1-((*S*)-4-(*tert*-butoxy)-3-((*tert*-butoxycarbonyl)((*S*)-3,4-di-*tert*-butoxy-4-oxobutyl)amino)-4-oxobutyl)azetidine-2-carboxylic acid (**28**)

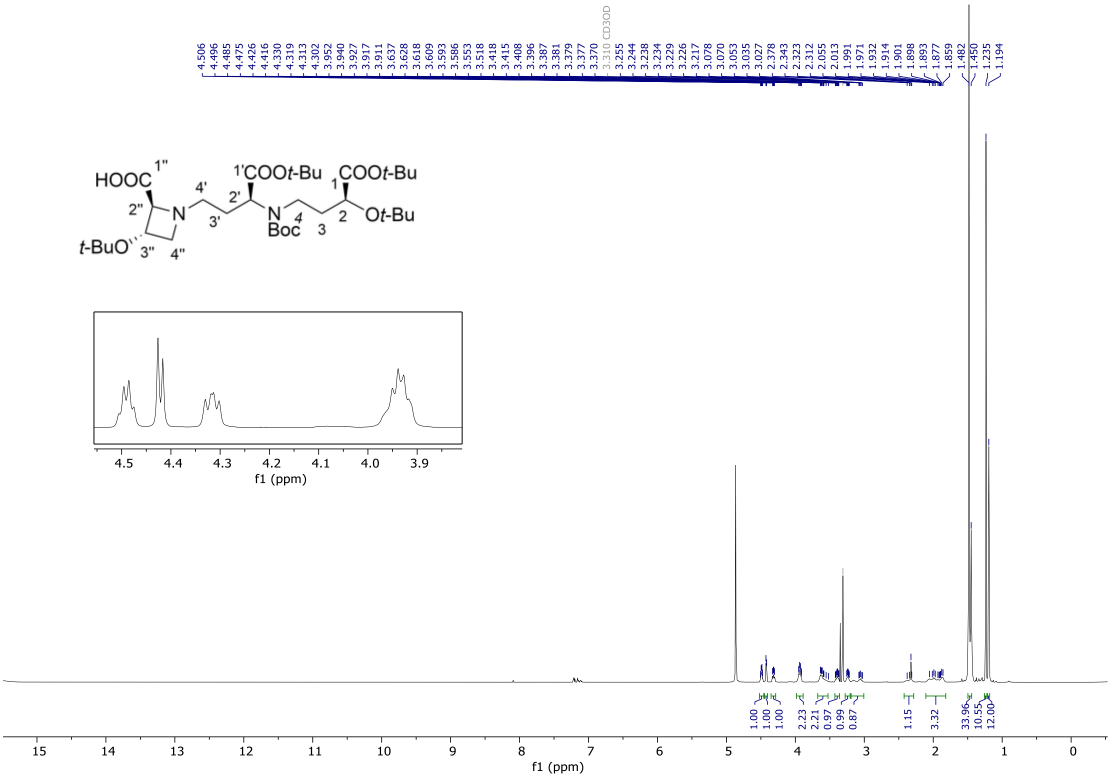

Figure 58: ^{13}C-NMR (151 MHz, CD_3OD) of (2*S*,3*S*)-3-(*tert*-butoxy)-1-((*S*)-4-(*tert*-butoxy)-3-((*tert*-butoxycarbonyl)((*S*)-3,4-di-*tert*-butoxy-4-oxobutyl)amino)-4-oxobutyl)azetidine-2-carboxylic acid (**28**)

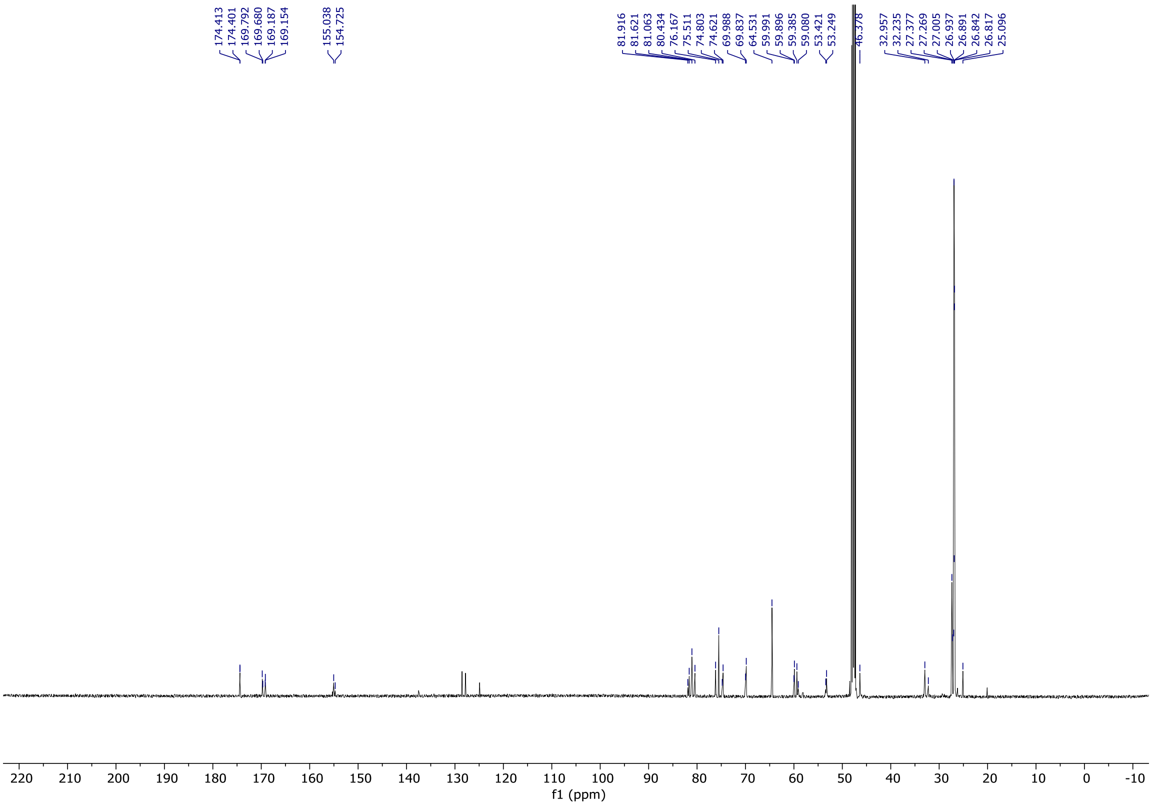

Figure 59: ^{1}H-NMR (600 MHz, CD_3OD) of (2*S*,3*R*)-3-(*tert*-butoxy)-1-((*S*)-4-(*tert*-butoxy)-3-((*tert*-butoxycarbonyl)((S)-3,4-di-*tert*-butoxy-4-oxobutyl)amino)-4-oxobutyl)azetidine-2-carboxylic acid (**29**)

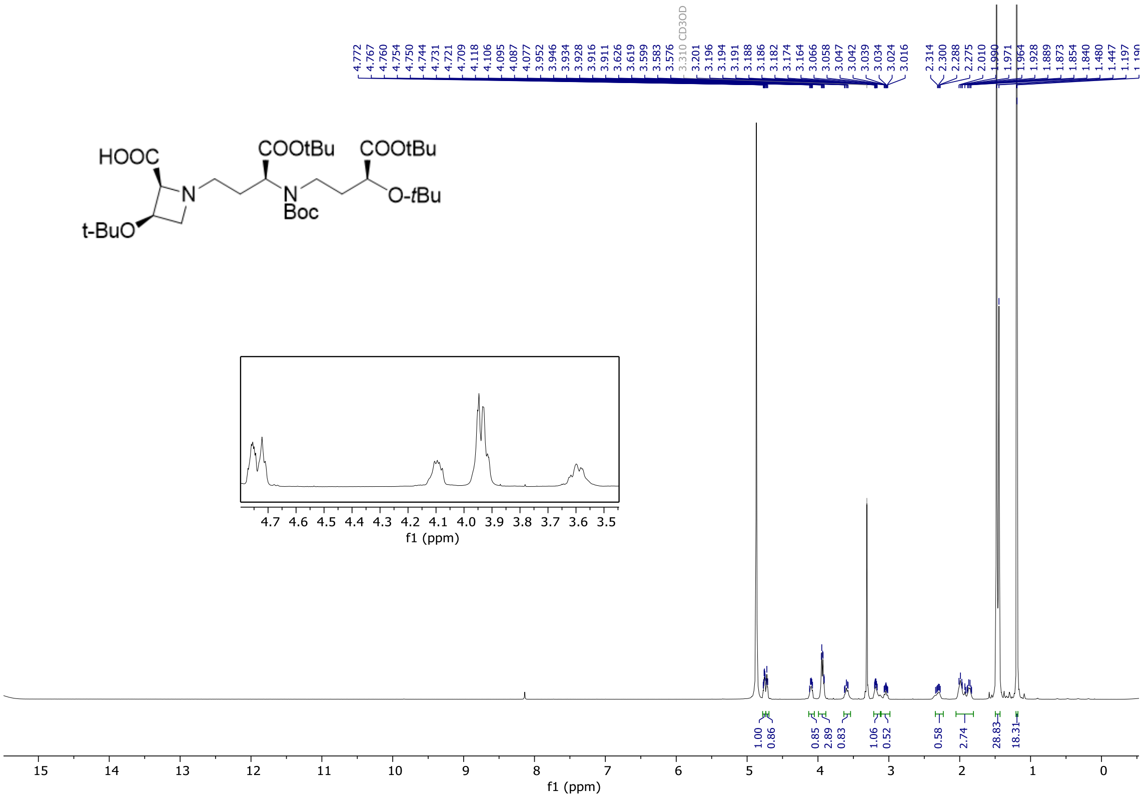

Figure 60: ^{13}C-NMR (151 MHz, CD_3OD) of (2*S*,3*R*)-3-(*tert*-butoxy)-1-((*S*)-4-(*tert*-butoxy)-3-((*tert*-butoxycarbonyl)((S)-3,4-di-*tert*-butoxy-4-oxobutyl)amino)-4-oxobutyl)azetidine-2-carboxylic acid (**29**)

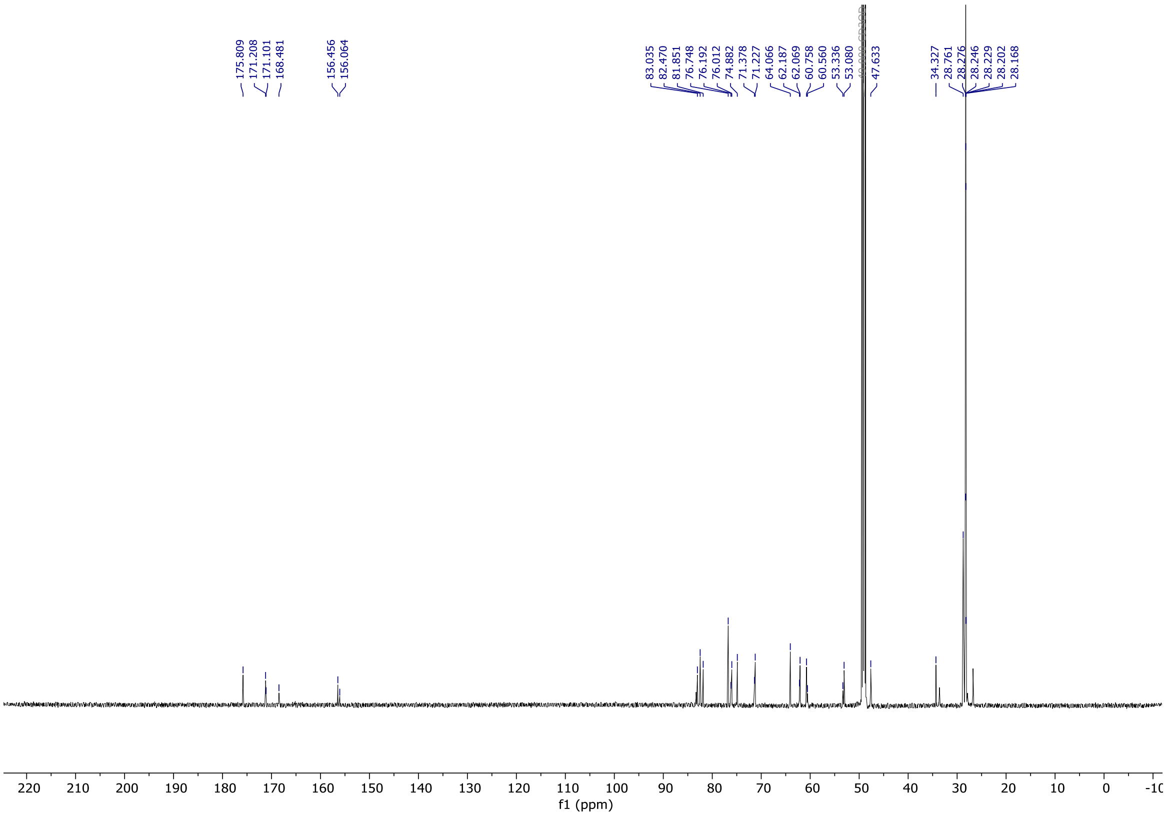

Figure 61: ^{1}H-NMR (600 MHz, $CDCl_3$) of *tert*-butyl (*S*)-2-(*tert*-butoxy)-4-(((*S*)-1-(*tert*-butoxy)-1-oxo-4-(((*S*)-2-oxotetra-hydrofuran-3-yl)amino)butan-2-yl)(*tert*-butoxycarbonyl) amino)butanoate (**45**)

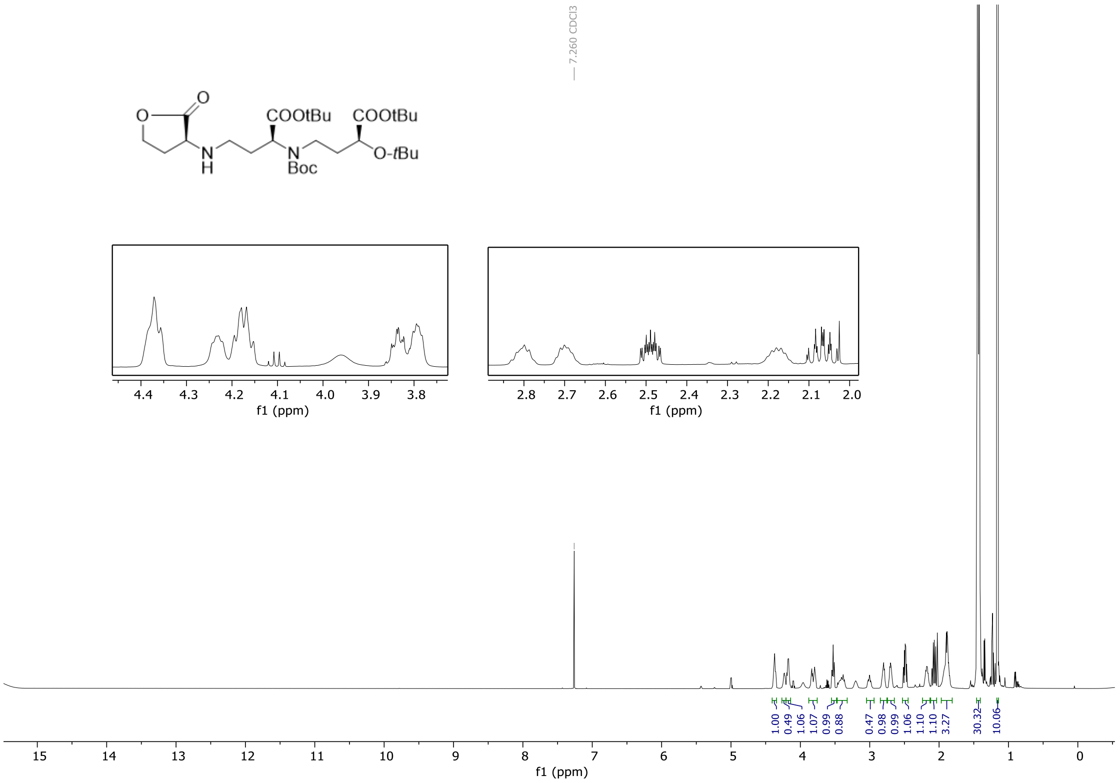

Figure 62: ^{13}C-NMR (151 MHz, $CDCl_3$) of *tert*-butyl (*S*)-2-(*tert*-butoxy)-4-(((*S*)-1-(*tert*-butoxy)-1-oxo-4-(((*S*)-2-oxotetra-hydrofuran-3-yl)amino)butan-2-yl)(*tert*-butoxycarbonyl) amino)butanoate (**45**)

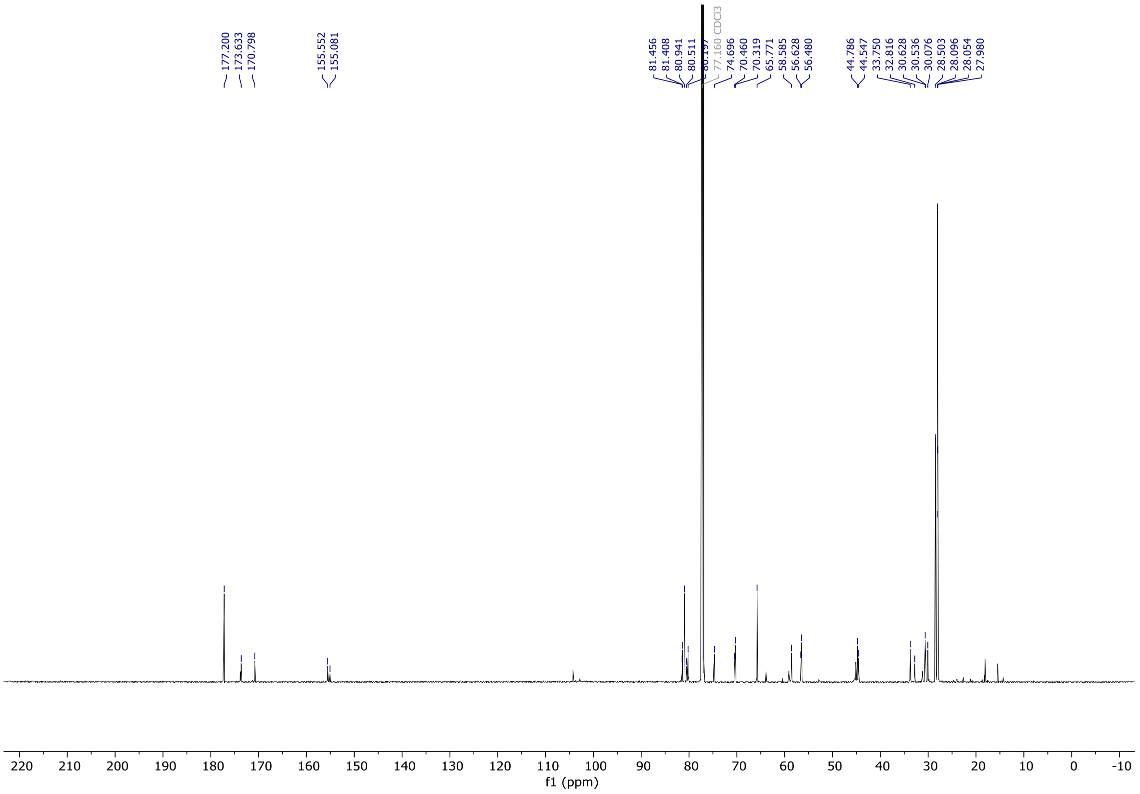

Figure 63: ^{1}H-NMR (600 MHz, D_2O) of mugineic acid (**VIII**)

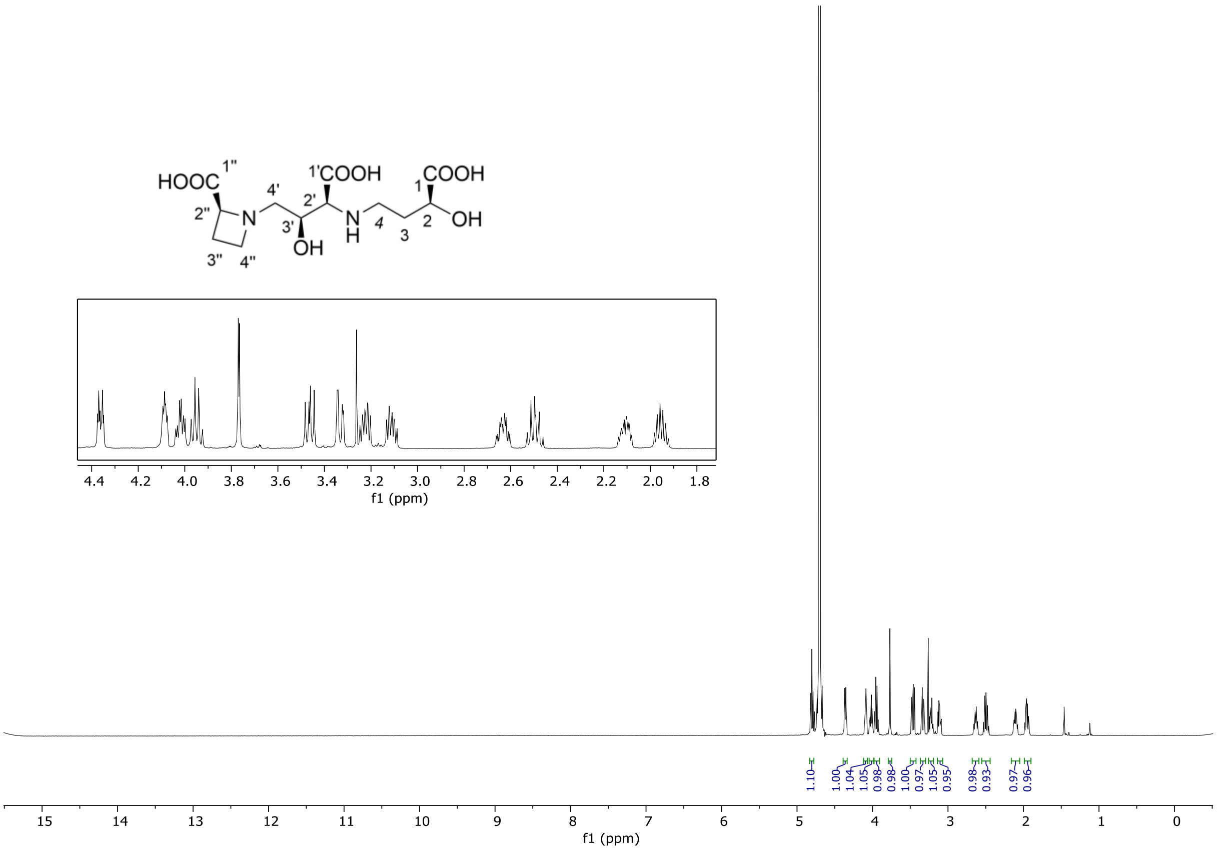

Figure 64: ^{13}C-NMR (151 MHz, D_2O) of mugineic acid (**VIII**)

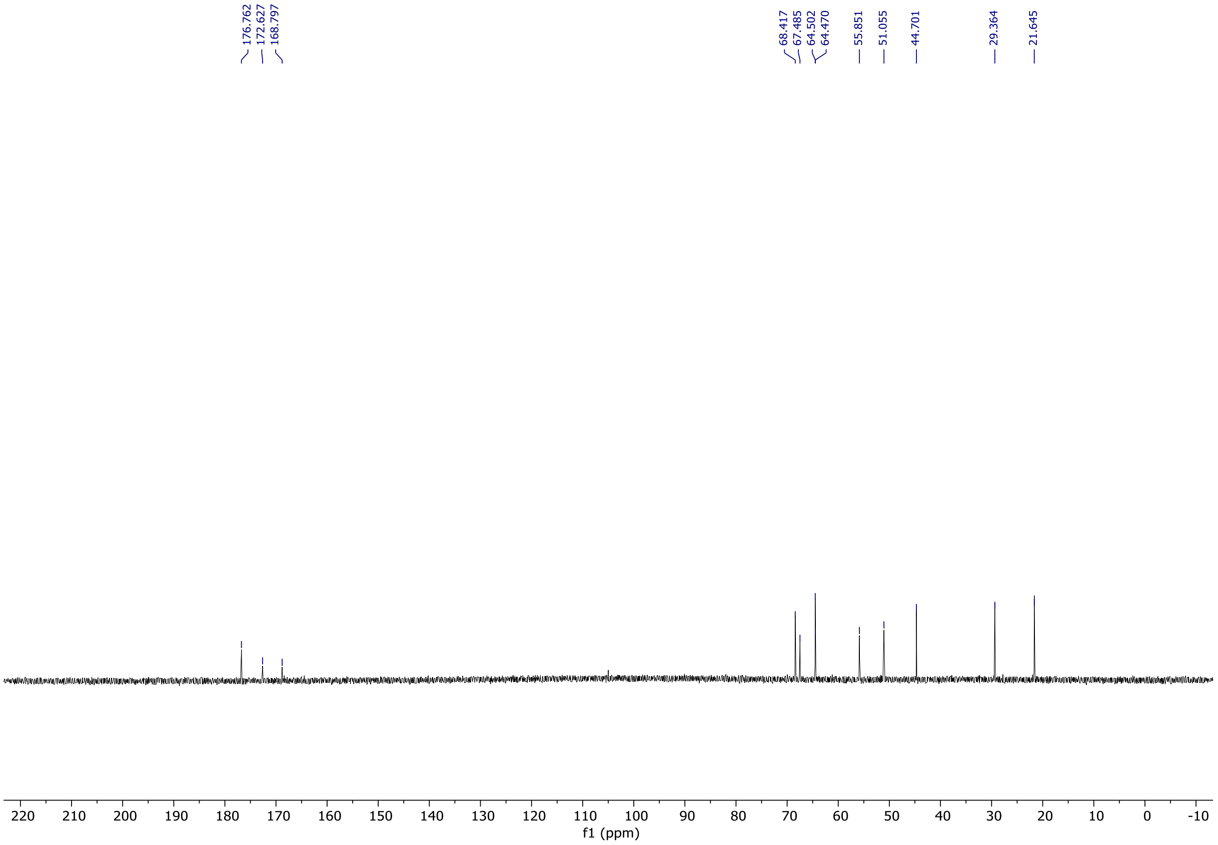

Figure 65: ^{1}H-NMR (600 MHz, D_2O) of 3’’-*epi*-hydroxy mugineic acid (**VI**)

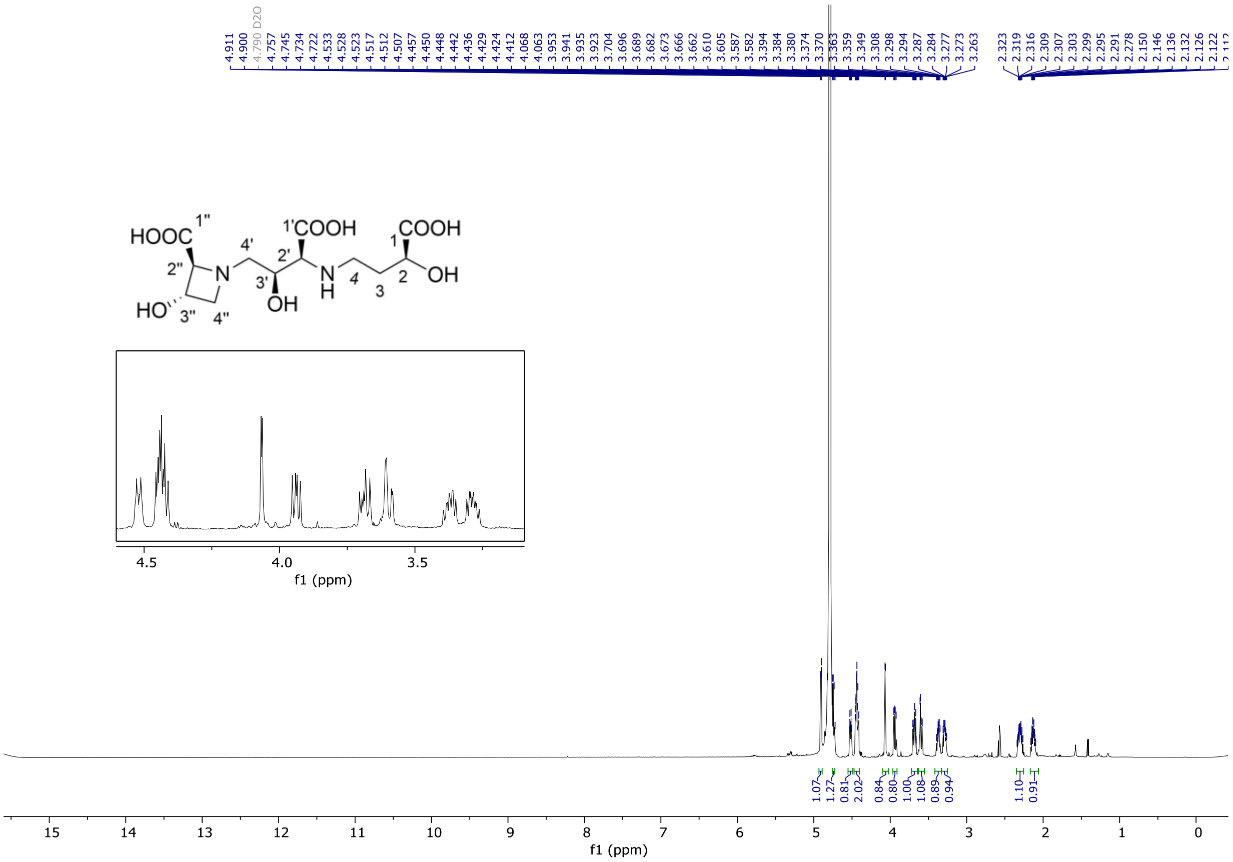

Figure 66: ^{13}C-NMR (151 MHz, D_2O) of 3’’-*epi*-hydroxy mugineic acid (**VI**)

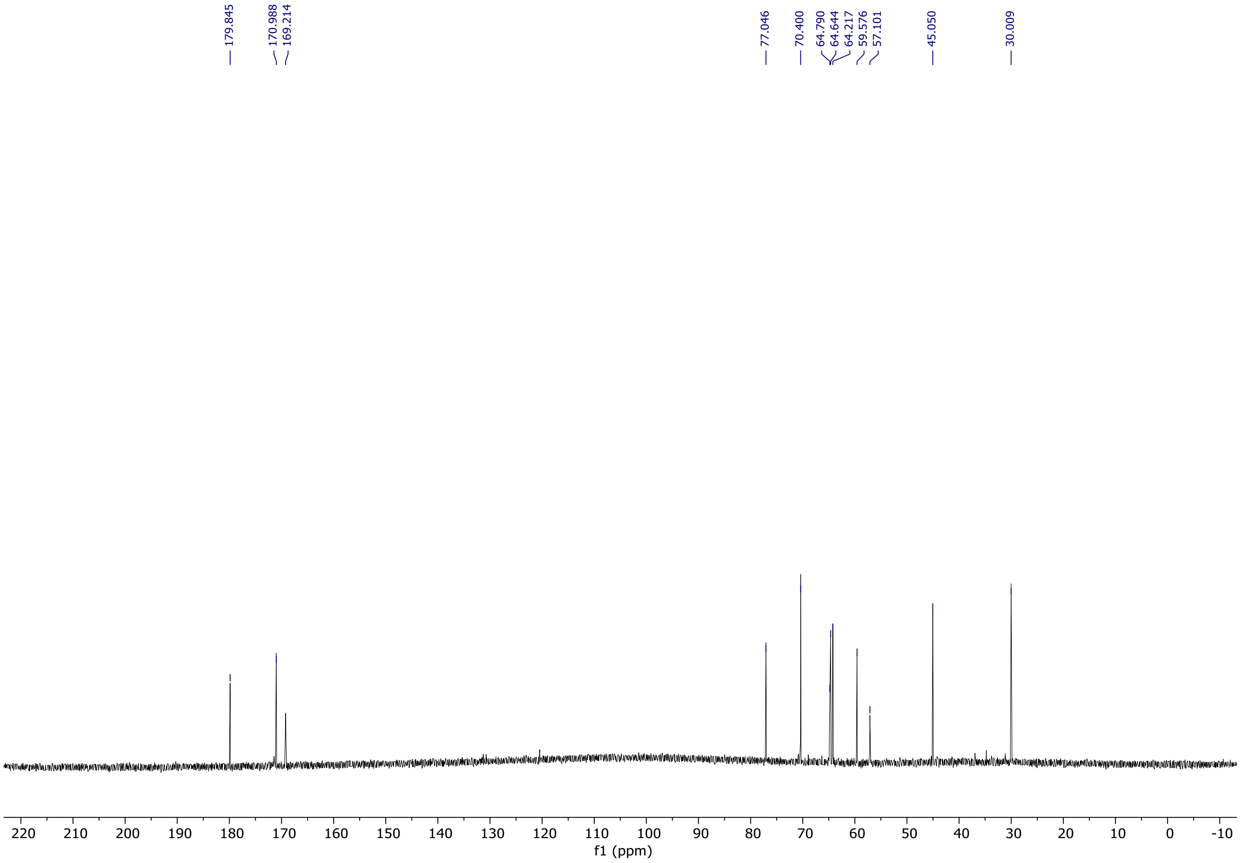

Figure 67: ^{1}H-NMR (600 MHz, D_2O) of 3’’-hydroxy mugineic acid (**VII**)

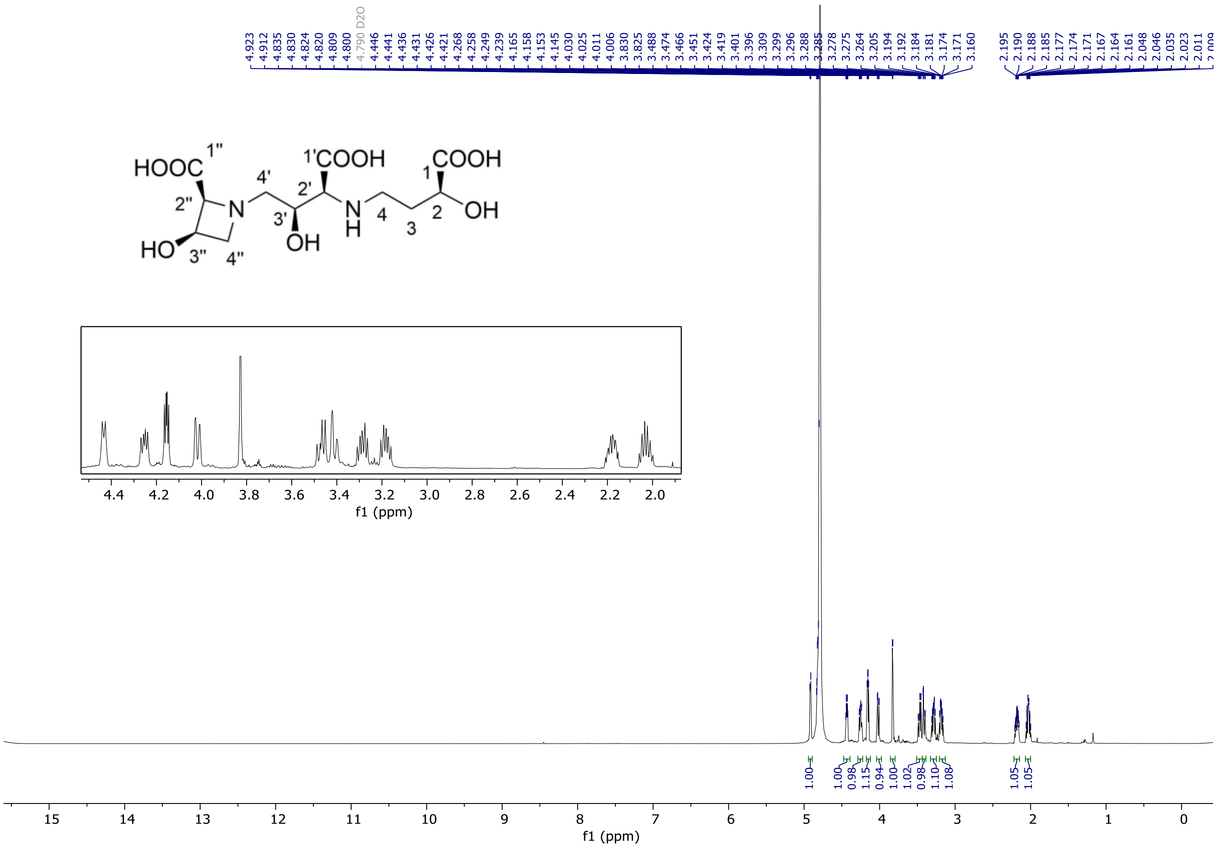

Figure 68: ^{13}C-DEPTQ (151 MHz, D_2O) of 3’’-hydroxy mugineic acid (**VII**)

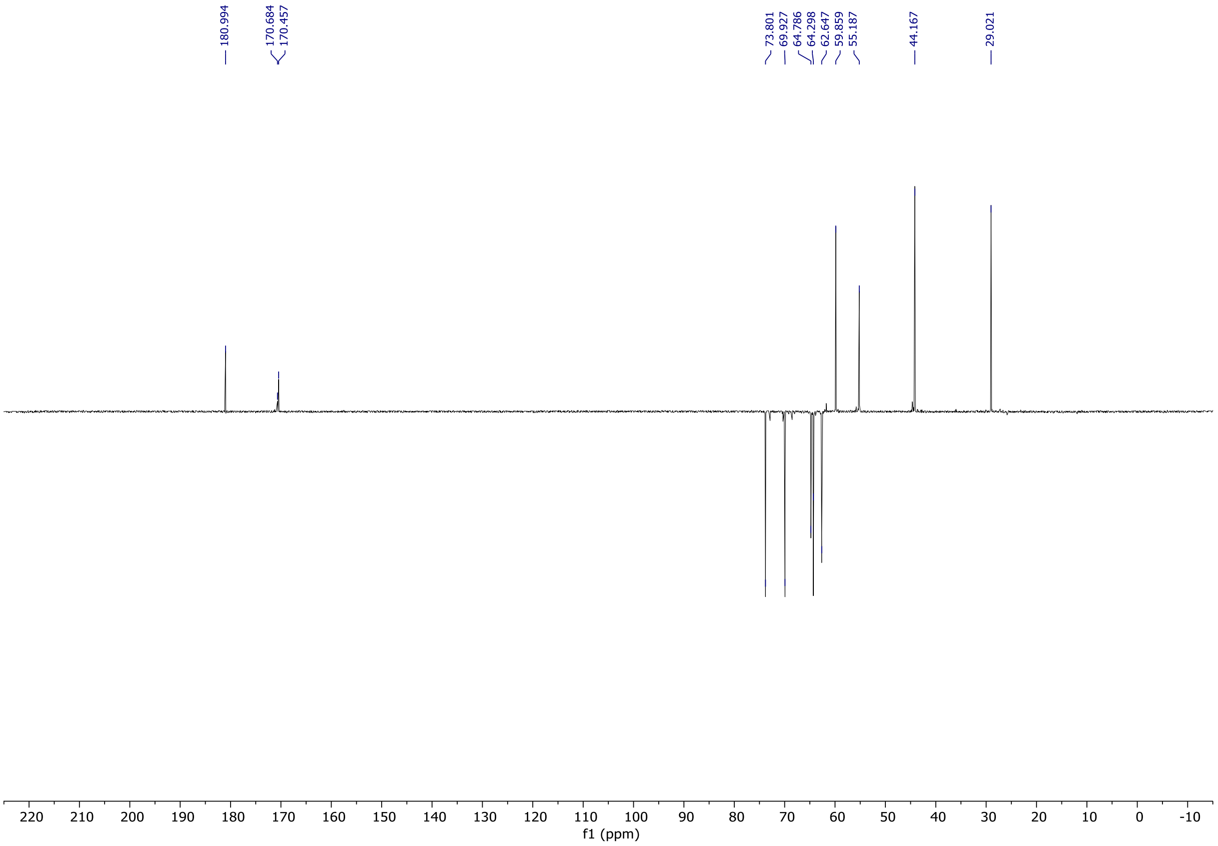

Figure 69: ^{1}H-NMR (600 MHz, D_2O) of (2*S*,3*S*)-2-(((*S*)-3-carboxy-3-hydroxypropyl)amino)-3-hydroxy-4-(((*S*)-2-oxotetrahydrofuran-3-yl)amino)butanoic acid (**46**)

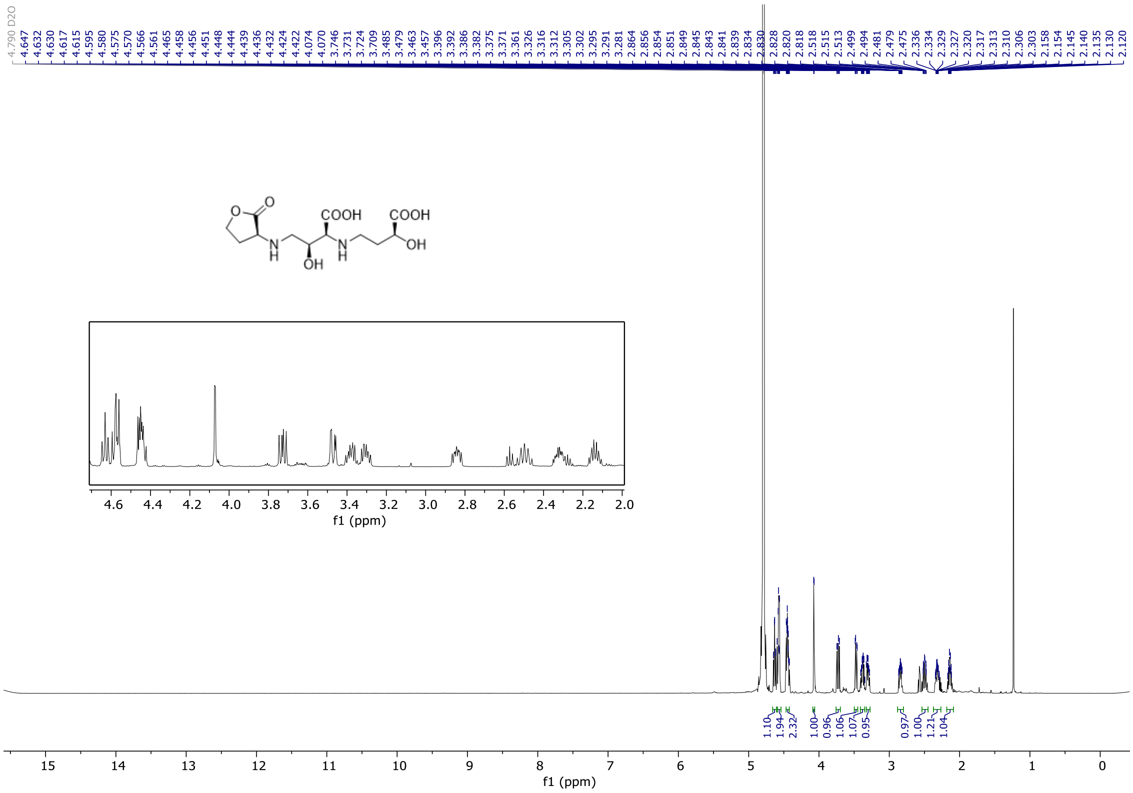

Figure 70: ^{13}C-NMR (151 MHz, D_2O) of (2*S*,3*S*)-2-(((*S*)-3-carboxy-3-hydroxypropyl)amino)-3-hydroxy-4-(((*S*)-2-oxotetrahydrofuran-3-yl)amino)butanoic acid (**46**)

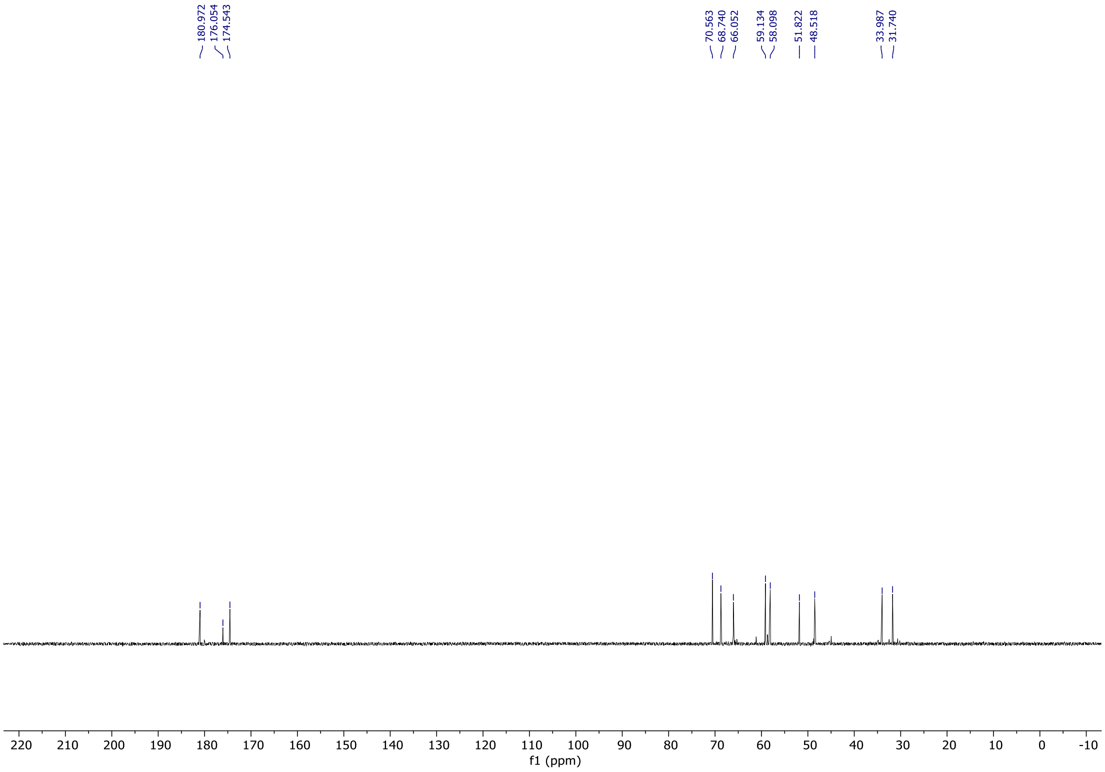

Figure 71: ^{1}H-NMR (600 MHz, D_2O) of hydroxyavenic acid A (**V**)

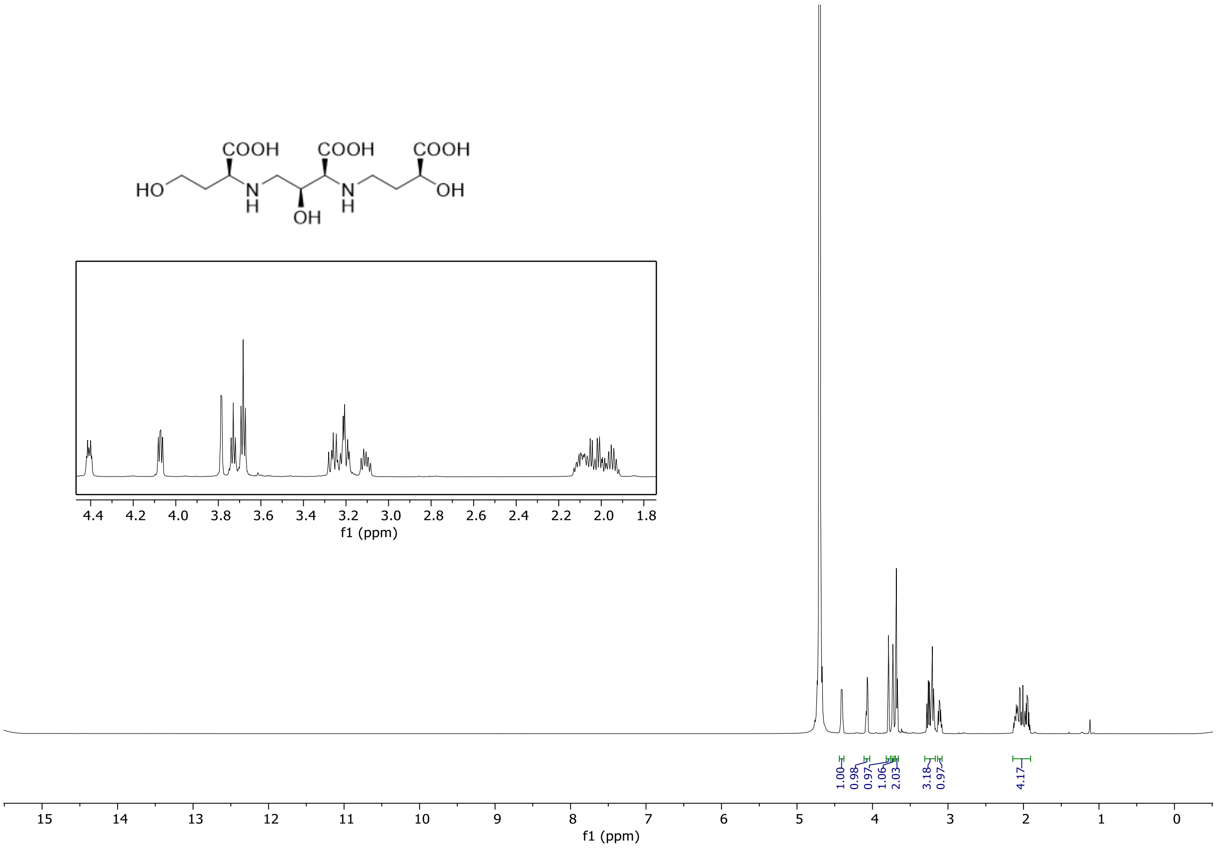

Figure 72: ^{13}C-DEPTQ (151 MHz, D_2O) of hydroxyavenic acid A (**V**)

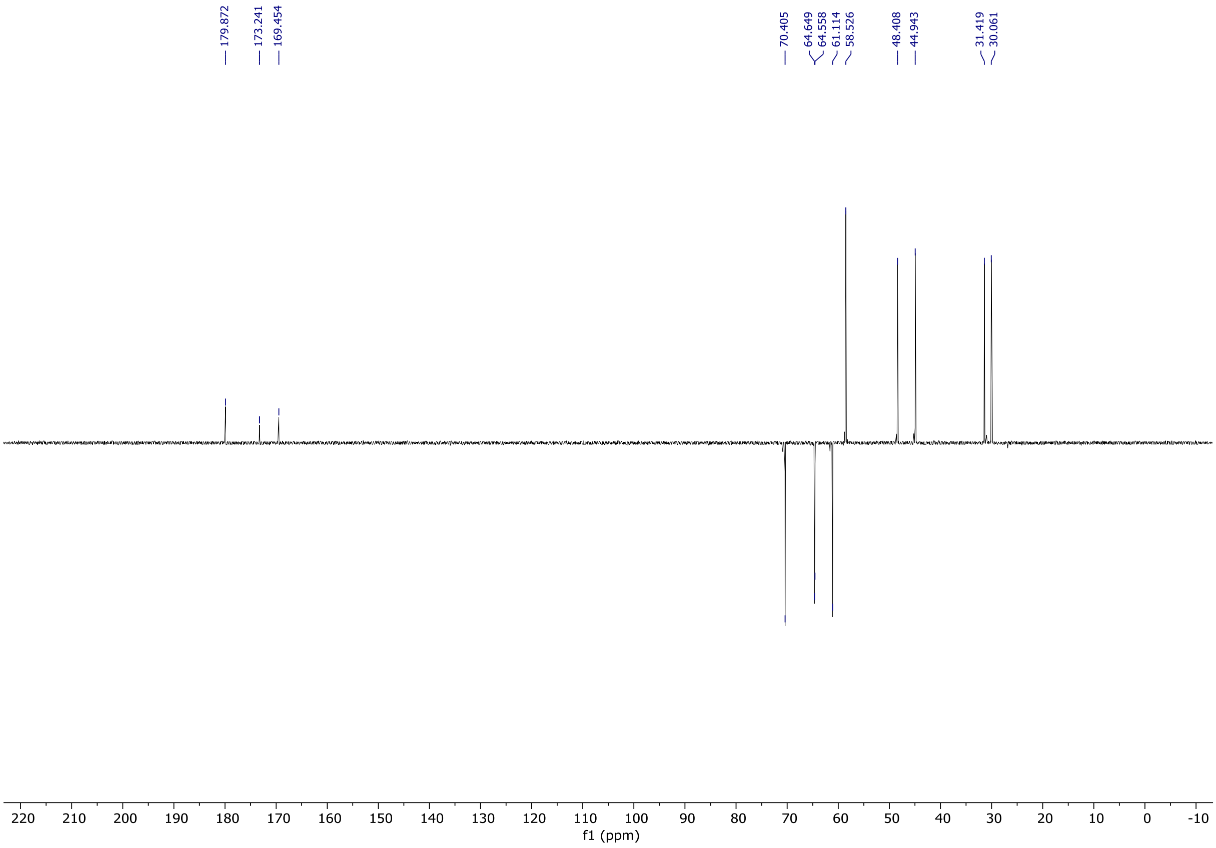

Figure 73: ^{1}H-NMR (600 MHz, D_2O) of deoxymugineic acid (**IV**)

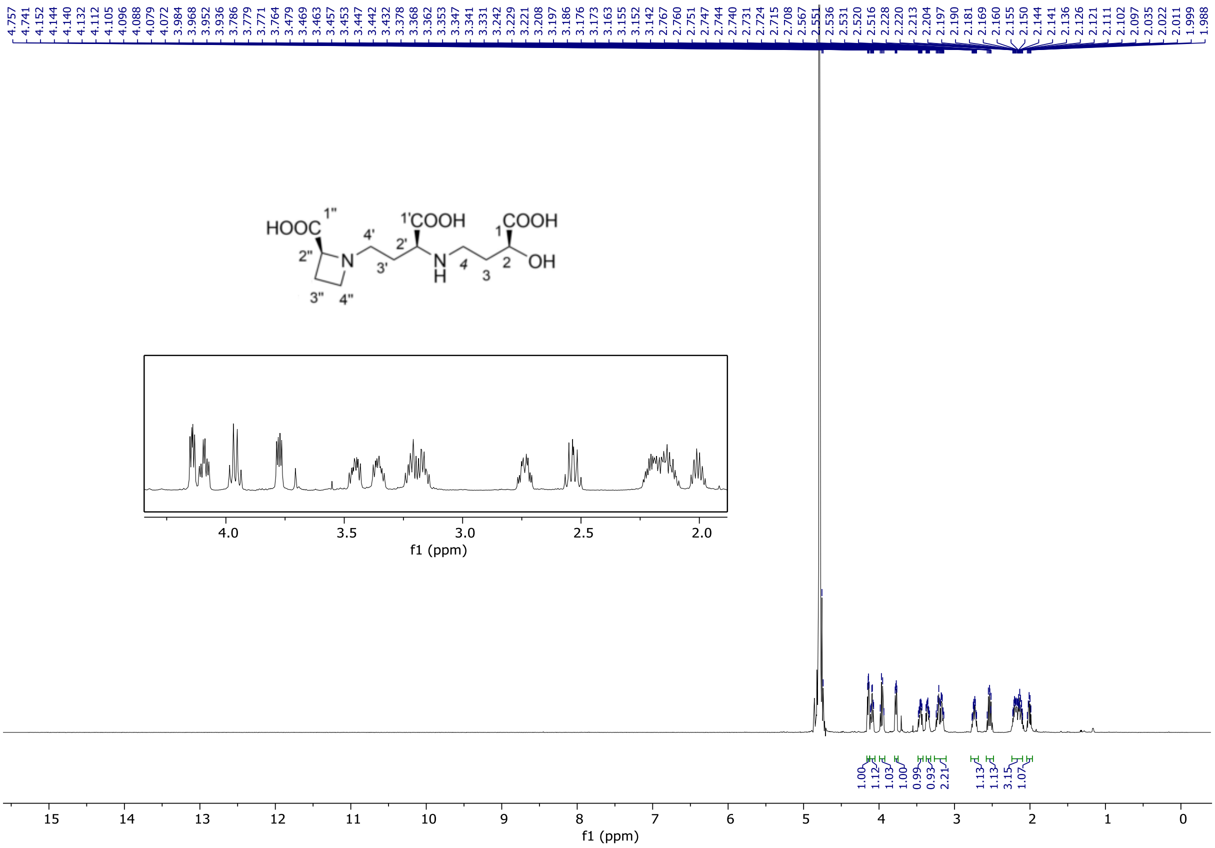

Figure 74: ^{13}C-DEPTQ (151 MHz, D_2O) of deoxymugineic acid (**IV**)

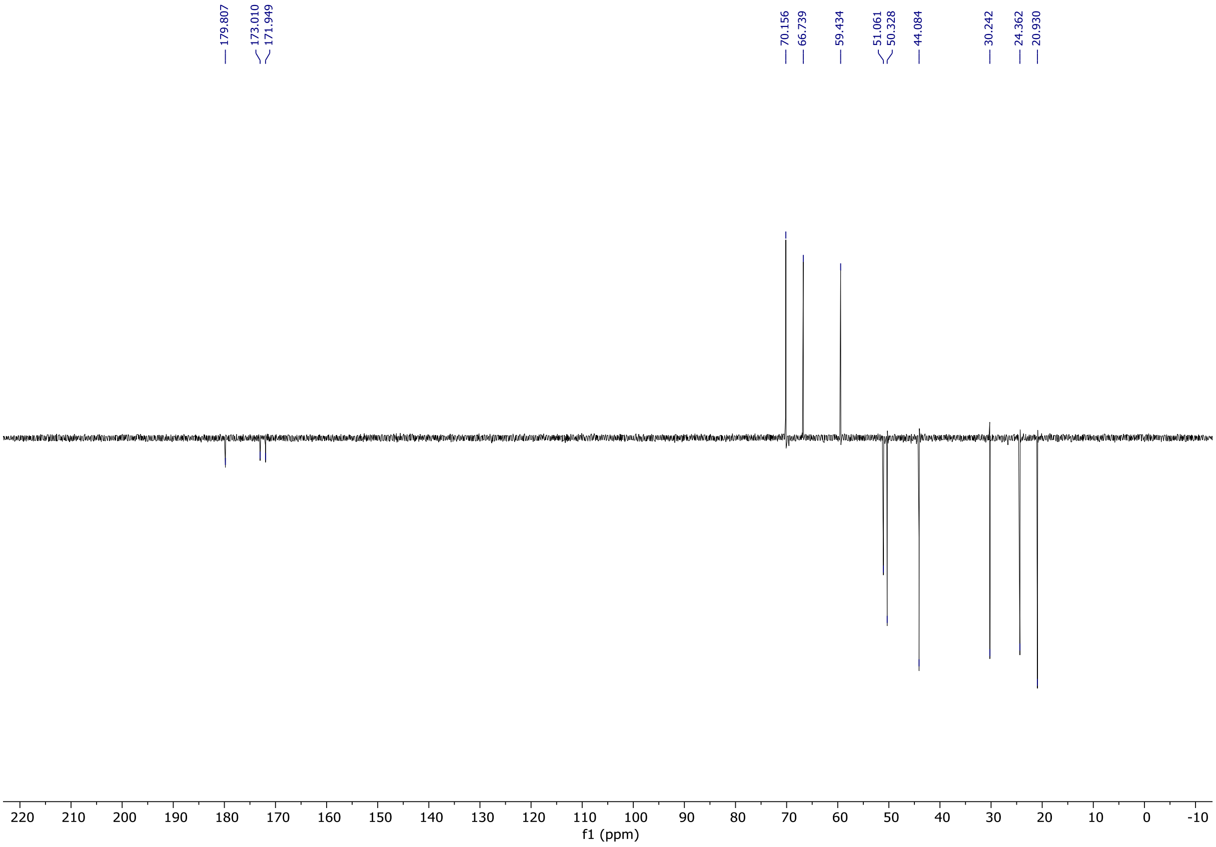

Figure 75: ^{1}H-NMR (600 MHz, D_2O) of 3''-*epi*-hydroxy-deoxymugineic acid (**III**)

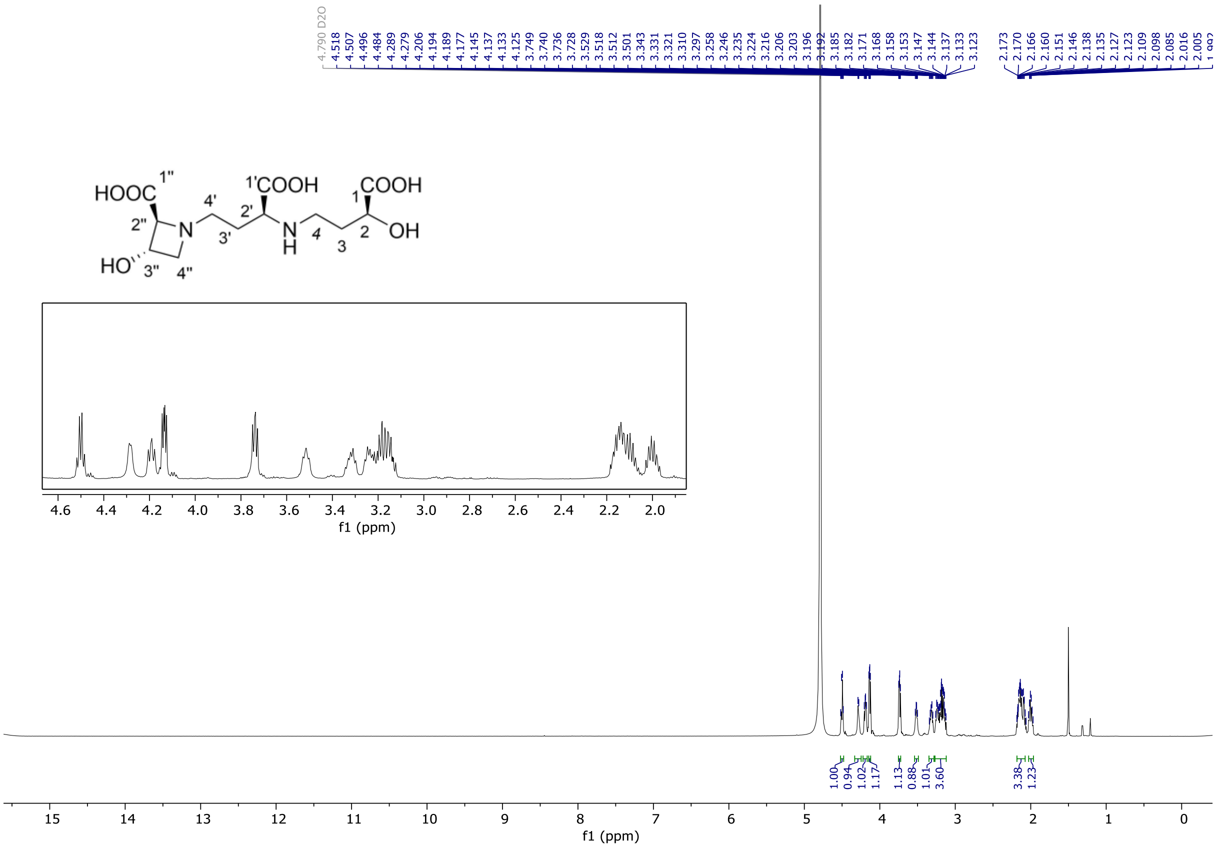

Figure 76: ^{13}C-NMR (151 MHz, D_2O) of 3''-*epi*-hydroxy-deoxymugineic acid (**III**)

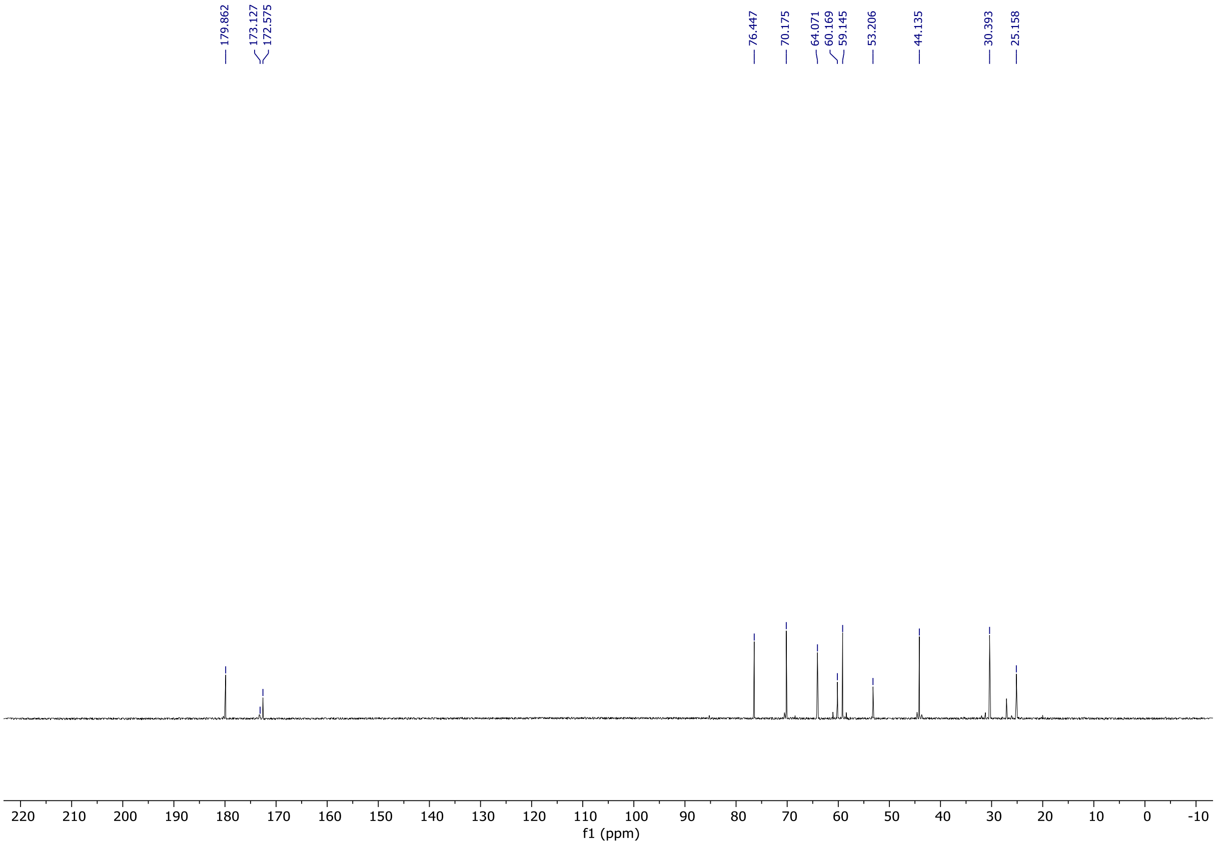

Figure 77: ^{1}H-NMR (600 MHz, D_2O) of 3''-hydroxy-deoxymugineic acid (**III**)

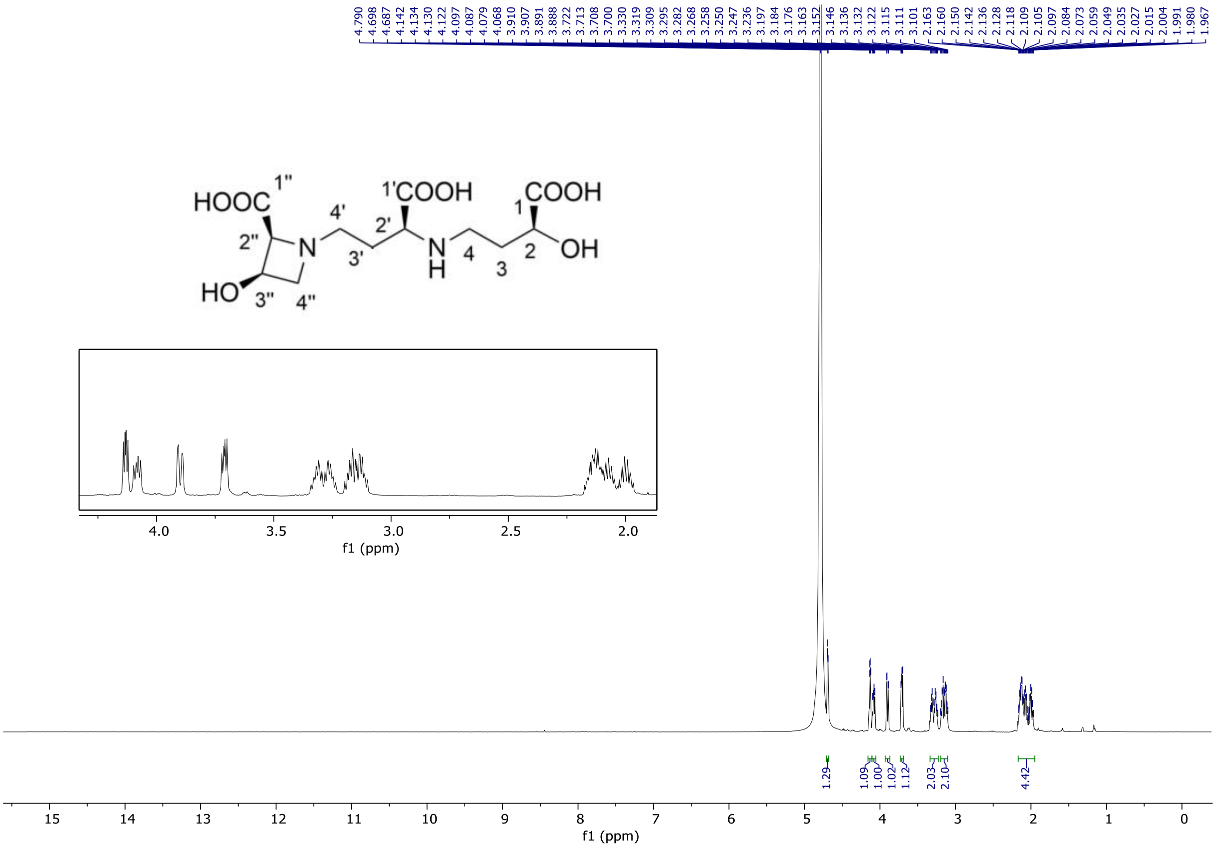

Figure 78: ^{13}C-NMR (151 MHz, D_2O) of 3''-hydroxy-deoxymugineic acid (**III**)

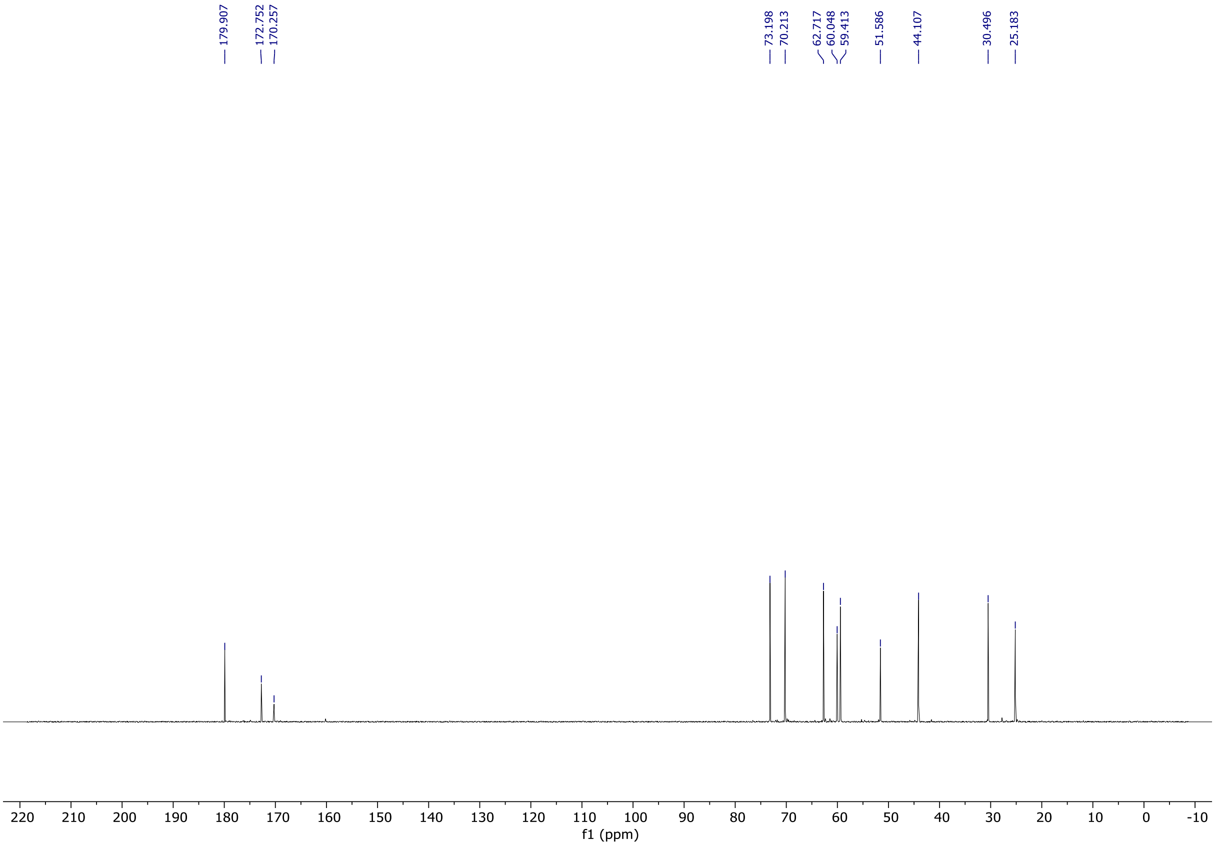

Figure 79: ^{1}H-NMR (600 MHz, D_2O) of (*S*)-4-(((*S*)-1-carboxy-3-(((*S*)-2-oxotetrahydrofuran-3-yl)amino)propyl)amino)-2-hydroxybutanoic acid (**47**)

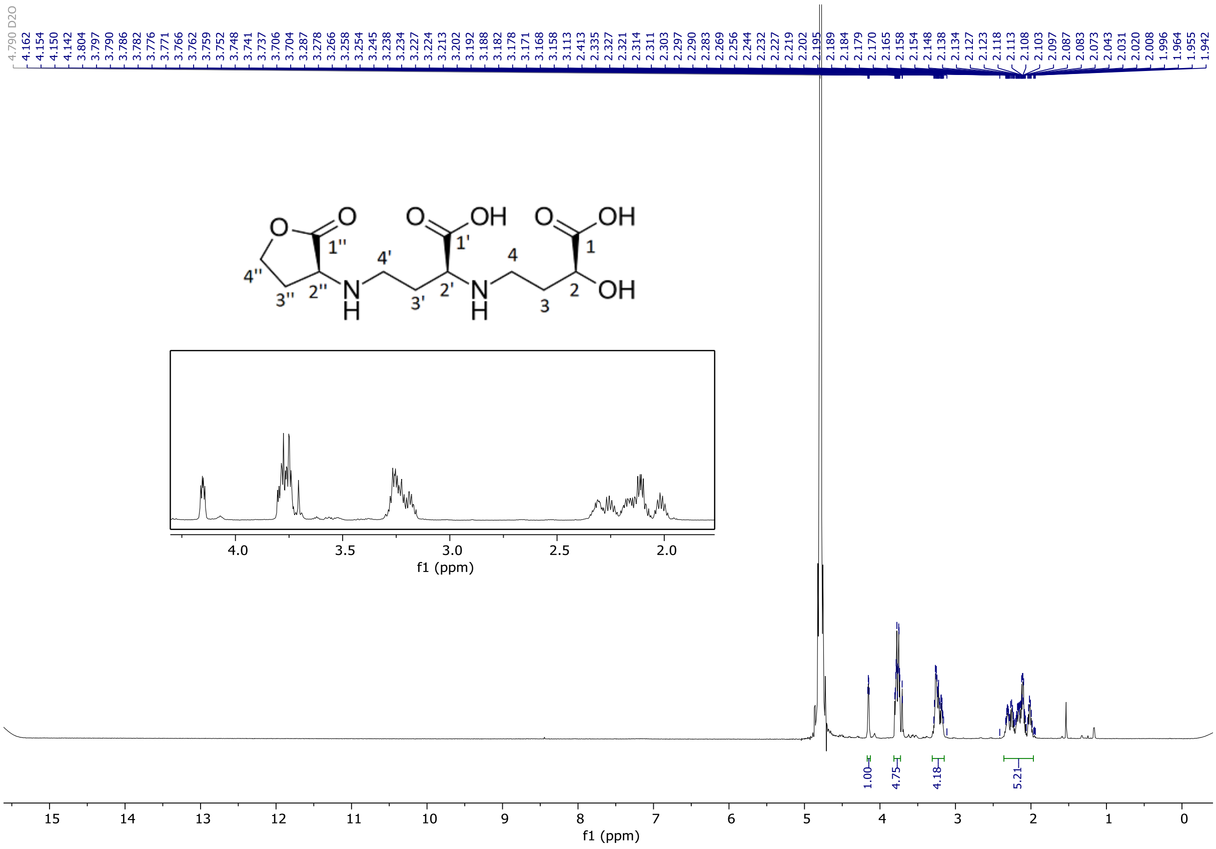

Figure 80: ^{13}C-NMR (151 MHz, D_2O) of (*S*)-4-(((*S*)-1-carboxy-3-(((*S*)-2-oxotetrahydrofuran-3-yl)amino)propyl)amino)-2-hydroxybutanoic acid (**47**)

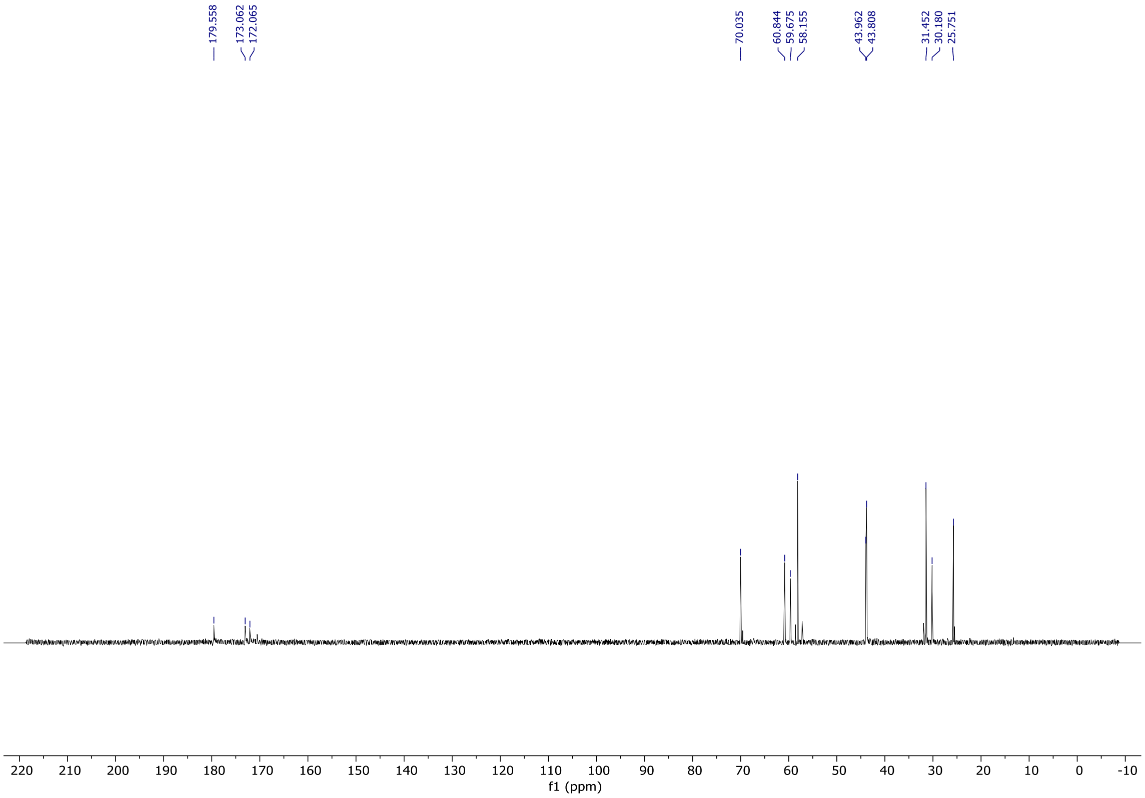

Figure 81: ^{1}H-NMR (600 MHz, D_2O) of avenic acid A (**I**)

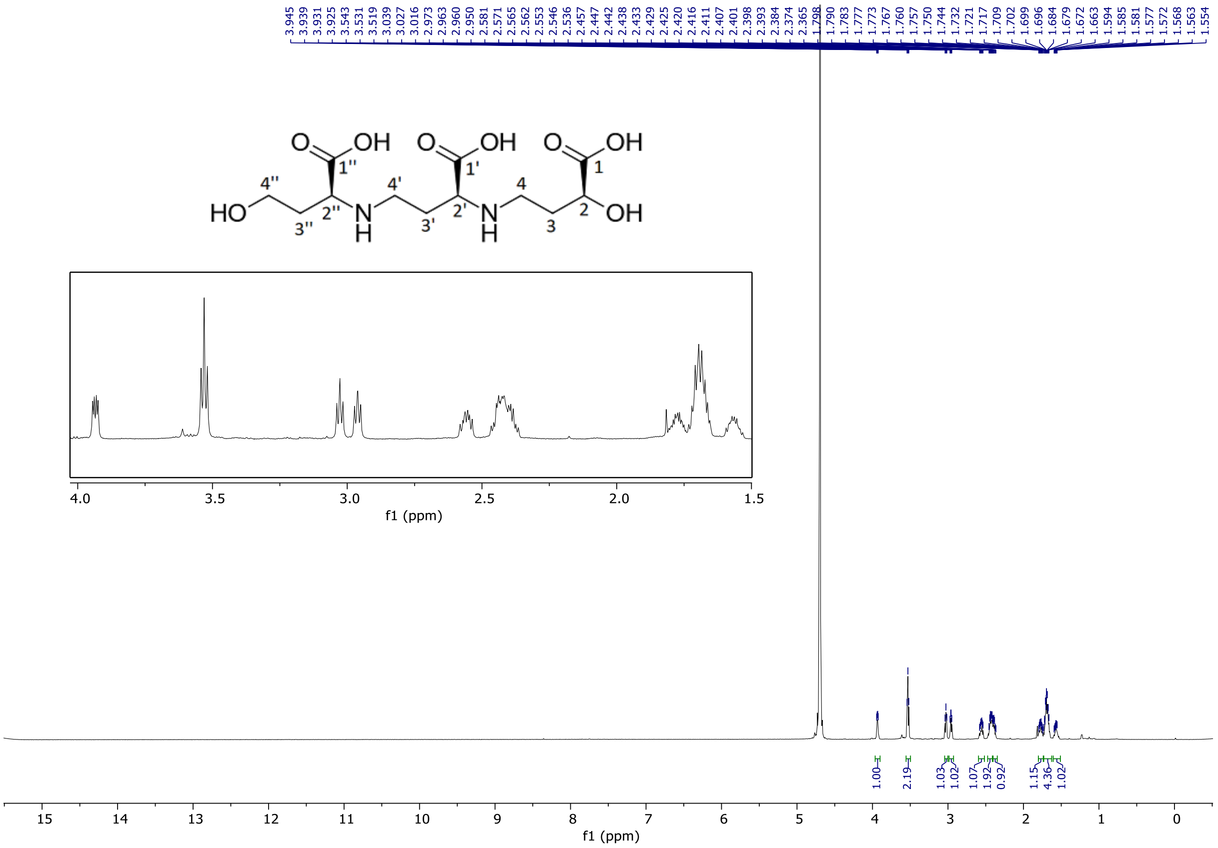

Figure 82: ^{13}C-NMR (151 MHz, D_2O) of avenic acid A (**I**)

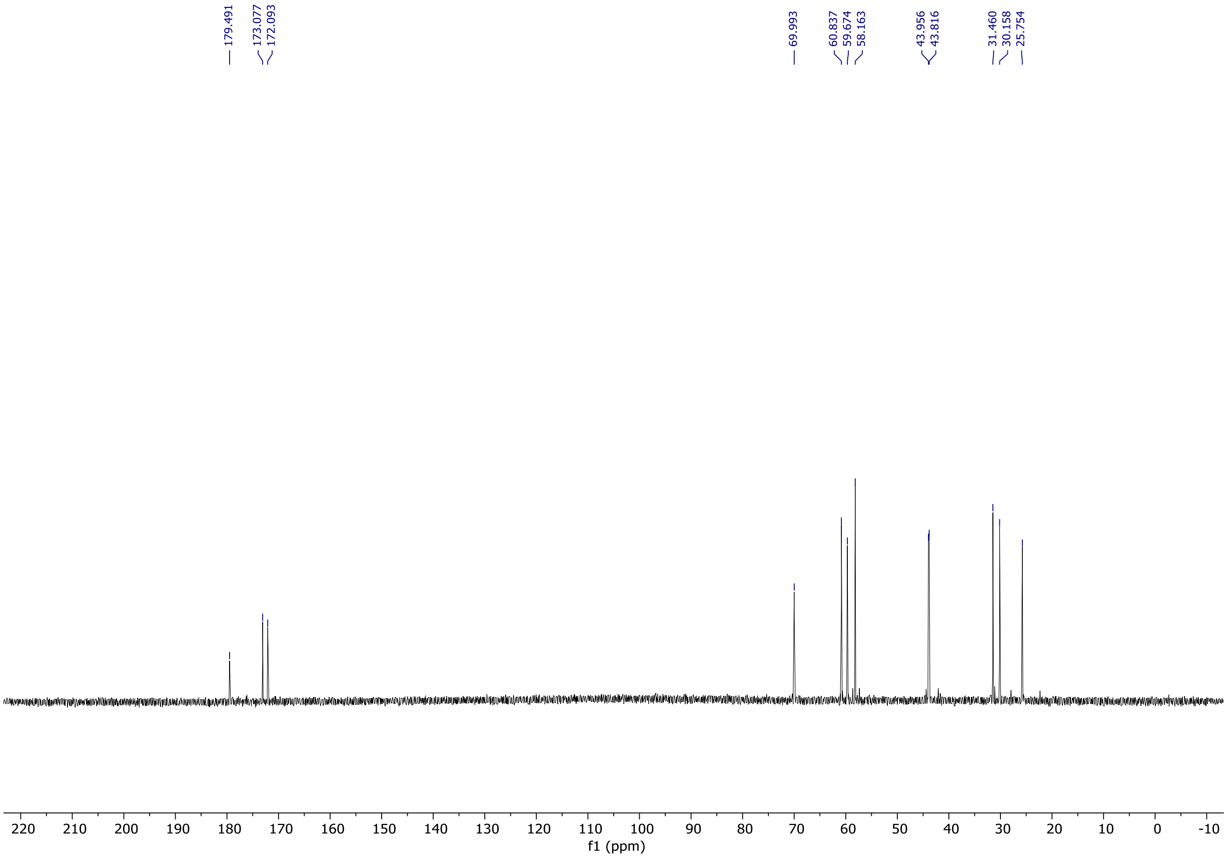

References

1. D. B. Collum, J. H. McDonald III, W. C. Still, *J. Am. Chem. Soc.* **1980**, 102, 2118 – 2120.
2. M. R. Walter, D. Artner, C. Stanetty, *J. Labelled Compd. Radiopharm*. **2014**, 57, 710 – 714.
3. K. Namba, Y. Murata, M. Horikawa, T. Iwashita, S. Kusumoto, *Angew. Chem. Int. Ed.* **2007**, 46, 7060 – 7063.
4. T.-L. Yeh, C.-C. Liao, B.-J. Uang, *Tetrahedron*, **1997**, 53, 11141 – 11152.
5. T. Oguri, N. Kawai, T. Shioiri, S.-I. Yamada, *Chem. Pharm. Bull.* **1978**, 26, 803 – 808.
6. C. Gravier-Pelletier, M. Milla, Y. L. Merrer, J. -C. Depezay, *Eur. J. Org. Chem.* **2001**, *2001*, 3089 – 3996.
7. U. Kazmaier, S. Pähler, R. Endermann, D. Häbich, H.-P. Kroll, B. Riedl, *Bioorg. Med. Chem.* **2002**, 10, 3905 – 3913.
8. F. Matsuura, Y. Hamada, T. Shioiri, *Tetrahedron* **1993**, 49, 8211 – 8222.
9. G. He, Y. Zhao, S. Zhang, C. Lu, G. Chen, *J. Am. Chem. Soc.* **2012**, 134, 3 – 6.
10. A. Turockin, R. Honeker, W. Raven, P. Selig, *J. Org. Chem*. **2016**, 81, 4516 – 4529.
11. F. Matsuura, Y. Hamada, T. Shioiri, *Tetrahedron* **1994**, 50, 265 – 271.
12. S. Fushiya, Y. Sato, S. Nozoe, K. Nomoto, T. Takemoto, S.-I. Takagi, *Tetrahedron Lett.* **1980**, 21, 3071 – 3072.
